# Comparison of pathway and gene-level models for cancer prognosis prediction

**DOI:** 10.1186/s12859-020-3423-z

**Published:** 2020-02-28

**Authors:** Xingyu Zheng, Christopher I. Amos, H. Robert Frost

**Affiliations:** 1 Department of Biomedical Data Science, Geisel School of Medicine, Dartmouth College, Hanover, NH 03755 USA; 2 Department of Medicine, Baylor College of Medicine, Institute for Clinical and Translational Research, 1 Baylor Plaza, Houston, TX 77030 USA

**Keywords:** Cancer prognosis prediction, Gene expression data, Pathway analysis, L1 penalized regression model, Inter-gene correlation

## Abstract

**Background:**

Cancer prognosis prediction is valuable for patients and clinicians because it allows them to appropriately manage care. A promising direction for improving the performance and interpretation of expression-based predictive models involves the aggregation of gene-level data into biological pathways. While many studies have used pathway-level predictors for cancer survival analysis, a comprehensive comparison of pathway-level and gene-level prognostic models has not been performed. To address this gap, we characterized the performance of penalized Cox proportional hazard models built using either pathway- or gene-level predictors for the cancers profiled in The Cancer Genome Atlas (TCGA) and pathways from the Molecular Signatures Database (MSigDB).

**Results:**

When analyzing TCGA data, we found that pathway-level models are more parsimonious, more robust, more computationally efficient and easier to interpret than gene-level models with similar predictive performance. For example, both pathway-level and gene-level models have an average Cox concordance index of ~ 0.85 for the TCGA glioma cohort, however, the gene-level model has twice as many predictors on average, the predictor composition is less stable across cross-validation folds and estimation takes 40 times as long as compared to the pathway-level model. When the complex correlation structure of the data is broken by permutation, the pathway-level model has greater predictive performance while still retaining superior interpretative power, robustness, parsimony and computational efficiency relative to the gene-level models. For example, the average concordance index of the pathway-level model increases to 0.88 while the gene-level model falls to 0.56 for the TCGA glioma cohort using survival times simulated from uncorrelated gene expression data.

**Conclusion:**

The results of this study show that when the correlations among gene expression values are low, pathway-level analyses can yield better predictive performance, greater interpretative power, more robust models and less computational cost relative to a gene-level model. When correlations among genes are high, a pathway-level analysis provides equivalent predictive power compared to a gene-level analysis while retaining the advantages of interpretability, robustness and computational efficiency.

## Background

Cancer prognosis prediction is an important research goal since prognosis is often heterogeneous among patients [1]. Using prognostic biomarkers such as overexpression of HER2 for breast cancer [2], it is possible to subgroup patients for certain types of cancer into populations with distinct risk profiles and design optimal treatment regimens for the heterogeneous subtypes of cancer [3]. In cases where mutation-based biomarkers do not exist, researchers have explored prognostic models based on tumor gene expression data. The proposed methods for selecting biomarkers include using univariate gene selection [4], penalized Cox regression [5, 6], supervised principal component analysis [7], partial least squares algorithm [8] and some other machine learning techniques such as Random Forest [9]. While some expression-based models such as Oncotype Dx have been successful, these models can be difficult to implement due to high cost, limited performance and complexity in their interpretation. One of the main shortcomings of past studies has been the failure to incorporate prior biological information into the predictive model [10]. With the advent of high-throughput profiling technologies, there exists a new challenge of extracting information from a huge number of expressed genes and proteins. One approach to this challenge has been to group genes by biological functions into smaller sets of pathways, a process that is called pathway analysis or gene set testing [11]. Compared to an analysis using separate variables for each profiled gene, a pathway analysis can yield greater statistical power, improved replication and superior interpretation [12]. Analysis at the functional pathway level can reduce the complexity and dimensions of the gene expression data resulting in increased power due to fewer tested hypotheses and improved replication of results across independent data sets. Pathway-level variables are also more readily interpreted since they represent biologically meaningful groups of genes, e.g., the genes involved in a specific signaling pathway or the genes whose expression is upregulated in response to a specific chemical perturbation.

Pathway analysis methods help cancer researchers identify the biological function of genes and gene sets within malignant tissues and thereby inform the design of new cancer therapies [13]. Most pathway analysis methods, such as GSEA [14] and Camera [15], operate on a population level, that is, they compute a single statistic for each pathway that captures the association of the genes in the pathway with an outcome of interest such as case/control status or survival. More recently, researchers have developed so-called single sample pathway analysis methods that compute a separate pathway statistic for each sample. These methods can transform a gene expression data set for *n* samples and *p* genes that is represented by a *n* × *p* matrix into an *n* × *m* matrix of single sample scores for *m* pathways. Similar to population-based gene set testing methods, single sample techniques can be broadly grouped into supervised and unsupervised categories. In this context, supervised methods evaluate the association of gene sets with a specific outcome variable whereas unsupervised methods ignore any sample label information. Examples of recent single sample methods include ASSESS [16], GSVA [17], PARADIGM [18] and Pathifier [19]. ASSESS [16] utilizes density estimates in the calculation of sample scores relative to a binary outcome. GSVA [17] also utilizes density estimates but computes pathway scores in an unsupervised fashion without regard for sample label information. PARADIGM [18] is also an unsupervised method that uses a probabilistic graphical model to integrate multi-omics data and infer the altered pathway activity of individual patients. Pathifier [19] is a supervised method and calculates the scores by measuring the deviation of each sample from a specified baseline condition. The pathway-level variables produced from such single sample techniques can be used as predictors in regression models to enable applications such as cancer prognosis prediction for individuals on a pathway level.

Various studies have focused on the application of pathway-based models for cancer prognosis prediction [12, 20, 21]. Huang et al. [20] have developed a novel computational model for breast cancer prognosis by combining the Pathifier, Cox regression and Lasso penalization. Liang et al. [21] developed a pathway-based prognosis prediction model for glioblastoma using a combination of univariate and multivariate Cox regression analysis, Pathifier, and Lasso penalization. Sinnott et al. [12] proposed multiple kernel learning methods to select informative pathways and aggregate their signals for prediction of censored survival outcomes and applied them under the Cox proportional hazards and semiparametric accelerated failure time models. Although various pathway-based prognostic models have been developed, a comprehensive comparison of pathway-level and gene-level prognostic models has not been performed. To address this gap, we sought to characterize the performance of both pathway and gene-level cancer prognostic models across a range of realistic gene expression structures to identify the specific range in the cancer transcriptomic landscape where either a gene-level model or pathway-level model can be expected to provide superior performance.

## Methods

### Data sources

We downloaded gene expression and clinical data from the UCSC Xena datahub [22] for 34 cohorts profiled by The Cancer Genome Atlas (TCGA) [23]. Among the 34 cohorts, 4 cohorts (Bile Duct Cancer cohort (CHOL), Formalin Fixed Paraffin-Embedded Pilot Phase II cohort (FPPP), Large B-cell Lymphoma cohort (DLBC) and Uterine Carcinosarcoma cohort (UCS)) were discarded because of an insufficient number of samples with gene expression data. We also analyzed three combinations of the subtype cohorts: colon and rectum adenocarcinoma (COADREAD), which is the combination of the colon adenocarcinoma (COAD) and rectum adenocarcinoma (READ) datasets, brain lower grade glioma and glioblastoma multiforme (GBMLGG), which is the combination of the brain lower grade glioma (LGG) and glioblastoma multiforme (GBM) datasets and lung cancer (LUNG), which is the combination of the lung squamous cell carcinoma (LUSC) and lung adenocarcinoma (LUAD) datasets. After these modifications, a total of 33 cohorts remained for analysis. The full list of these cohorts and the corresponding sample sizes, death rates and predictive performance results are provided in Table 1.

**Table 1:** Predictive performance results for all 33 analyzed TCGA cohorts. For each cohort, the table includes the sample size (*n*), death rate and average Cox concordance index values for both the gene-level (GLv CI) and pathway-level (PLv CI) models

	Full name	n	Death rate	GLv CI	PLv CI
ACC	Adrenocortical Carcinoma	79	0.37	0.77	0.77
UVM	Ocular Melanoma	80	0.29	0.74	0.70
MESO	Mesothelioma	87	0.84	0.70	0.71
KICH	Kidney Chromophobe	91	0.13	0.53	0.59
READ	Rectal Cancer	105	0.17	0.48	0.53
THYM	Thymoma	122	0.07	0.57	0.51
TGCT	Testicular Cancer	156	0.03	0.48	0.57
GBM	Glioblastoma	172	0.78	0.52	0.54
LAML	Acute Myeloid Leukemia	173	0.60	0.61	0.55
PAAD	Pancreatic Cancer	183	0.55	0.59	0.60
PCPG	Pheochromocytoma and Paraganglioma	187	0.04	0.52	0.51
ESCA	Esophageal Cancer	196	0.41	0.49	0.50
UCEC	Endometroid Cancer	201	0.16	0.50	0.48
SARC	Sarcoma	265	0.38	0.66	0.62
CESC	Cervical Cancer	308	0.23	0.65	0.67
OV	Ovarian Cancer	308	0.57	0.50	0.50
KIRP	Kidney Papillary Cell Carcinoma	323	0.17	0.79	0.77
COAD	Colon Cancer	329	0.22	0.54	0.49
LIHC	Liver Cancer	423	0.39	0.65	0.65
BLCA	Bladder Carcinoma	426	0.45	0.60	0.60
COADREAD	Colon and Rectal Cancer	434	0.21	0.53	0.55
STAD	Stomach Cancer	450	0.33	0.53	0.58
SKCM	Skin Cutaneous Melanoma	474	0.47	0.51	0.49
LGG	Lower Grade Glioma	530	0.25	0.82	0.77
PRAD	Prostate Cancer	550	0.02	0.47	0.47
LUSC	Lung Squamous Cell Carcinoma	553	0.45	0.52	0.52
HNSC	Head and Neck Cancer	566	0.45	0.57	0.59
THCA	Thyroid Cancer	572	0.03	0.51	0.56
LUAD	Lung Adenocarcinoma	576	0.34	0.61	0.63
KIRC	Kidney Clear Cell Carcinoma	606	0.36	0.68	0.66
GBMLGG	Lower grade glioma and glioblastoma	702	0.54	0.85	0.83
LUNG	Lung Cancer	1129	0.40	0.57	0.57
BRCA	Breast Cancer	1218	0.16	0.63	0.61

The Hallmark pathway collection, a set of 50 well characterized biological pathways, was obtained from the Molecular Signatures Database (MSigDB) version 6.2 [14]. For all analyses, we only kept genes existing in both the TCGA gene expression data and MSigDB pathways. We also discarded one gene, which was missing all expression values in the TCGA.

### Prognostic models

In this study we used penalized Cox proportional hazards models with either gene-level or pathway-level predictors as the prognostic models.

**Fig. 1:**
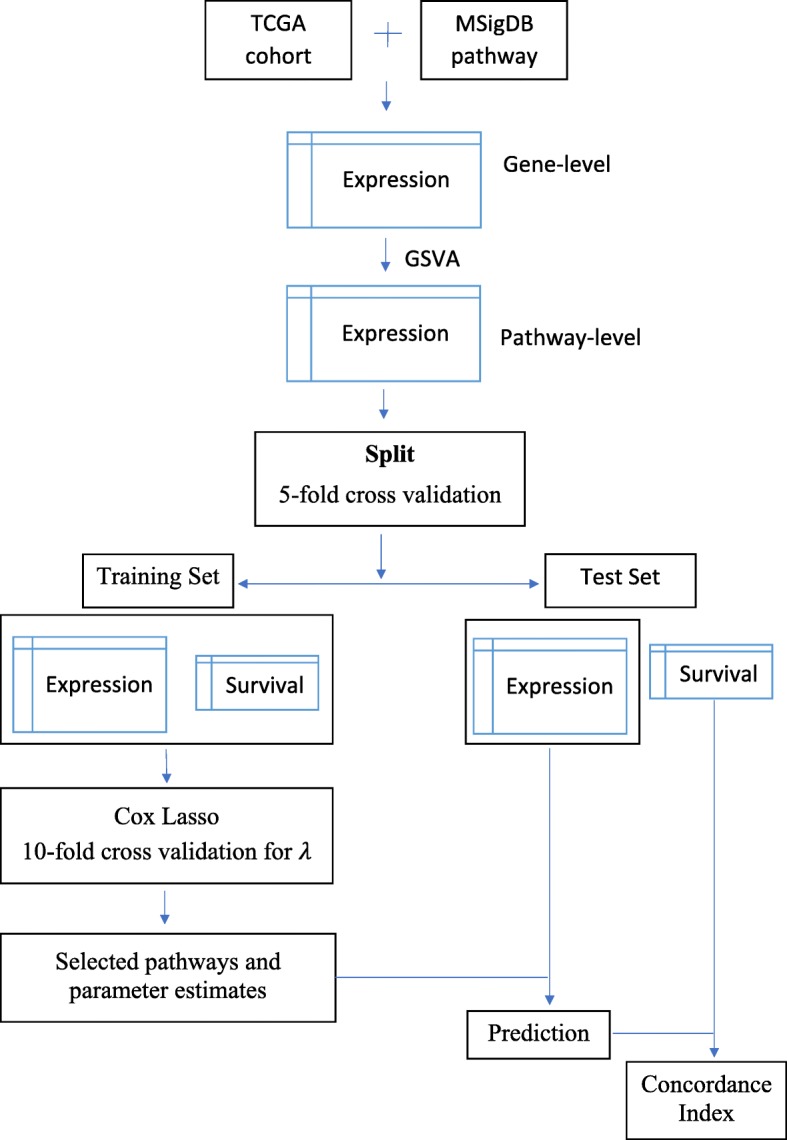
Workflow for pathway-level models. In this study, TCGA was used as the source of gene expression data and MSigDB as the source of pathway definitions. The first step of the workflow converts the gene-level expression data matrix into pathway-level variables via the unsupervised single sample gene set method GSVA. After obtaining a pathway-level data matrix, nested cross validation was used to train and evaluate a Lasso-penalized Cox proportional hazards model. Cross validation was employed both for the training vs. test split and within each training fold for selection of the Lasso penalty parameter. With the selected pathways and estimated parameters, we performed prediction on the test data subset by applying the Cox proportional hazards regression model that had been identified in the training data subset

Our workflow for pathway-level models, illustrated in Fig. 1, uses the single sample pathway scores generated from tumor gene expression data to perform survival prediction. Figure 1 shows TCGA as the source of gene expression data and MSigDB as the source of pathway definitions, but our approach is not limited to these sources. One can easily conduct this workflow on other cancer gene expression data sets and gene set collections.

The first step of the workflow converts the gene-level expression data matrix into pathway-level variables via the unsupervised single sample gene set method GSVA (Gene Set Variation Analysis) [17]. After obtaining a pathway-level data matrix, nested cross validation was used to train and evaluate a Lasso-penalized Cox proportional hazards model. Cross validation was employed both for the training vs. test split and within each training fold for selection of the Lasso penalty parameter. The standard Lasso method [24] solves the *L*_1_-penalized regression problem by minimizing the expression:

1 $$ {\sum}_{i=1}^n{\left({y}_i-{\sum}_{j=1}^p{x}_{ij}{\beta}_j\right)}^2+\lambda {\sum}_{j=1}^p\left|{\beta}_j\right|={\sum}_{i=1}^n{\left({y}_i-{\sum}_{j=1}^p{x}_{ij}{\beta}_j\right)}^2+\lambda {\left\Vert \beta \right\Vert}_1 $$

*x*_*ij*_ denotes the observed value of *j* th variable (*j* = 1, …, *p*) for the *i* th subject (*i* = 1, …, *n*). *y*_*i*_ denotes the observed centered outcome of subject *i* and ‖∙‖_1_ denotes the *L*_1_-norm. The *L*_1_-penalty shrinks some of the estimated coefficients to 0 and the amount of shrinkage is determined by the parameter *λ*, which is in practice most often chosen using cross-validation. This model was generalized to Cox proportional hazards regression (in the case of a censored time to event data) by replacing the term ${\sum}_{i=1}^n{\left({y}_i-{\sum}_{j=1}^p{x}_{ij}{\beta}_j\right)}^2$ with −*l*(*β*, *γ*), where *l*(., .) stands for the log-likelihood function and *γ* for the intercept [25].

With the selected pathways and estimated parameters, we performed prediction on the test data subset by applying the Cox proportional hazards regression model that had been identified in the training data subset.

**Fig. 2:**
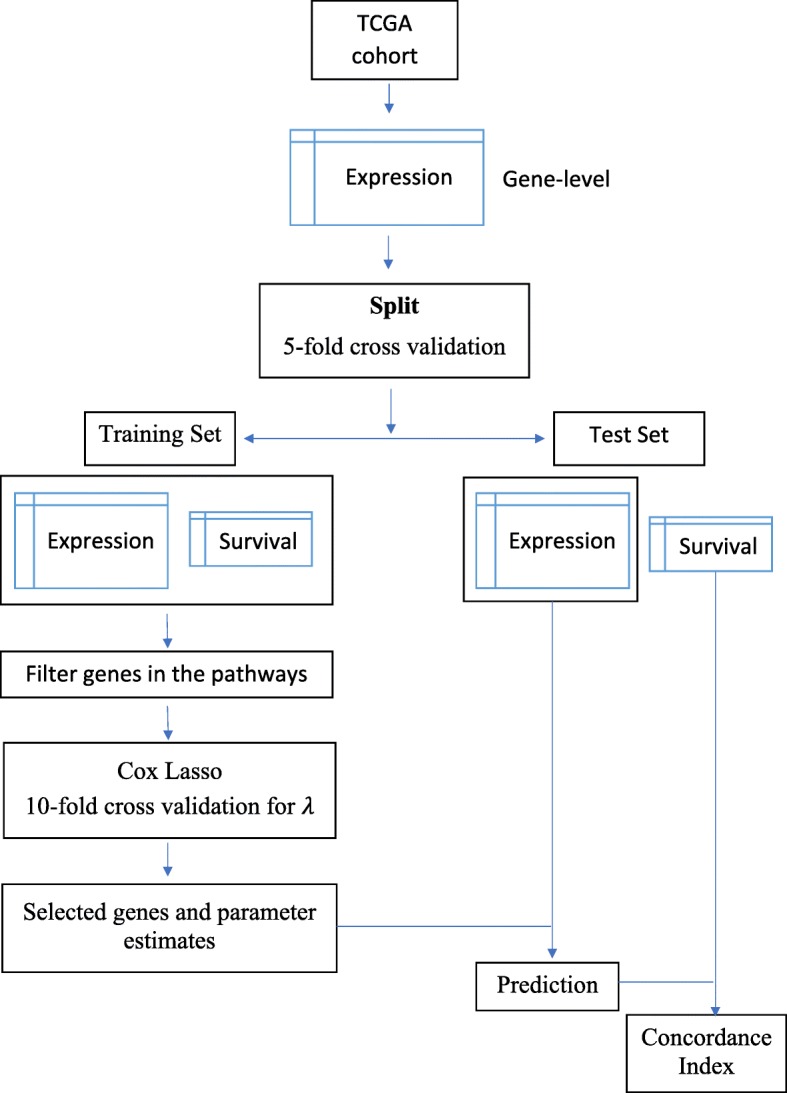
Workflow for gene-level models. In this study, TCGA was used as the source of gene expression data. The expression data used for the gene-level models was filtered to only contain the genes mapped to the pathways considered for the pathway-level models. Nested cross validation was used to train and evaluate a Lasso-penalized Cox proportional hazards model. Cross validation was employed both for the training vs. test split and within each training fold for selection of the Lasso penalty parameter. With the selected genes and estimated parameters, we performed prediction on the test data subset by applying the Cox proportional hazards regression model that had been identified in the training data subset

Performance of the pathway-level models was evaluated relative to gene-level models. The gene-level workflow is illustrated in Fig. 2. Similar to the pathway-level workflow, Lasso-penalized Cox models were trained and evaluated using nested cross validation. The expression data used for the gene-level models was filtered to only contain the genes mapped to the pathways considered for the pathway-level models.

### Simulation based on TCGA expression data and MSigDB pathways

To investigate the relative cancer prognostic performance of the gene-level and pathway-level models, we designed two simulation studies, which used TCGA gene expression data and simulated survival times that were associated with the expression values for genes belonging to a MSigDB pathway or an equal sized group of randomly selected genes. The basic assumption of our simulation studies is that cancer progression operates through dysregulation of key pathways and that all the genes in specific pathways contribute to prognosis and survival. We simulated survival times based on the average expression values of either the genes in a single MSigDB pathway or a randomly selected group of genes, as described in formula 2 below; in both cases, the number of genes associated with survival was identical. Simulations were performed using four MSigDB Hallmark pathways, Hallmark estrogen response late (*n* =200, correlation = 0.17 in LGG cohort), Hallmark E2F targets (*n* =193, correlation = 0.34 in LGG cohort), Hallmark TGF beta signaling (*n* =54, correlation = 0.22 in LGG cohort) and Hallmark MYC targets V2 (*n* =58, correlation = 0.27 in LGG cohort) respectively, which were selected to represent the four combinations of large or small pathway size and high or low mean absolute inter-gene correlation. Using these simulated data sets, we evaluated how the size and mean absolute inter-gene correlation of the survival-associated pathway affected the relative performance of the gene-level and pathway-level models. To achieve stable results, we simulated 20 independent sets of survival times for each pathway and noise combination, and for each simulation, we evaluated predicted performance using nested cross validation as detailed above. We used the average concordance index [26], which can have values between 0 and 1, to quantify the predictive power of each model. The concordance index (CI), or c-index, is one of the most commonly used performance measures for survival models and can be interpreted as the fraction of all pairs of subjects where the observation with the higher survival time has the higher probability of survival predicted by the model [27]. A CI of 1 indicates perfect prediction accuracy and a CI of 0.5 represents random prediction. We used the Fleiss kappa statistic [28] to evaluate the repeatability and stability of the models. The kappa statistic is frequently used to test interrater reliability. A Fleiss Kappa statistic of 1 indicates perfect agreement and a value of equal or less than 0 indicates no agreement. Measurement of the extent to which raters assign the same score to the same variable is called the interrater reliability [29]. In our situation, each trained model is a rater that is assigning each variable (gene or pathway) to either belong or not belong to the model. We conducted 20 replications of 5-fold cross validation so had 100 trained models, or 100 raters, in total. We used the average number of predictors in the 100 trained models to measure model parsimony.

In the first simulation study, we only simulated survival times. Specifically, we generated the survival times as:

2 $$ {T}_i={\mathit{\exp}}^{\left(\ \frac{\sum_{j=1}^{j=m}{G}_{ij}}{m}+{\varepsilon}_i+10\right)}/10 $$

where *G*_*ij*_ is the centered and standardized gene expression matrix with *i* = 1. . *n* samples and *j* = 1. . *m* genes which are the genes in the specific pathway. *ε*_*i*_~*N*(0, *σ*) corresponds to random noise added into the simulated survival. The standard deviation parameter *σ* controls the magnitude of noise. The additive value of 10 and the divisor of 10 ensure the generated *T* are around several thousand, which are reasonable survival times in years. To make the censoring time uninformative, we shuffled the generated *T* values to get the censoring times. A sample was considered to be censored when the censoring time was smaller than the survival time. Thus, each sample was randomly censored or dead with an average censoring rate of approximately 0.5. With the TCGA data, MSigDB pathway collection and the simulated survival times, we ran the pathway-level model workflow and gene-level model workflow separately.

In the second simulation study, to investigate how the complex correlation structure of the gene expression data affected predictive performance, we broke the correlation structure of the TCGA data. Specifically, we shuffled each gene row so that the correlations between genes are broken meanwhile the distribution of each gene among samples was kept unchanged. Then we used the same approach to generate the survival times as in the first simulation study and ran the pathway-level model workflow and gene-level model workflow separately.

### Comparative null models

**Fig. 3:**
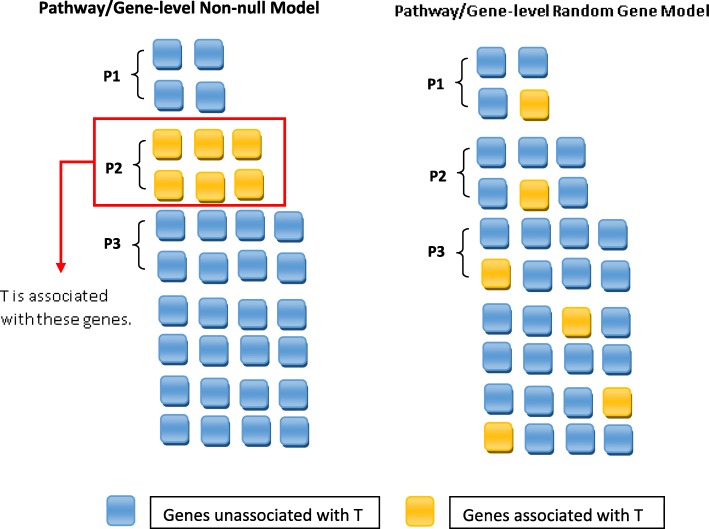
Random gene model design. In the random gene model, the survival time was associated with a group of random genes whose size was equal to the size of the pathway that was associated with survival time in the non-null model

To ensure the signals in the simulation studies were not generated randomly and that the prediction accuracy was not inflated in our analyses, we designed two comparative null models to evaluate the performance of pathway-level and gene-level models, which are referred to as the random gene model and the null model. In the random gene model, as illustrated in Fig. 3, we associated the survival time with random genes with the number of random genes equal to the size of the pathway that was associated with survival time in the non-null model. In this case, *j* (*j* = 1. . *m*) in Eq. 2 are randomly selected genes so that the pathway is no longer associated with survival. In the null model, survival is independent of the gene expression data. Specifically, we permuted survival times *T* after generating them using Eq. 2 to break the association between *T* and the gene expression data.

### Evaluating TCGA expression and survival data

We ran both the pathway-level model workflow and gene-level model workflow on all the 33 TCGA cohorts using the real survival data and the MSigDB Hallmark pathway collection. Similar to the simulation studies, we evaluated model predictive performance using the concordance index, evaluated interpretative performance by model parsimony, and evaluated model stability using the Fleiss kappa statistic. We also evaluated the biological plausibility of the selected pathway and gene predictors.

## Results

### Simulation study results

**Fig. 4:**
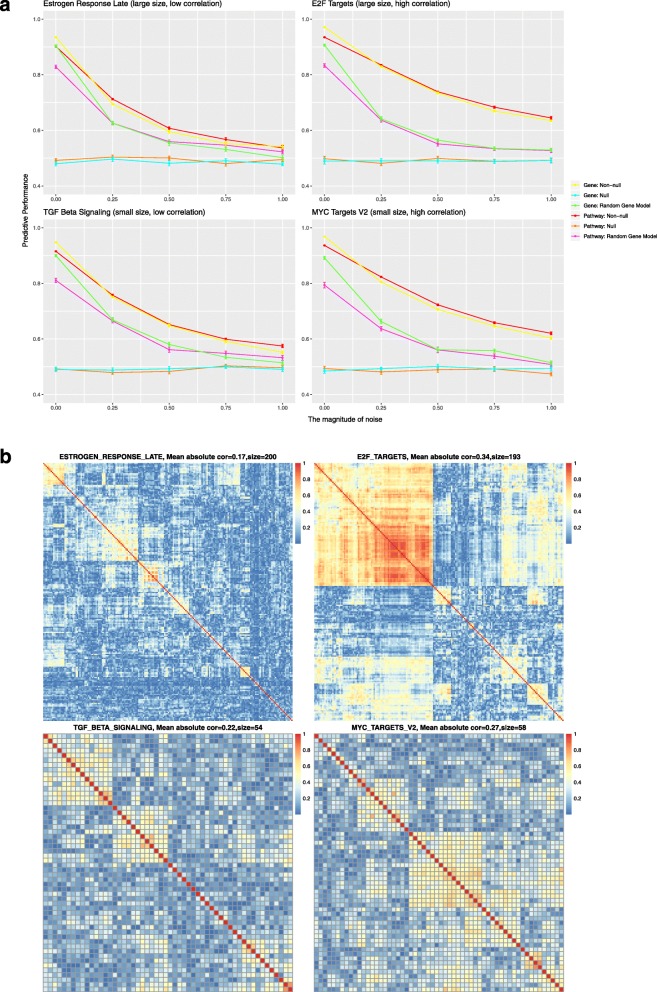
Results of the simulation study based on gene expression data from the LGG cohort and representative pathways from the MSigDB Hallmark collection. **a** Each panel plots the predictive performance of the evaluated gene-level and pathway-level models for simulation studies that associated survival with one of four Hallmark pathways (*Hallmark estrogen response late*, *Hallmark E2F targets*, *Hallmark TGF beta signaling* and *Hallmark MYC targets V2* respectively) selected to represent the four possible combinations of large or small pathway size and high or low average inter-gene correlation. In these plots, the Cox concordance index is plotted on the y-axis with the x-axis representing the standard deviation of the Gaussian noise added to the simulated survival times. The error bars represent the standard error over 20 replications. **b** Heatmaps that represent the inter-gene correlation structure of the four corresponding Hallmark pathways as computed on the LGG cohort gene expression data

Figure 4a displays the results for the first simulation study design using gene expression data from the TCGA low-grade glioma cohort (TCGA LGG cohort, *n* = 530) and survival times simulated from the expression values for each of the four representative MSigDB Hallmark pathways. Figure 4b illustrates the complex correlation structure for these pathways in the LGG cohort. As shown in Fig. 4a, the predictive performance of the pathway-level and gene-level models was similar for all four pathways and all tested simulation models. When noise is not added to the simulated survival times, the average concordance index (CI) can be as high as 0.9 for both models. As the level of added noise was increased, predictive performance decreased until the mean CI was 0.5 (the level consistent with random guessing). Comparison of the results from the large and small pathways demonstrates that the performance of both the gene-level and pathway-level models are not sensitive to pathway size. In contrast, model predictive performance was significantly impacted by the level of inter-gene correlation with both the gene-level and pathway-level models exhibiting better performance and less sensitivity to noise when the correlation among gene expression values was high.

**Fig. 5:**
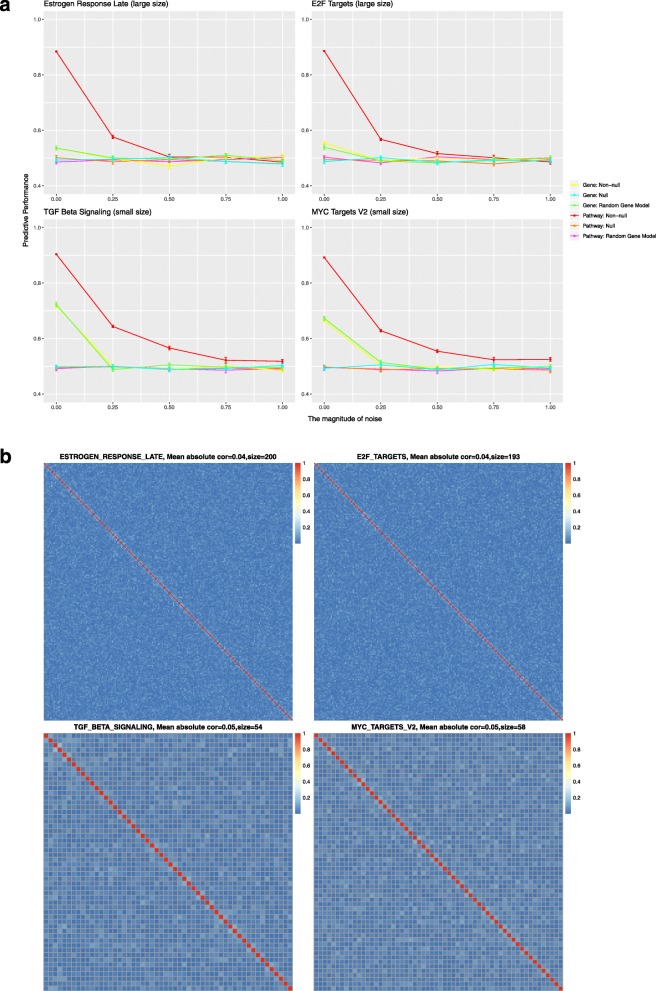
Results of the simulation study based on gene expression data from the TCGA LGG cohort without inter-gene correlation and representative pathways from the MSigDB Hallmark collection. **a** The correlation in the gene expression data has been broken by randomly permuting the values for each gene. Each panel plots the predictive performance of the evaluated gene-level and pathway-level models for simulation studies that associated survival with one of four Hallmark pathways (*Hallmark estrogen response late*, *Hallmark E2F targets*, *Hallmark TGF beta signaling* and *Hallmark MYC targets V2* respectively) selected to represent the four possible combinations of large or small pathway size and high or low average inter-gene correlation. In these plots, the Cox concordance index is plotted on the y-axis with the x-axis representing the standard deviation of the Gaussian noise added to the simulated survival times. The error bars represent the standard error over 20 replications. **b** Heatmaps that represent the lack of inter-gene correlation for the four corresponding Hallmark pathways after random permutation of the gene expression values

Figure 5a displays the results for the second simulation study design, which broke the gene expression correlation structure, using the LGG cohort and the four representative pathways. Figure 5b shows the successful breaking of this correlation structure. Without correlation in the expression data, the performance of gene-level model dropped to a CI of approximately 0.5. The gene-level model only had non-null performance when the pathway size was small and no noise was added to the simulated survival times. Even in this case, performance for the gene-level model drops to 0.5 when the sample size decreases to around 400 as shown in Additional file 1 for other TCGA cohorts. In contrast, the pathway-level model retained good predictive performance (average CI of 0.9 without noise) even in the absence of inter-gene correlation, although performance was more sensitive to noise in this case.

Equivalent simulation results for all 33 TCGA cohorts are included in Additional file 1. The results for the other 32 cohorts follow the same general trends observed for the LGG cohort. To investigate if the choice of pathway used to simulate survival times impacts model performance, we also tested the models on survival times generated using each of the 50 pathways in the MSigDB Hallmark collection; these results are presented in Additional file 2 and are consistent with the results for the four pathways shown in Figs. 4 and 5.

**Fig. 6:**
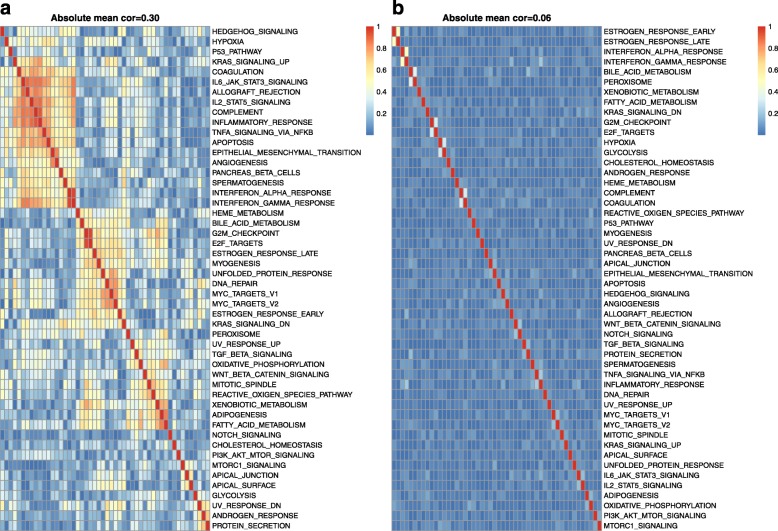
Correlation of single sample pathway scores. **a** Heatmap illustrating the correlation between the GSVA single sample scores for the pathways in the MSigDB Hallmark collection as computed using the TCGA LGG cohort gene expression data. **b** Heatmap illustrating the single sample pathway score correlations after breaking the inter-gene correlation structure

Besides the main findings above, it is worth noting that, in the first simulation study, gene-level models and pathway-level models perform equally well, irrespective of the simulation model (non-null group, random gene group or null group). For each of these groups, the gene-level and pathway-level models were tested on the identical simulated data and, for the first simulation study, perform almost equally. For the null group, it is as expected that both the gene-level and pathway-level models will have c-index values of close to 0.5 given the lack of association between the gene expression data and survival outcomes in the simulated data. For the non-null group, in the simulated data, the survival time is associated with the average expression of all genes in a specific pathway. Therefore, we had expected the pathway-level model to have better predictive performance than gene-level model and were surprised that they in fact had very similar predictive power. We believe this can be explained by the fact that the genes in the MSigDB pathways are highly correlated in the TCGA gene expression data. As shown in Fig. 4b, the lowest average correlation of all genes in a pathway is still around 0.2. This high inter-gene correlation makes it easier for the gene-level model to pick out the associated gene(s) or genes correlated with those associated genes. Our second simulation study showed that without correlation, the gene-level model failed to work, which provides further support for this hypothesis. For the random gene group, in the simulated data, randomly selected genes are associated with survival. Therefore, we had expected the gene-level model to have better predictive performance than pathway-level model and were surprised that performance was in fact very similar. Similar to the non-null group scenario, we believe this can be explained by the complex correlation structure among pathway genes. Figure 6a displays the correlation among pathway scores of the first simulation study. Even though the random genes are less correlated than the genes in a pathway, the associated transformed pathway variables can still be highly correlated. This high inter-pathway correlation makes it easier for the pathway-level model to pick out the associated pathway(s) or pathways correlated with those associated pathways. Our second simulation study showed that without correlation, the pathway-level model failed to work, supporting this hypothesis.

**Fig. 7:**
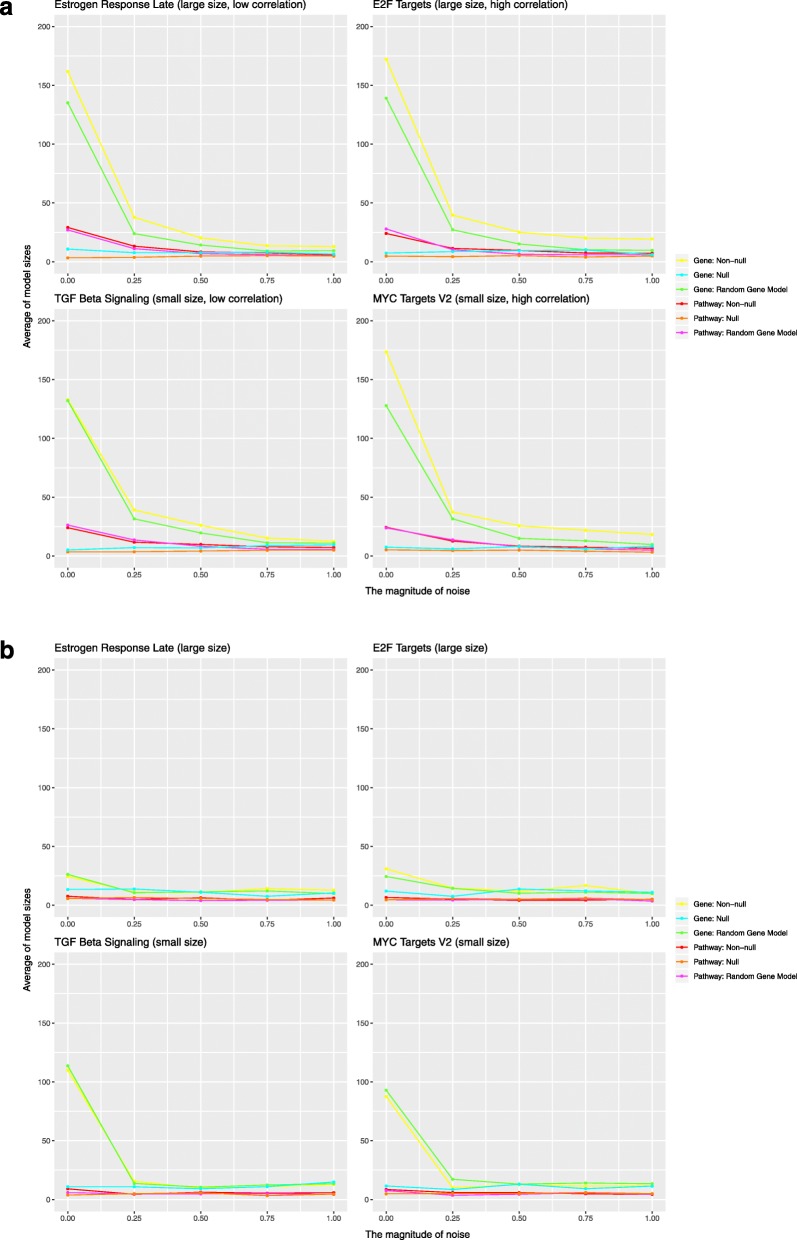
Average number of predictors in the non-null models. **a** Each plot shows the average number of predictors as a function of added noise for the simulation studies that did not alter the inter-gene correlation structure. **b** The average number predictors for the simulation studies where the inter-gene correlation structure was broken

**Fig. 8:**
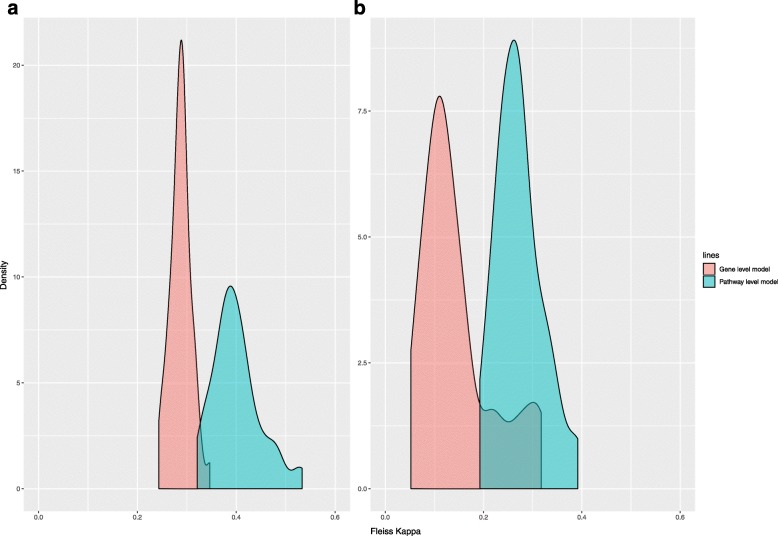
Density distribution of Fleiss Kappa statistics across 50 pathways for the non-null models when there’s no noise in the simulation. Fifty pathways from hallmark collection have been separately used in the non-null model workflows for total 100 runs. Fleiss Kappa was calculated to measure the agreement among these 100 runs. **a** Distribution of Fleiss Kappa in the first simulation study. Pathway-level model has better model stability than gene-level model. **b** Distribution of Fleiss Kappa in the second simulation which broke the inter-gene correlation. Without inter-gene correlation, pathway-level model became more advantageous in model stability compared with gene-level model

In addition to superior predictive performance when inter-gene correlation is low, the pathway-level model has the advantages of model parsimony, stability and computational speed relative to the gene-level model. To evaluate model parsimony, we plotted the average number of predictors in the non-null models, as shown in Fig. 7. Overall, the pathway-level models included far fewer predictors than the gene-level models. Comparing Fig. 7a with Fig. 4a and comparing Fig. 7b with Fig. 5a demonstrates that model predictive performance was associated with the number of predictors in the model, especially for the gene-level models. To evaluate model stability, we calculated Fleiss Kappa statistics for the non-null models with no noise; the distribution of these kappa statistics is shown in Fig. 8. For both the first and second simulation study designs, the pathway-level model was more stable than the gene-level models. We assessed the relative computational cost of the gene-level and pathway-level models by measuring total execution time for the first simulation study design without noise. In this case, estimation of the pathway-level model required on average only 2.3% of the time needed to estimate the gene-level model, a dramatic difference in total computational cost.

### Results on real TCGA data

We also compared the predictive performance of the pathway-level and gene-level models using the TCGA survival data.

**Fig. 9:**
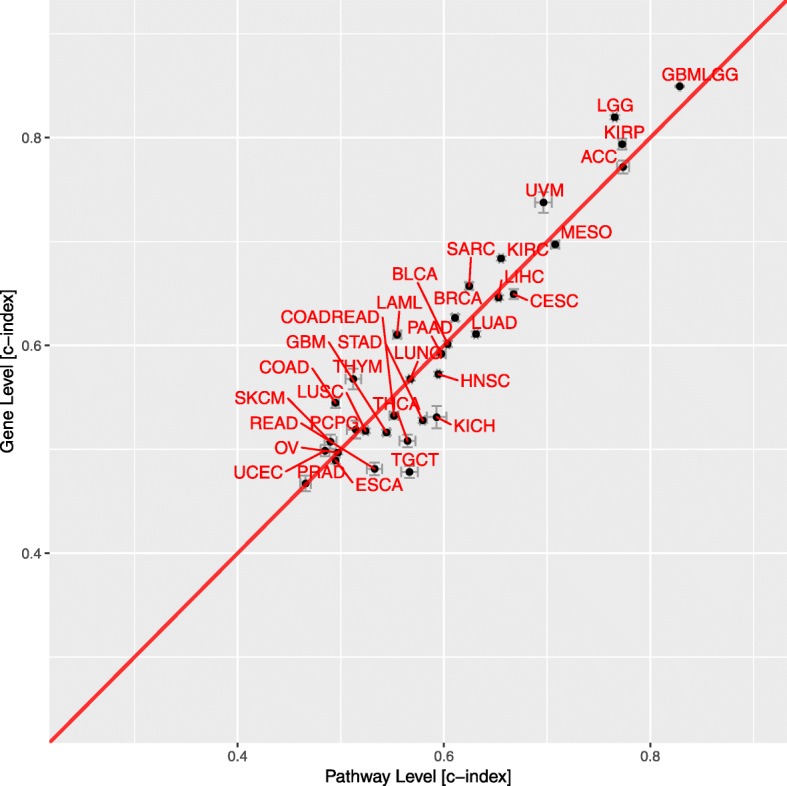
Predictive performance of gene-level and pathway-level models for 33 TCGA cohorts. Each point represents the average Cox concordance index of 50 replications for the pathway-level and gene-level models for a given TCGA cohort. Error bars represent the standard error of the estimates across50 replications

Figure 9 displays the predictive performance of gene-level and pathway-level models on each of the TCGA cohorts using real expression and survival values. Similar to the simulation studies, predictive performance on the real survival data was quantified by the concordance index (CI), which was averaged over 50 replications of 5-fold nested cross validation. In Fig. 9, the mean CI and CI standard error for both gene-level and pathway-level models are displayed for each of the 33 evaluated TCGA cohorts. Table 1 includes equivalent results. As seen in Fig. 9, the pathway-level and gene-level models had similar predictive performance on the TCGA data when taking into account the CI standard error. Mean CI values for each cohort ranged from around ~ 0.5 to ~ 0.8.

**Fig. 10:**
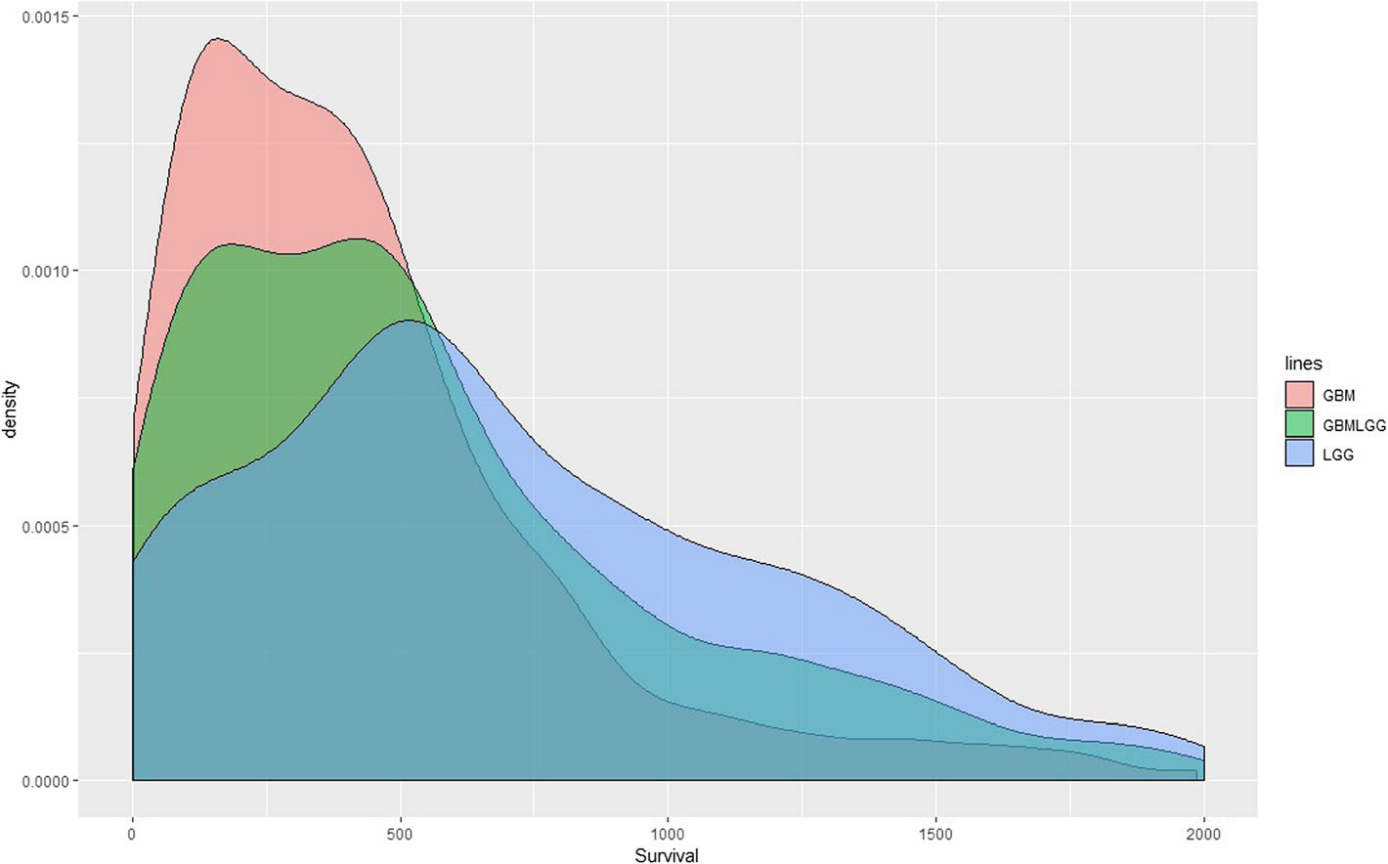
The distribution of survival times for LGG, GBM, GBMLGG cohorts

It’s worth noting that only some of the cancer types have a high concordance index. We believe that the observed differences in the predictive power for different cancer types may be due to factors that include survival data quality (e.g., number of death events and likelihood that observed deaths are in fact due to the cancer), patient clinical characteristics (e.g., smokers vs. non-smokers), cancer clinical characteristics (e.g., benign vs. malignant) and underlying cancer biology (e.g., cancers driven by CNVs vs. point mutations). As an interesting example, one can look at the relative predictive performance values for three related cancer types: low grade glioma (LGG), glioblastoma (GBM), low grade glioma and glioblastoma (GBMLGG). The GBMLGG cohort works quite well, the c-index is as high as 0.85 for gene-level model and 0.83 for pathway-level model. The LGG cohort alone drops a little to 0.82 for gene-level model and 0.77 for pathway-level model while GBM cohort alone is almost 0.5 (0.52 for gene-level model and 0.54 for pathway-level model). We believe that this pattern may be partly explained by the variance of survival times of these three cancers. As shown in Fig. 10, GBM has the worst prognosis and little variance in survival times while LGG has a comparatively better prognosis, longer expected survival time and higher survival time variance. The increased variance in survival times for LGG may allow for the improved predictive power for this cohort relative to GBM. Once the LGG and GBM are combined into the single cohort GBMLGG, the predictive performance is further increased due to both the larger sample size and the fact that the classification of each expression profile as either LGG or GBM provides a powerful predictive signal.

## Discussion

### Impact of inter-gene correlation on predictive performance

Our analysis of cancer prognostic models using simulated and real cancer gene expression data demonstrates that inter-gene correlation has a significant impact on model predictive performance. Our second simulation, which removed inter-gene correlation, and does not represent a biologically realistic scenario. Therefore, this simulation study cannot be used to support an assertion that a pathway-based model would generate superior predictive performance to a gene-level model on real expression data. But we included this simulation scenario to highlight the impact of inter-gene correlation on the relative performances of gene-level and pathway-level models; it should not be viewed as a scenario that researchers might encounter in practice. For both gene-level and pathway-level models, predictive performance was positively correlated with the level of inter-gene correlation, i.e., higher levels of correlation in the gene expression data were associated with improved performance. The inter-gene correlation worked as an amplifier of the association signal since all genes in a correlated group became associated with survival even when just a single gene was selected as an associated predictor in the simulation model. This larger pool of prognostic genes improves the ability of the penalized estimation procedure to identify a predictive model. The predictive performance of the gene-level models is attributed largely to this amplification effect. The predictive performance of the gene-level model dropped to null when there was no inter-gene correlation in the second simulation study.

Besides, our simulation approach of generating the survival time using the average of a random group is substantially more challenging than the case where the survival time is simulated using the correlated genes within a pathway. It is therefore expected that predictive performance for the random genes models is significantly lower than for the non-null model. The link between inter-gene correlation and predictive performance is also expected for similar reasons.

### Impact of pathway size on predictive performance

To investigate if the choice of pathway used to simulate survival times impacts model performance, we tested the models using survival times generated using each of the 50 pathways in the MSigDB Hallmark collection, presented in Additional file 2. The pathway sizes ranged from 32 to 200. Comparison of the results from the large and small pathways demonstrates that the performance of the pathway-level models is not sensitive to pathway size. Although the performance of gene-level models was not sensitive to pathway size in the first simulation study that included moderate inter-gene correlation, it became sensitive in the second simulation study that lacked inter-gene correlation. For this case, the gene-level model only had non-null predictive performance when the pathway size was small and no noise was added to the simulated survival times, which implies that the gene-level models can only work when the number of associated variables is small and signal is strong, and it also demonstrates that the inter-gene correlation works as amplifier of the association signal.

### Impact of sample size and death rate on predictive performance

As expected, the predictive performance for both the real data analysis and simulation studies improved with larger sample sizes. For the second simulation study when sample size was lower than 400, the gene-level prognostic model had close to null predictive performance, even when the number of associated variables was small and no noise was added to the simulated survival times. Although there was no strong correlation between sample size and predictive performance, or between death rate and predictive performance, there was a strong correlation between death rate and the standard error (for pathway-level model, the Spearman correlation was − 0.57 and for gene-level model, the correlation was − 0.61) since lower death rates imply a higher censoring rate and thus greater uncertainty. The incomplete annotation of patient clinical data and relatively short-term follow-up interval for most TCGA cohorts has been noted by several studies [30, 31] and may increase the standard error in our analysis. Although some studies have attempted to avoid this data quality issue by using a curated and filtered clinical and survival data [32], this is still an impactful issue.

### Limitations

One limitation of the pathway-level models is that not all genes are mapped to pathways and, because our analysis only included genes that exist in at least one pathway, the models may fail to consider some genes that have a true association with patient survival. Moreover, the misspecification of pathways in existing databases also affects the performance of pathway-level model. Unknown driver genes may be missed and irrelevant genes may be included. One possible solution to this limitation involves the use of de novo enrichment methods, such as [33] which could extract de novo pathways from molecular interaction networks in the context of pathway-based prediction. Although the pathway-based prediction is used for cancer subtypes classification, it is a great inspiration for other pathway-based prediction models, such as cancer prognosis prediction. Another issue is that when Lasso deals with correlated variables, it tends to retain just one random variable from each correlated group of variables [34]. Therefore, when a specific pathway or gene is selected by a model, it may not be the only pathway or gene that could have been considered. For prognostic evaluation, the specific choice of pathway may not matter, but if pathway analysis is used for selecting therapies for patients, then identifying the full range of pathways that may be helpful for managing a patient is important. In that case, other predictive modeling techniques, such as random forests or other variants of Lasso [35, 36], may help.

## Conclusions

In this study, we used penalized Cox proportional hazards models with either gene-level or pathway-level predictors for cancer prognosis prediction from tumor gene expression data. We evaluated and compared the gene-level and pathway-level models using tumor gene-expression data from the TCGA and either real or simulated survival times. We found that models using pathway-level predictors were more interpretable, stable and computationally efficient as compared to models using gene-level predictors. For realistic gene expression correlation structures, the pathway-level and gene-level models had similar predictive performance. In cases where the level of correlation between the expression values of prognostic genes is low, the pathway-level model had superior predictive power relative to the gene-level model. These findings provide guidance for researchers who are interested in building prognostic models from tumor gene expression data. If researchers expect a high level of inter-gene correlation in the expression data, both the gene-level method and pathway-level models can provide good prognosis prediction with the pathway-level model having the benefits of parsimony, stability and efficiency and the gene-level having the advantage of identifying specific genes for downstream experiments. If the level of inter-gene correlation is low, a pathway-level model may also outperform a gene-level model in predictive power. The gene-level model may work when the number of variables is small and the signal is strong but will be worse than the pathway-level model without inter-gene correlation structure in the data.

## Supplementary information

**Additional file 1.** Supplementary results of the simulation studies for all TCGA cohorts.

**Additional file 2.** Supplementary results of associating different pathways in the simulation studies for LGG cohort.

## Data Availability

The datasets analyzed during the current study are available in the UCSC TCGA repository, http://xena.ucsc.edu/, and MSigDB database, http://software.broadinstitute.org/gsea/msigdb/genesets.jsp?collection=H
