# Supplementary material for: Comparison of pathway and gene-level models for cancer prognosis prediction
Source: BMC Bioinformatics. 2020 Feb 28;21:76. doi: 10.1186/s12859-020-3423-z (PMC7048092; doi:10.1186/s12859-020-3423-z)
Supplement: Supplementary file 1 — Additional file 1. Supplementary results of the simulation studies for all TCGA cohorts. [file 12859_2020_3423_MOESM1_ESM.pdf]

Supplementary Material: Comparison of pathway and gene-level models for cancer prognosis prediction

Xingyu Zheng, Christopher I. Amos, H. Robert Frost

Supplementary results of the simulation studies for all TCGA cohorts:

Results of the 2 simulation studies based on gene expression data from 33 cohorts in TCGA and representative pathways from the MSigDB Hallmark collection. Figure S1 to S66 are displayed below and two figures corresponding to two simulation studies for each cohort are displayed sequentially. Since they share the same figure captions, we display the captions here.

Figures of simulation 1 have this figure caption:

Results of the simulation study based on gene expression data from the LGG cohort and representative pathways from the MSigDB Hallmark collection. Each panel plots the predictive performance of the evaluated gene-level and pathway-level models for simulation studies that associated survival with one of four Hallmark pathways (*Hallmark estrogen response late*, *Hallmark E2F targets*, *Hallmark TGF beta signaling* and *Hallmark MYC targets V2* respectively) selected to represent the four possible combinations of large or small pathway size and high or low average inter-gene correlation. In these plots, the Cox concordance index is plotted on the y-axis with the x-axis representing the standard deviation of the Gaussian noise added to the simulated survival times. The error bars represent the standard error over 20 replications.

Figures of simulation 2 have this figure caption:

Results of the simulation study based on gene expression data from the TCGA LGG cohort without inter-gene correlation and representative pathways from the MSigDB Hallmark collection. The correlation in the gene expression data has been broken by randomly permuting the values for each gene. Each panel plots the predictive performance of the evaluated gene-level and pathway-level models for simulation studies that associated survival with one of four Hallmark pathways (*Hallmark estrogen response late*, *Hallmark E2F targets*, *Hallmark TGF beta signaling* and *Hallmark MYC targets V2* respectively) selected to represent the four possible combinations of large or small pathway size and high or low average inter-gene correlation. In these plots, the Cox concordance index is plotted on the y-axis with the x-axis representing the standard deviation of the Gaussian noise added to the simulated survival times. The error bars represent the standard error over 20 replications.

Figure S1: ACC cohort, simulation 1

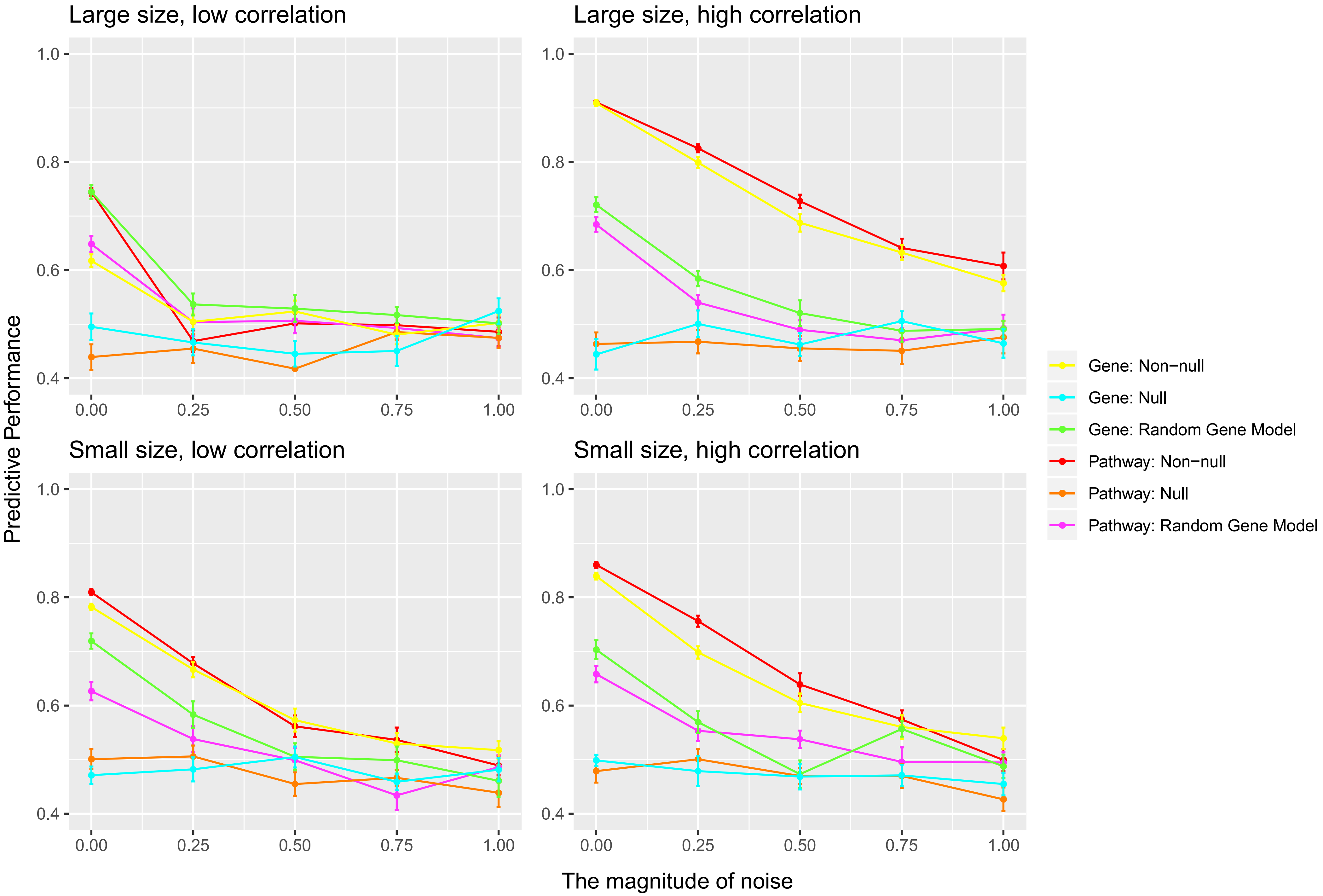

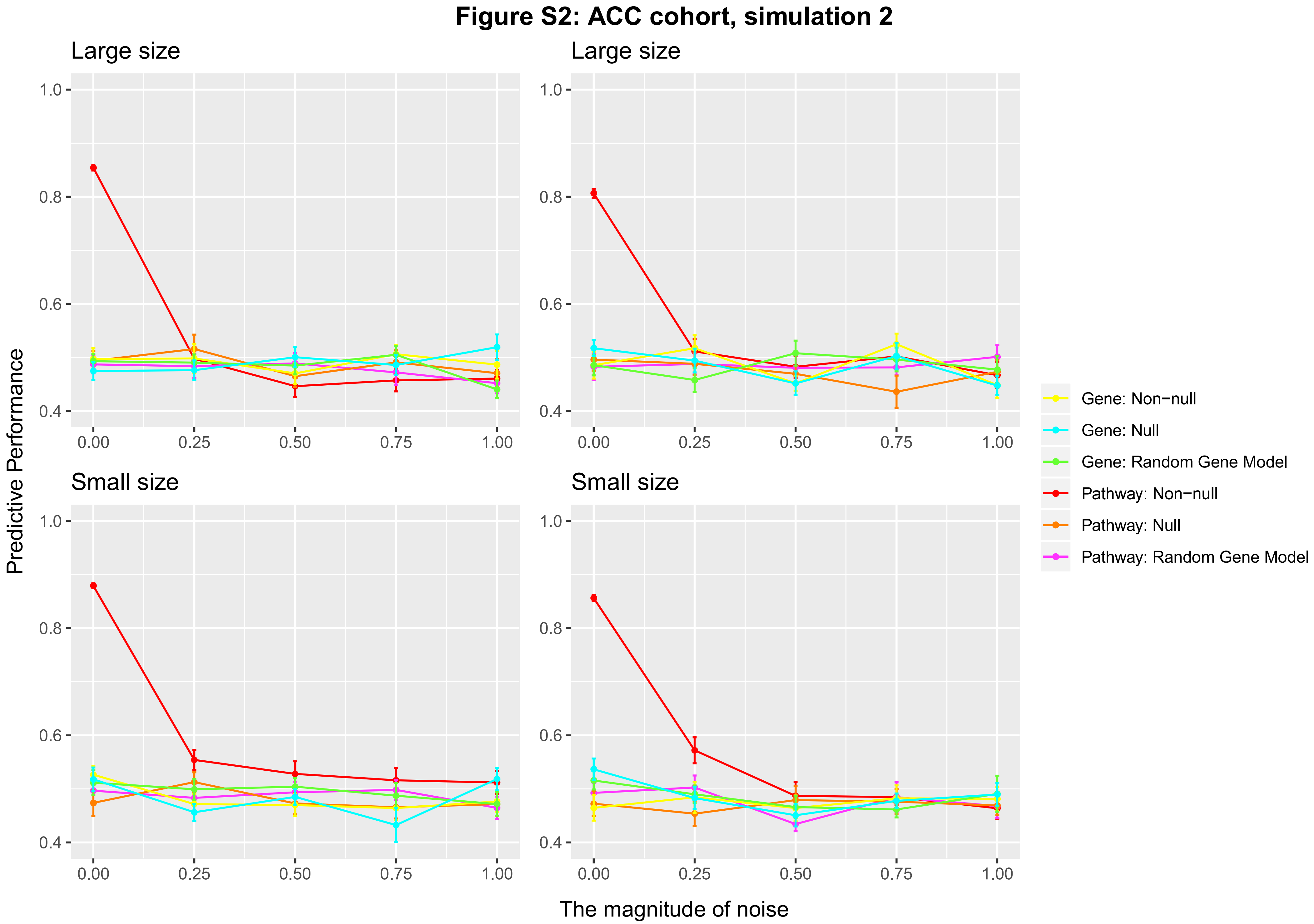
Figure S2: ACC cohort, simulation 2
Large size
Large size
Small size
Small size
Predictive Performance
The magnitude of noise
1.0
0.8
0.6
0.4
0.00
0.25
0.50
0.75
1.00
Gene: Non−null
Gene: Null
Gene: Random Gene Model
Pathway: Non−null
Pathway: Null
Pathway: Random Gene Model

Figure S3: BLCA cohort, simulation 1

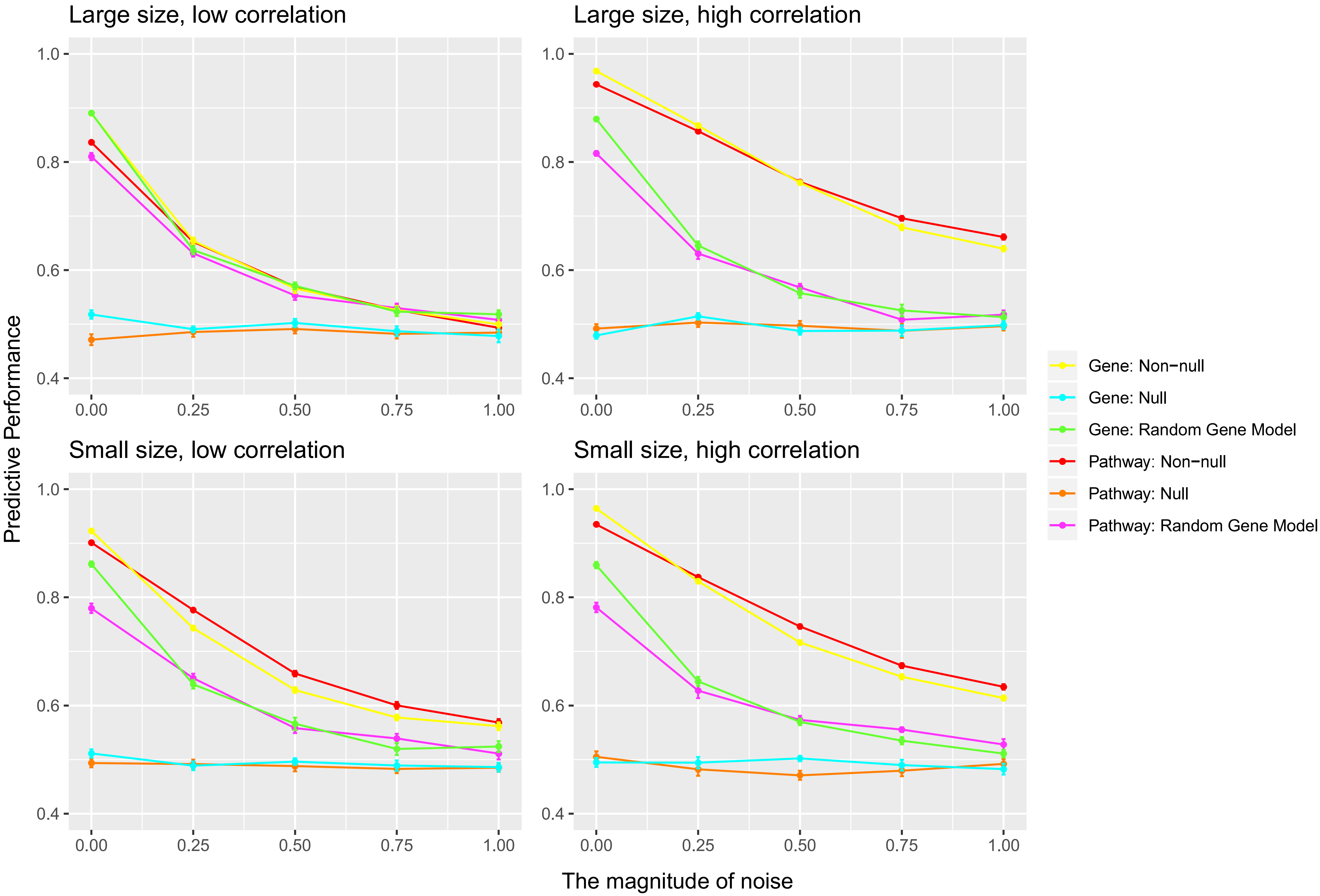

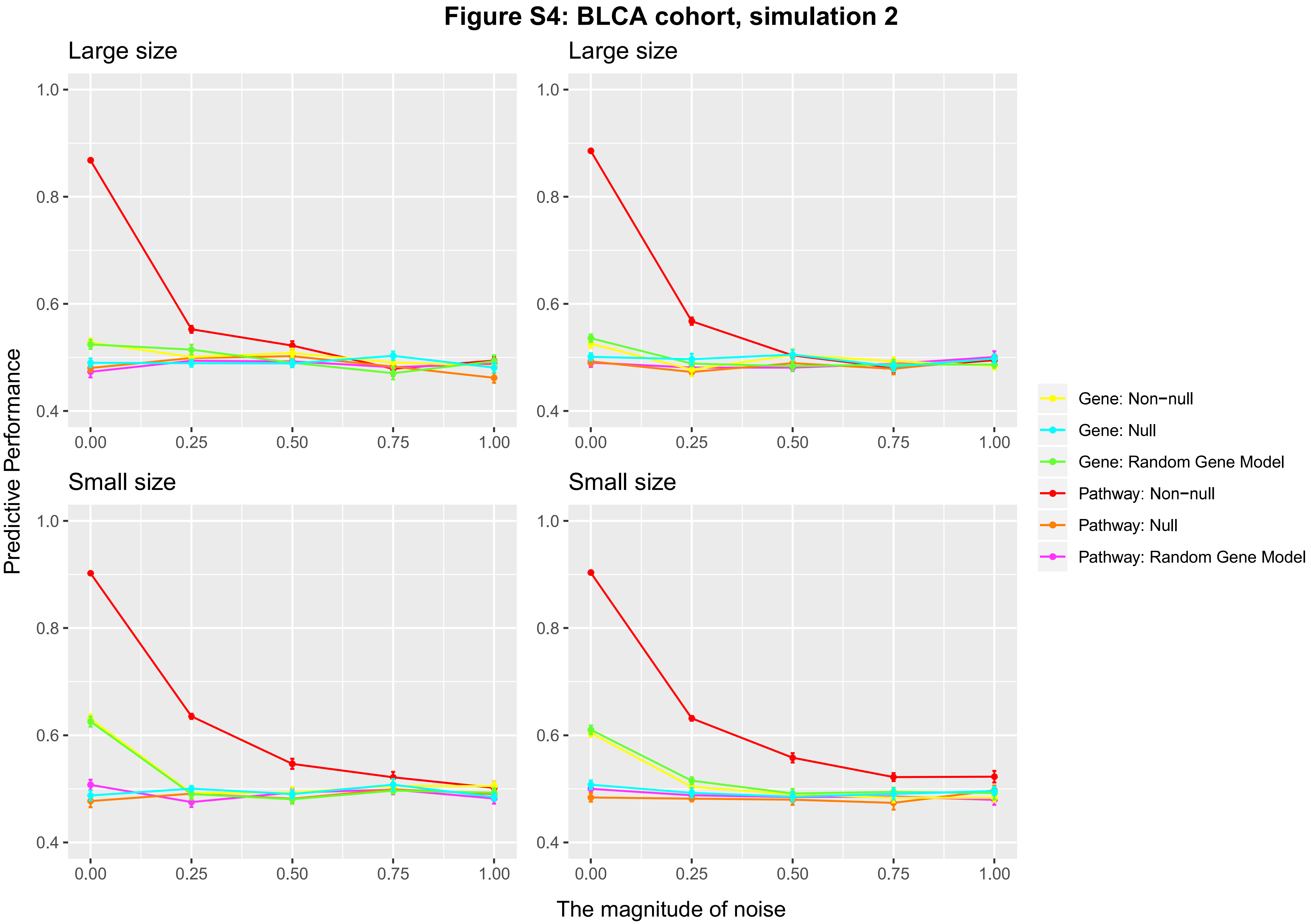

Figure S4: BLCA cohort, simulation 2
Large size
Large size
Small size
Small size
Predictive Performance
The magnitude of noise
0.4
0.6
0.8
1.0
0.00
0.25
0.50
0.75
1.00
Gene: Non−null
Gene: Null
Gene: Random Gene Model
Pathway: Non−null
Pathway: Null
Pathway: Random Gene Model

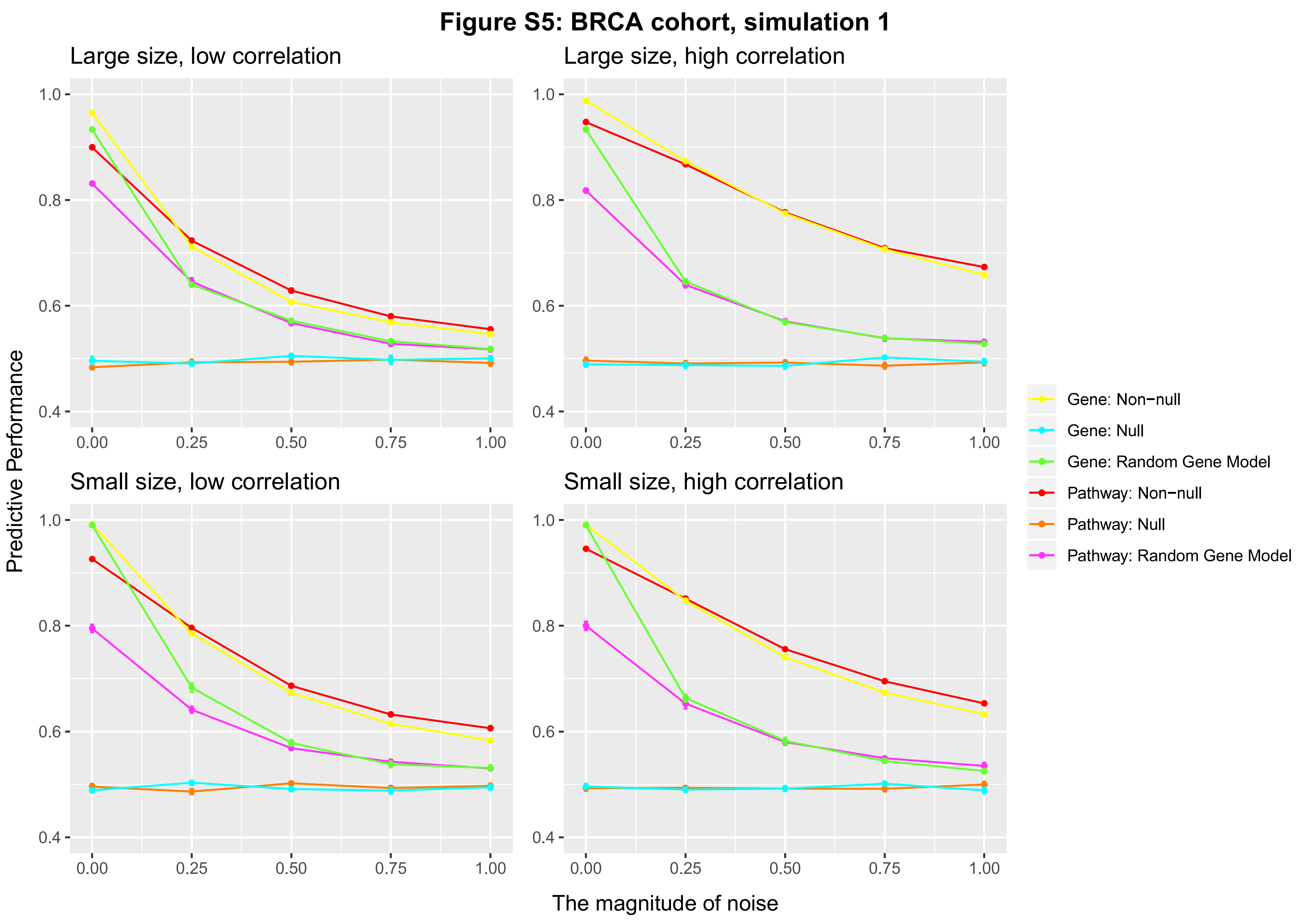

Figure S5: BRCA cohort, simulation 1
Large size, low correlation
Large size, high correlation
Small size, low correlation
Small size, high correlation
Predictive Performance
The magnitude of noise
0.4
0.6
0.8
1.0
0.00
0.25
0.50
0.75
1.00
Gene: Non−null
Gene: Null
Gene: Random Gene Model
Pathway: Non−null
Pathway: Null
Pathway: Random Gene Model

Figure S6: BRCA cohort, simulation 2

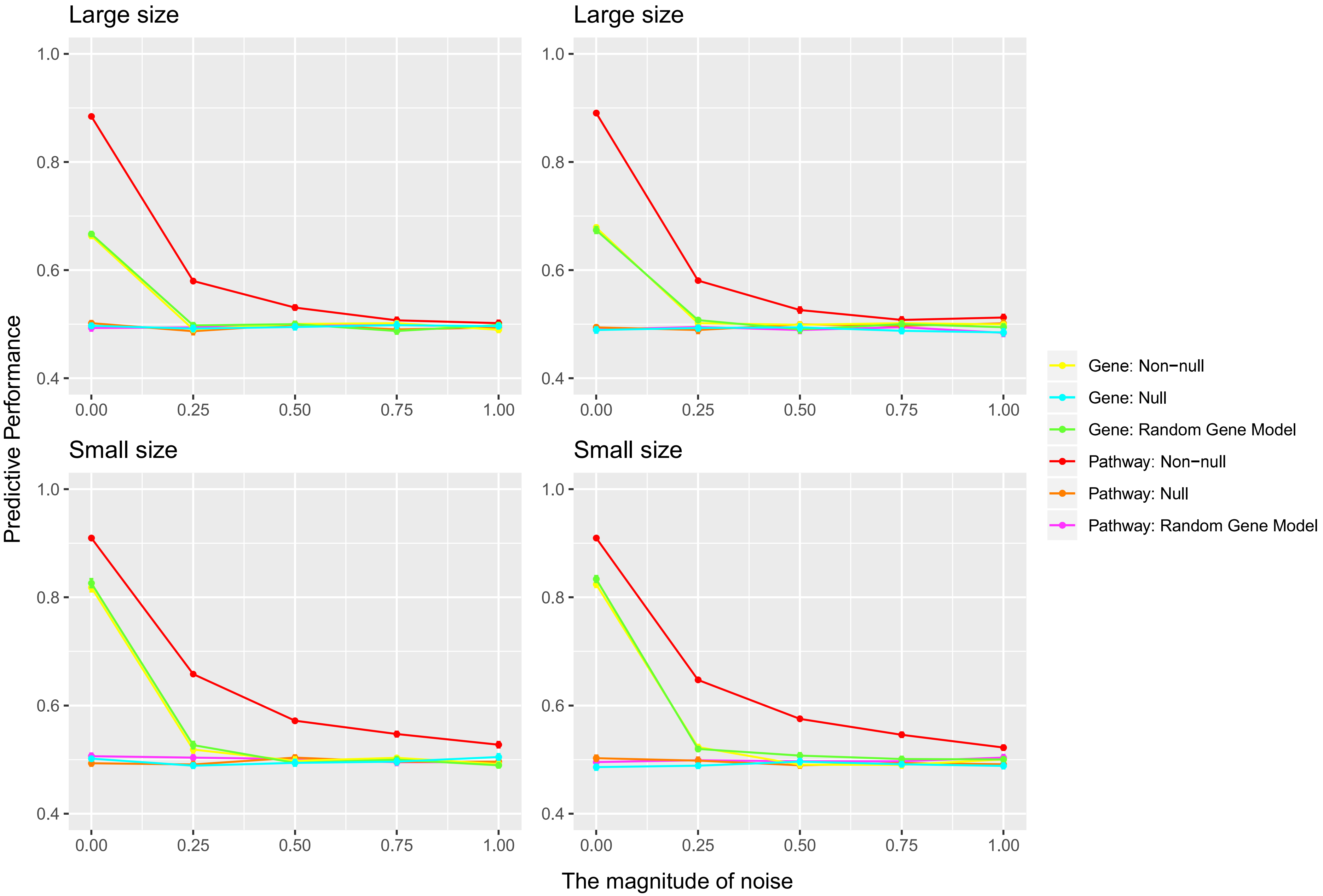

Figure S7: CESC cohort, simulation 1

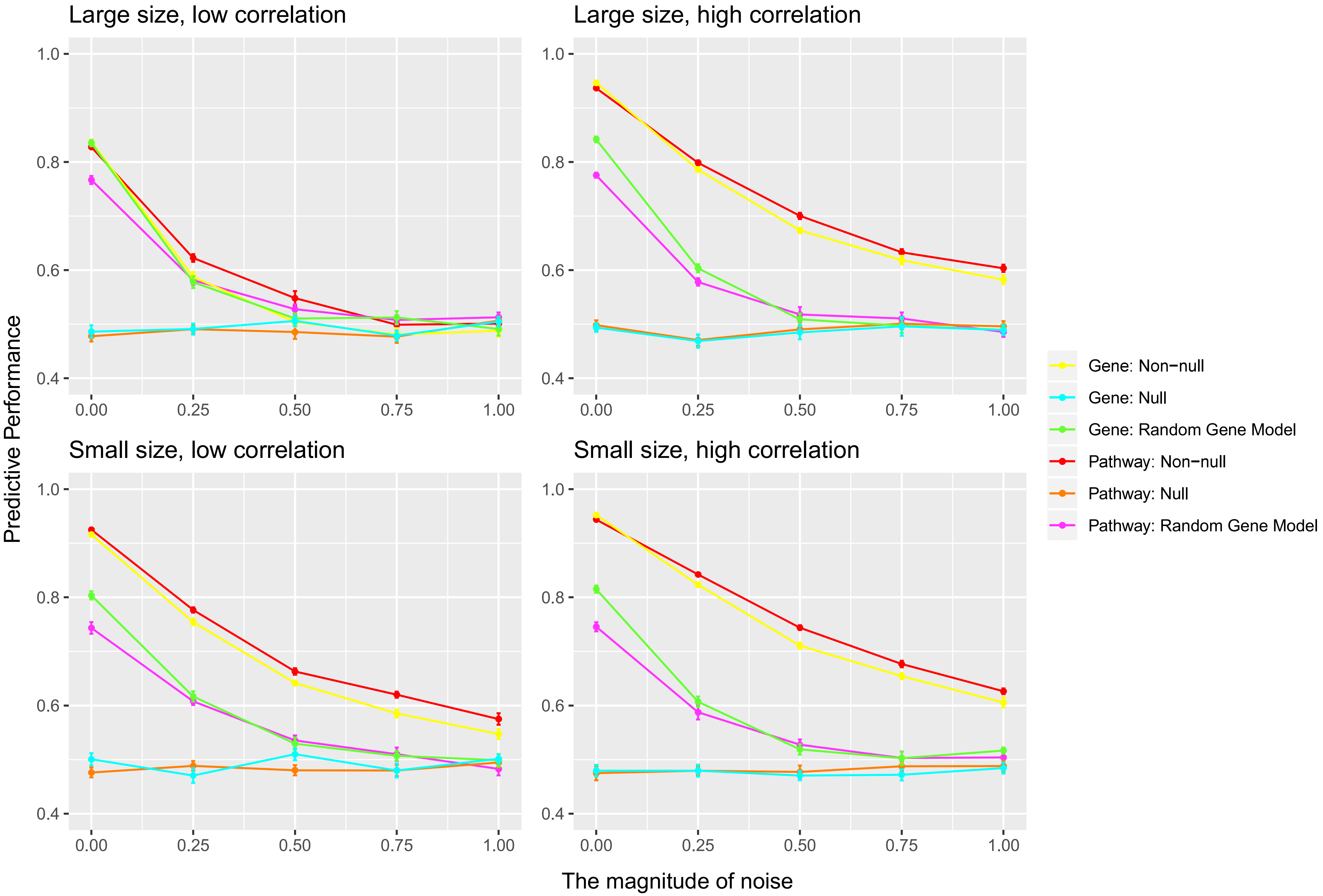

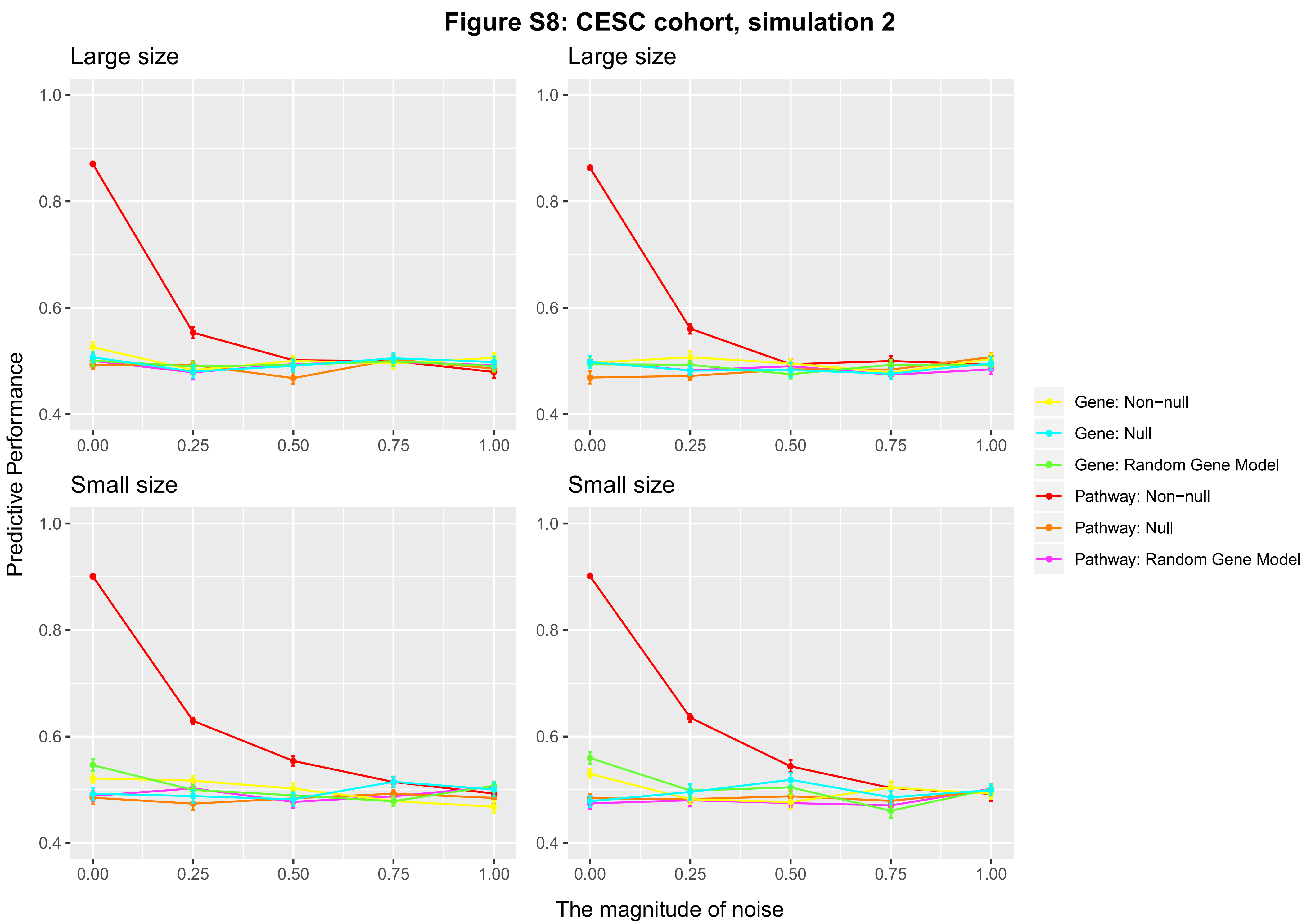
Figure S8: CESC cohort, simulation 2
Large size
Large size
Small size
Small size
Predictive Performance
The magnitude of noise
1.0
0.8
0.6
0.4
0.00
0.25
0.50
0.75
1.00
Gene: Non−null
Gene: Null
Gene: Random Gene Model
Pathway: Non−null
Pathway: Null
Pathway: Random Gene Model

Figure S9: COAD cohort, simulation 1

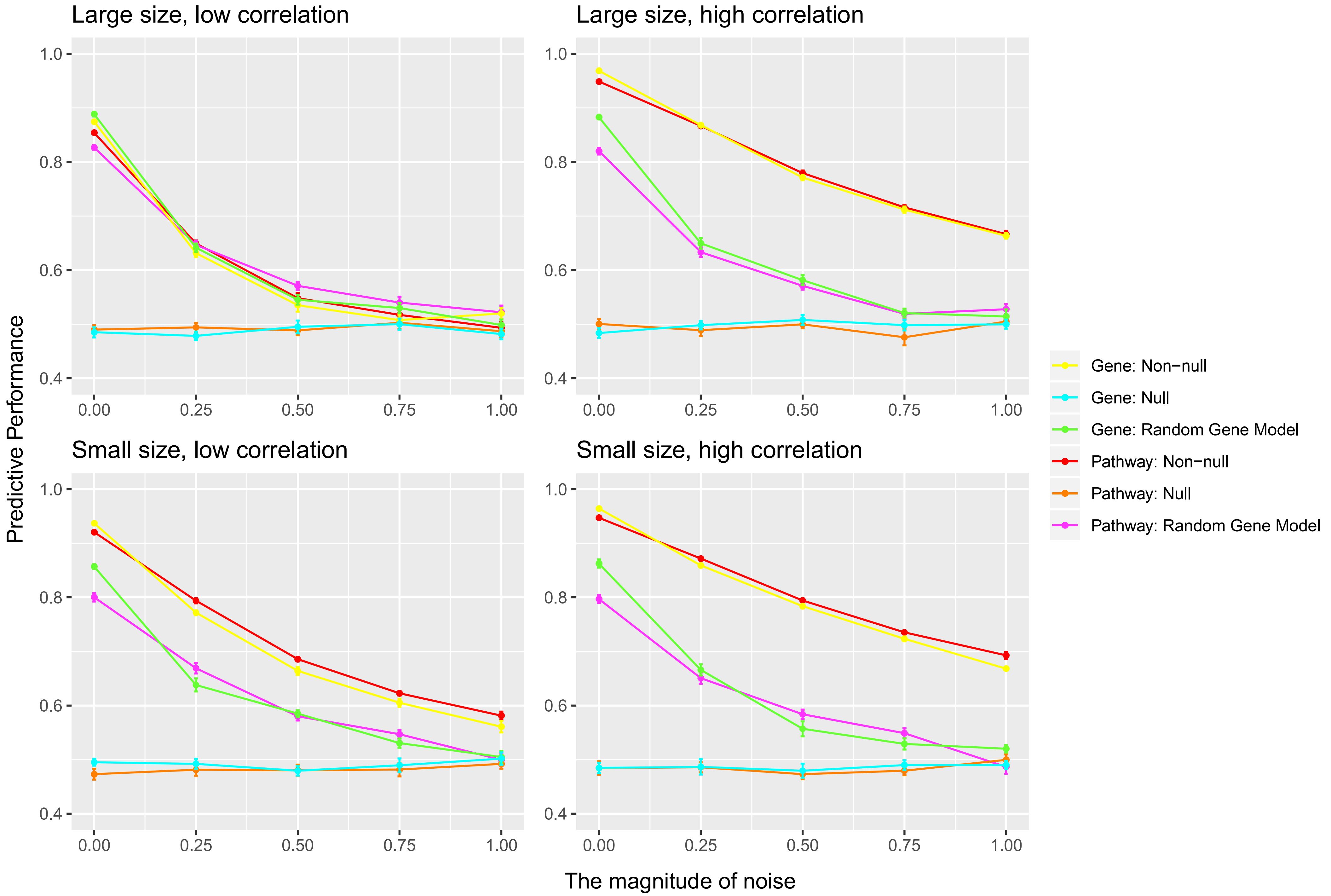

Figure S10: COAD cohort, simulation 2

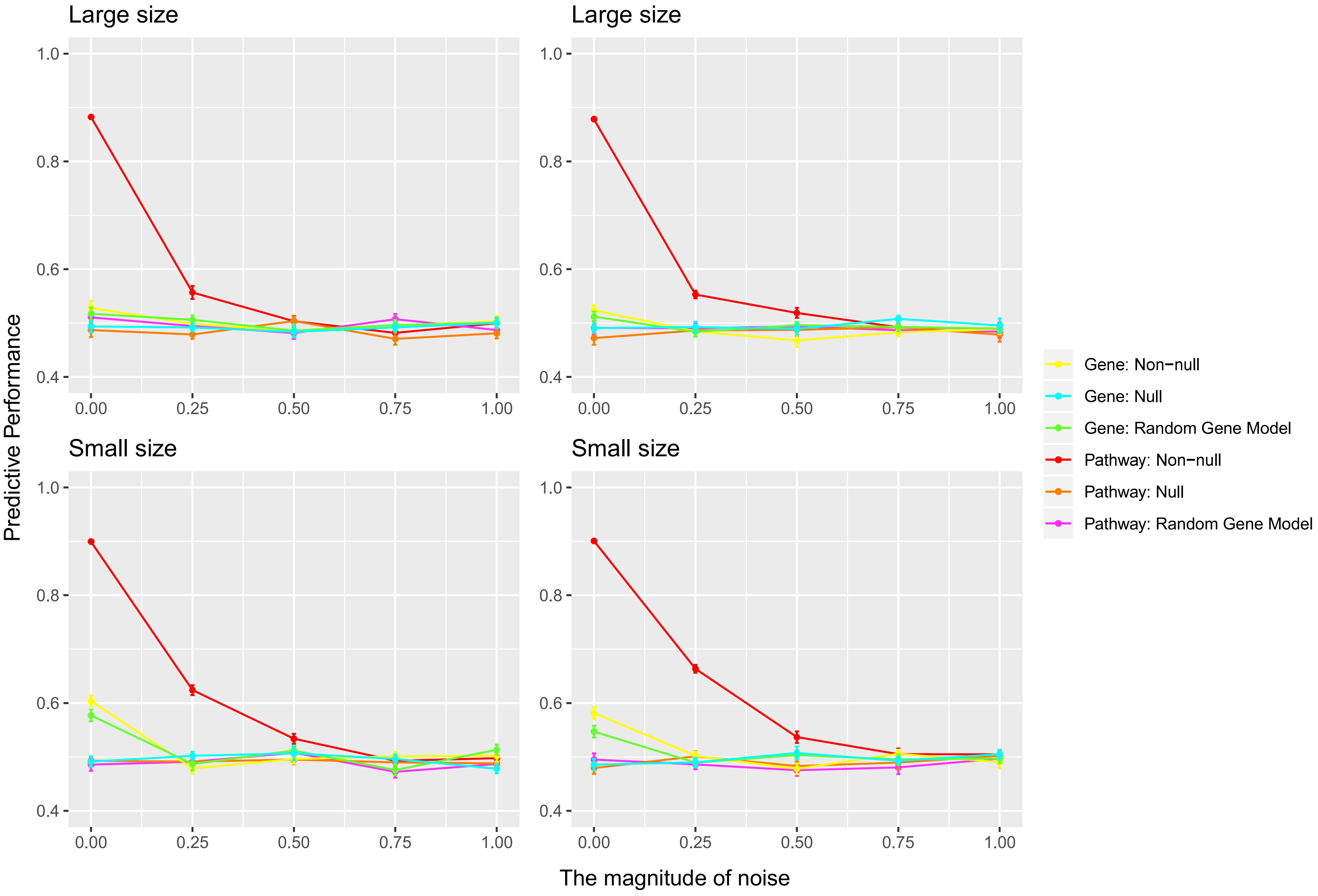

Figure S11: COADREAD cohort, simulation 1

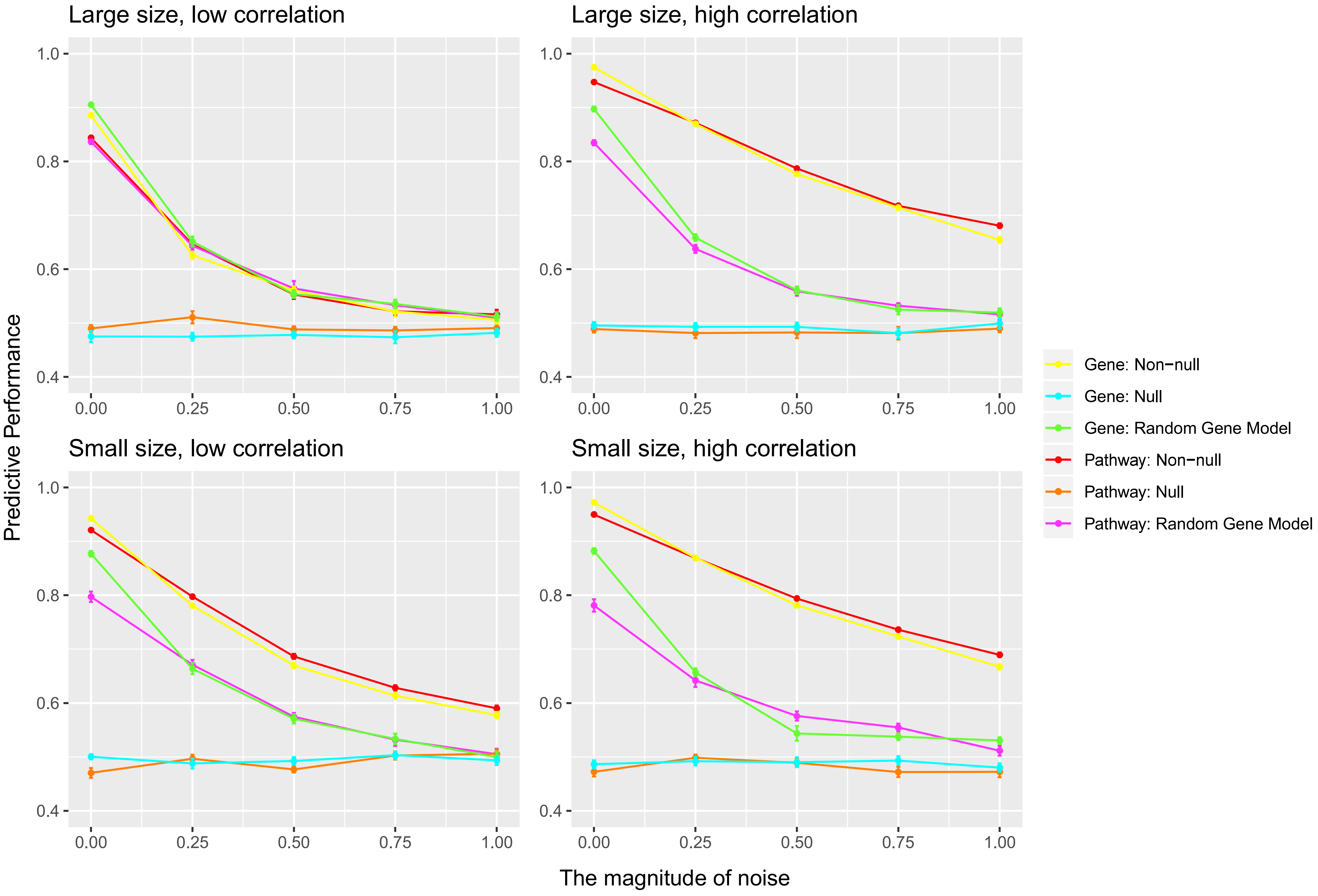

Figure S12: COADREAD cohort, simulation 2

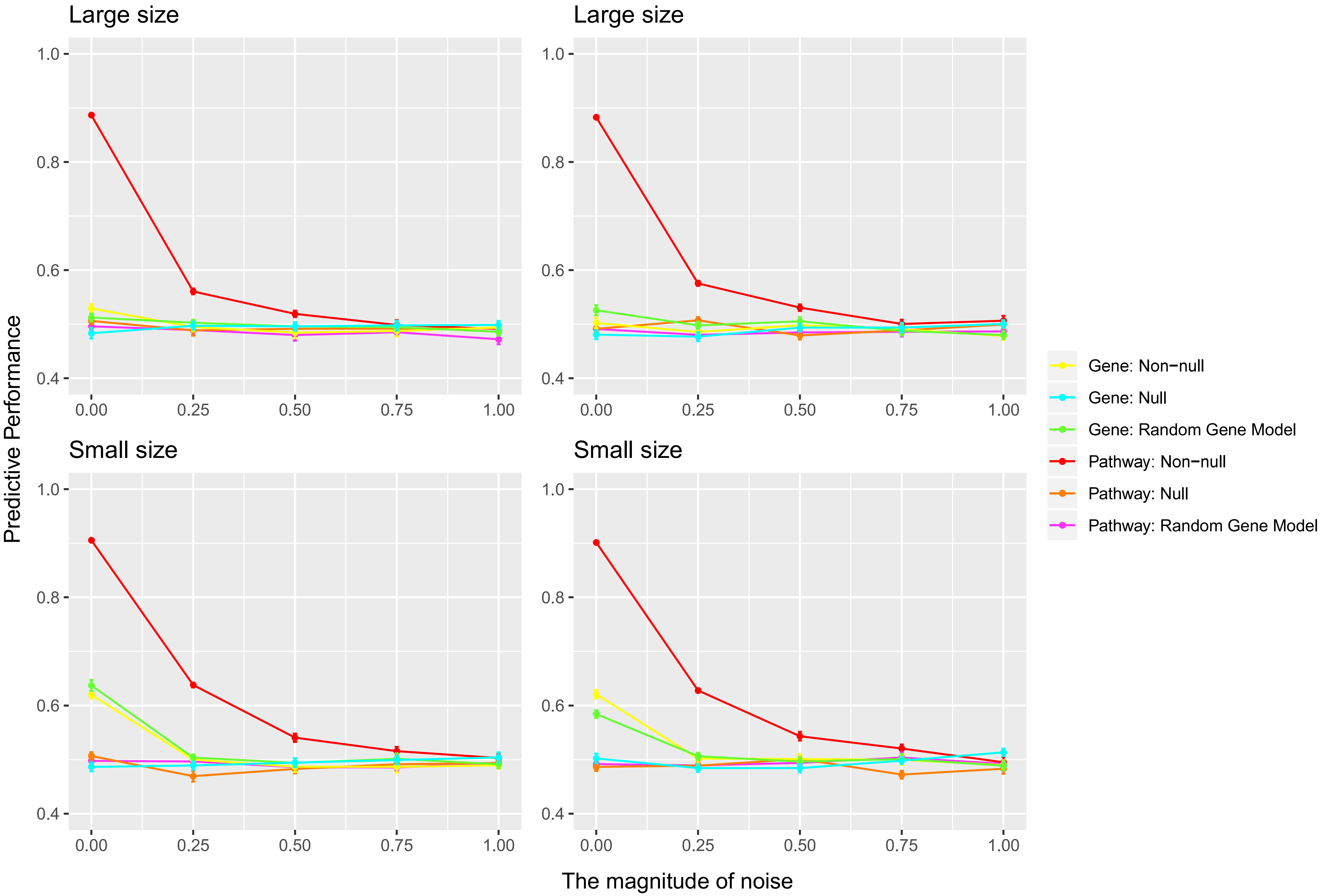

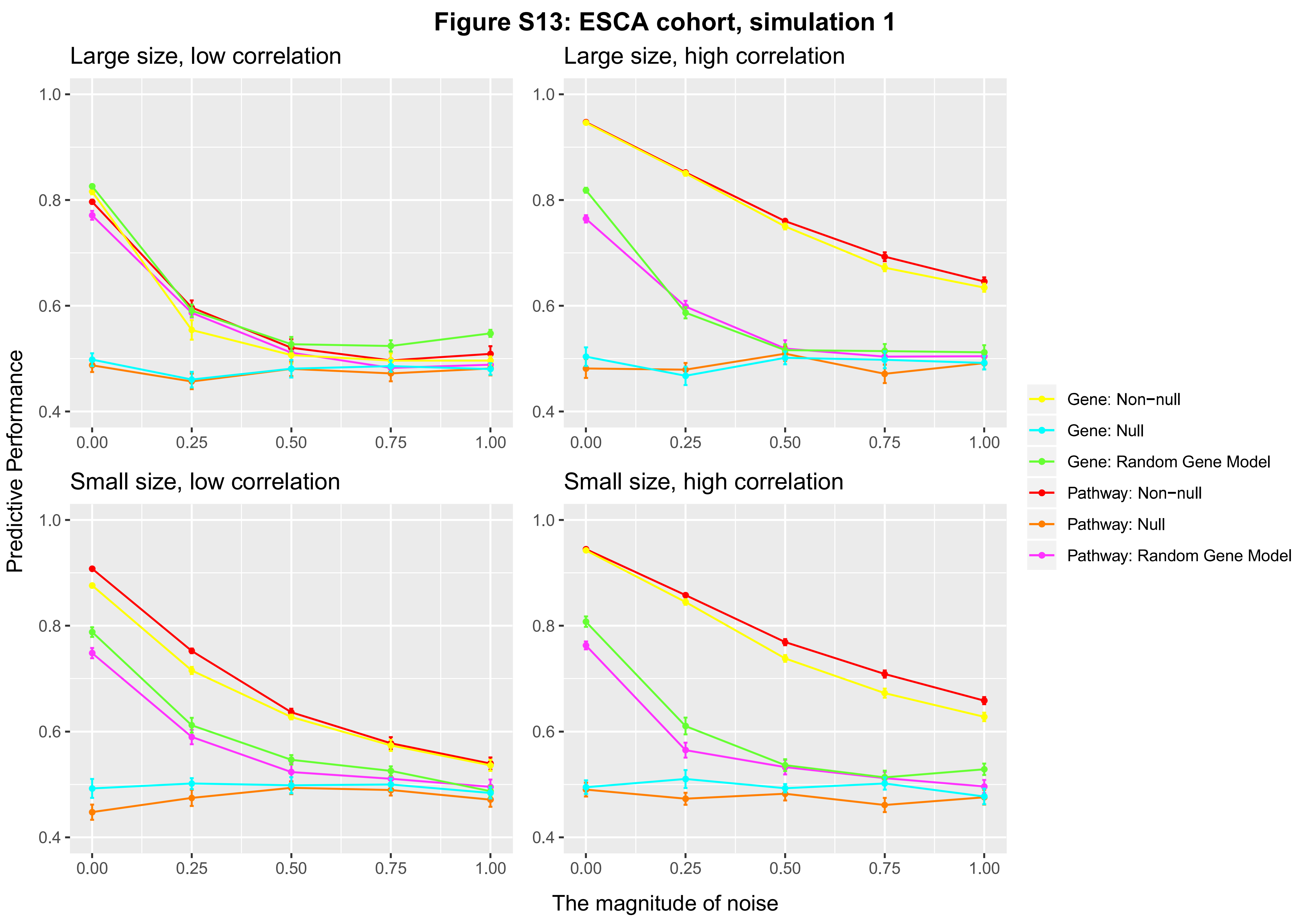
Figure S13: ESCA cohort, simulation 1
Large size, low correlation
Large size, high correlation
Small size, low correlation
Small size, high correlation
Predictive Performance
The magnitude of noise
1.0
0.8
0.6
0.4
0.00
0.25
0.50
0.75
1.00
Gene: Non−null
Gene: Null
Gene: Random Gene Model
Pathway: Non−null
Pathway: Null
Pathway: Random Gene Model

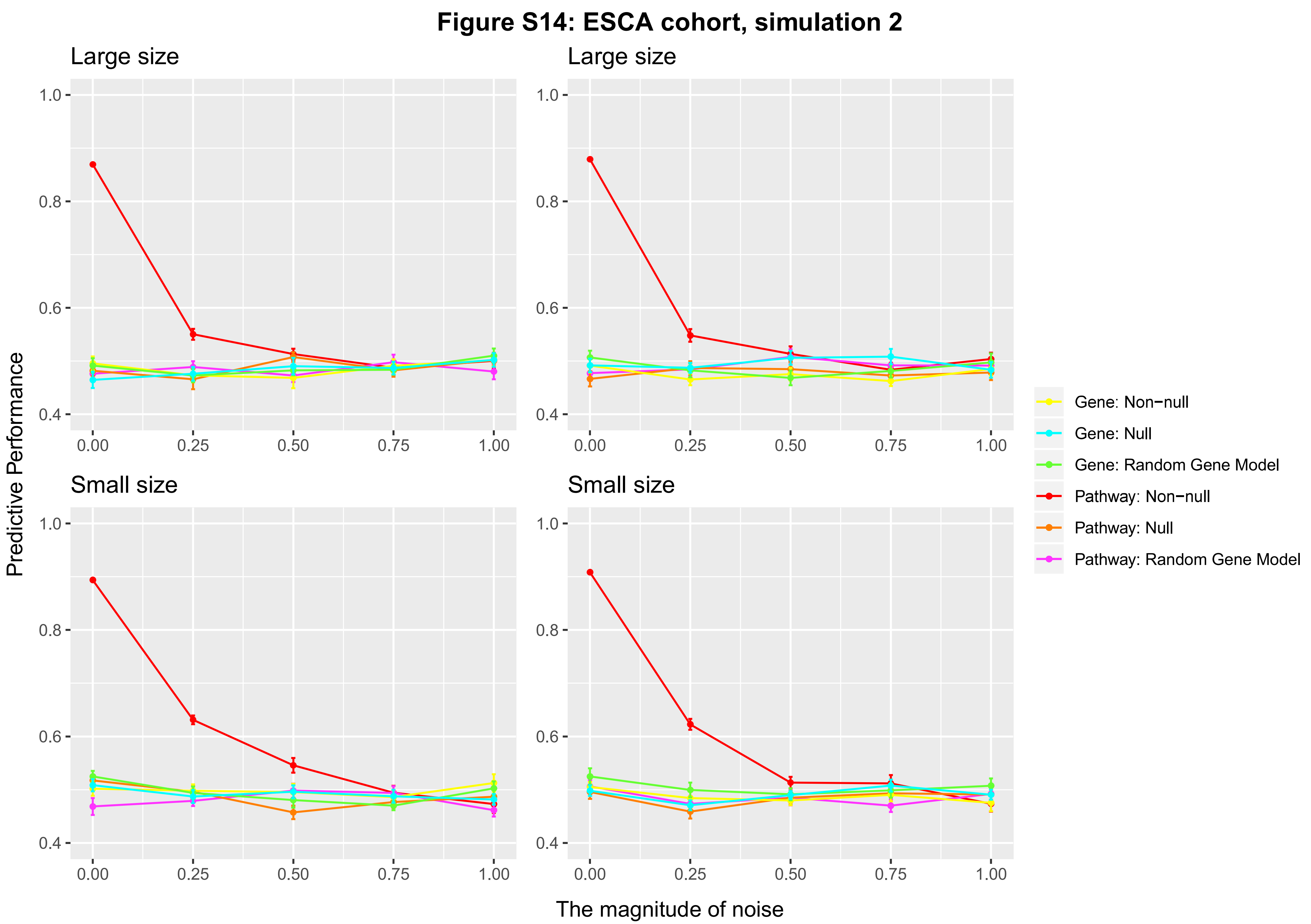

Figure S14: ESCA cohort, simulation 2
Large size
Large size
Small size
Small size
Predictive Performance
The magnitude of noise
1.0
0.8
0.6
0.4
0.00
0.25
0.50
0.75
1.00
Gene: Non−null
Gene: Null
Gene: Random Gene Model
Pathway: Non−null
Pathway: Null
Pathway: Random Gene Model

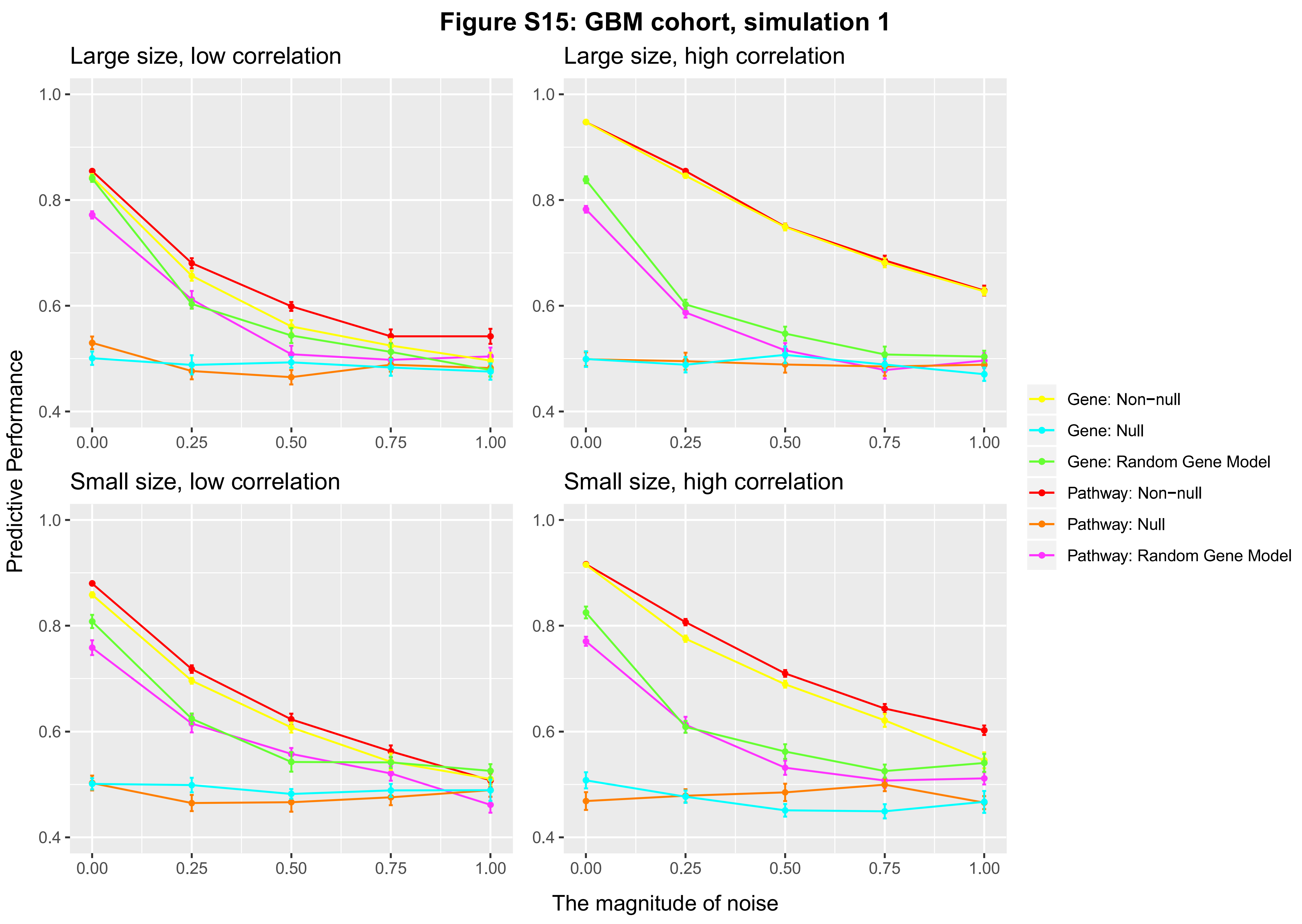

Figure S15: GBM cohort, simulation 1
Large size, low correlation
Large size, high correlation
Small size, low correlation
Small size, high correlation
1.0
0.8
0.6
0.4
0.00
0.25
0.50
0.75
1.00
Predictive Performance
The magnitude of noise
Gene: Non−null
Gene: Null
Gene: Random Gene Model
Pathway: Non−null
Pathway: Null
Pathway: Random Gene Model

Figure S16: GBM cohort, simulation 2

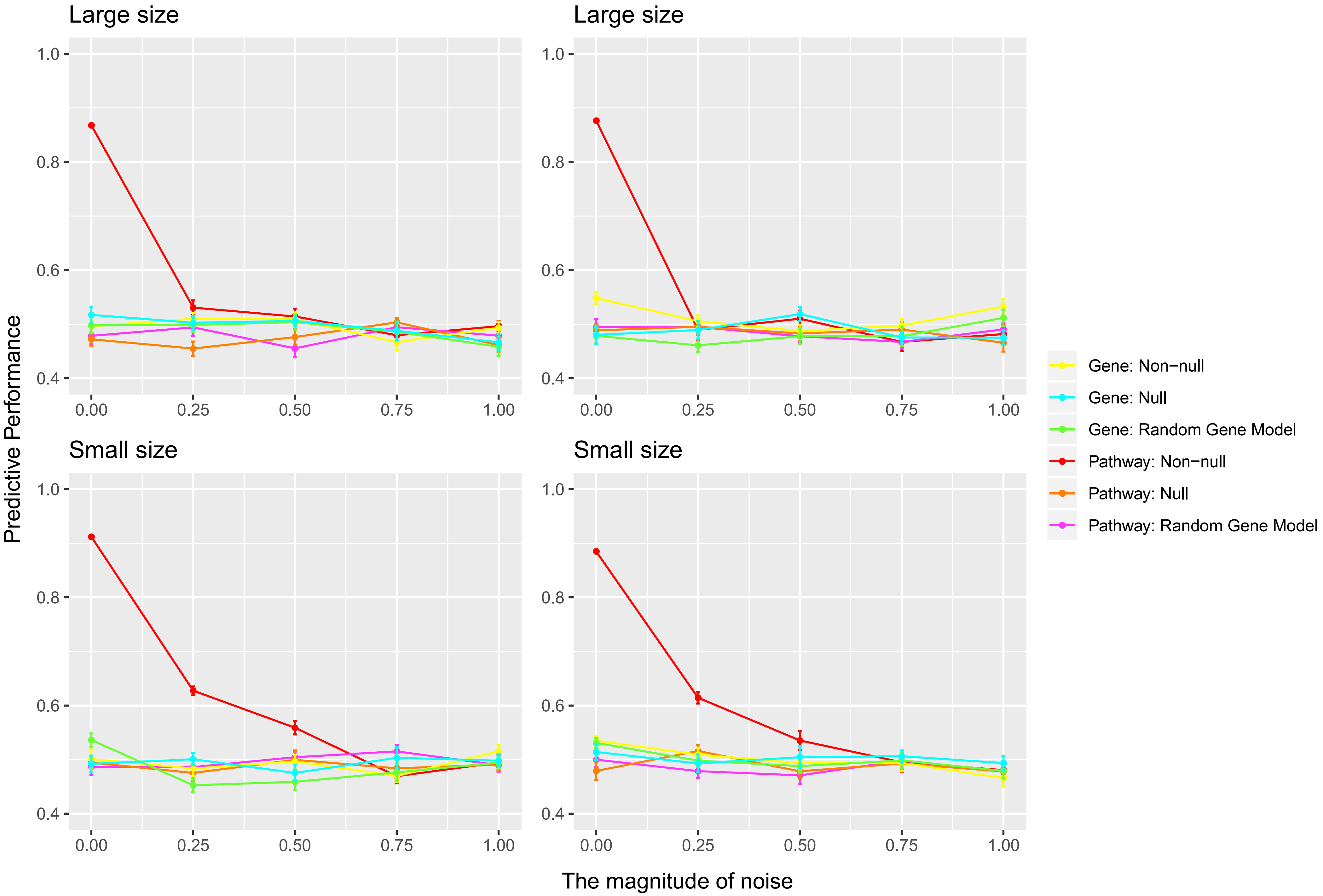

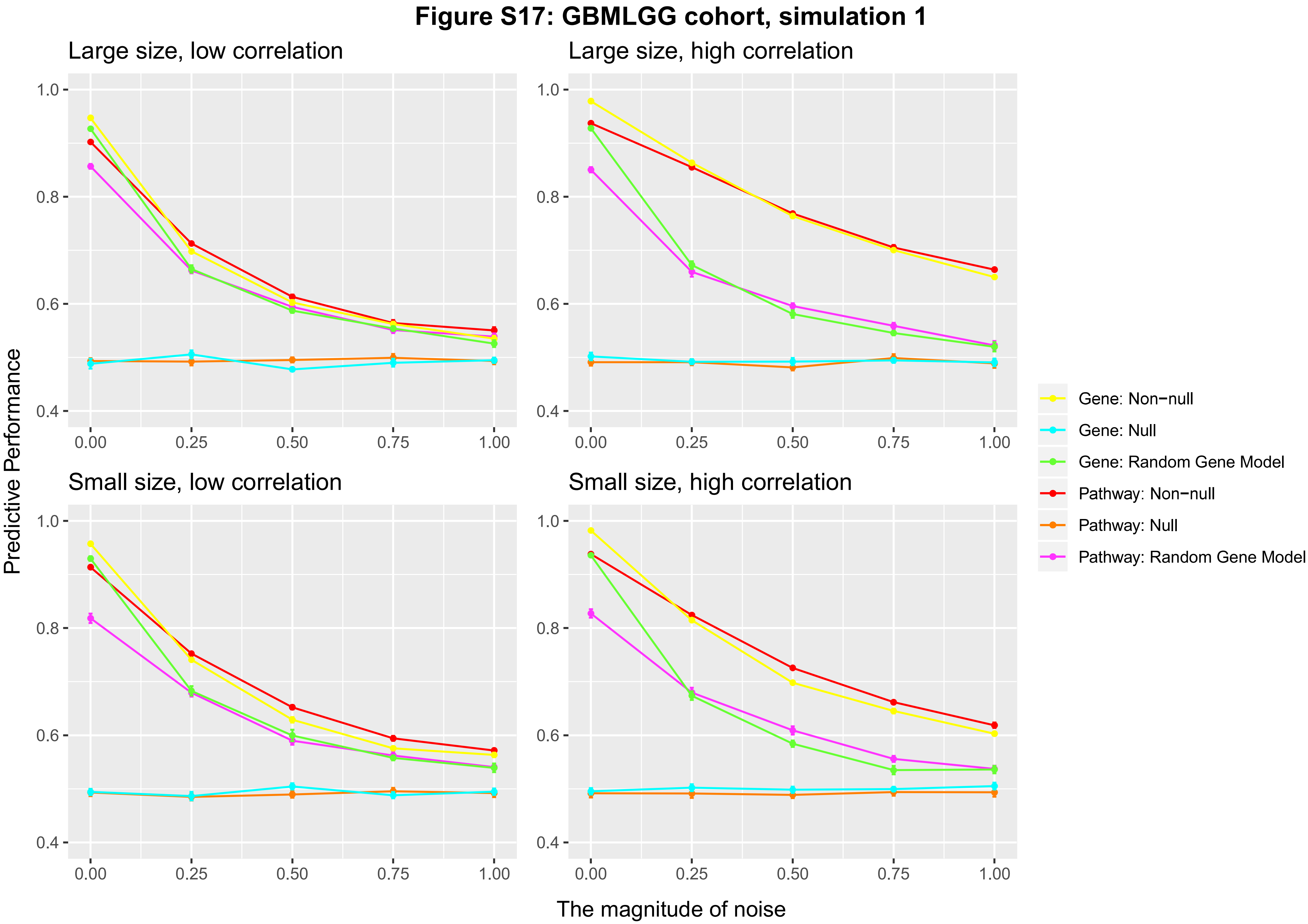

Figure S17: GBMLGG cohort, simulation 1
Large size, low correlation
Large size, high correlation
Small size, low correlation
Small size, high correlation
Predictive Performance
The magnitude of noise
1.0
0.8
0.6
0.4
0.00
0.25
0.50
0.75
1.00
Gene: Non−null
Gene: Null
Gene: Random Gene Model
Pathway: Non−null
Pathway: Null
Pathway: Random Gene Model

Figure S18: GBMLGG cohort, simulation 2

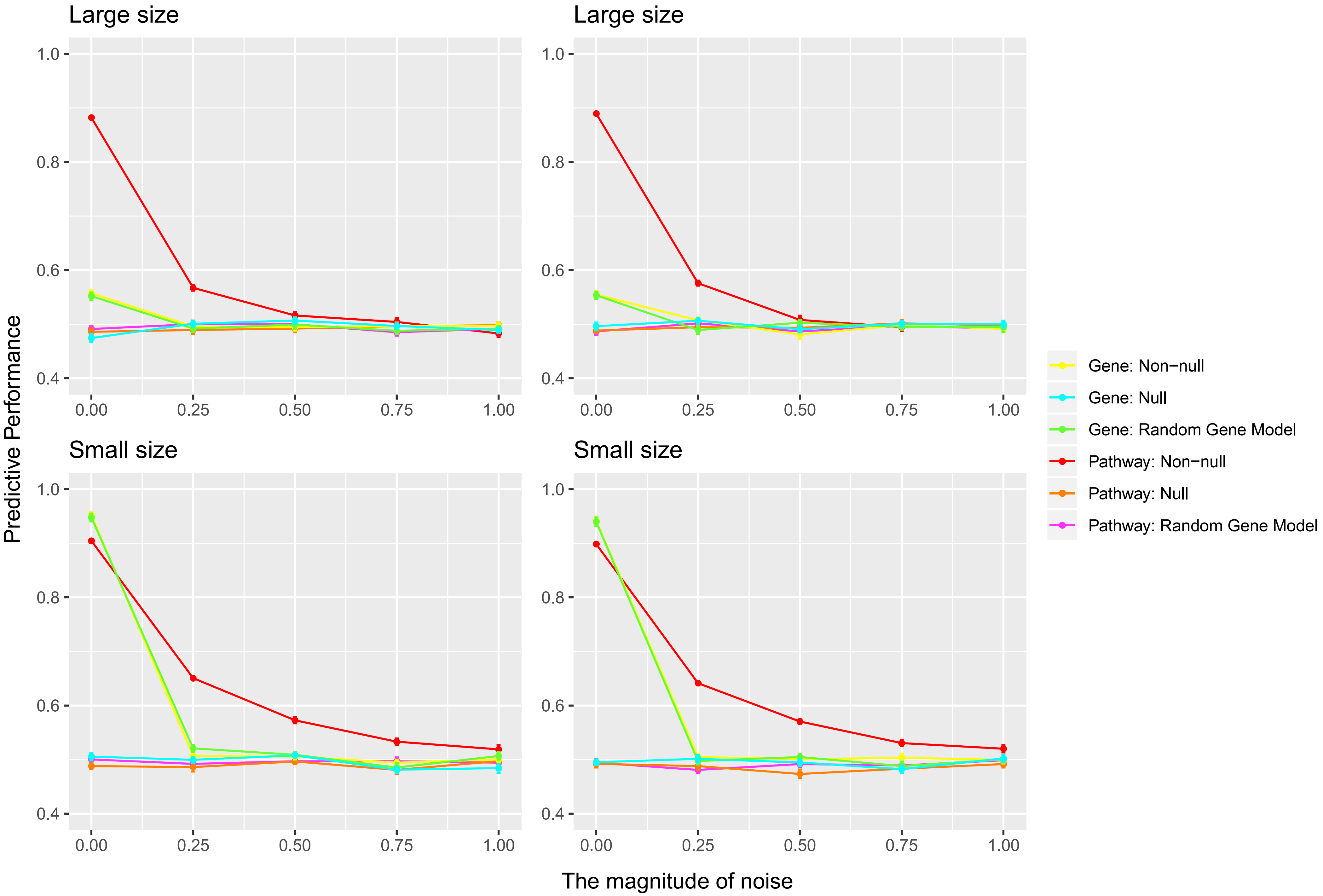

Figure S19: HNSC cohort, simulation 1

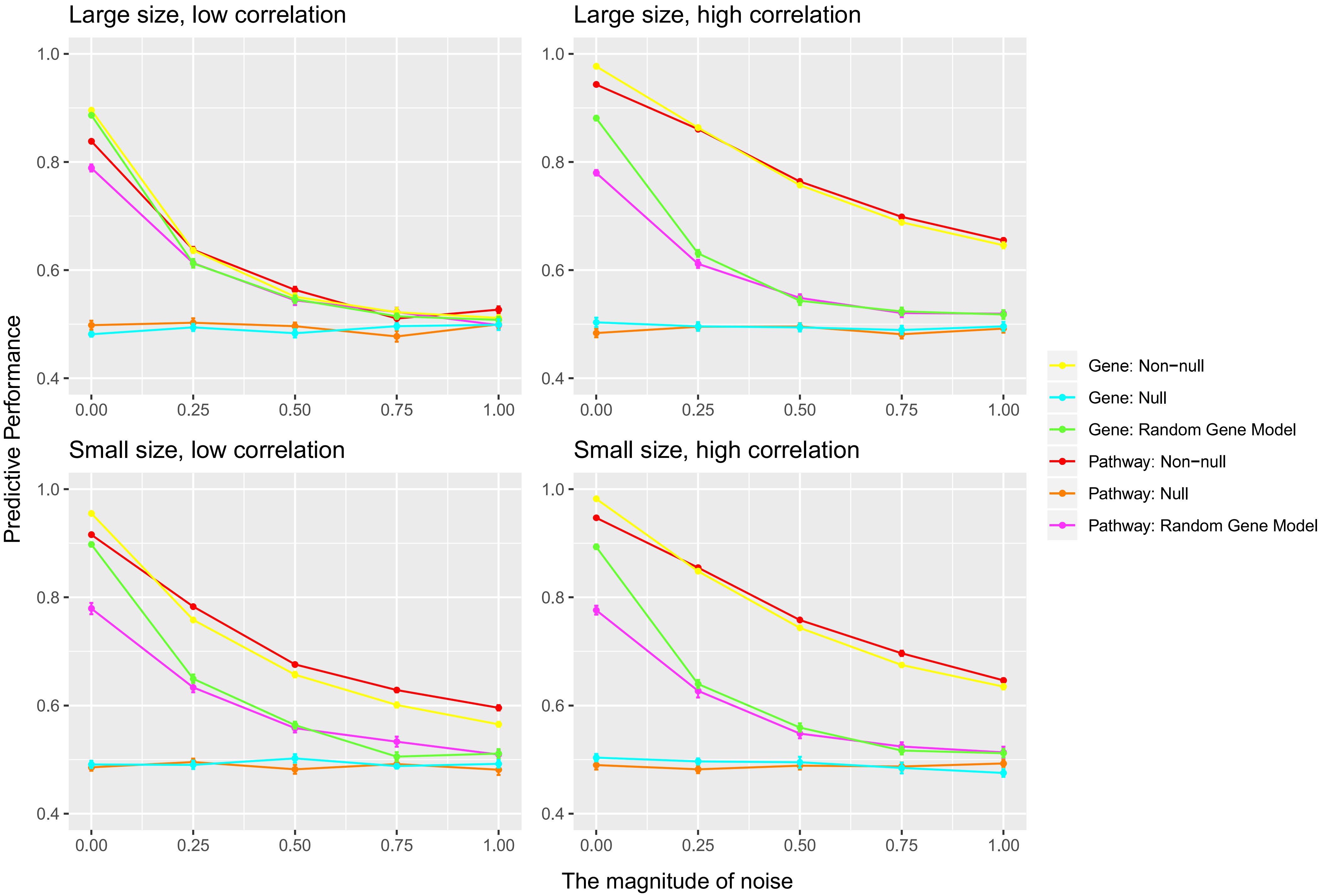

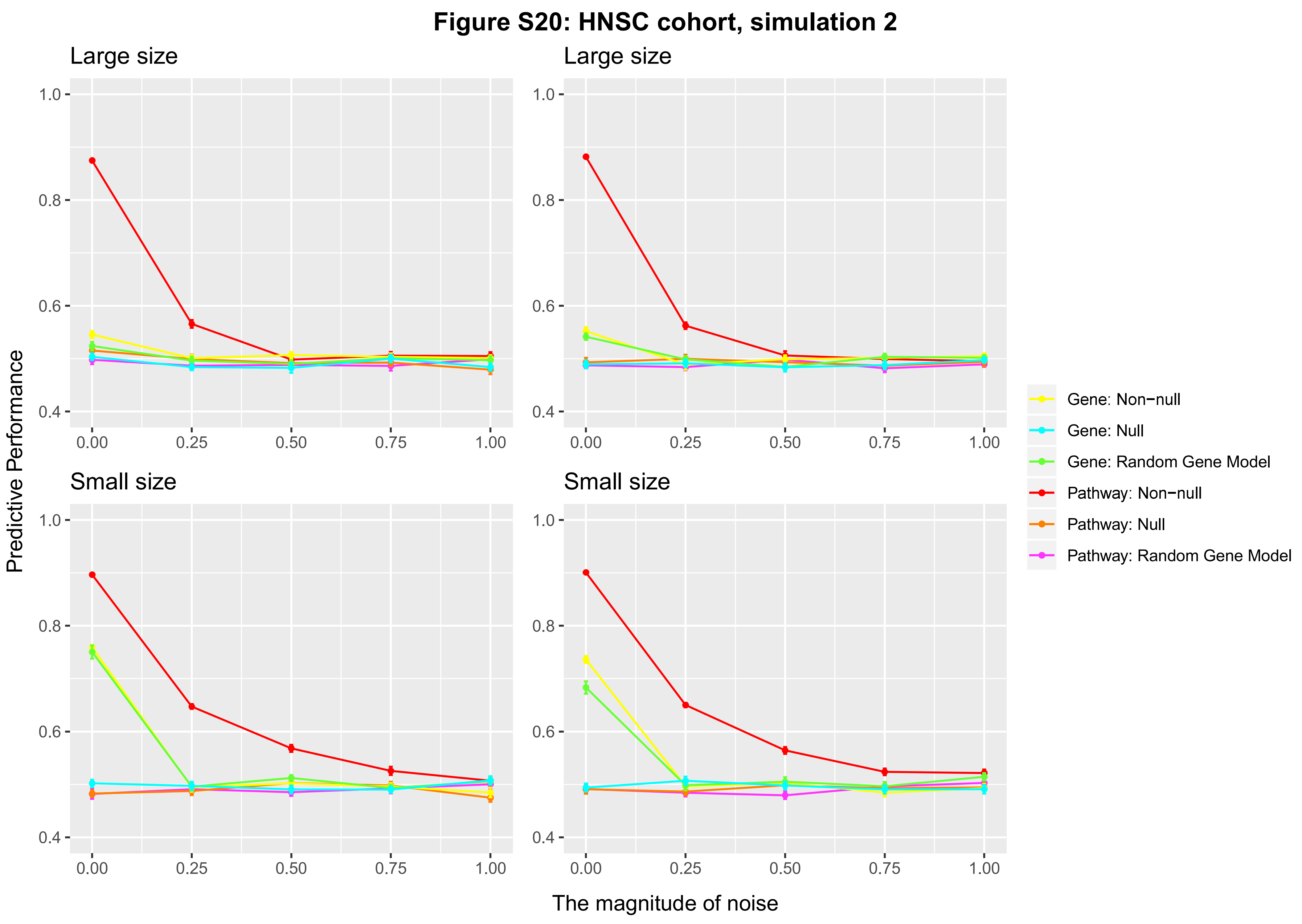

Figure S20: HNSC cohort, simulation 2
Large size
Large size
Small size
Small size
Predictive Performance
The magnitude of noise
1.0
0.8
0.6
0.4
0.00
0.25
0.50
0.75
1.00
Gene: Non−null
Gene: Null
Gene: Random Gene Model
Pathway: Non−null
Pathway: Null
Pathway: Random Gene Model

Figure S21: KICH cohort, simulation 1

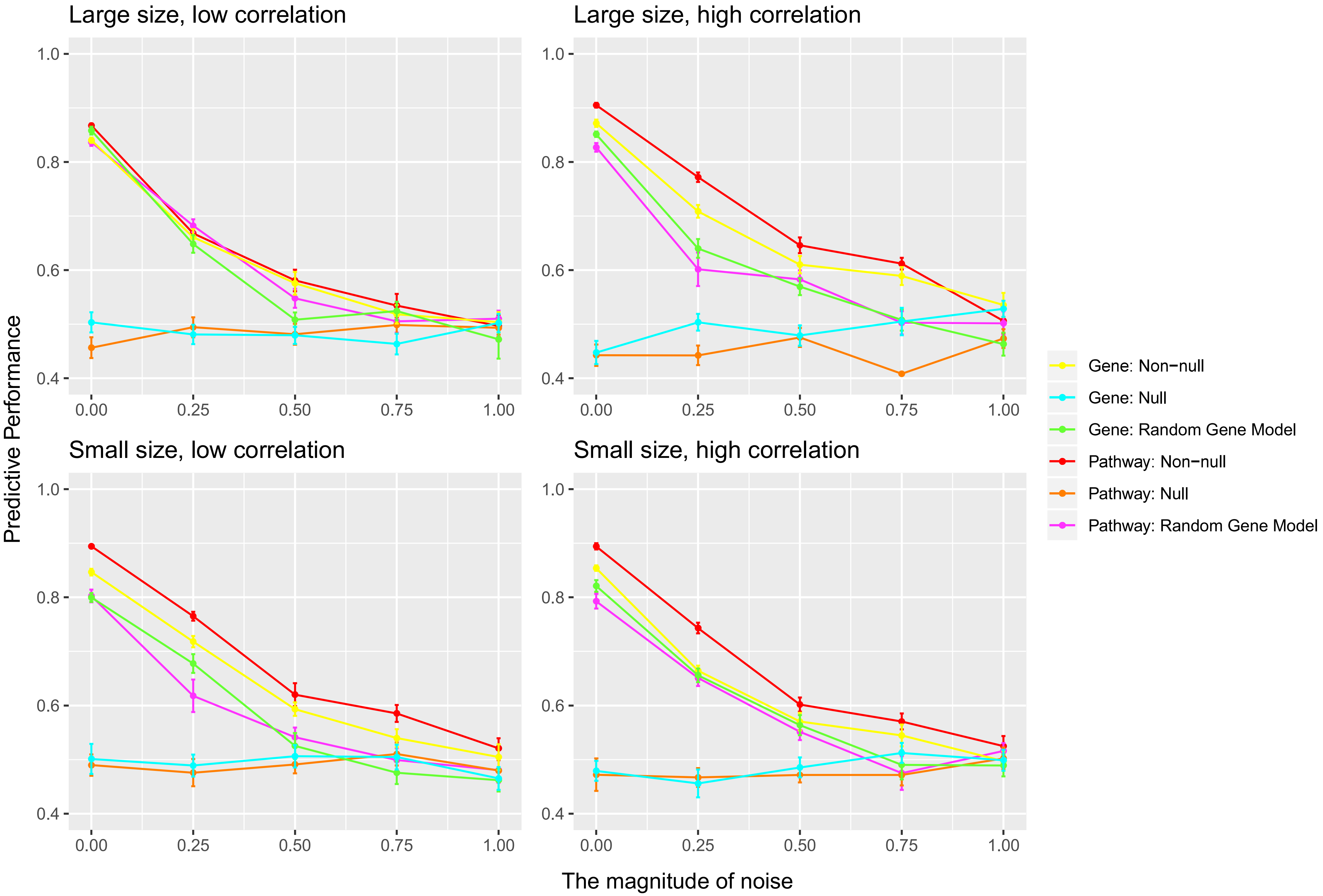

Figure S22: KICH cohort, simulation 2

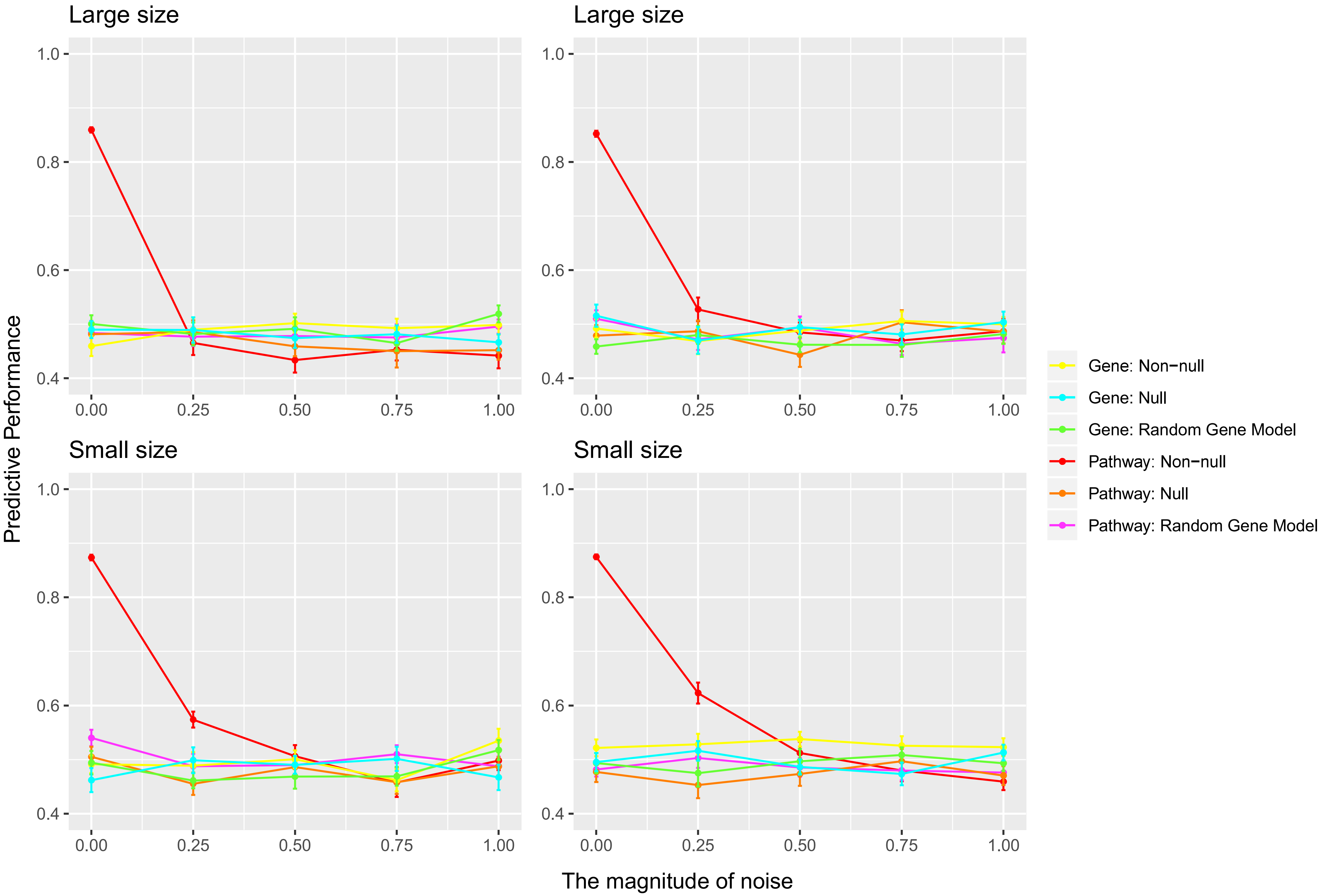

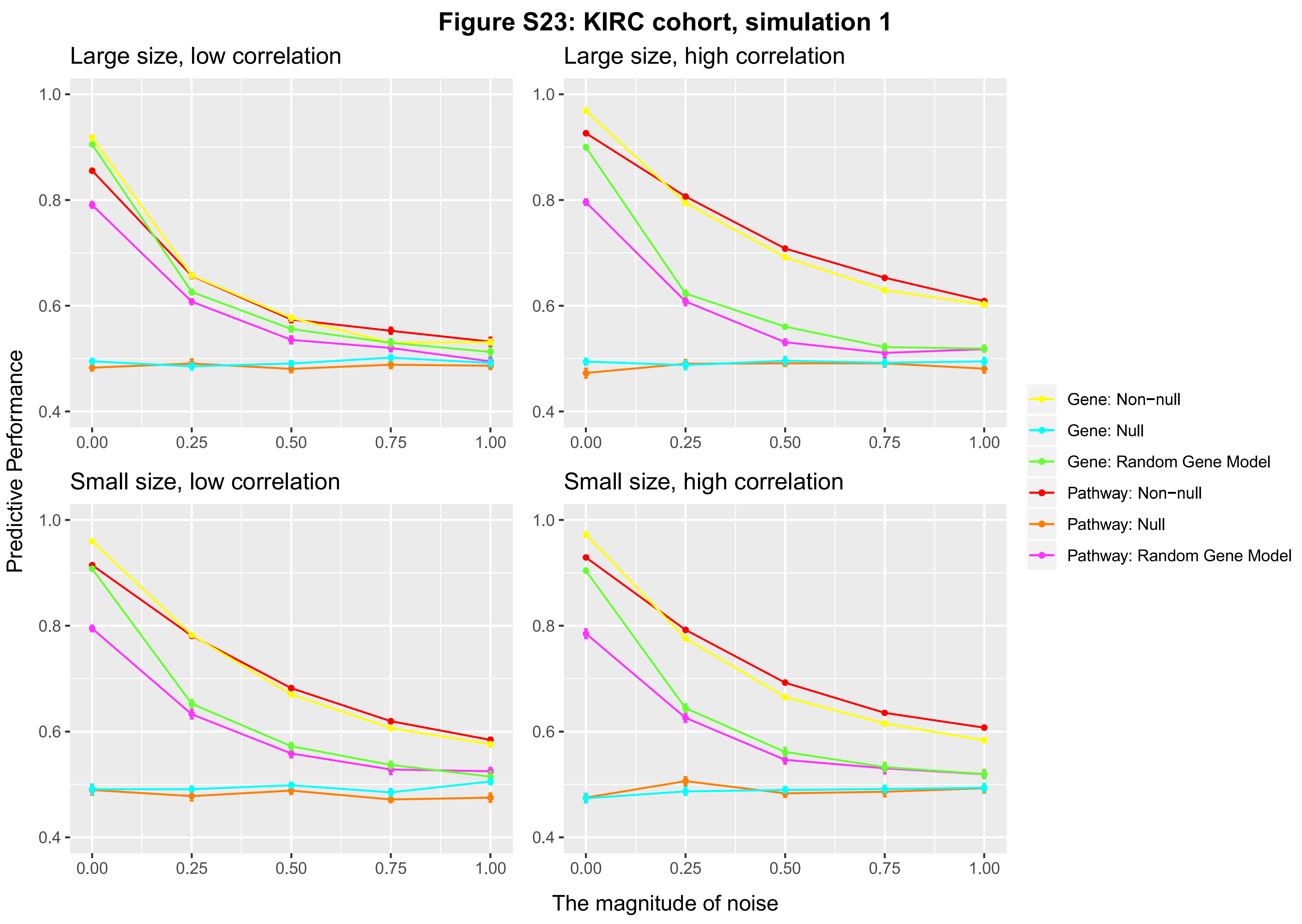

Figure S23: KIRC cohort, simulation 1
Large size, low correlation
Large size, high correlation
Small size, low correlation
Small size, high correlation
Predictive Performance
The magnitude of noise
1.0
0.8
0.6
0.4
0.00
0.25
0.50
0.75
1.00
Gene: Non−null
Gene: Null
Gene: Random Gene Model
Pathway: Non−null
Pathway: Null
Pathway: Random Gene Model

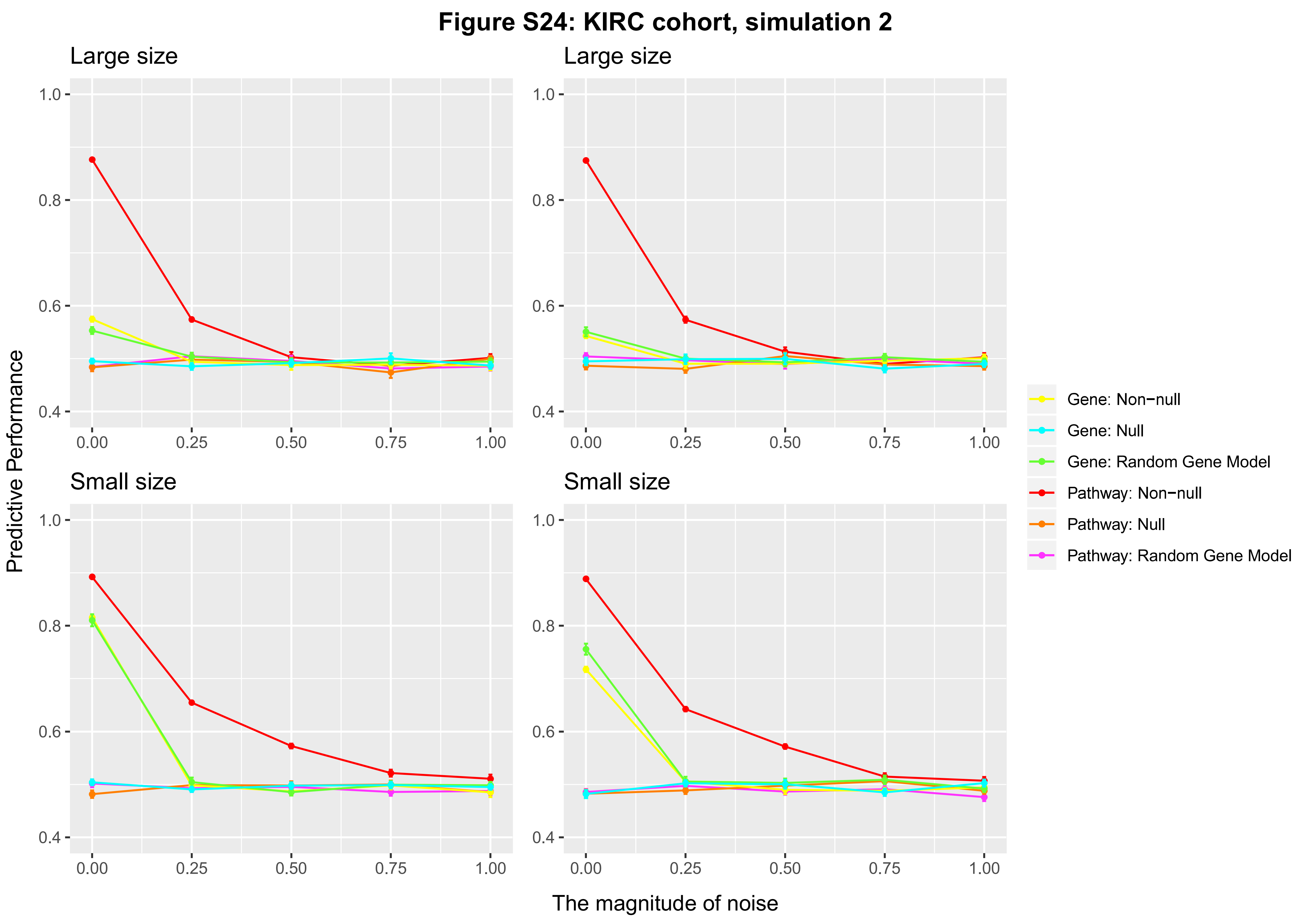
Figure S24: KIRC cohort, simulation 2
Large size
Large size
Small size
Small size
Predictive Performance
The magnitude of noise
1.0
0.8
0.6
0.4
0.00
0.25
0.50
0.75
1.00
Gene: Non−null
Gene: Null
Gene: Random Gene Model
Pathway: Non−null
Pathway: Null
Pathway: Random Gene Model

Figure S25: KIRP cohort, simulation 1

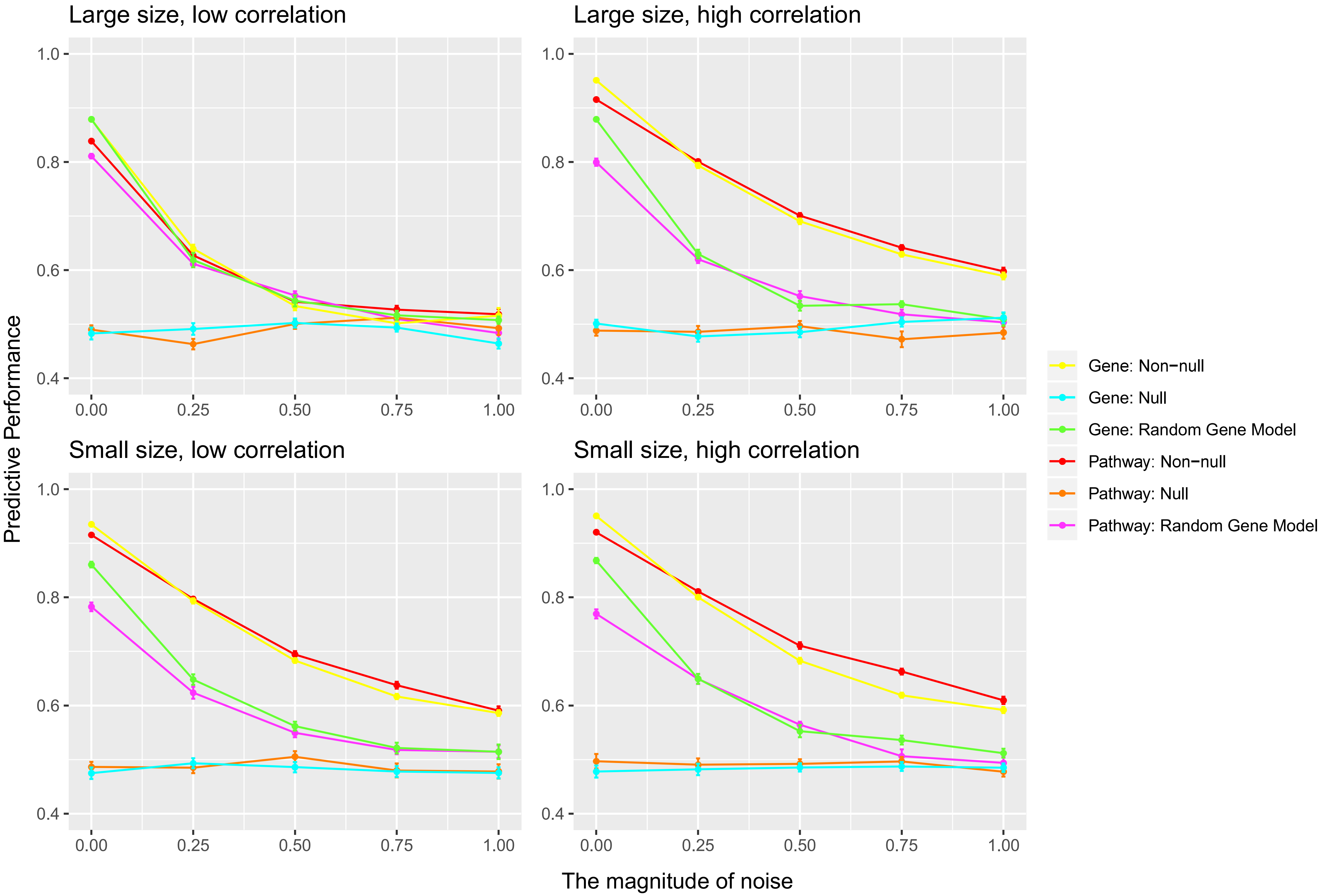

Figure S26: KIRP cohort, simulation 2

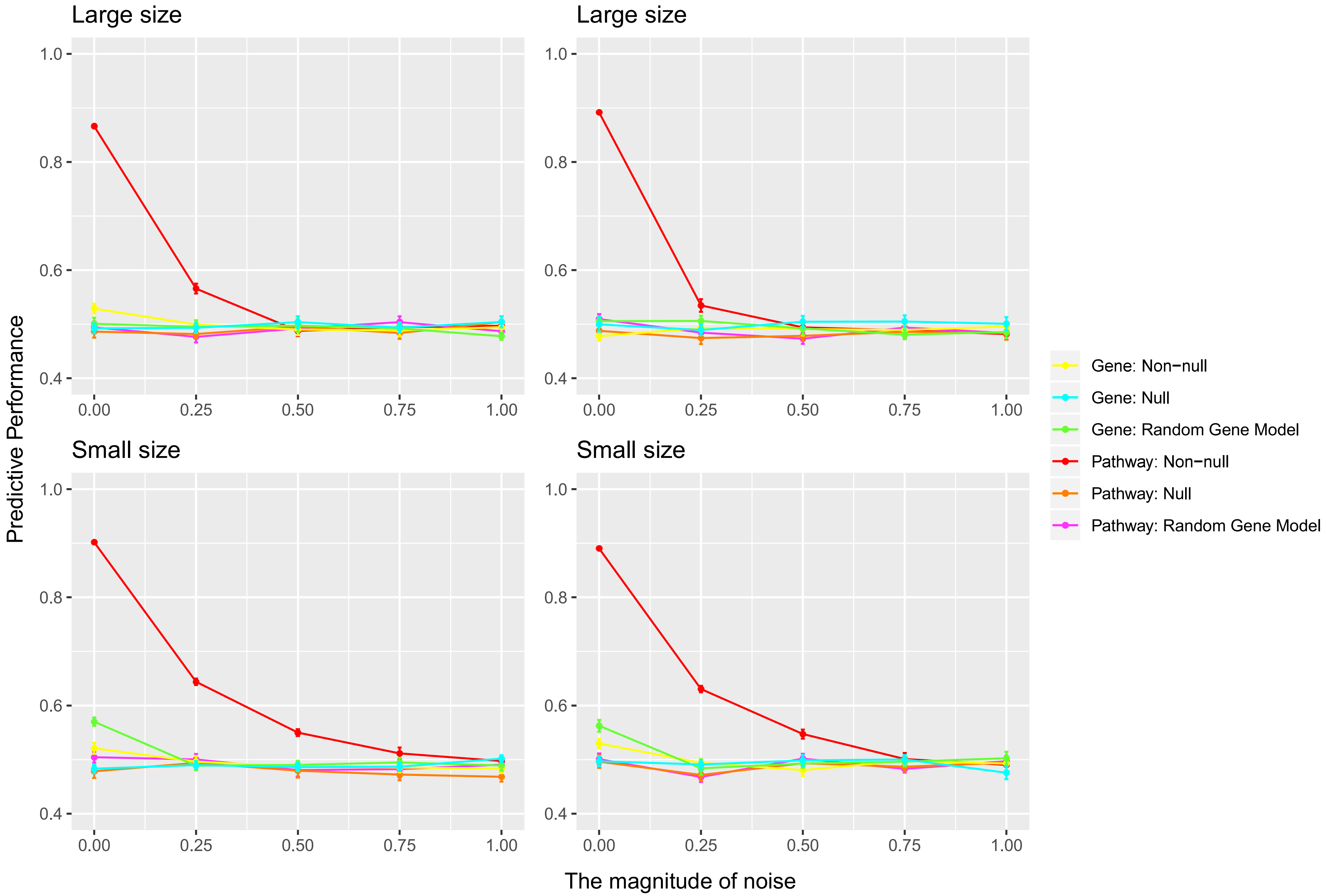

Figure S27: LAML cohort, simulation 1

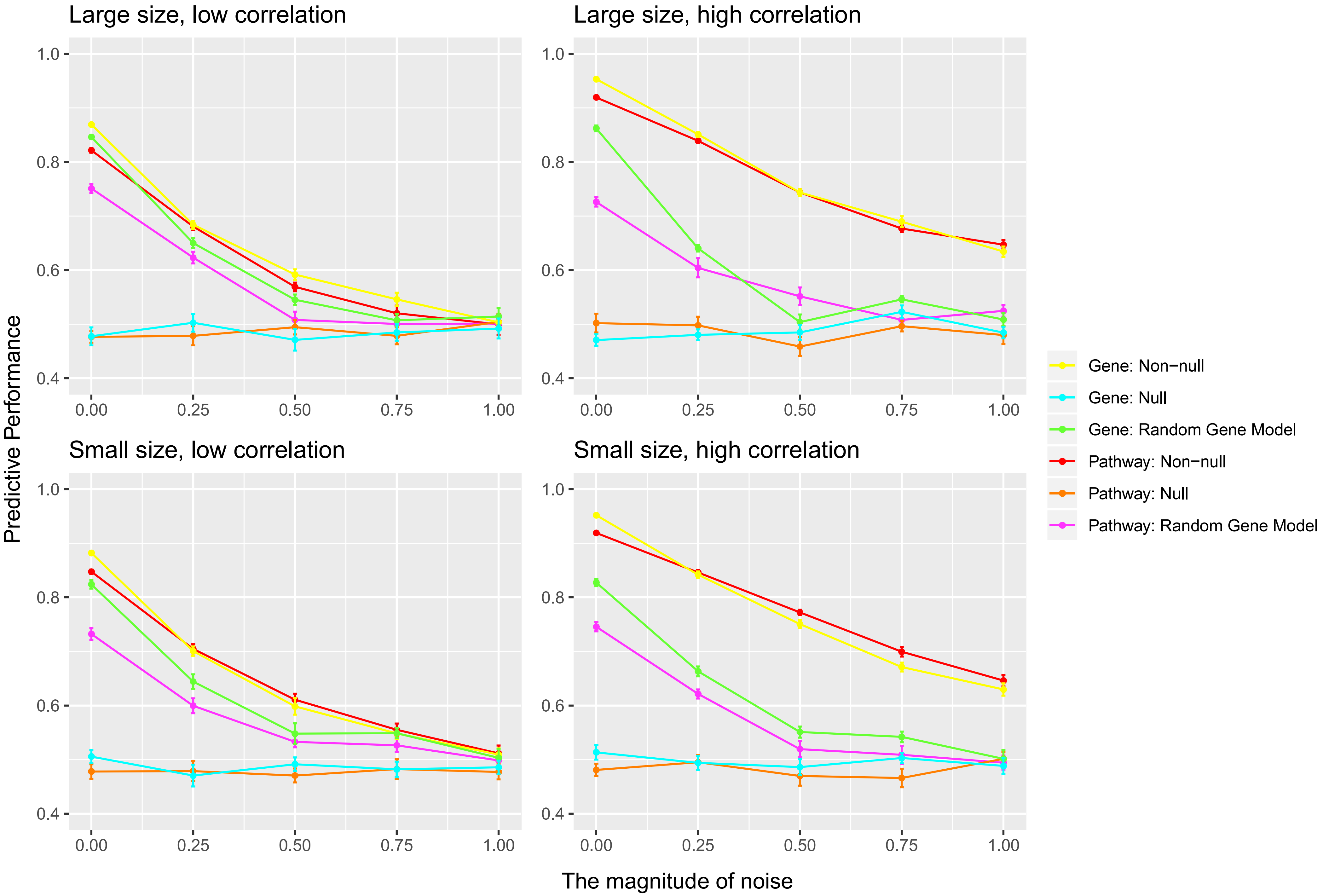

Figure S28: LAML cohort, simulation 2

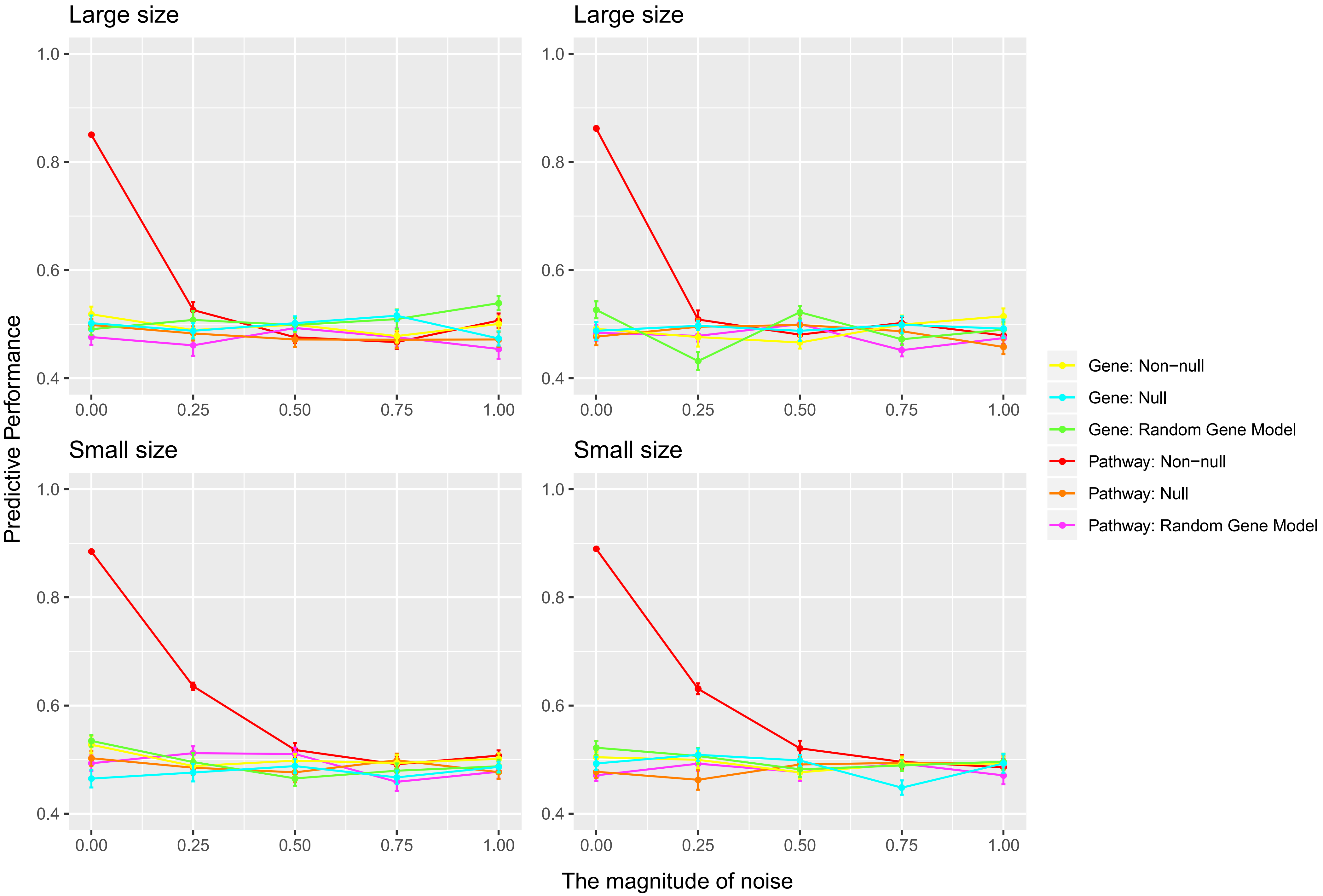

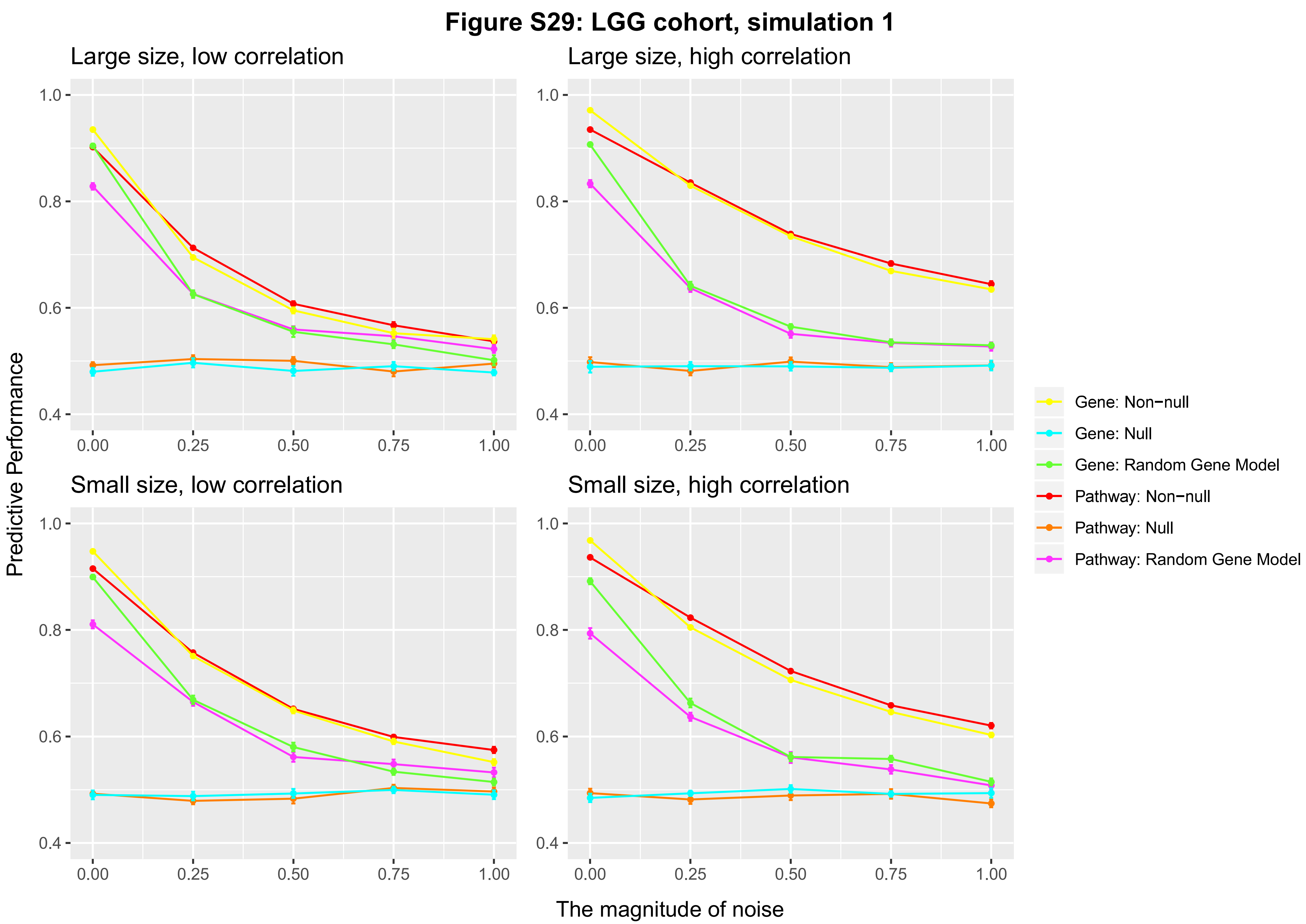

Figure S29: LGG cohort, simulation 1
Large size, low correlation
Large size, high correlation
Small size, low correlation
Small size, high correlation
Predictive Performance
The magnitude of noise
1.0
0.8
0.6
0.4
0.00
0.25
0.50
0.75
1.00
Gene: Non−null
Gene: Null
Gene: Random Gene Model
Pathway: Non−null
Pathway: Null
Pathway: Random Gene Model

Figure S30: LGG cohort, simulation 2

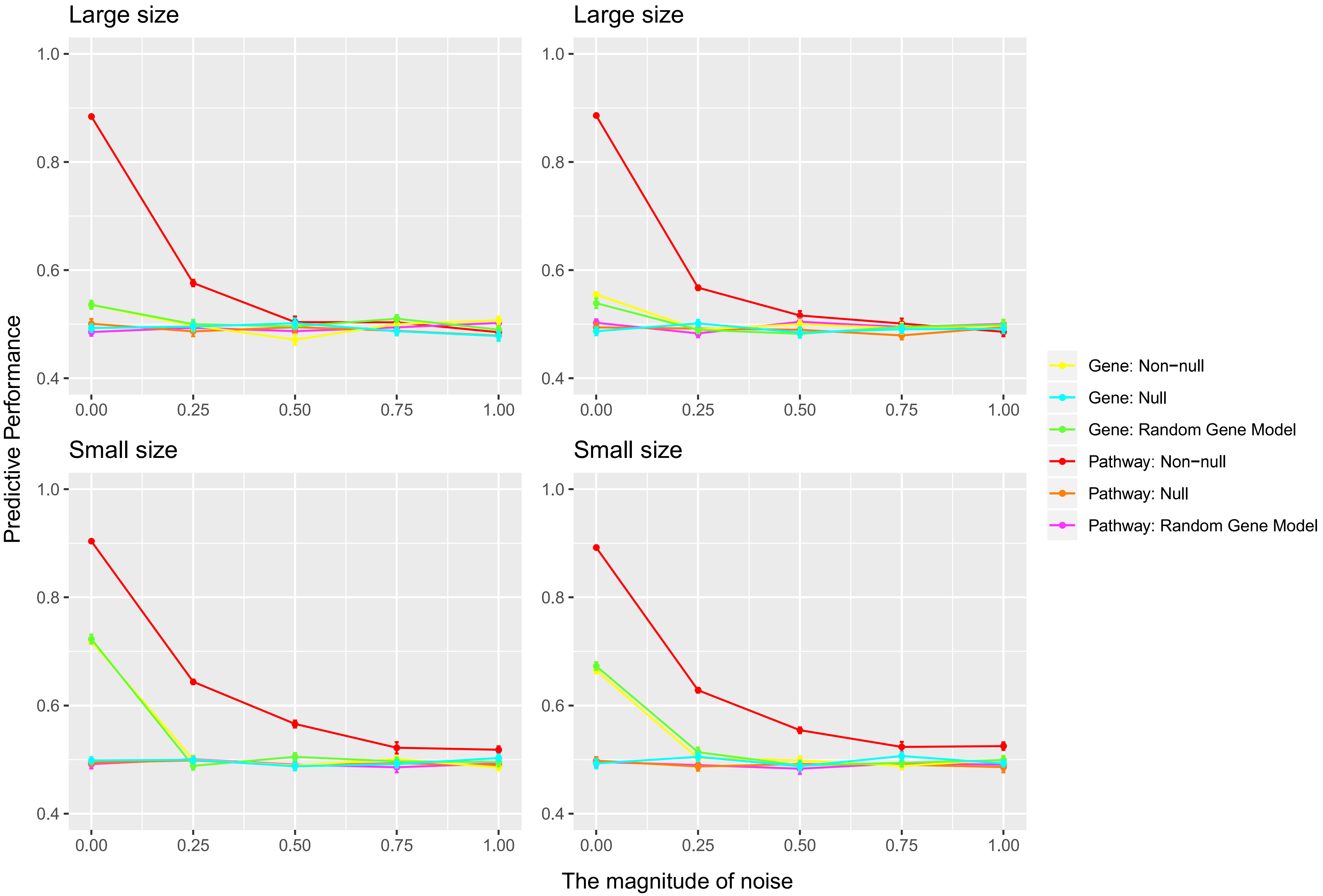

Figure S31: LIHC cohort, simulation 1

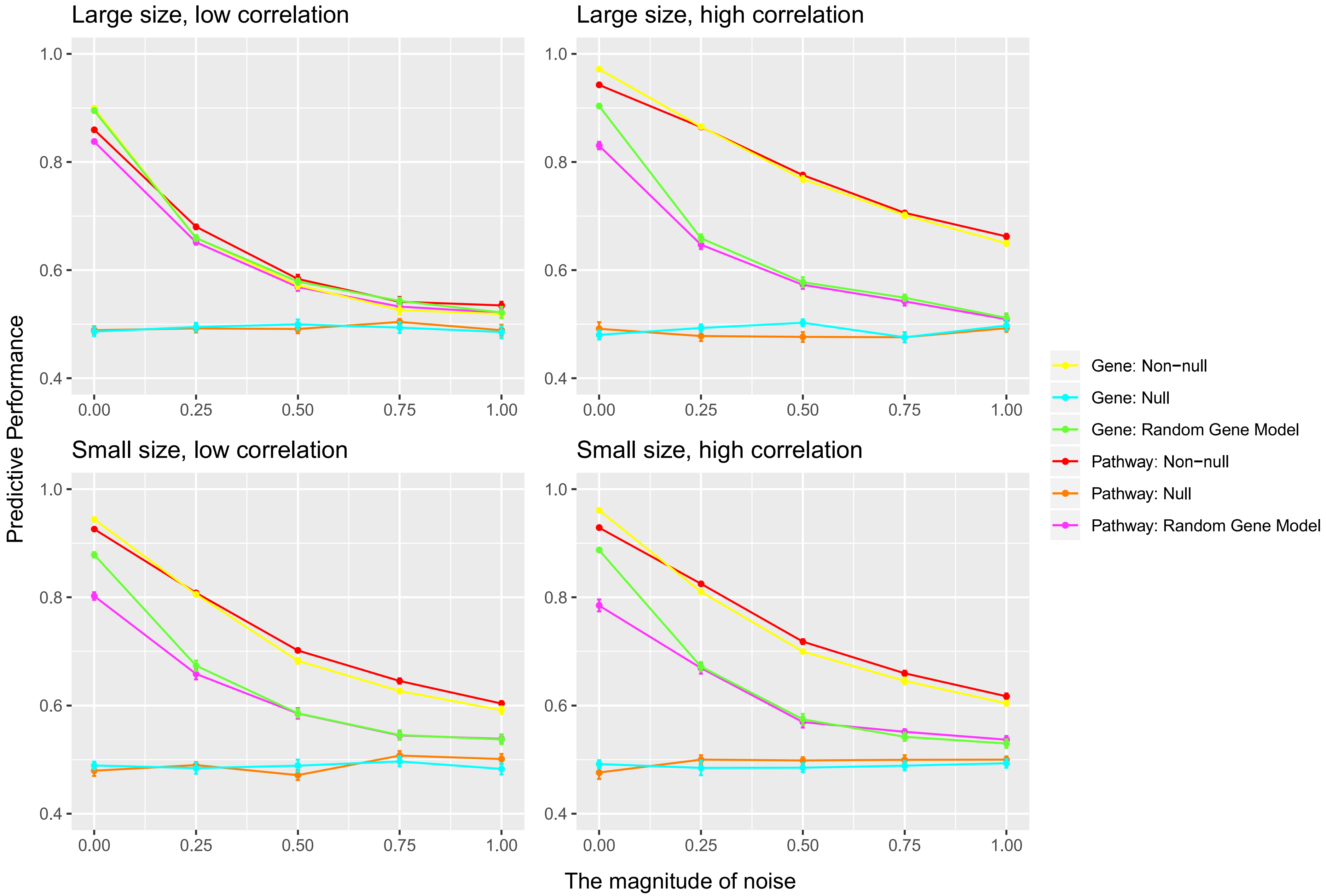

Figure S32: LIHC cohort, simulation 2

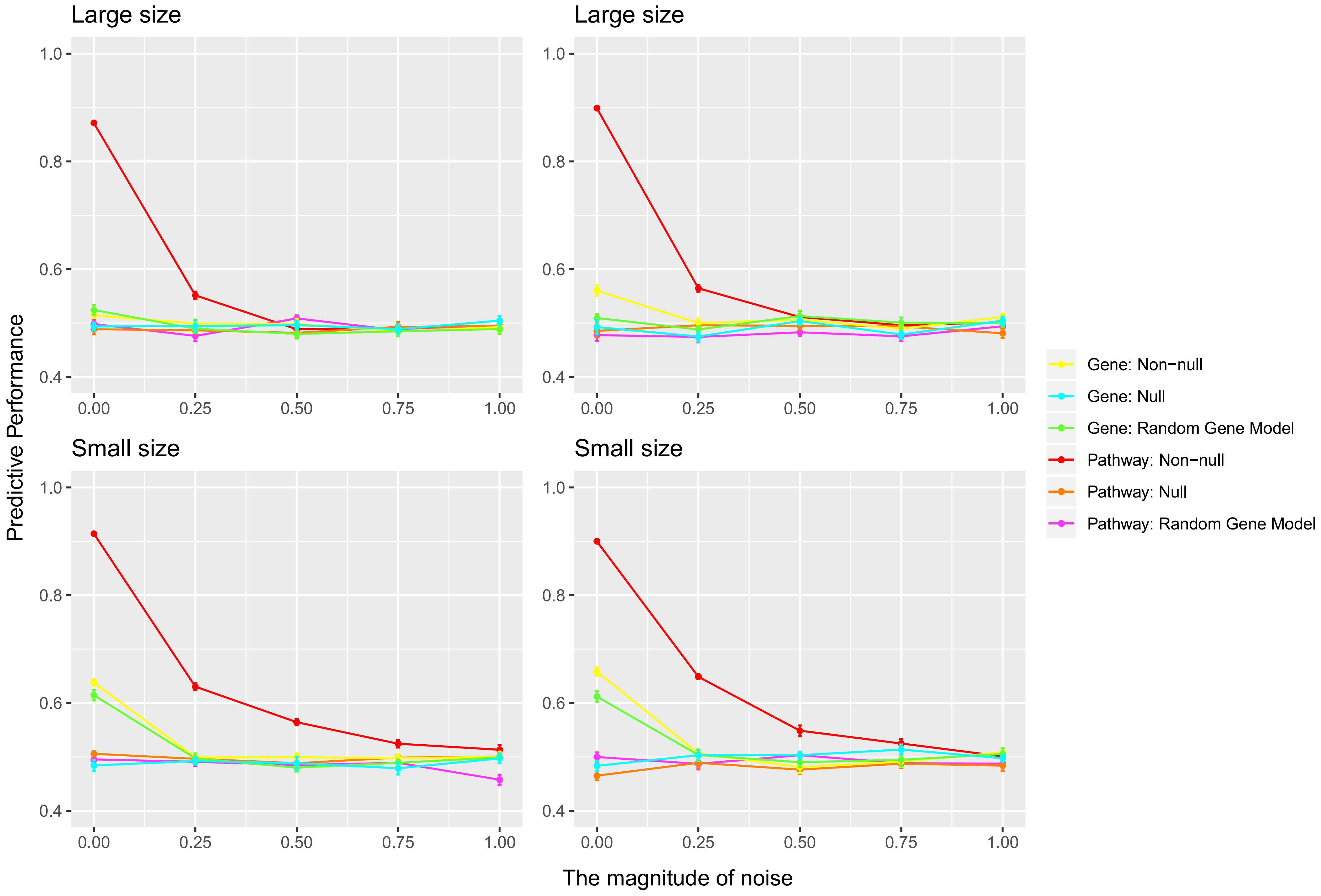

Figure S33: LUAD cohort, simulation 1

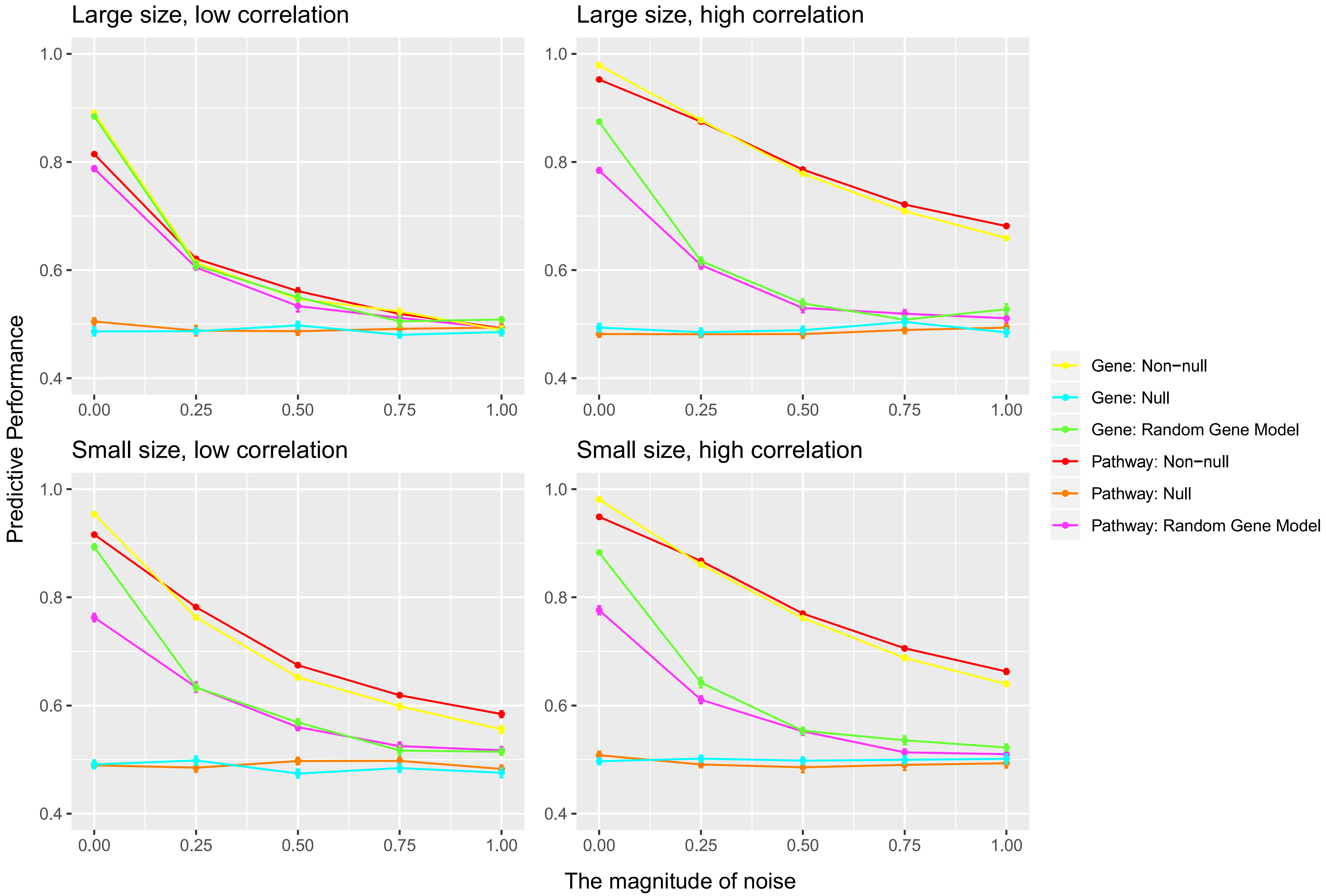

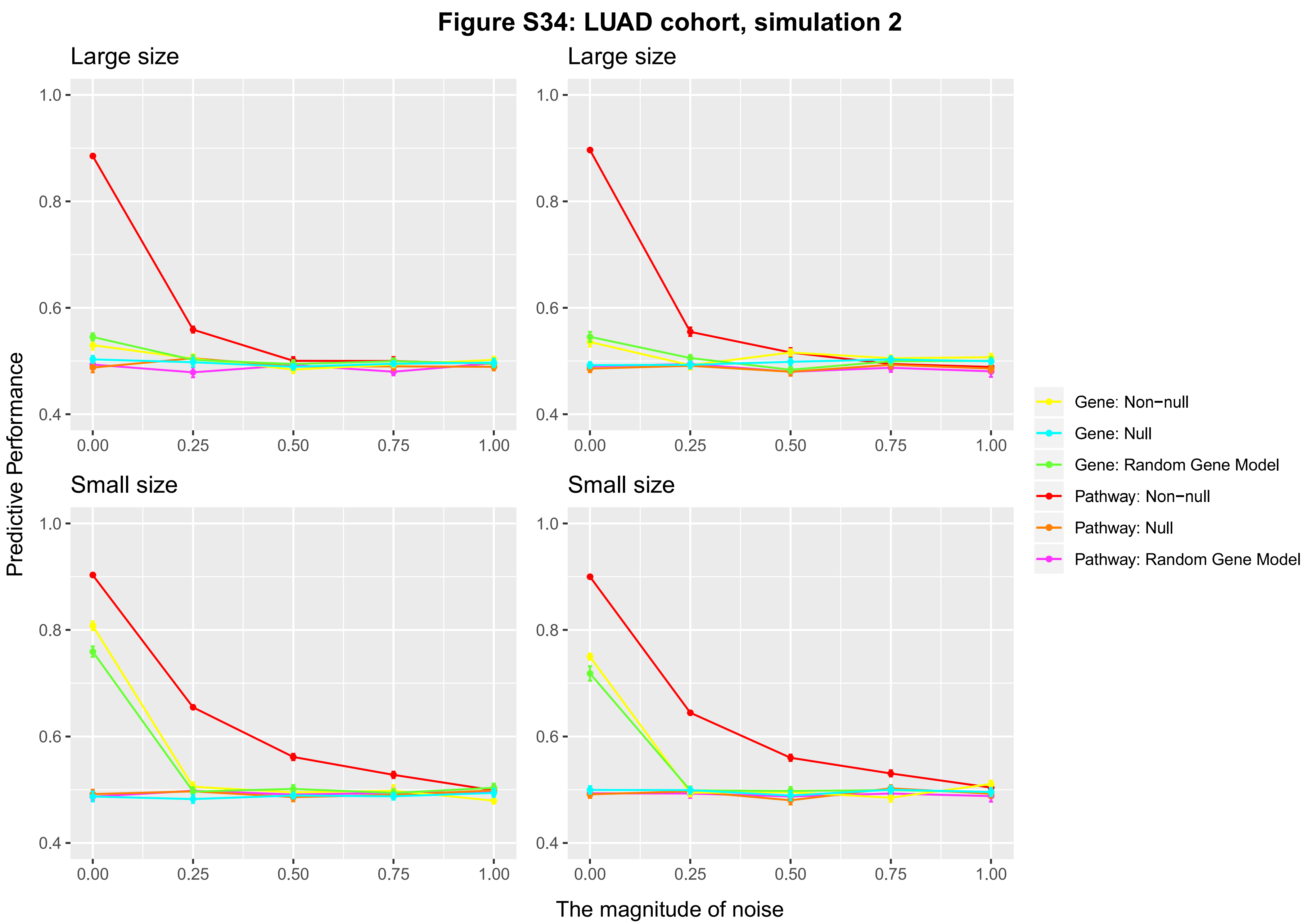

Figure S34: LUAD cohort, simulation 2
Large size
Large size
Small size
Small size
1.0
0.8
0.6
0.4
0.00
0.25
0.50
0.75
1.00
Predictive Performance
The magnitude of noise
Gene: Non−null
Gene: Null
Gene: Random Gene Model
Pathway: Non−null
Pathway: Null
Pathway: Random Gene Model

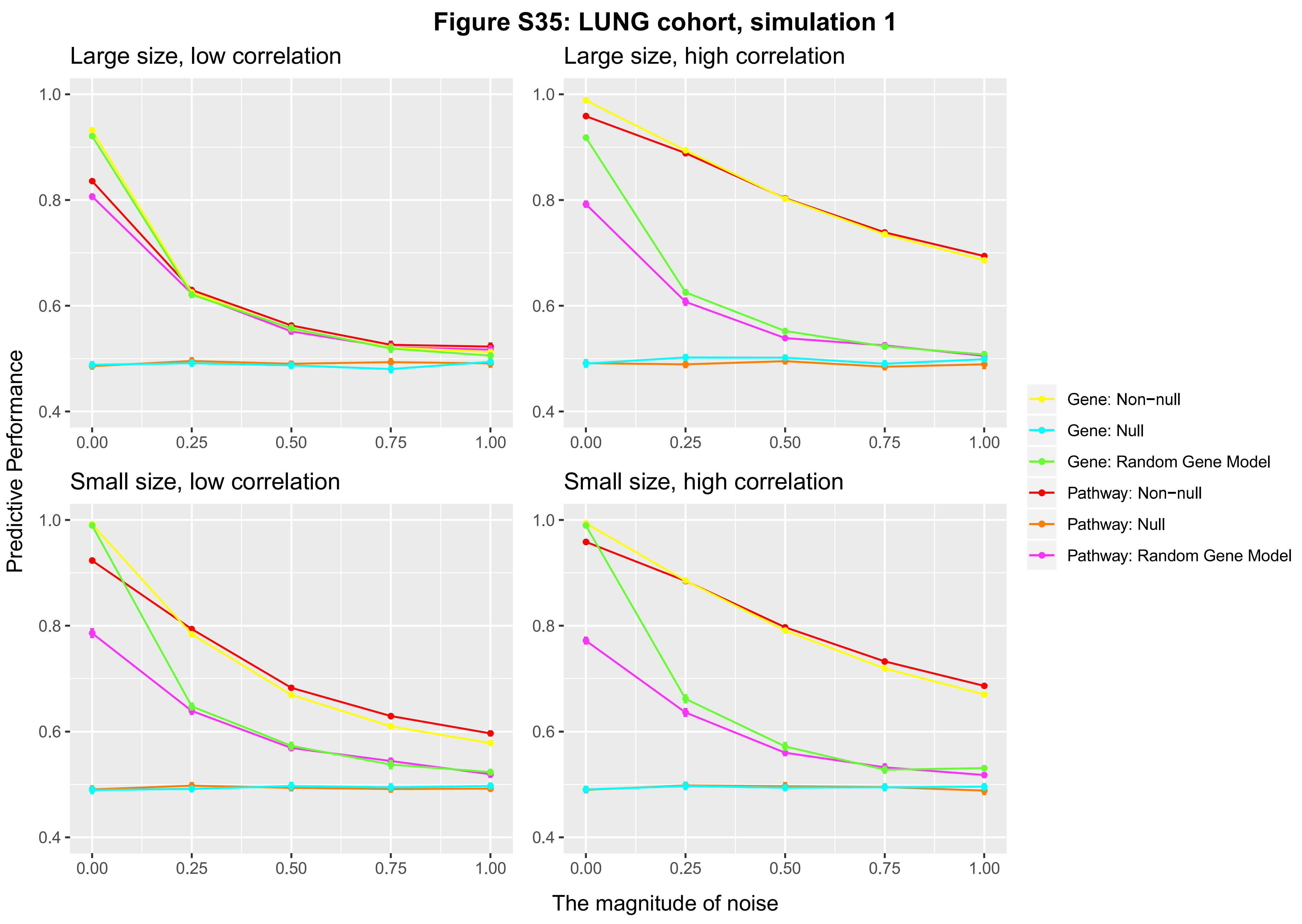
Figure S35: LUNG cohort, simulation 1
Large size, low correlation
Large size, high correlation
Small size, low correlation
Small size, high correlation
Predictive Performance
The magnitude of noise
1.0
0.8
0.6
0.4
0.00
0.25
0.50
0.75
1.00
Gene: Non−null
Gene: Null
Gene: Random Gene Model
Pathway: Non−null
Pathway: Null
Pathway: Random Gene Model

Figure S36: LUNG cohort, simulation 2

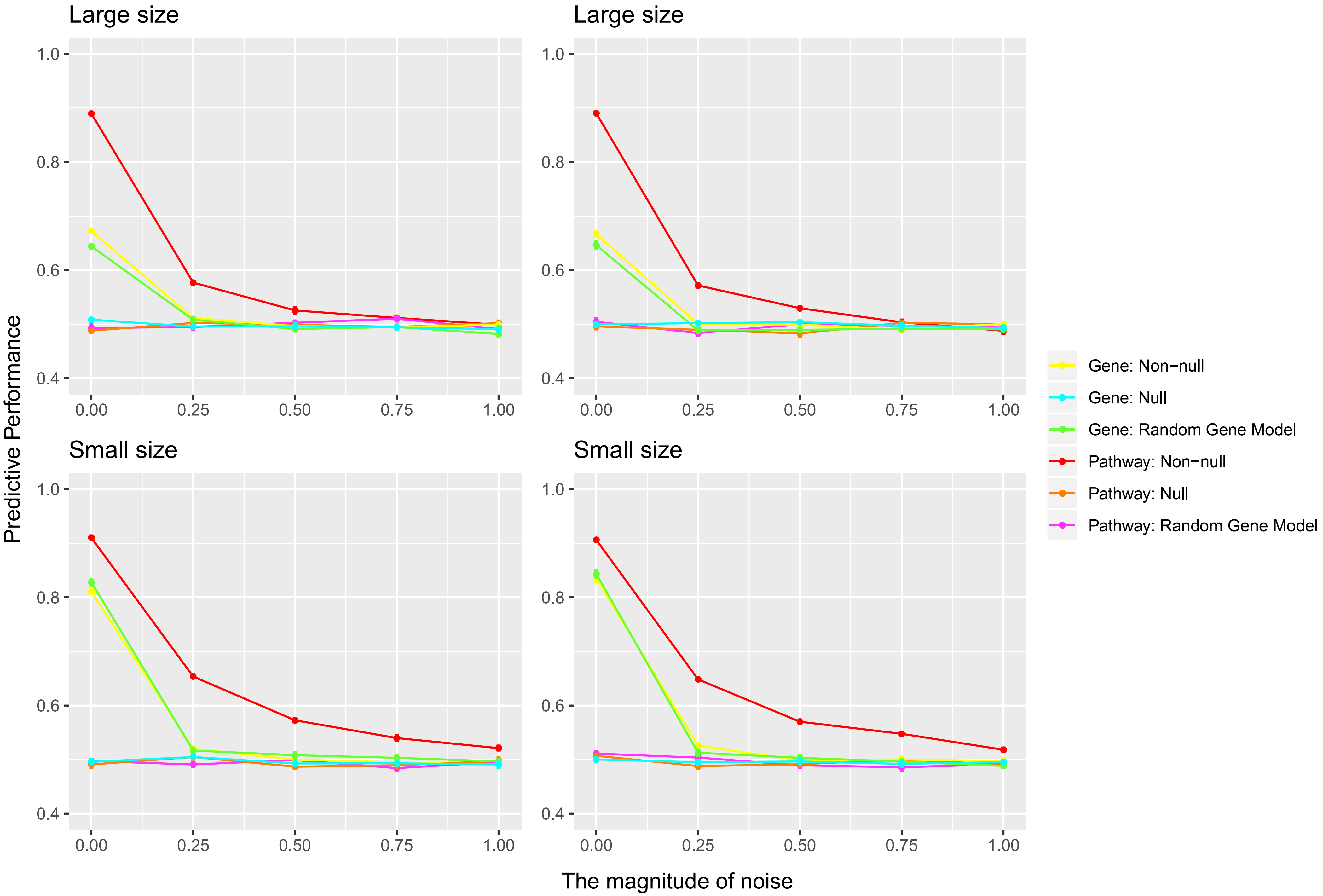

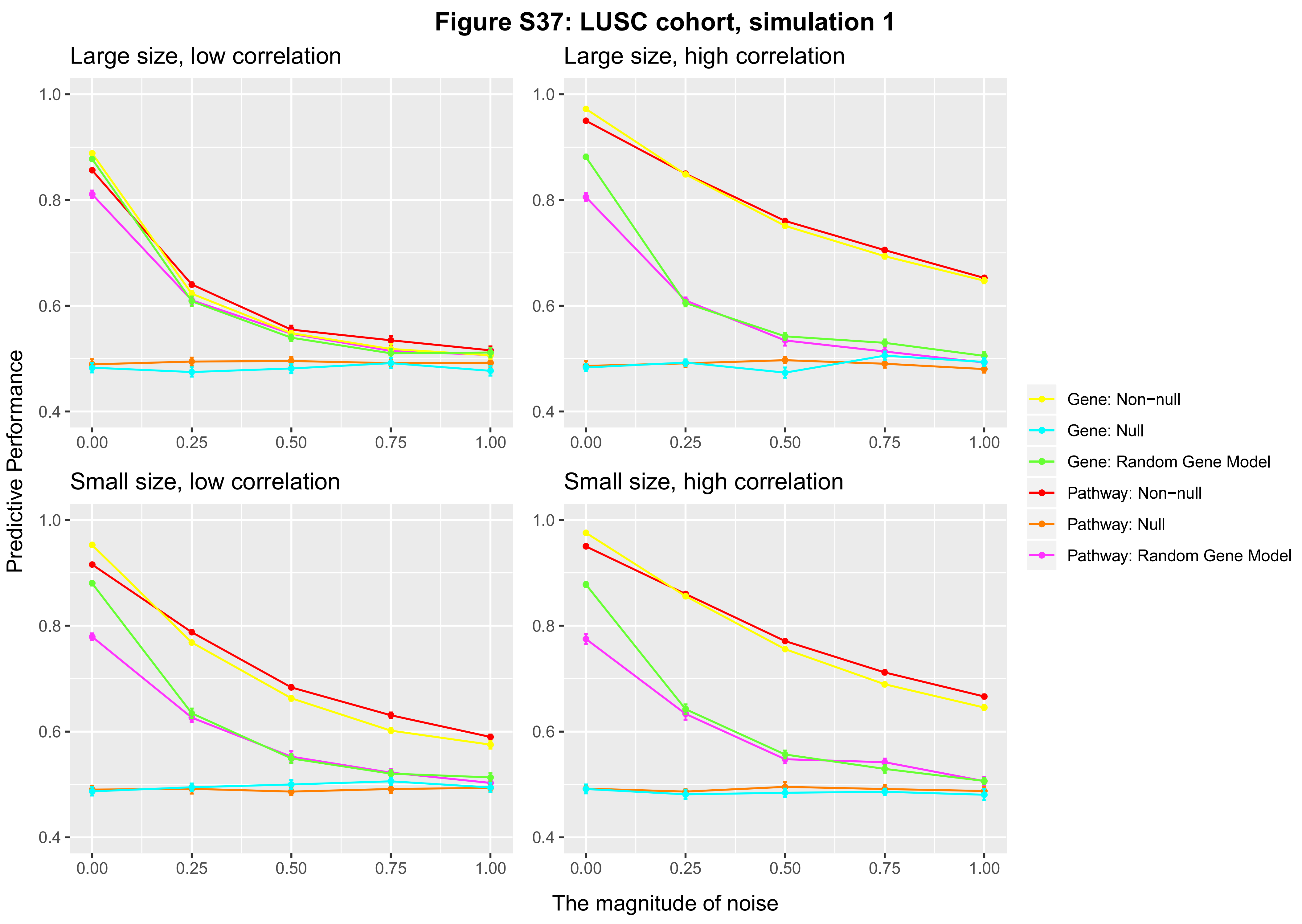

Figure S37: LUSC cohort, simulation 1
Large size, low correlation
Large size, high correlation
Small size, low correlation
Small size, high correlation
Predictive Performance
The magnitude of noise
1.0
0.8
0.6
0.4
0.00
0.25
0.50
0.75
1.00
Gene: Non−null
Gene: Null
Gene: Random Gene Model
Pathway: Non−null
Pathway: Null
Pathway: Random Gene Model

Figure S38: LUSC cohort, simulation 2

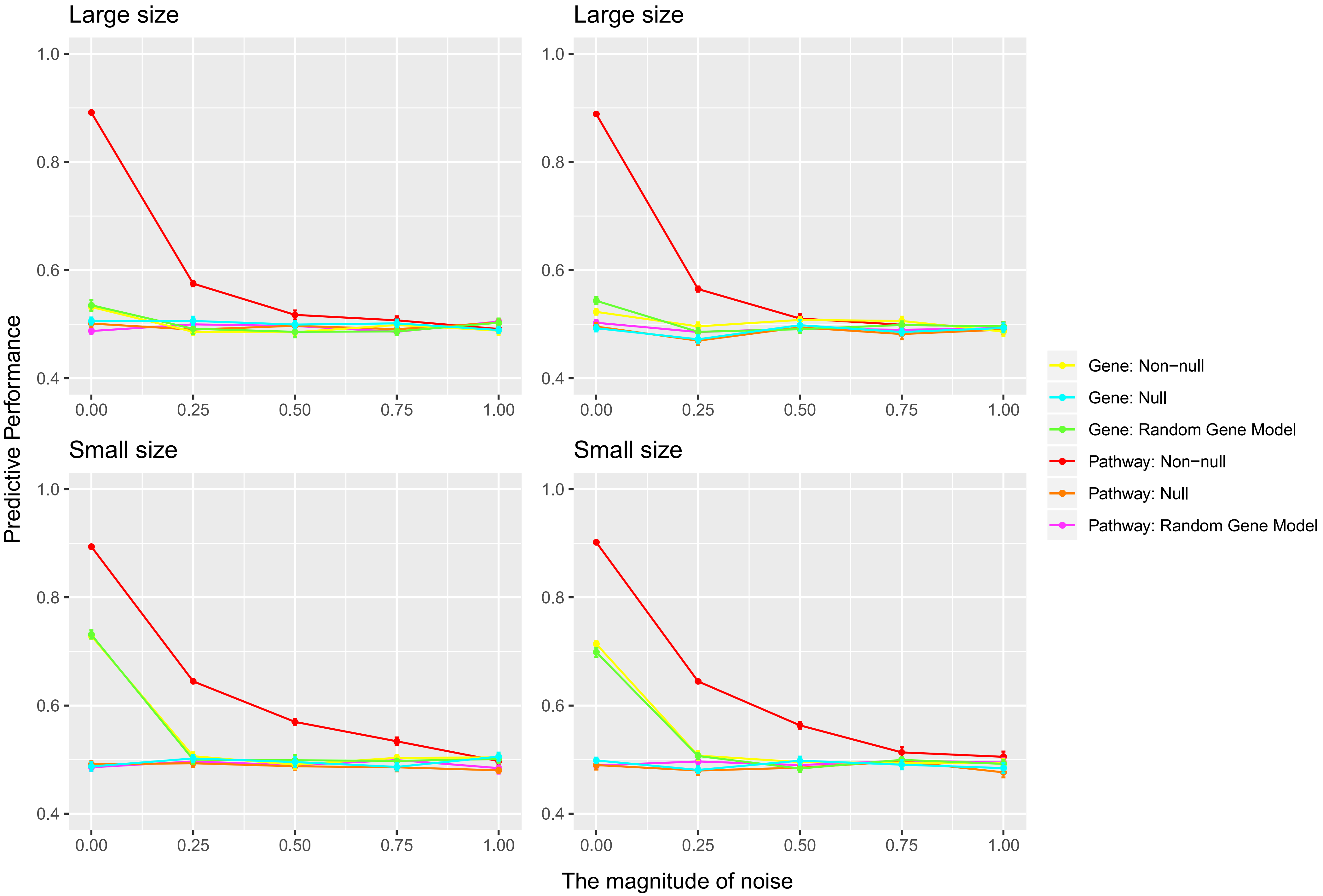

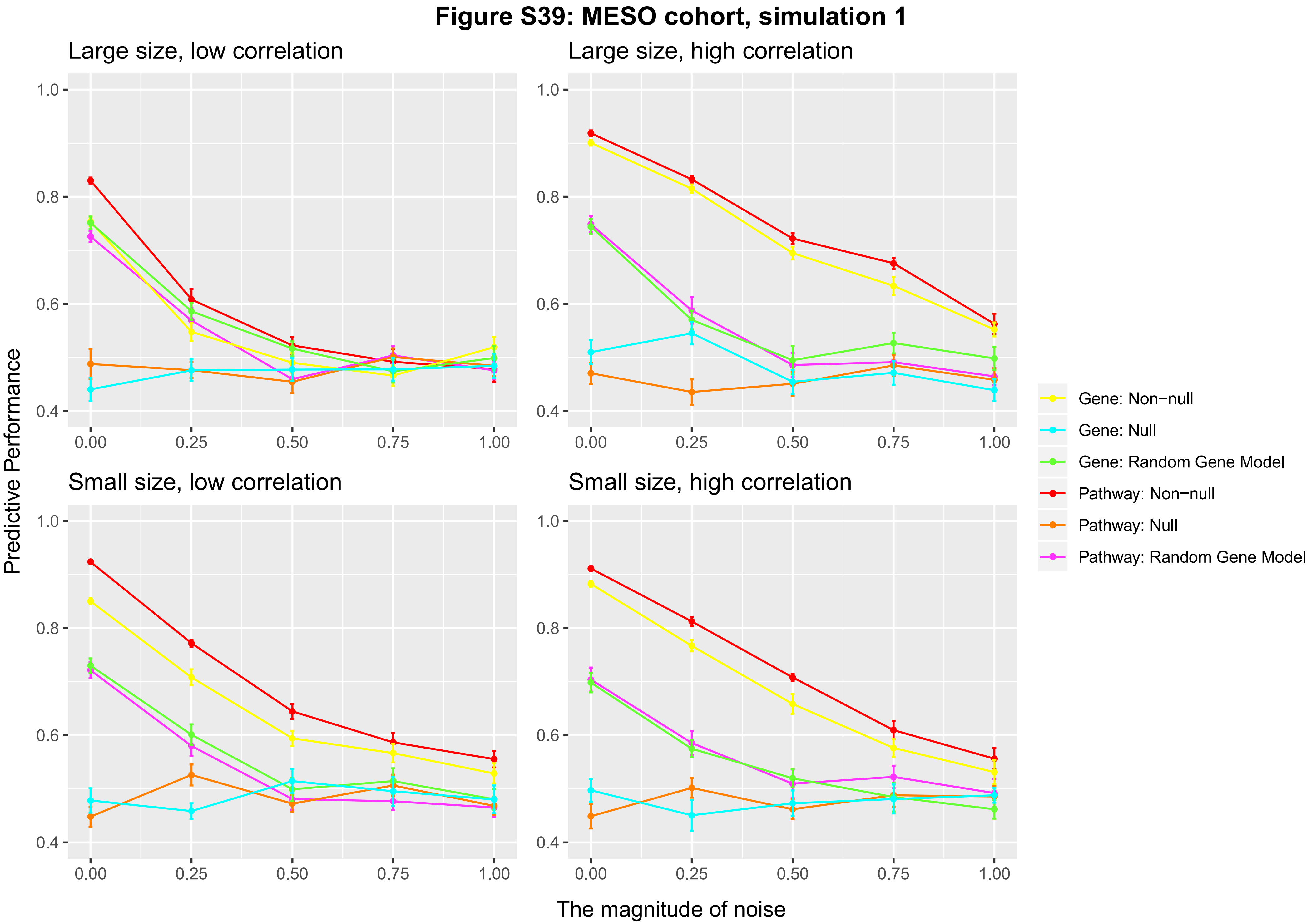

Figure S39: MESO cohort, simulation 1
Large size, low correlation
Large size, high correlation
Small size, low correlation
Small size, high correlation
Predictive Performance
The magnitude of noise
1.0
0.8
0.6
0.4
0.00
0.25
0.50
0.75
1.00
Gene: Non−null
Gene: Null
Gene: Random Gene Model
Pathway: Non−null
Pathway: Null
Pathway: Random Gene Model

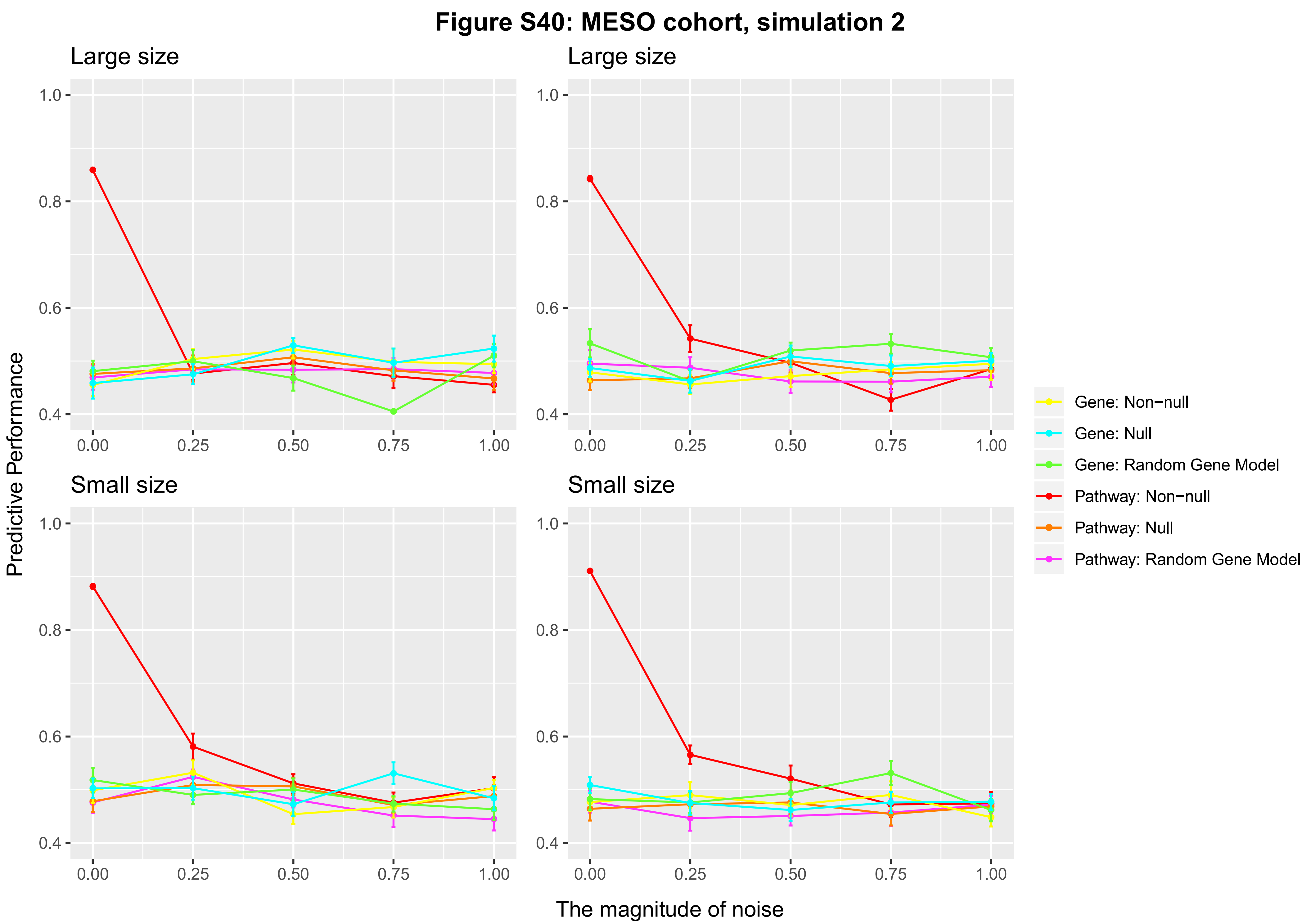

Figure S40: MESO cohort, simulation 2
Large size
Large size
Small size
Small size
Predictive Performance
The magnitude of noise
1.0
0.8
0.6
0.4
0.00
0.25
0.50
0.75
1.00
Gene: Non−null
Gene: Null
Gene: Random Gene Model
Pathway: Non−null
Pathway: Null
Pathway: Random Gene Model

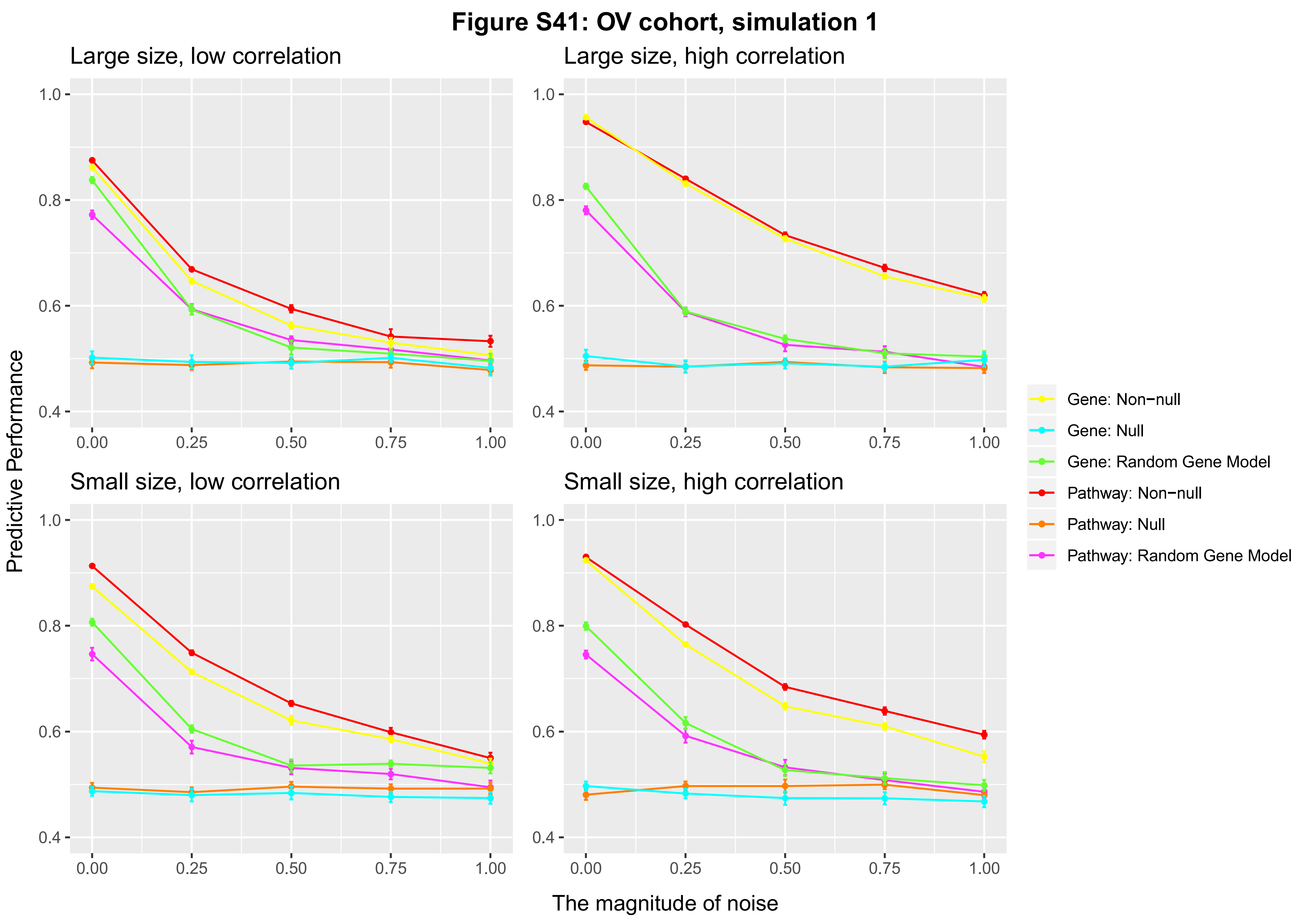

Figure S41: OV cohort, simulation 1
Large size, low correlation
Large size, high correlation
Small size, low correlation
Small size, high correlation
Predictive Performance
The magnitude of noise
0.4
0.6
0.8
1.0
0.00
0.25
0.50
0.75
1.00
Gene: Non−null
Gene: Null
Gene: Random Gene Model
Pathway: Non−null
Pathway: Null
Pathway: Random Gene Model

Figure S42: OV cohort, simulation 2

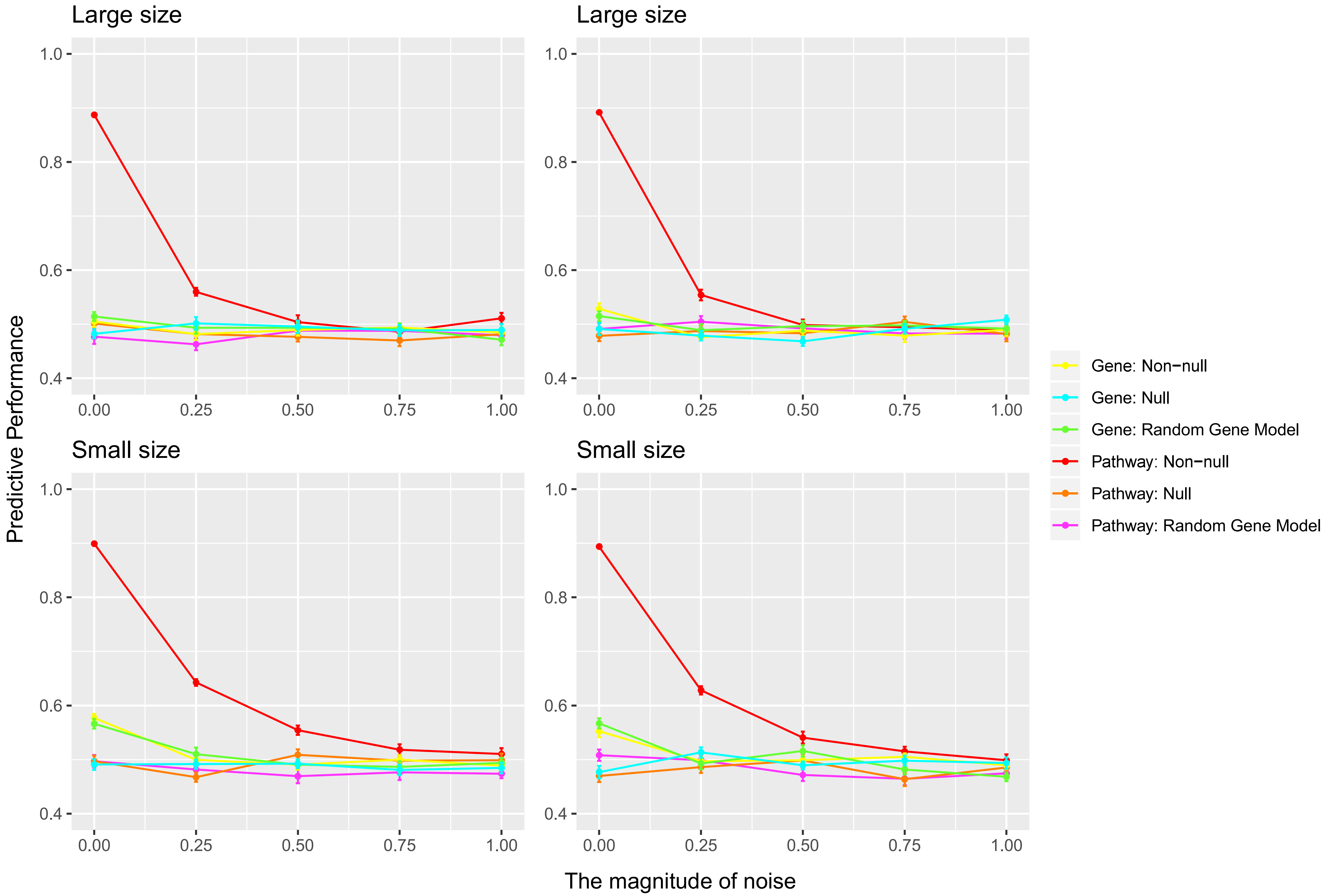

Figure S43: PAAD cohort, simulation 1

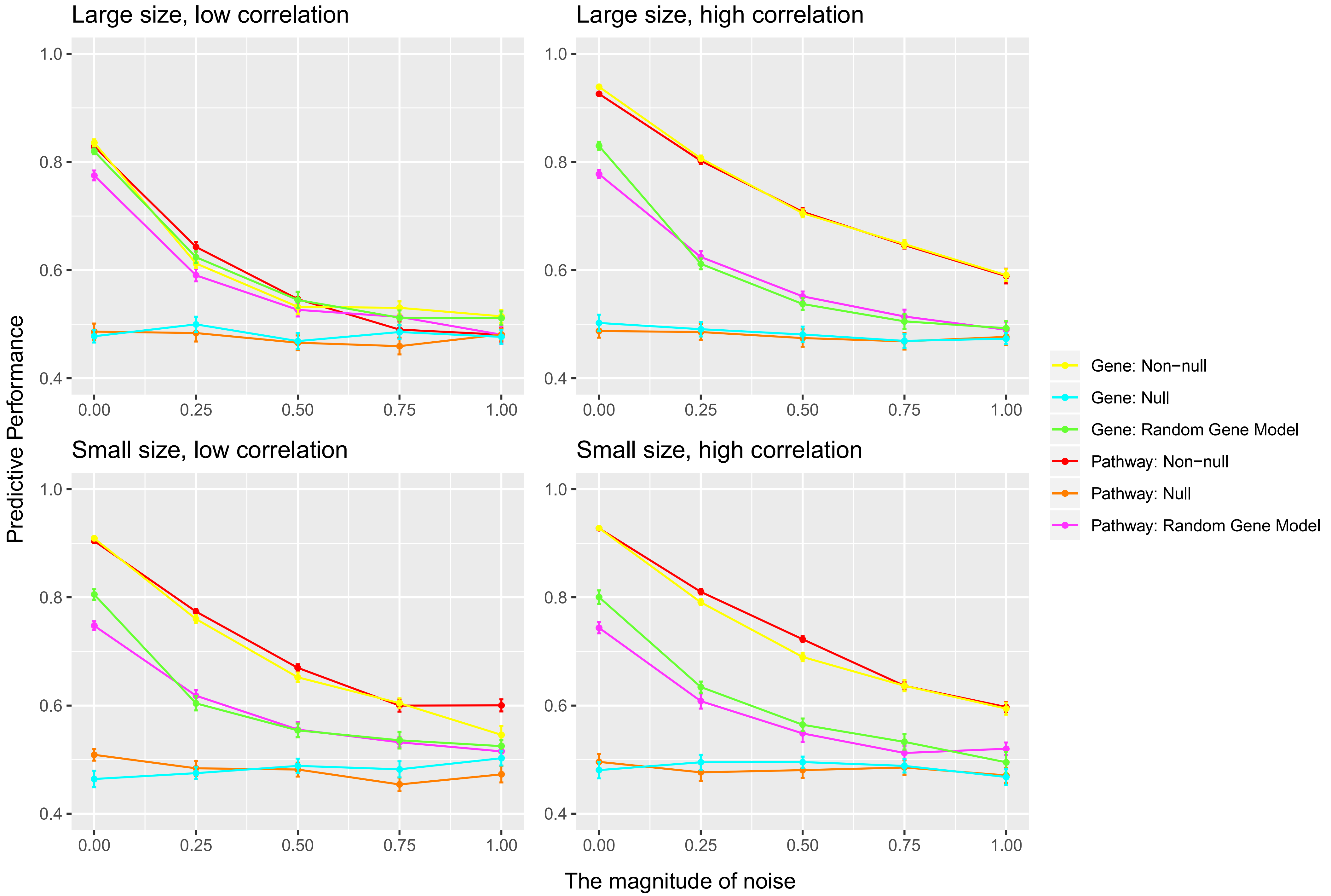

Figure S44: PAAD cohort, simulation 2

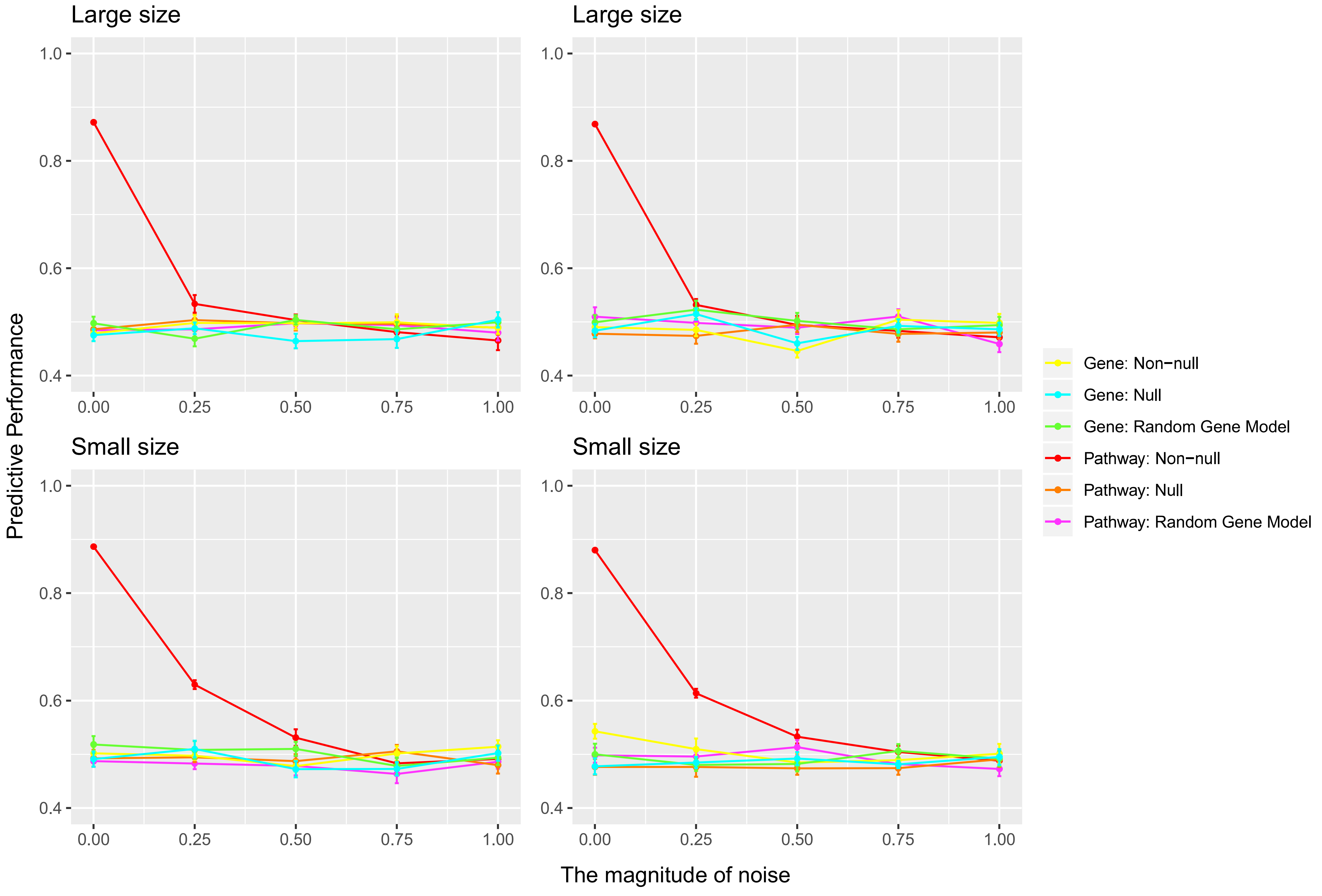

Figure S45: PCPG cohort, simulation 1

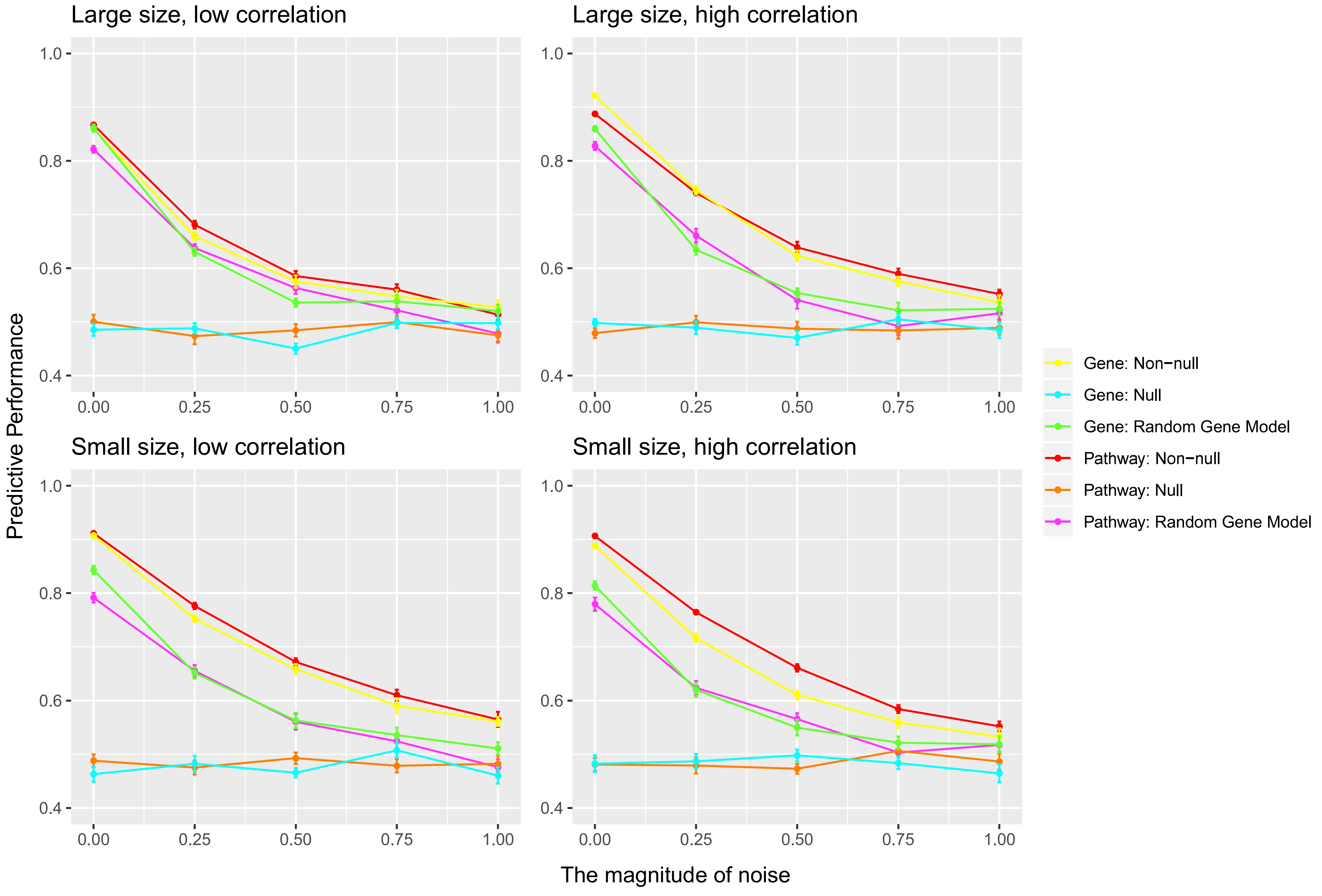

Figure S46: PCPG cohort, simulation 2

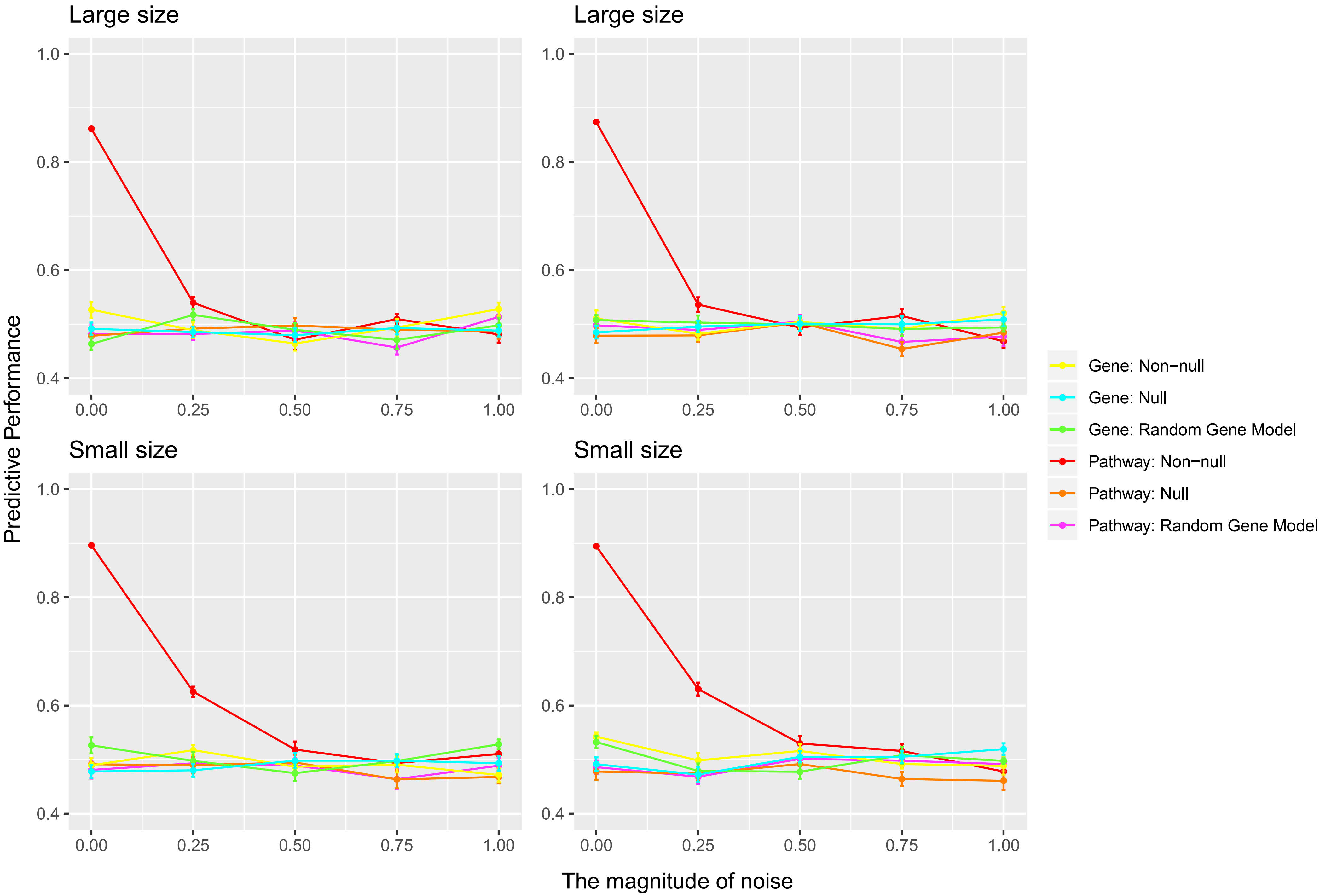

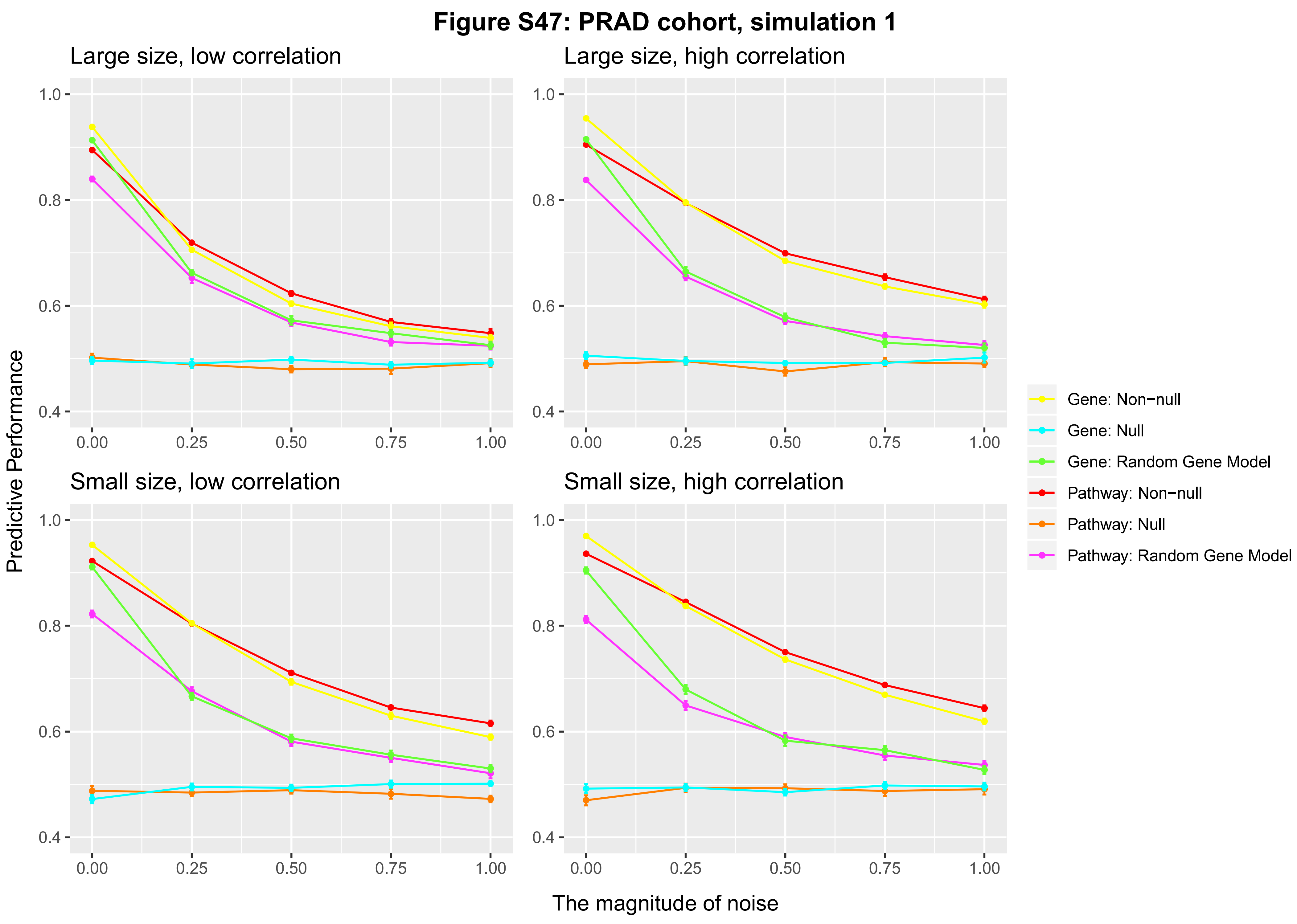

Figure S47: PRAD cohort, simulation 1
Large size, low correlation
Large size, high correlation
Small size, low correlation
Small size, high correlation
Predictive Performance
The magnitude of noise
1.0
0.8
0.6
0.4
0.00
0.25
0.50
0.75
1.00
Gene: Non−null
Gene: Null
Gene: Random Gene Model
Pathway: Non−null
Pathway: Null
Pathway: Random Gene Model

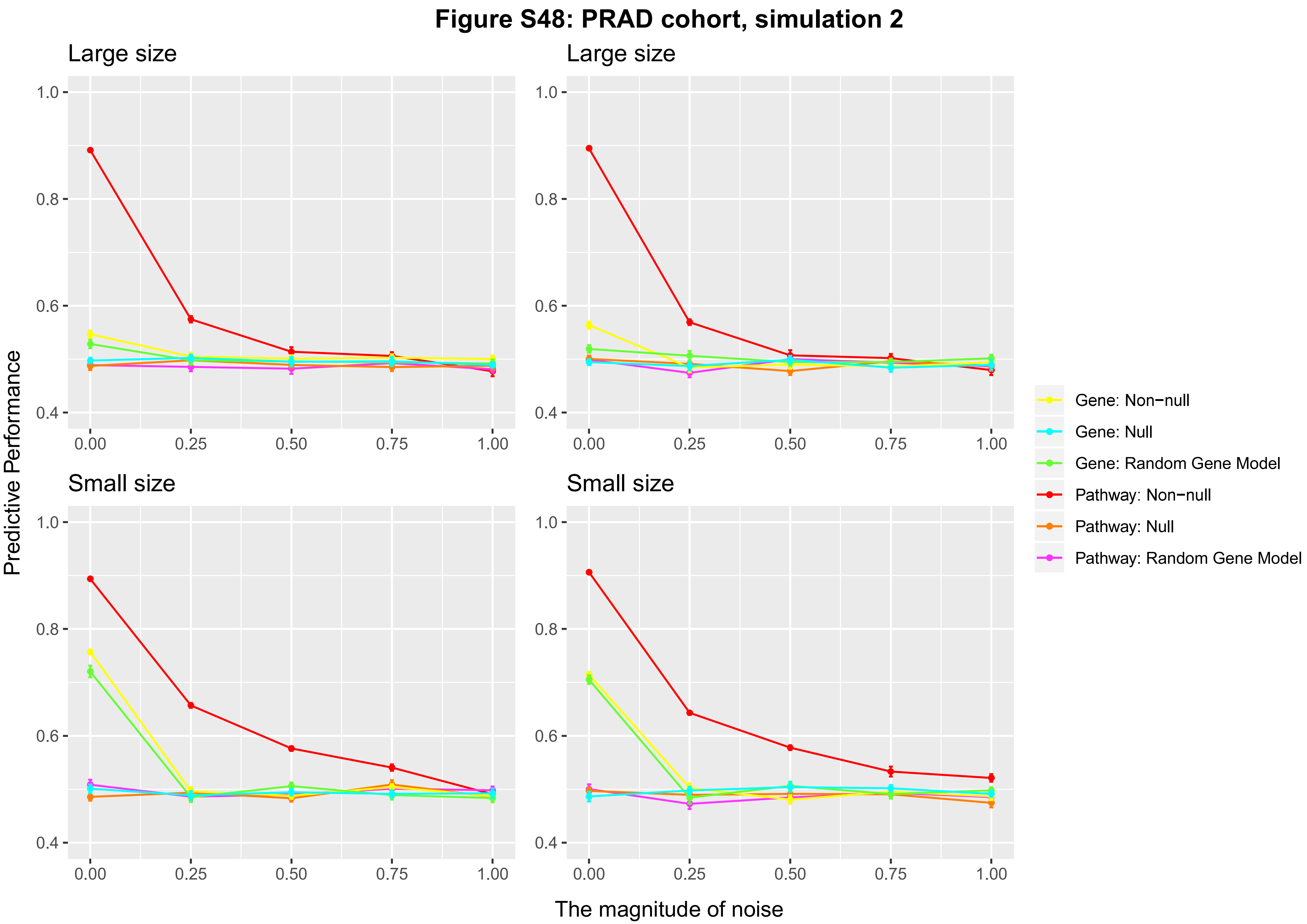
Figure S48: PRAD cohort, simulation 2
Large size
Large size
Small size
Small size
1.0
0.8
0.6
0.4
0.00
0.25
0.50
0.75
1.00
Predictive Performance
The magnitude of noise
Gene: Non−null
Gene: Null
Gene: Random Gene Model
Pathway: Non−null
Pathway: Null
Pathway: Random Gene Model

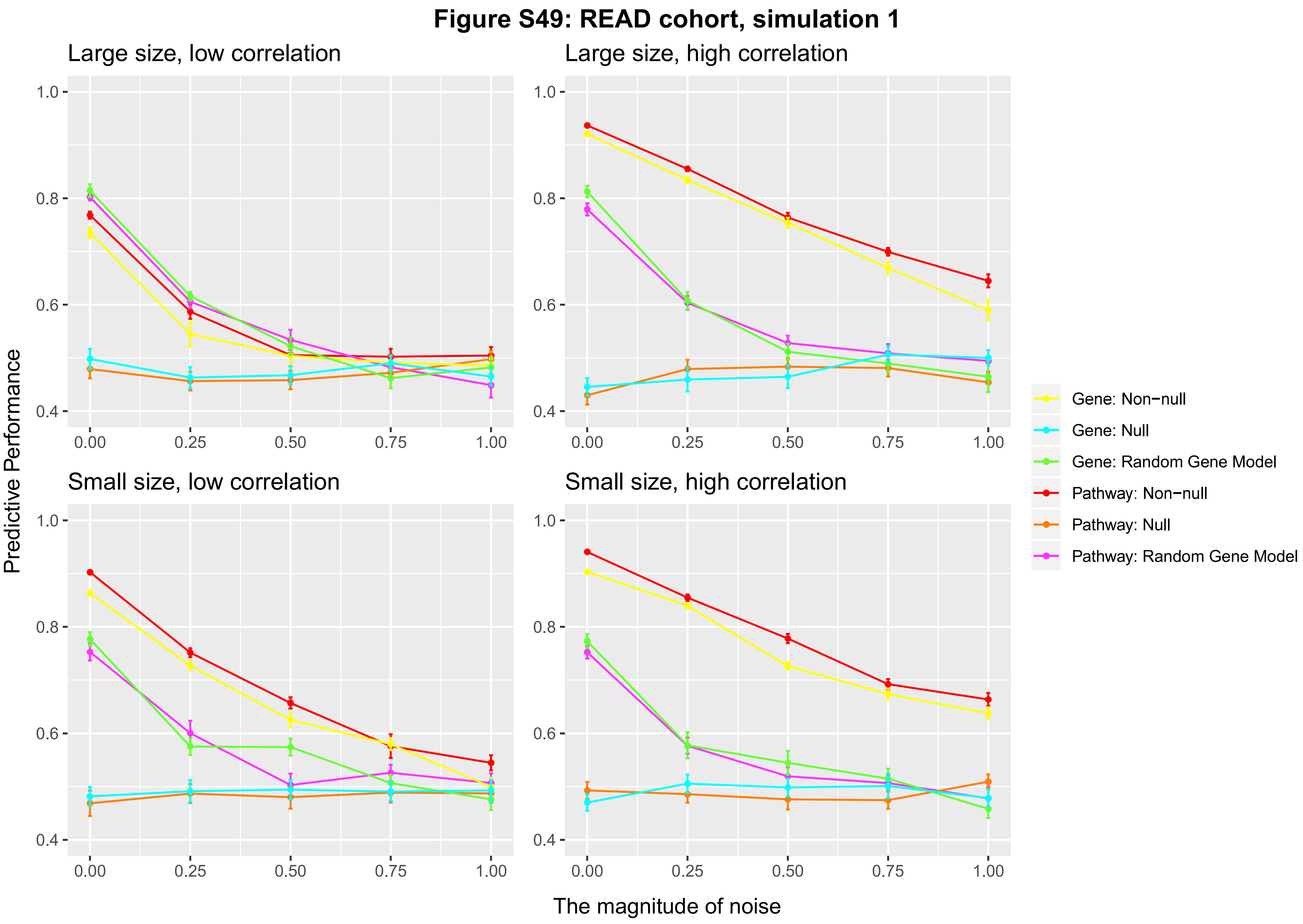
Figure S49: READ cohort, simulation 1
Large size, low correlation
Large size, high correlation
Small size, low correlation
Small size, high correlation
Predictive Performance
The magnitude of noise
1.0
0.8
0.6
0.4
0.00
0.25
0.50
0.75
1.00
Gene: Non−null
Gene: Null
Gene: Random Gene Model
Pathway: Non−null
Pathway: Null
Pathway: Random Gene Model

Figure S50: READ cohort, simulation 2

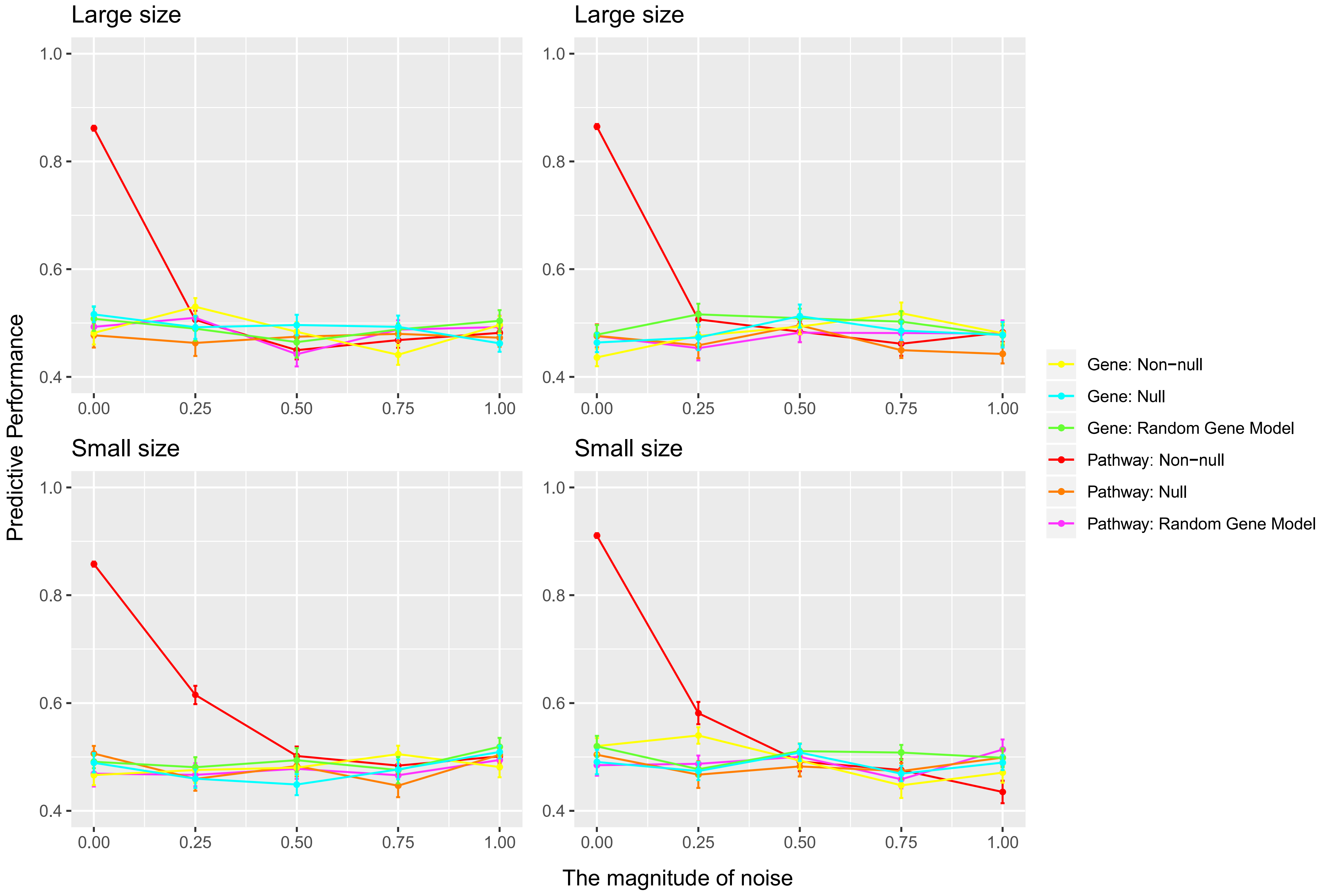

Figure S51: SARC cohort, simulation 1

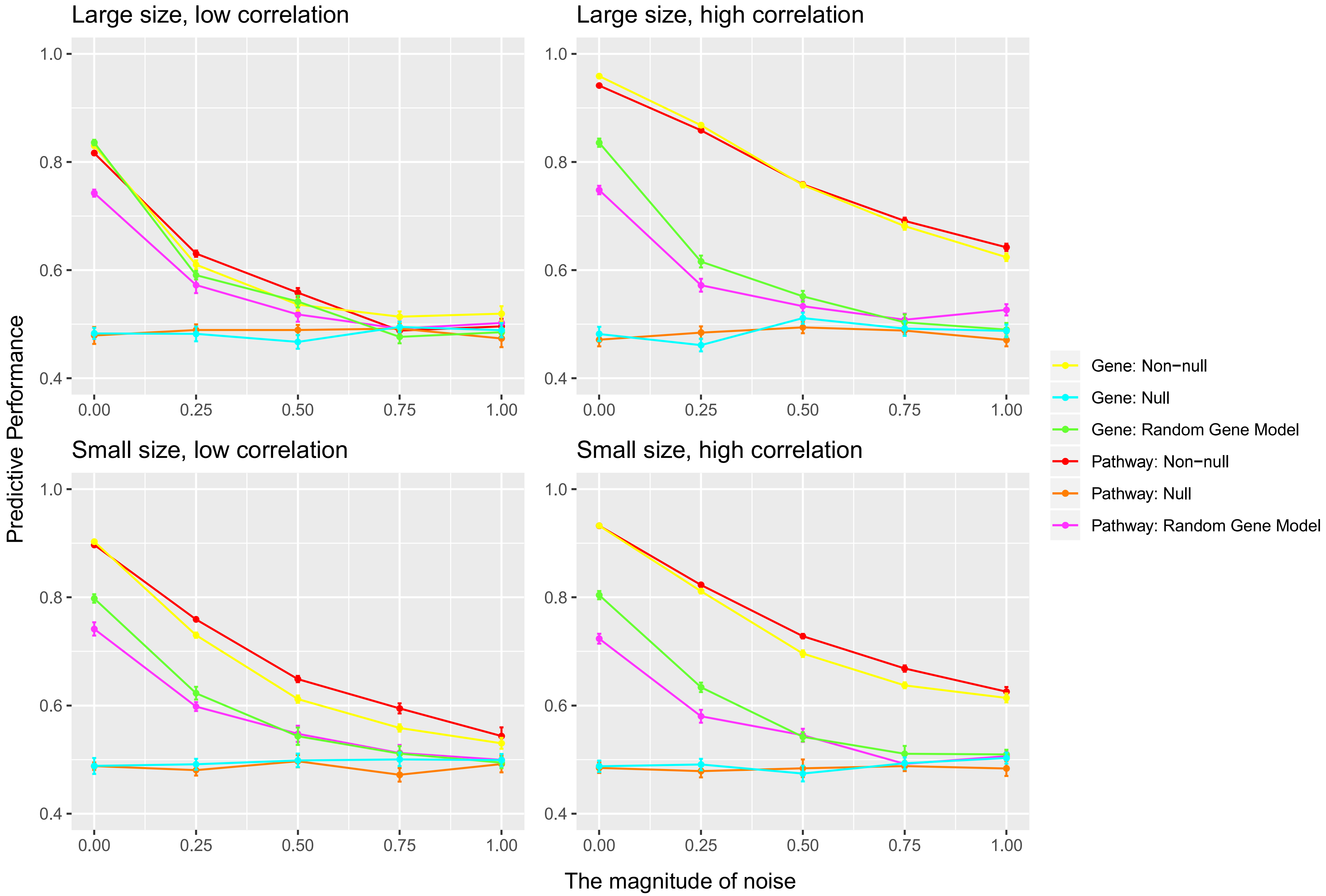

Figure S52: SARC cohort, simulation 2

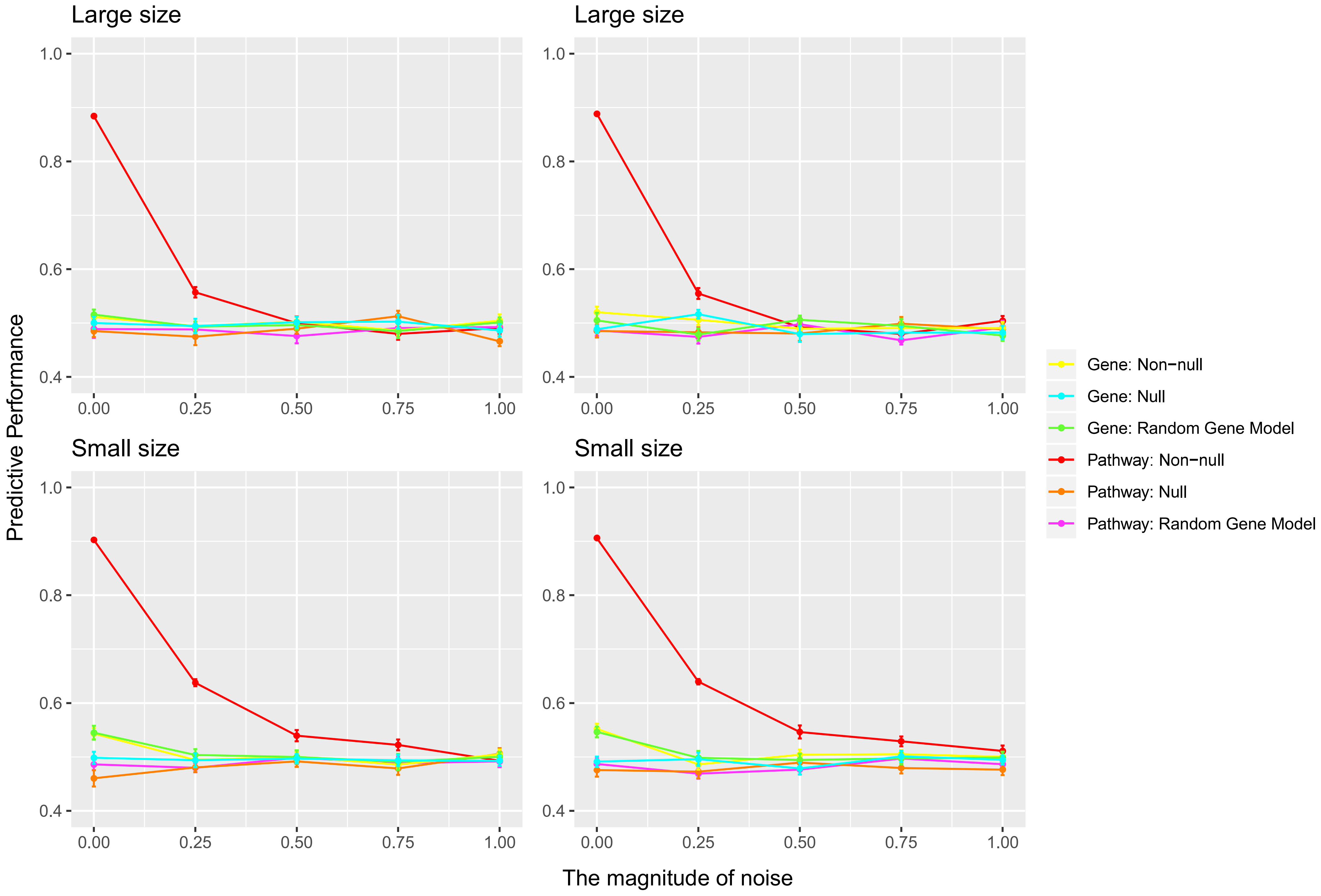

Figure S53: SKCM cohort, simulation 1

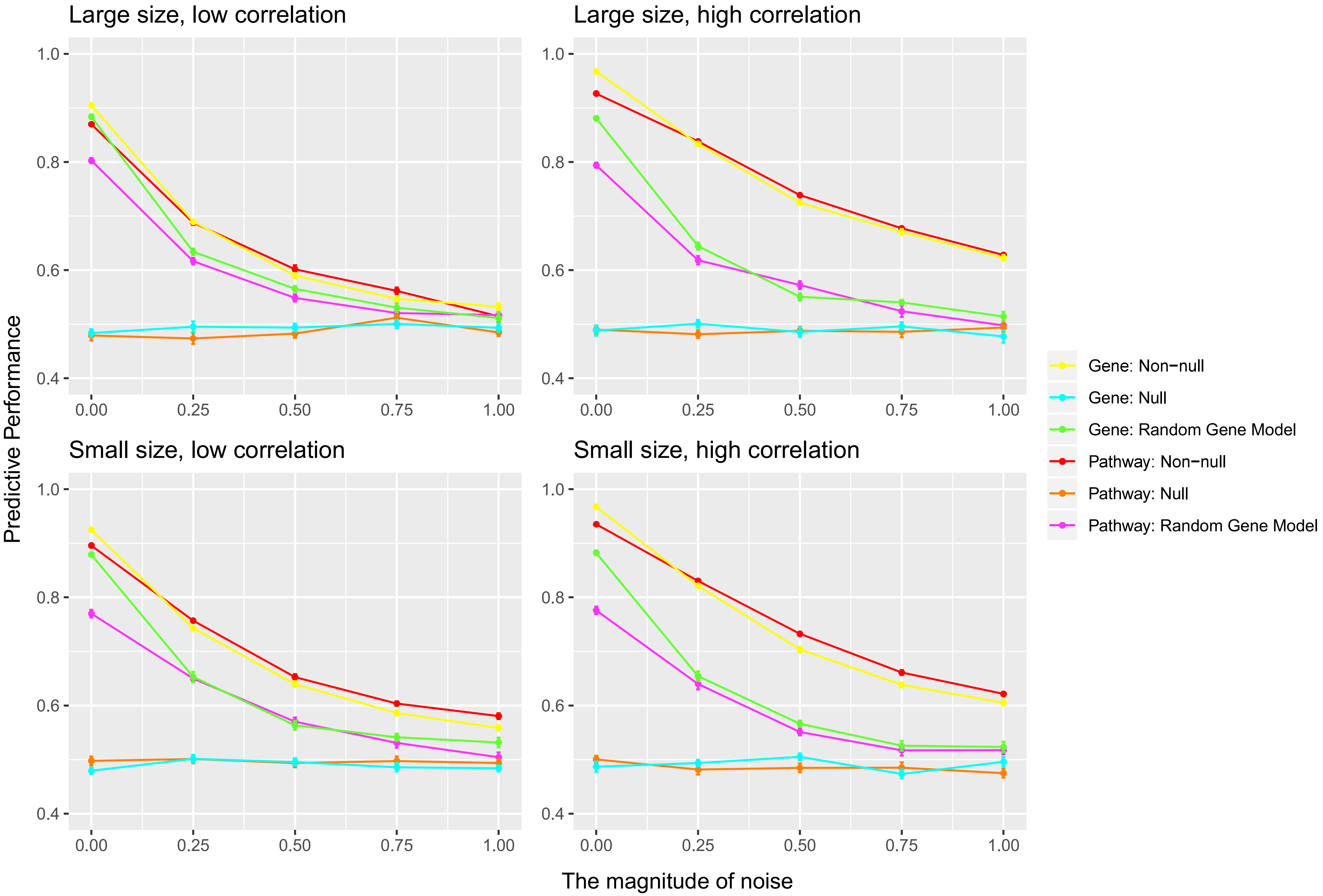

Figure S54: SKCM cohort, simulation 2

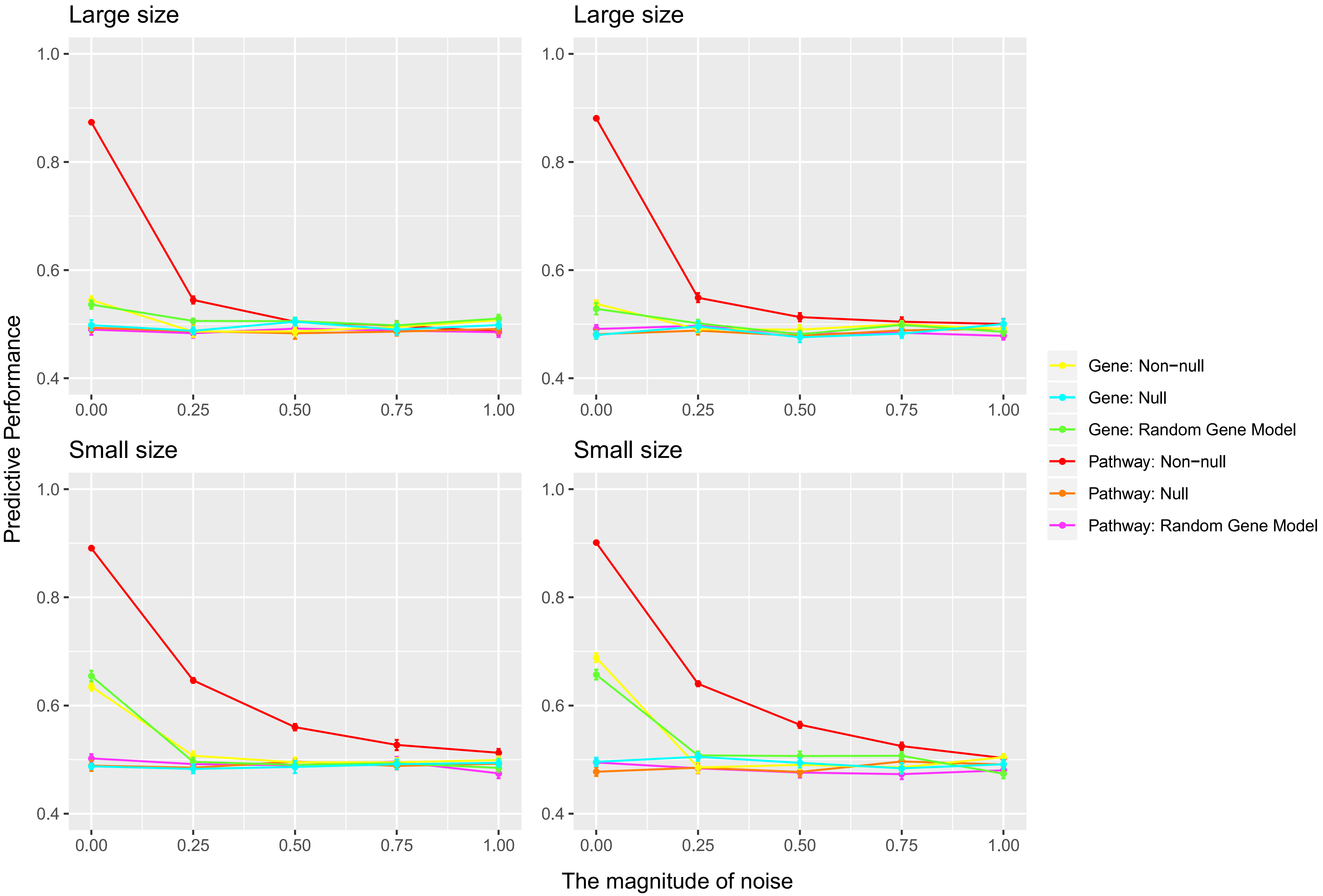

Figure S55: STAD cohort, simulation 1

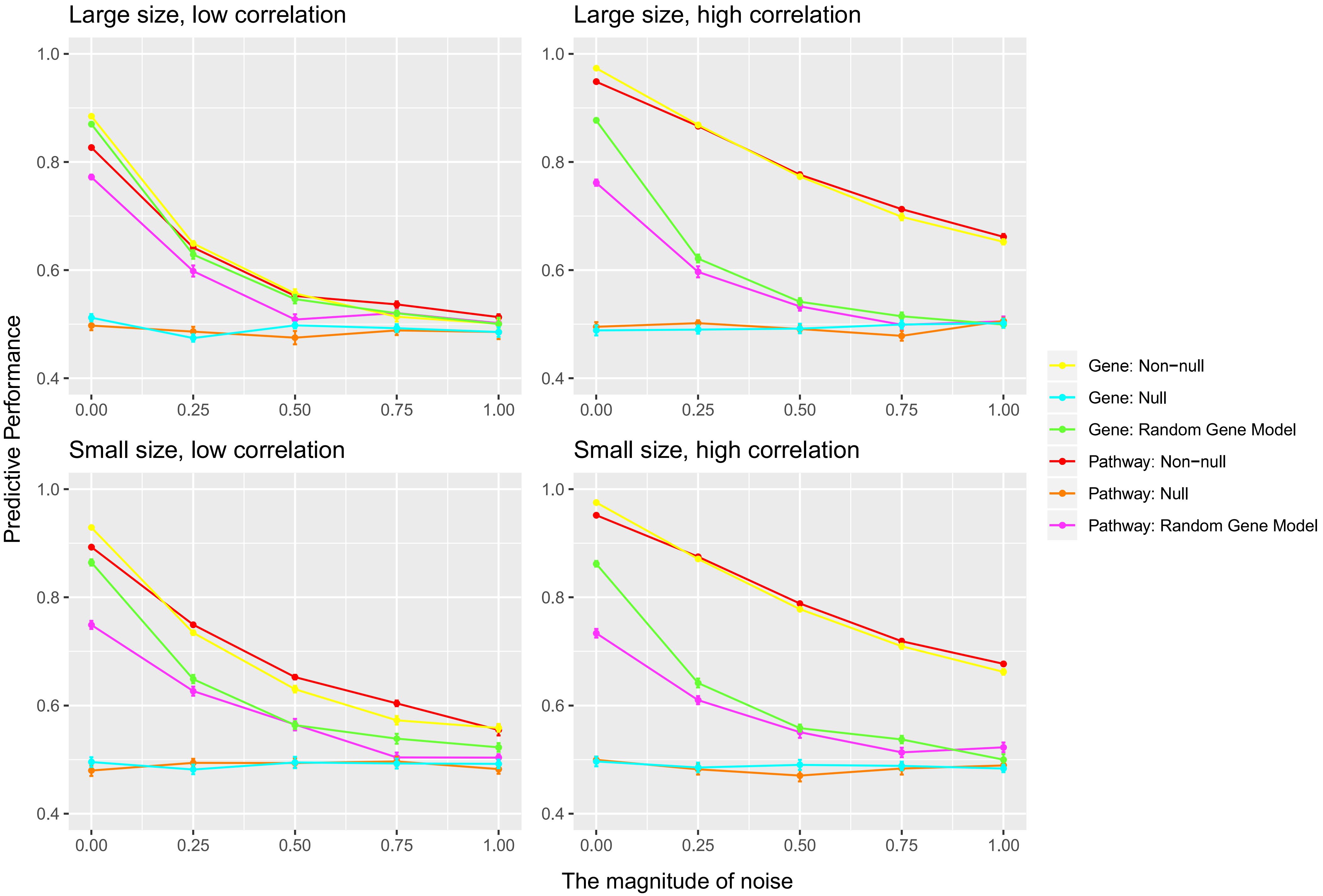

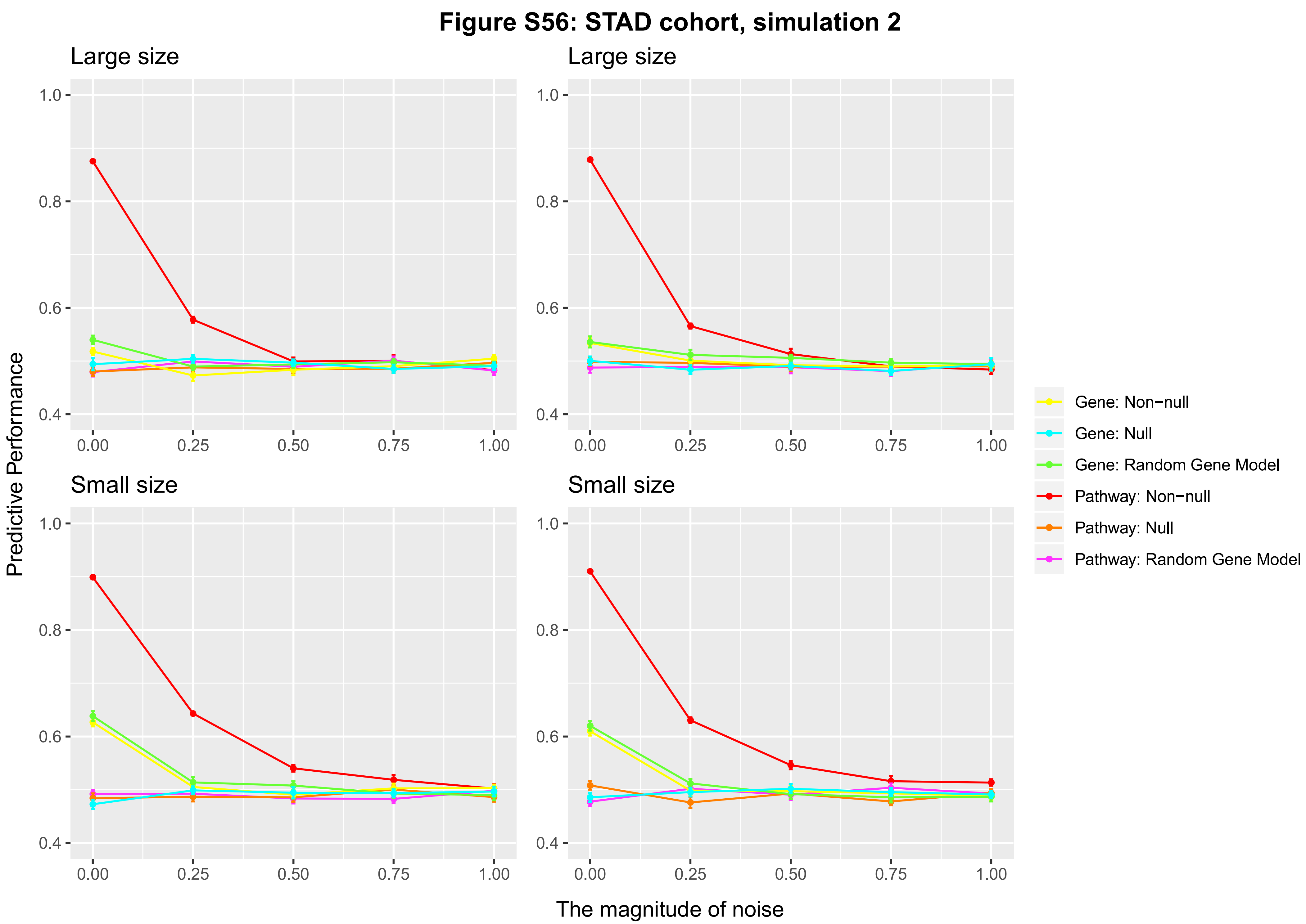
Figure S56: STAD cohort, simulation 2
Large size
Large size
Small size
Small size
Predictive Performance
The magnitude of noise
1.0
0.8
0.6
0.4
0.00
0.25
0.50
0.75
1.00
Gene: Non−null
Gene: Null
Gene: Random Gene Model
Pathway: Non−null
Pathway: Null
Pathway: Random Gene Model

Figure S57: TGCT cohort, simulation 1

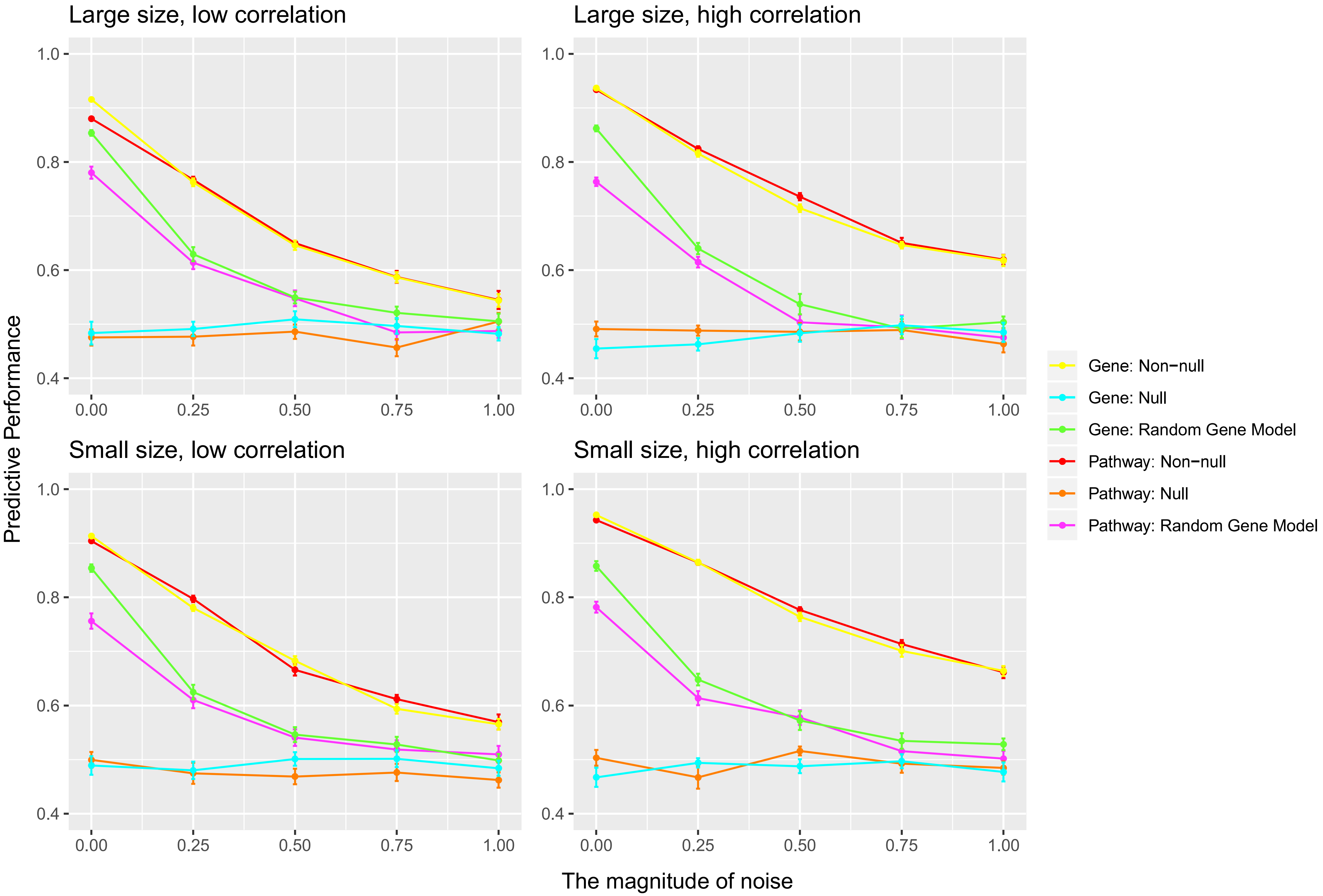

Figure S58: TGCT cohort, simulation 2

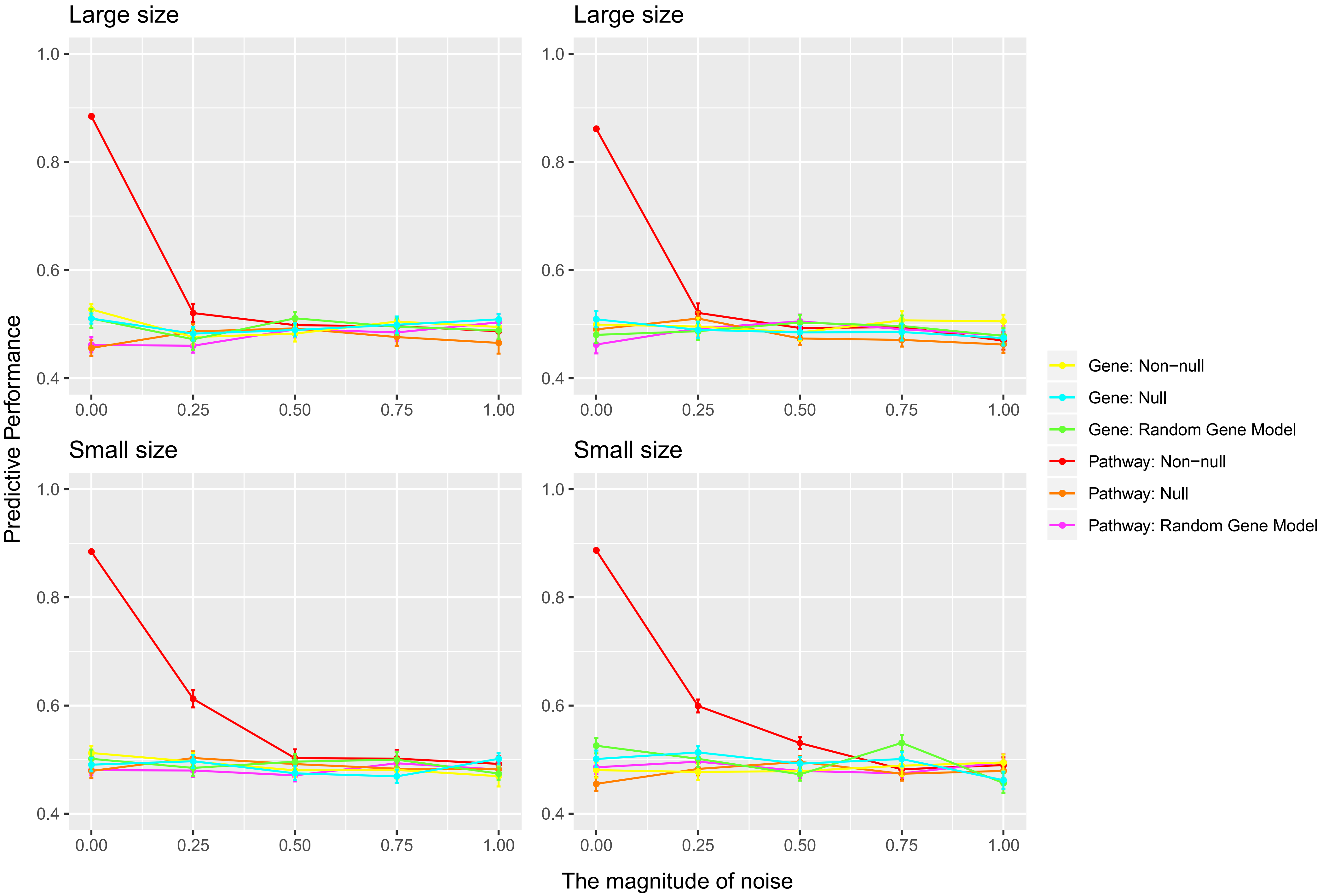

Figure S59: THCA cohort, simulation 1

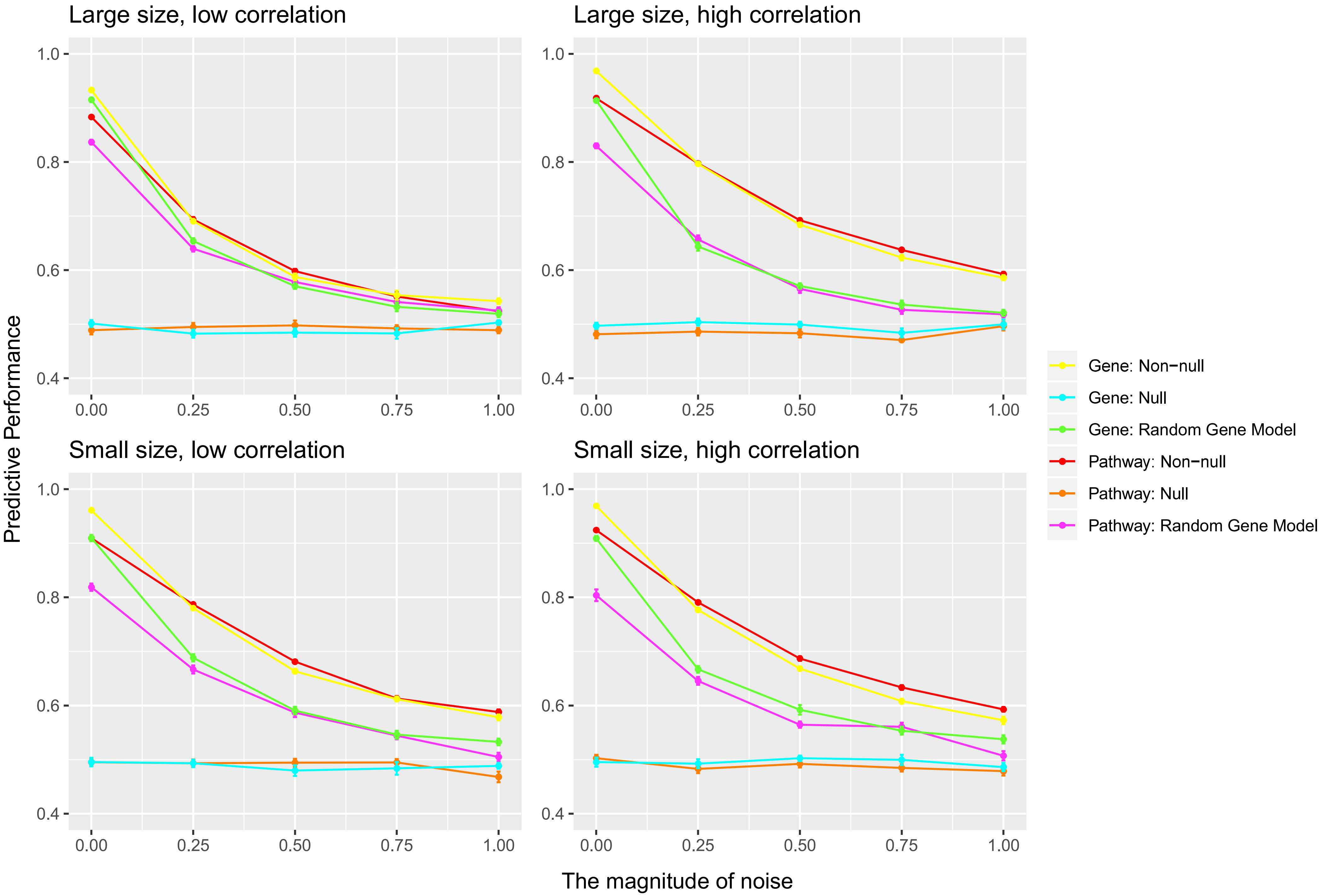

Figure S60: THCA cohort, simulation 2

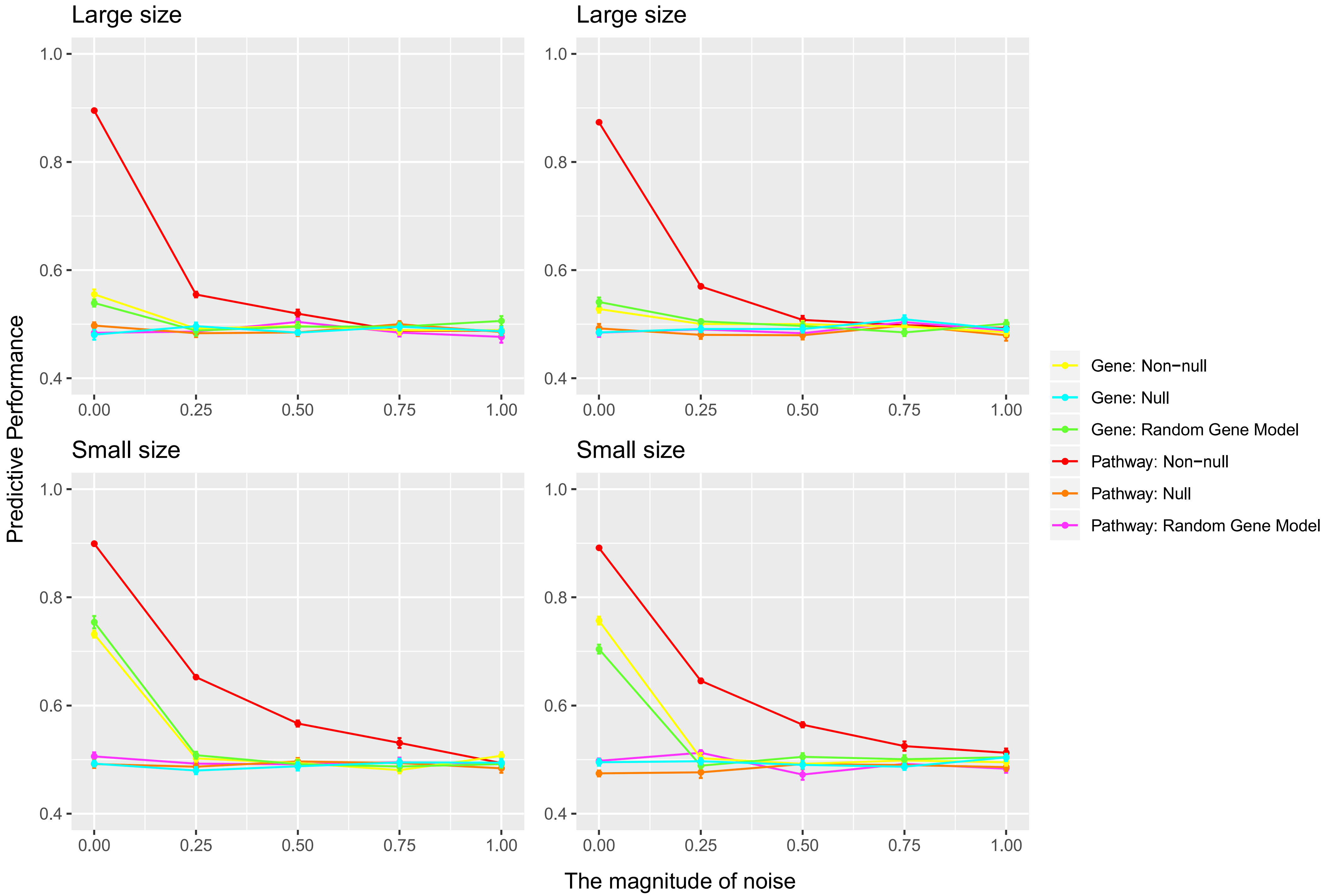

Figure S61: THYM cohort, simulation 1

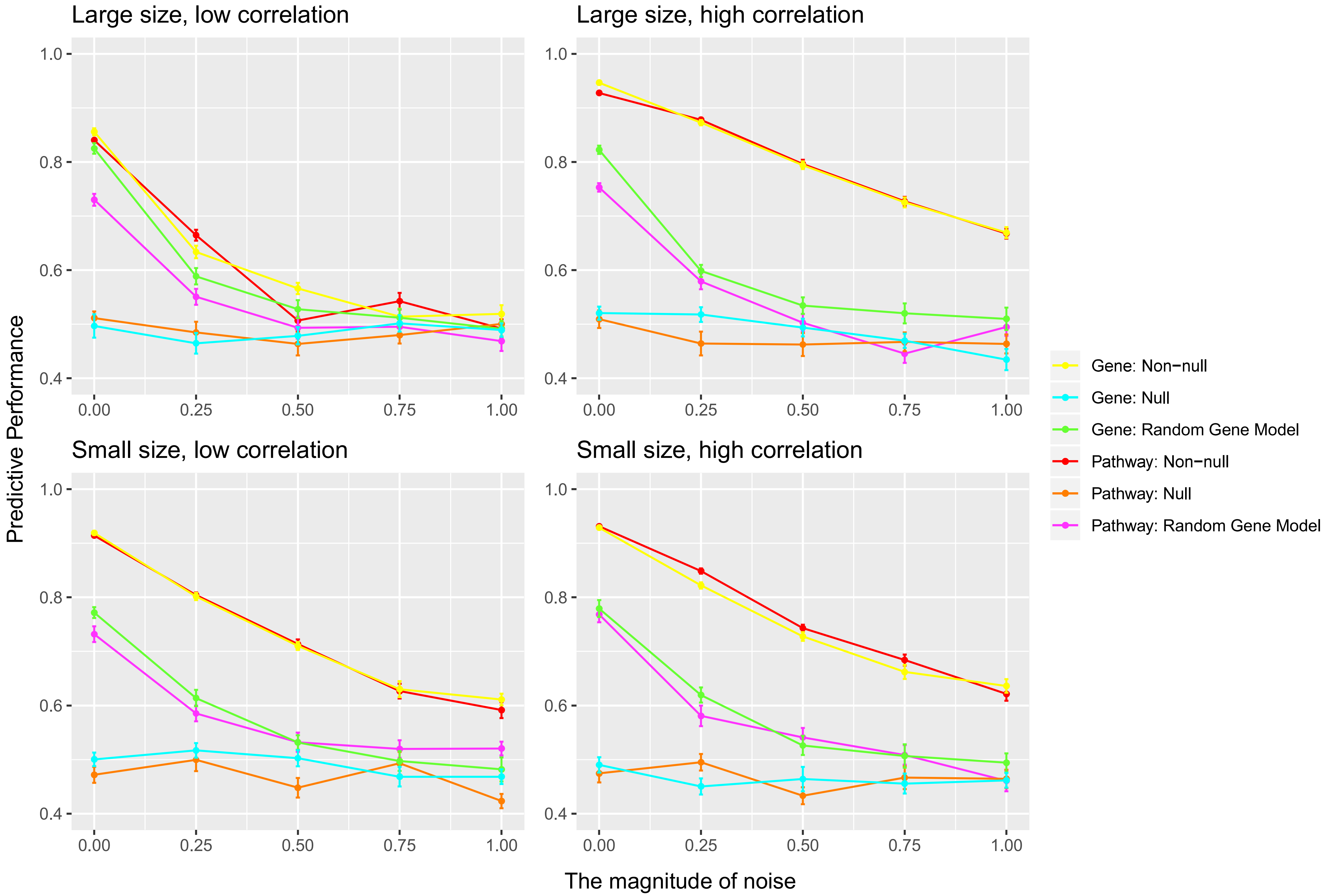

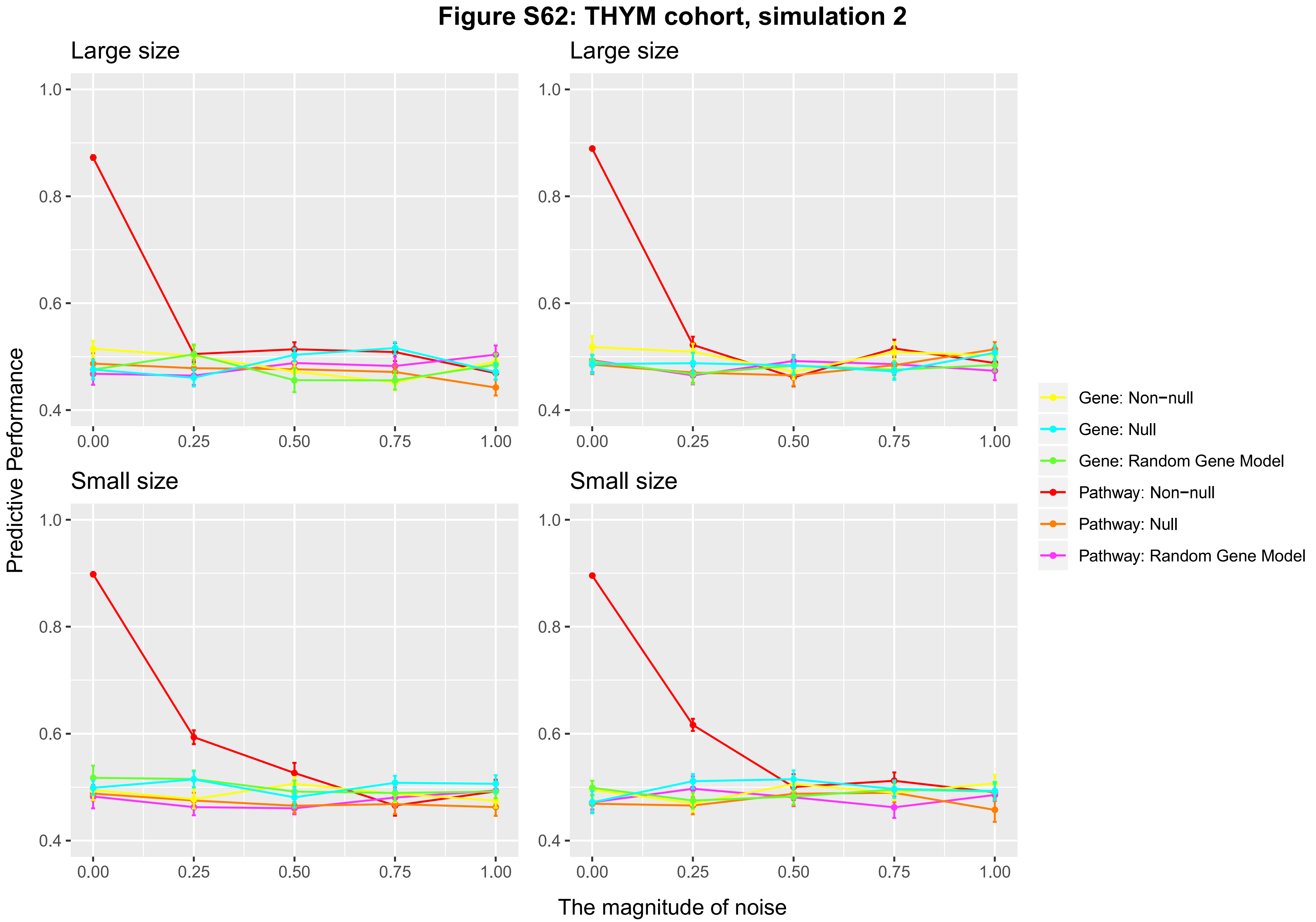

Figure S62: THYM cohort, simulation 2
Large size
Large size
Small size
Small size
Predictive Performance
The magnitude of noise
1.0
0.8
0.6
0.4
0.00
0.25
0.50
0.75
1.00
Gene: Non−null
Gene: Null
Gene: Random Gene Model
Pathway: Non−null
Pathway: Null
Pathway: Random Gene Model

Figure S63: UCEC cohort, simulation 1

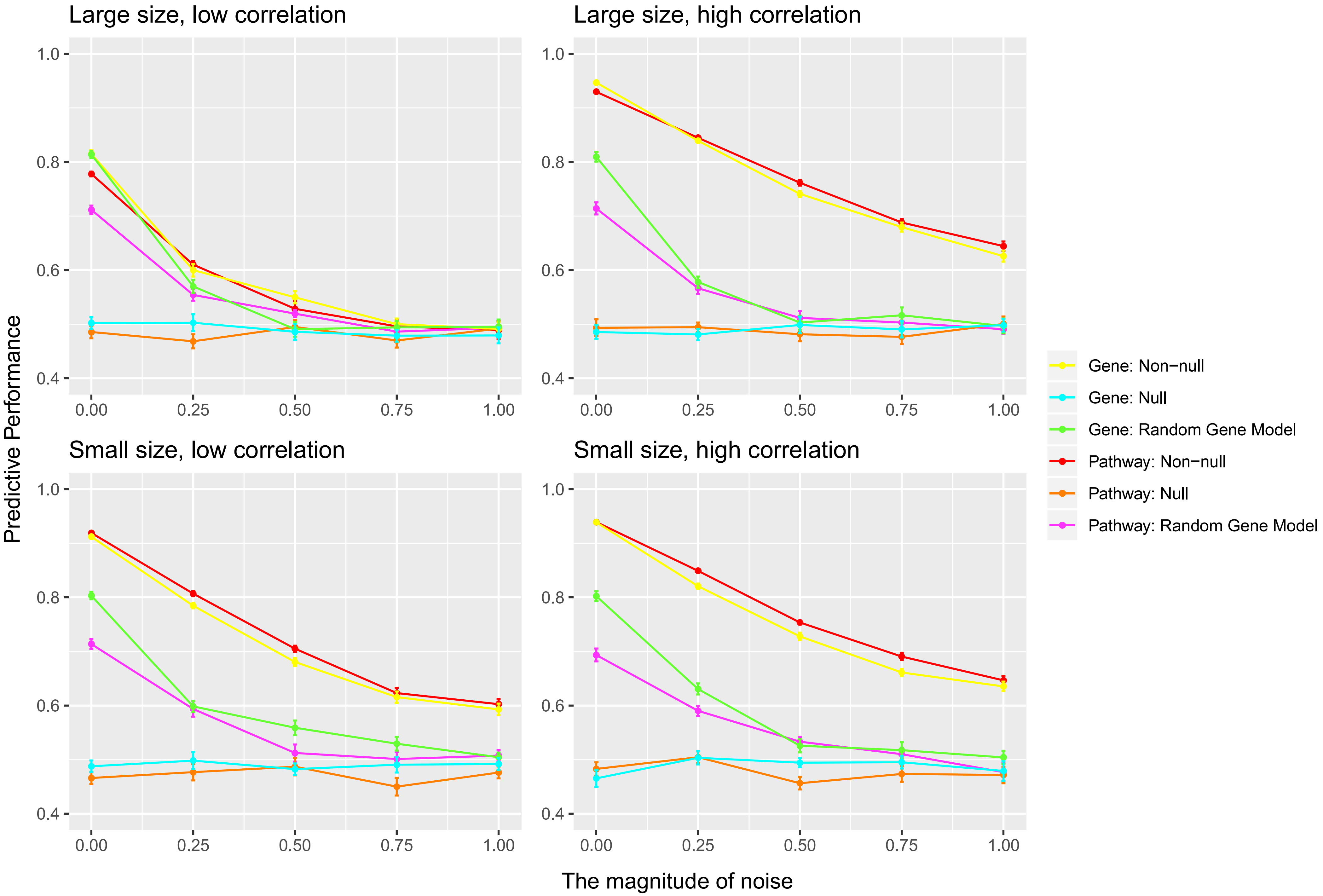

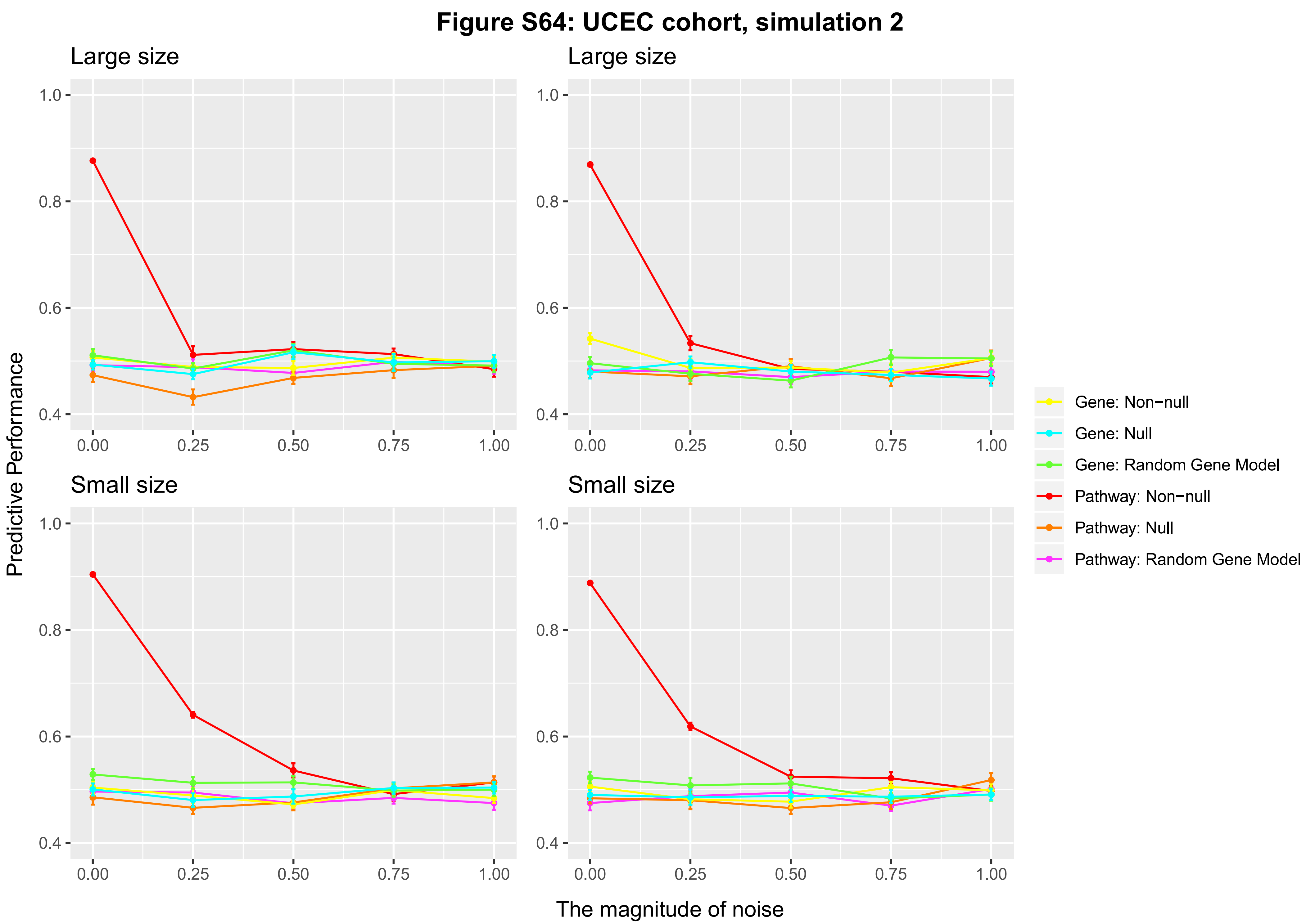

Figure S64: UCEC cohort, simulation 2
Large size
Large size
Small size
Small size
Predictive Performance
The magnitude of noise
1.0
0.8
0.6
0.4
0.00
0.25
0.50
0.75
1.00
Gene: Non−null
Gene: Null
Gene: Random Gene Model
Pathway: Non−null
Pathway: Null
Pathway: Random Gene Model

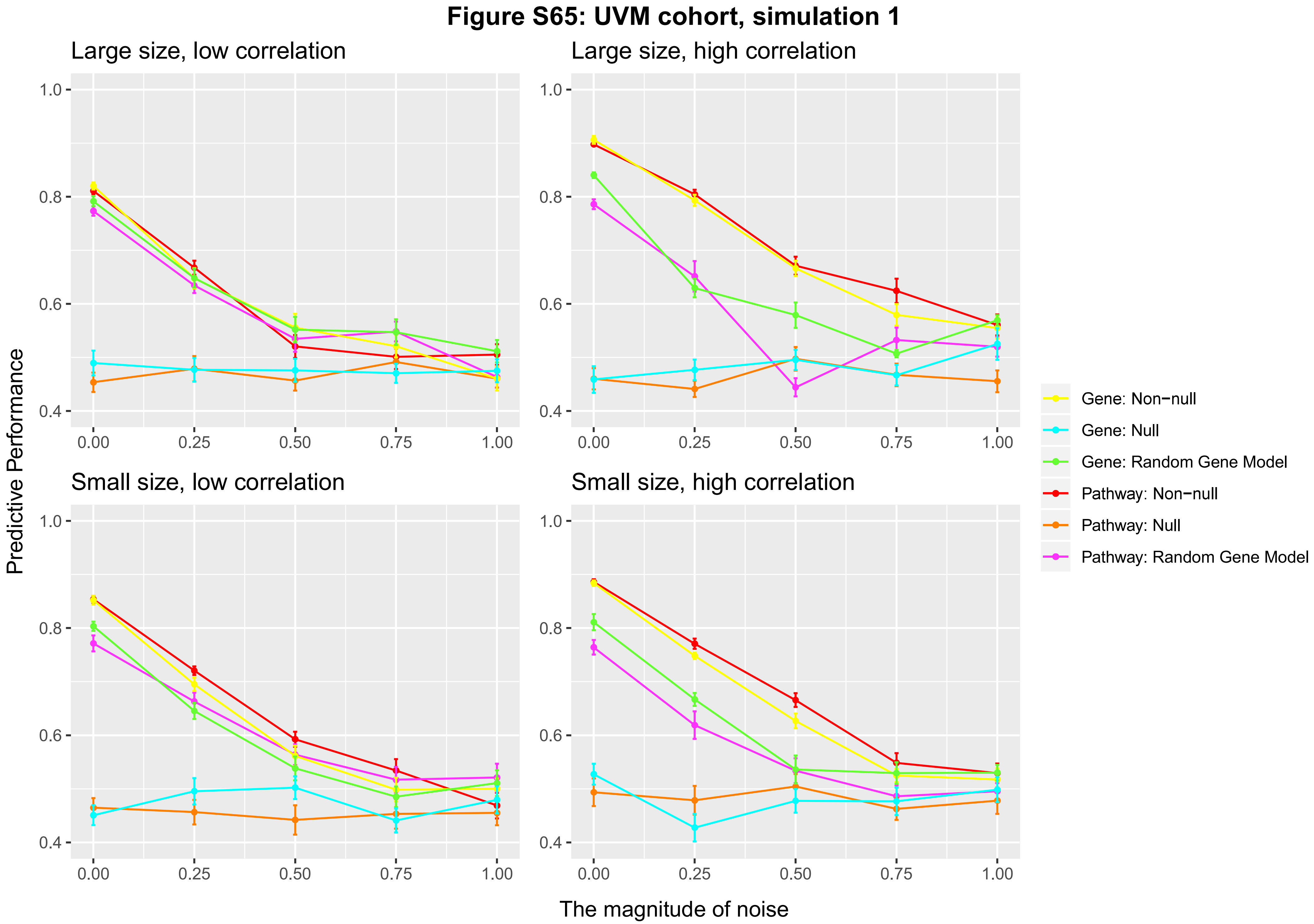

Figure S65: UVM cohort, simulation 1
Large size, low correlation
Large size, high correlation
Small size, low correlation
Small size, high correlation
Predictive Performance
The magnitude of noise
1.0
0.8
0.6
0.4
0.00
0.25
0.50
0.75
1.00
Gene: Non−null
Gene: Null
Gene: Random Gene Model
Pathway: Non−null
Pathway: Null
Pathway: Random Gene Model

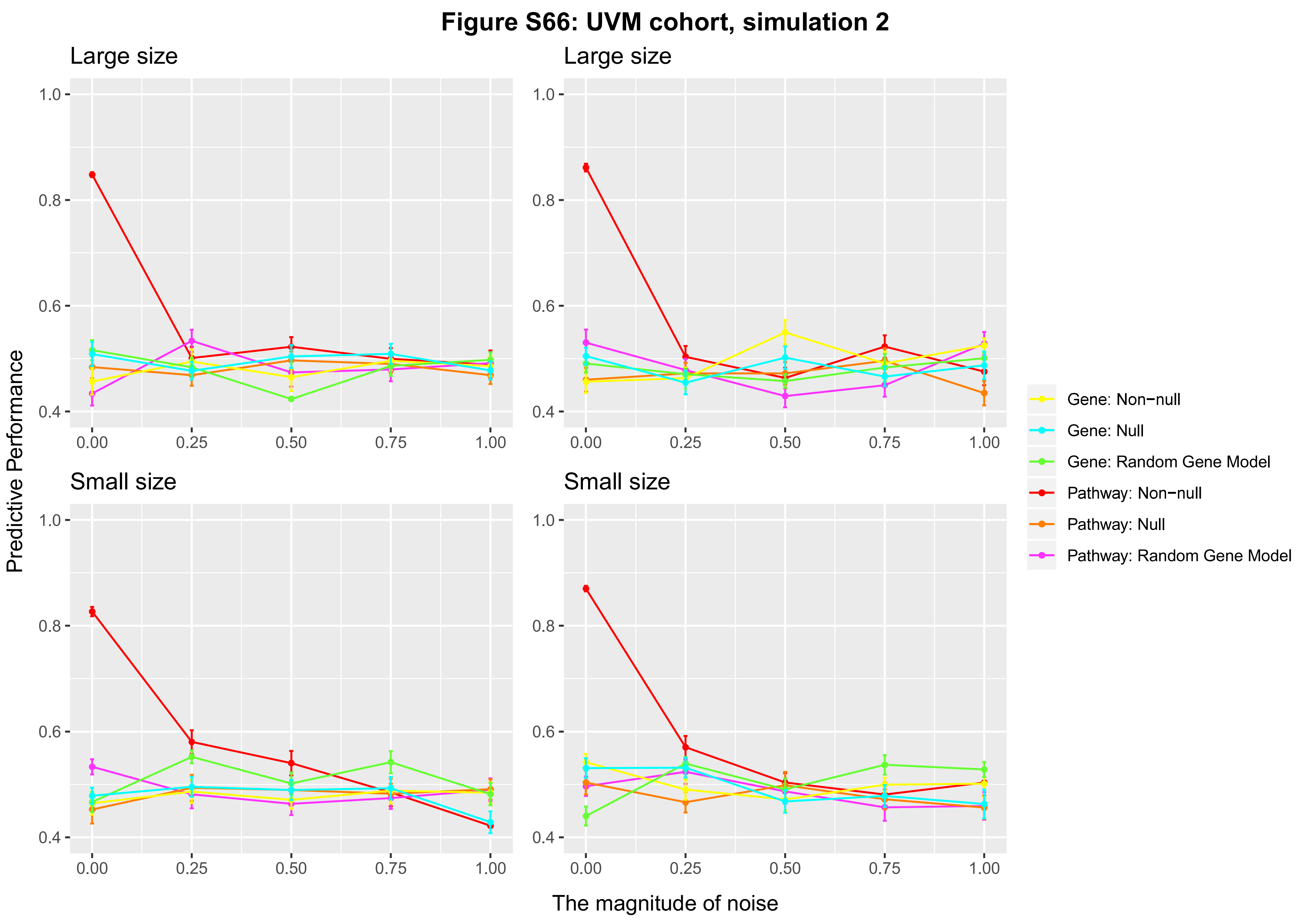
Figure S66: UVM cohort, simulation 2
Large size
Large size
Small size
Small size
Predictive Performance
The magnitude of noise
1.0
0.8
0.6
0.4
0.00
0.25
0.50
0.75
1.00
Gene: Non−null
Gene: Null
Gene: Random Gene Model
Pathway: Non−null
Pathway: Null
Pathway: Random Gene Model
