# Supplementary material for: Comparison of pathway and gene-level models for cancer prognosis prediction
Source: BMC Bioinformatics. 2020 Feb 28;21:76. doi: 10.1186/s12859-020-3423-z (PMC7048092; doi:10.1186/s12859-020-3423-z)

Supplementary Material: Comparison of pathway and gene-level models for cancer prognosis prediction

Xingyu Zheng, Christopher I. Amos, H. Robert Frost

Supplementary results of associating different pathways in the simulation studies for LGG cohort:

To investigate if the choice of pathway used to simulate survival times impacts model performance, we also tested the models on survival times generated using each of the 50 pathways in the MSigDB Hallmark collection for LGG cohort. Figure S67 to S116 are displayed below and two plots corresponding to two simulation studies for each pathway are displayed in one figure. Since they share the same figure captions, we display the captions here.

Figures of simulation 1 have this figure caption:

Results of the simulation study based on gene expression data from the LGG cohort and the representative pathway from the MSigDB Hallmark collection. Each panel plots the predictive performance of the evaluated gene-level and pathway-level models for simulation studies that associated survival with this Hallmark pathway. The Cox concordance index is plotted on the y-axis with the x-axis representing the standard deviation of the Gaussian noise added to the simulated survival times. The error bars represent the standard error over 20 replications.

Figures of simulation 2 have this figure caption:

Results of the simulation study based on gene expression data from the TCGA LGG cohort without inter-gene correlation and the representative pathway from the MSigDB Hallmark collection. The correlation in the gene expression data has been broken by randomly permuting the values for each gene. Each panel plots the predictive performance of the evaluated gene-level and pathway-level models for simulation studies that associated survival with this Hallmark pathway. The Cox concordance index is plotted on the y-axis with the x-axis representing the standard deviation of the Gaussian noise added to the simulated survival times. The error bars represent the standard error over 20 replications.

Figure S67: No.1 HALLMARK_TNFA_SIGNALING_VIA_NFKB (size=200, absolute mean correlation=0.25)

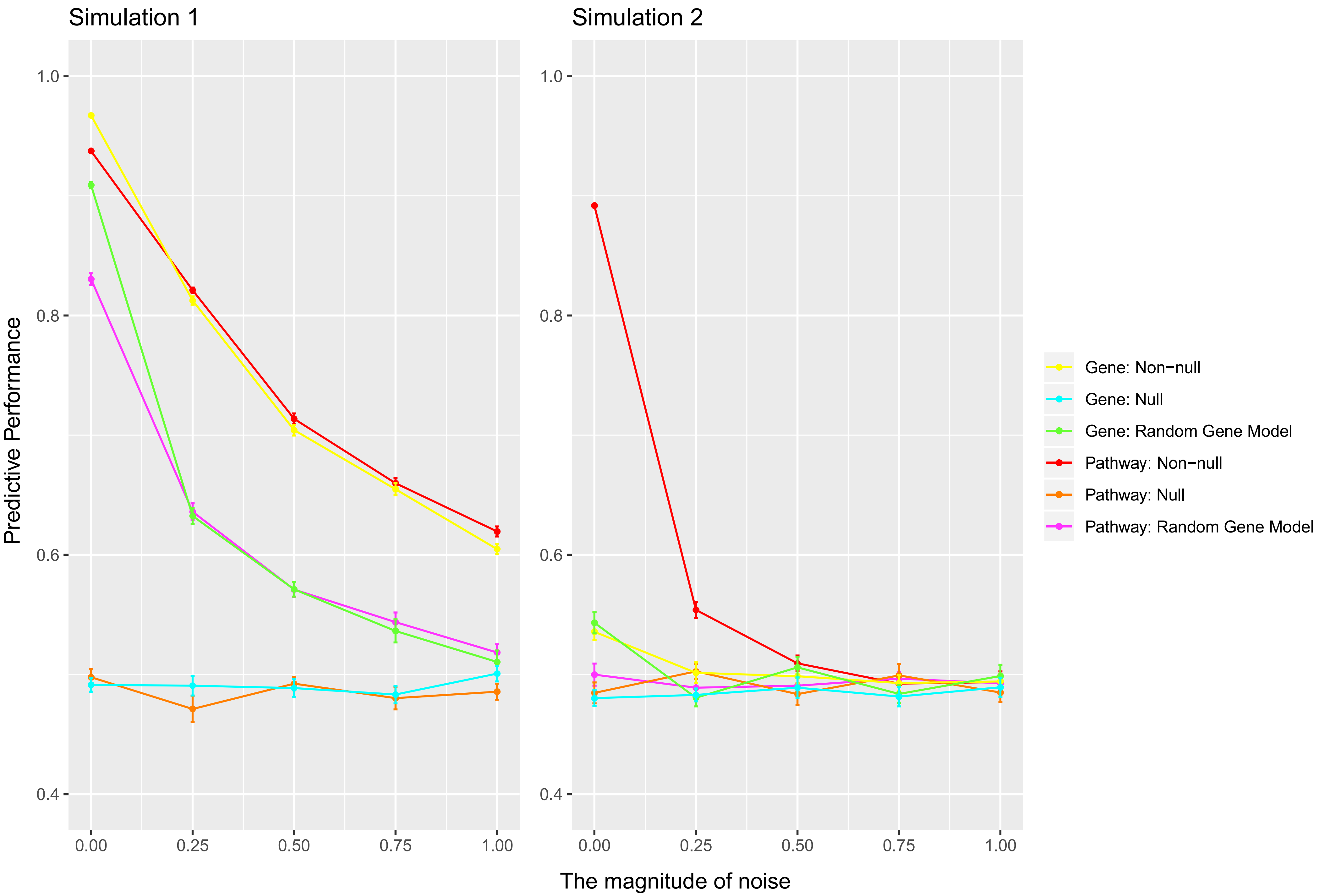

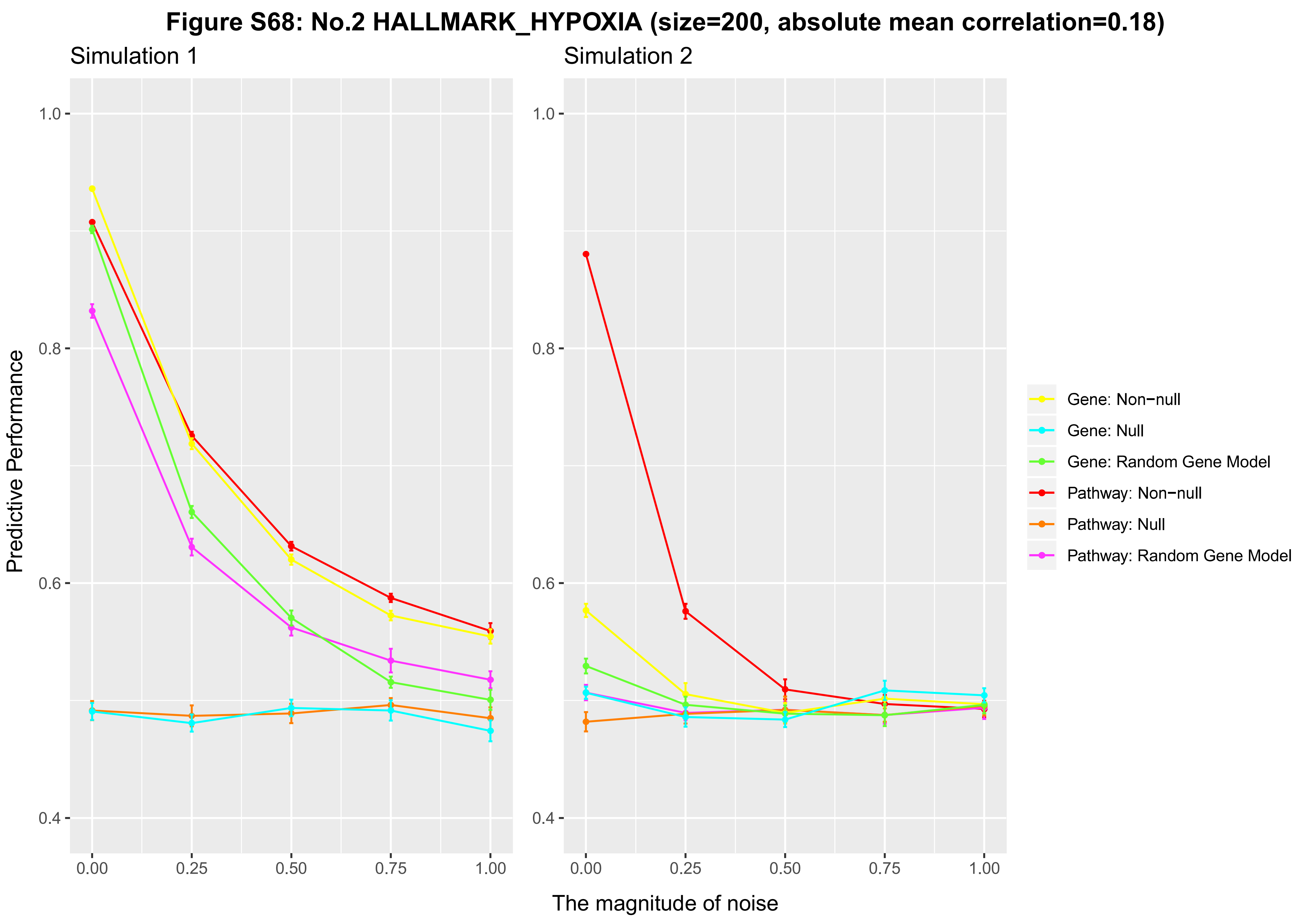

Figure S68: No.2 HALLMARK_HYPOXIA (size=200, absolute mean correlation=0.18)
Simulation 1
Simulation 2
Predictive Performance
The magnitude of noise
1.0
0.8
0.6
0.4
0.00
0.25
0.50
0.75
1.00
Gene: Non−null
Gene: Null
Gene: Random Gene Model
Pathway: Non−null
Pathway: Null
Pathway: Random Gene Model

Figure S69: No.3 HALLMARK_CHOLESTEROL_HOMEOSTASIS (size=74, absolute mean correlation=0.25)

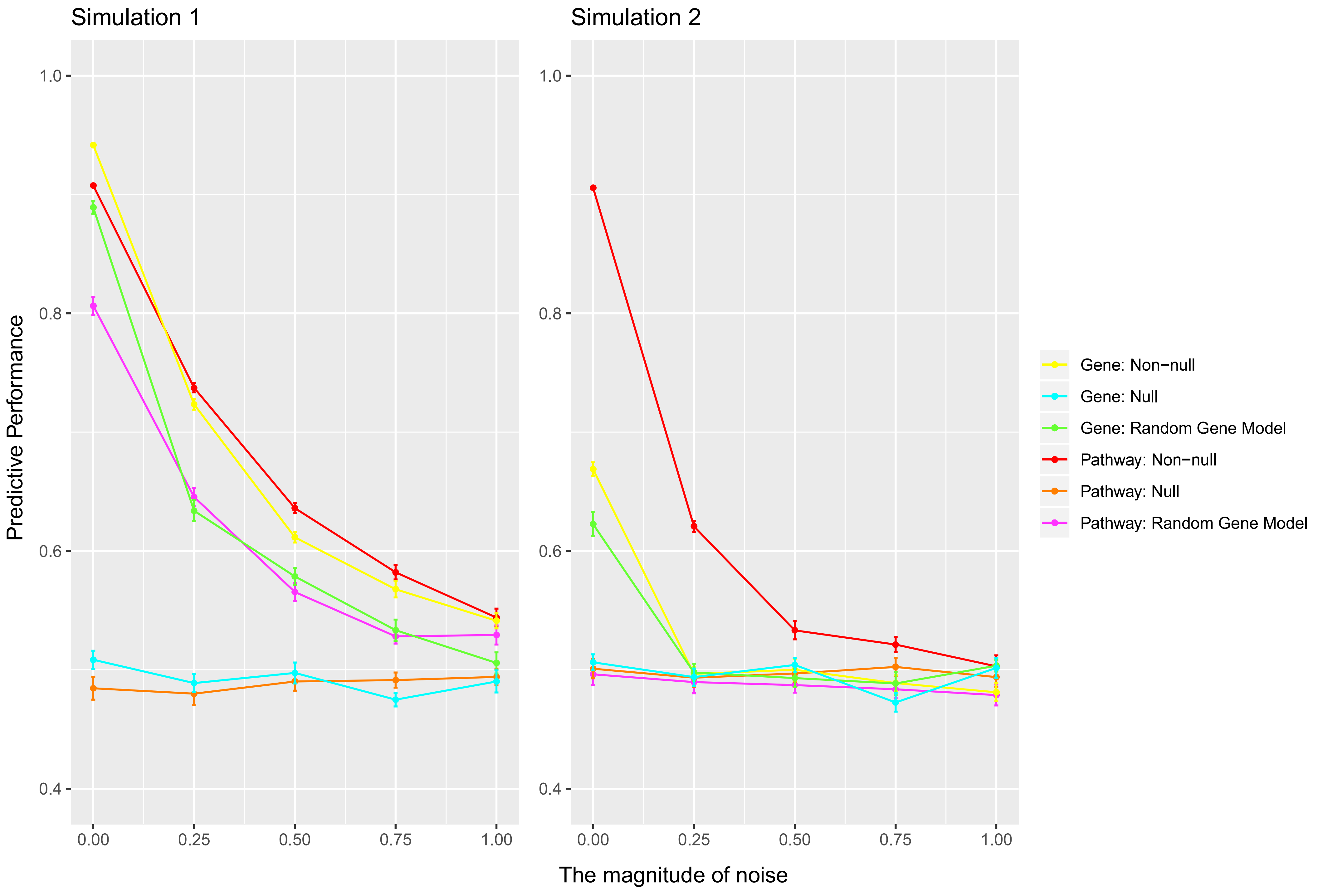

Figure S70: No.4 HALLMARK_MITOTIC_SPINDLE (size=200, absolute mean correlation=0.23)

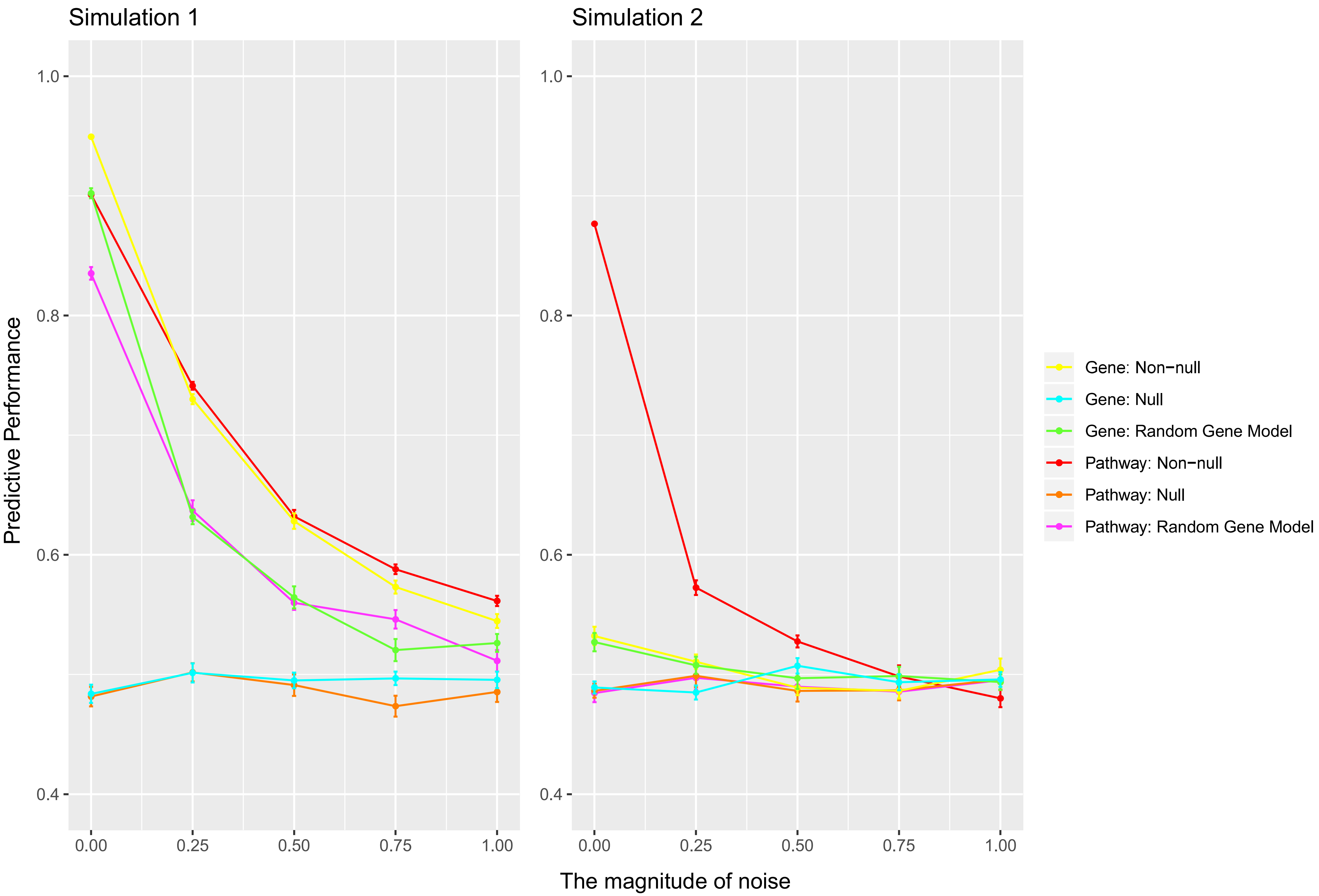

Figure S71: No.5 HALLMARK_WNT_BETA_CATENIN_SIGNALING (size=42, absolute mean correlation=0.22

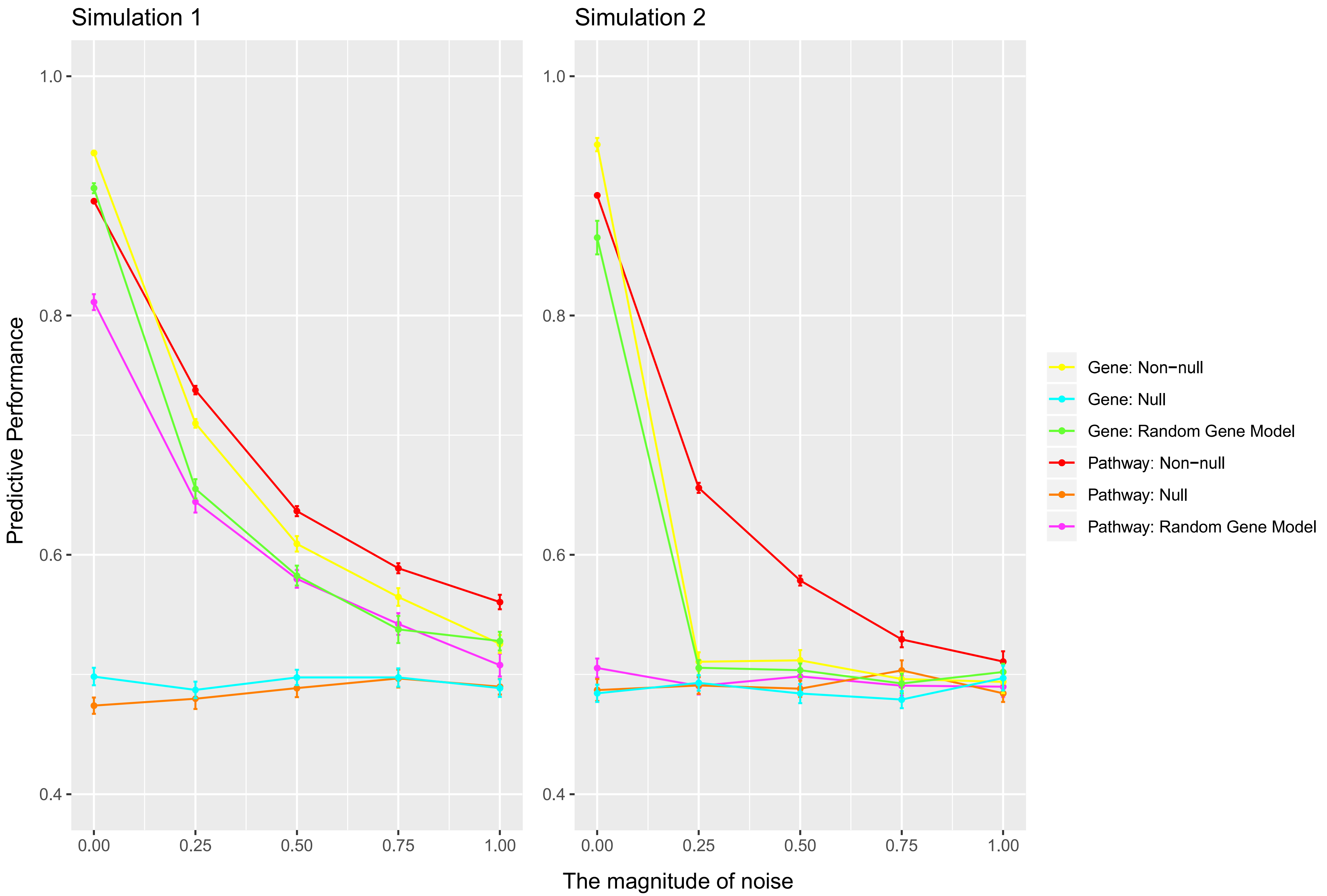

Figure S72: No.6 HALLMARK_TGF_BETA_SIGNALING (size=54, absolute mean correlation=0.22)

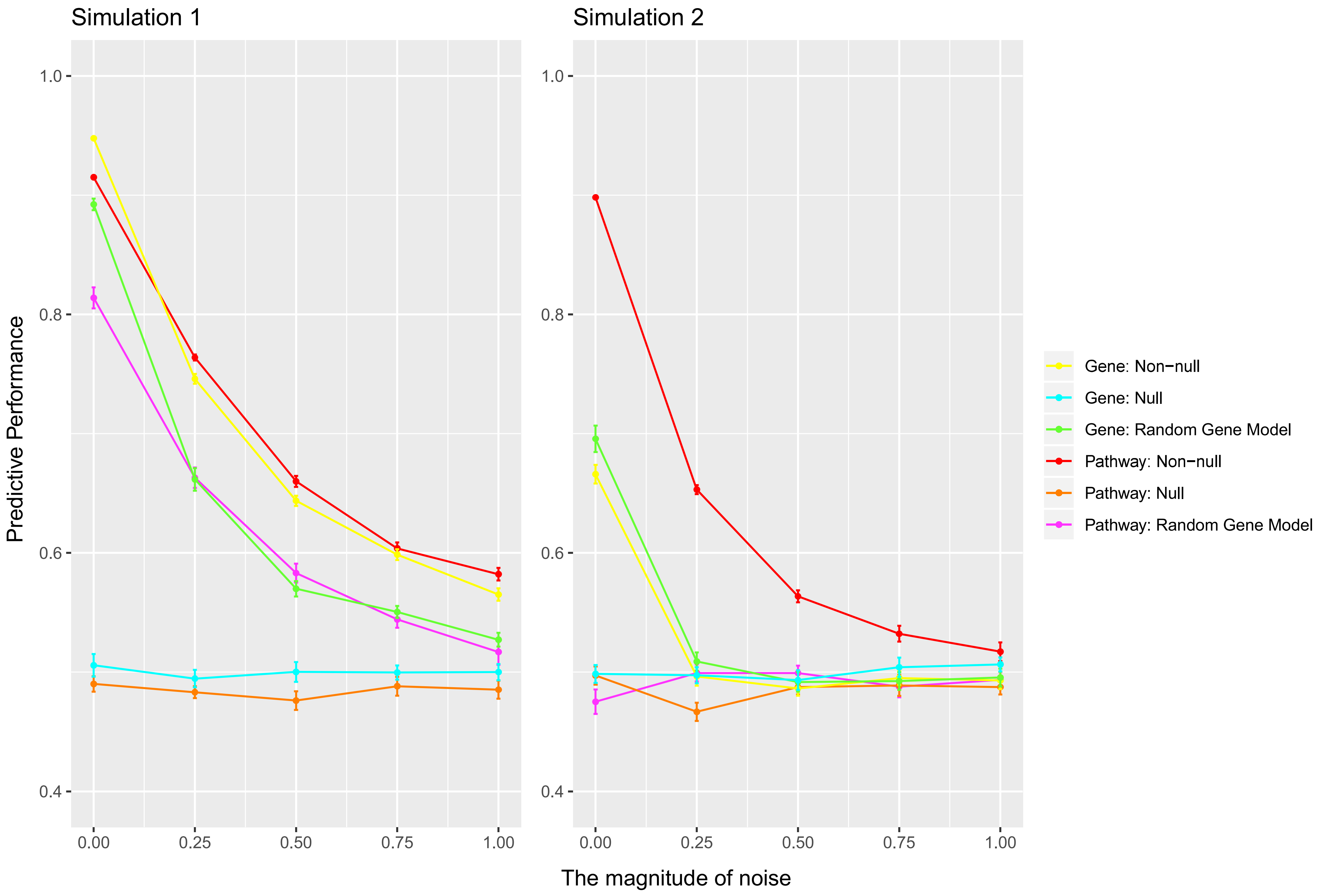

Figure S73: No.7 HALLMARK_IL6_JAK_STAT3_SIGNALING (size=87, absolute mean correlation=0.27)

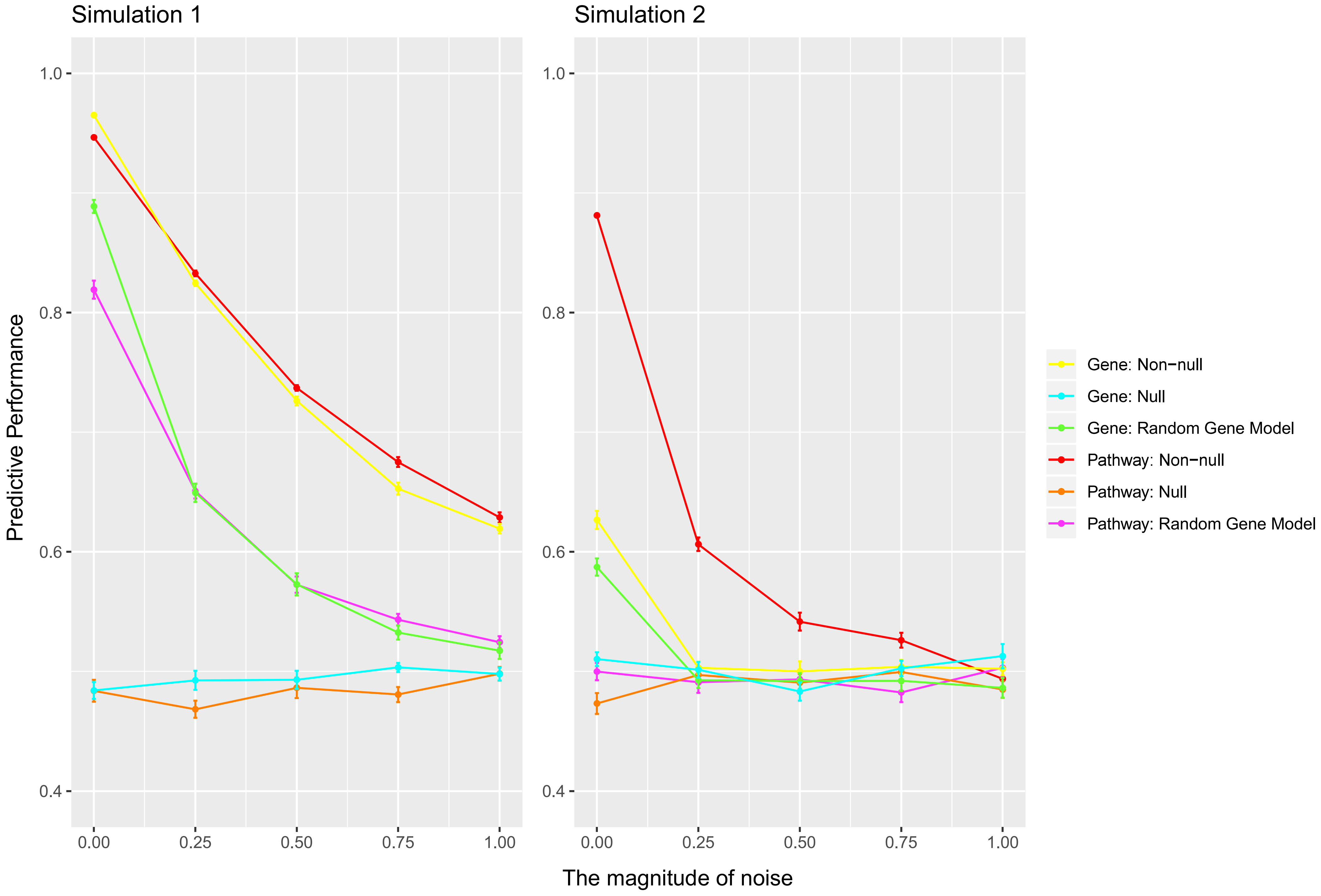

Figure S74: No.8 HALLMARK_DNA_REPAIR (size=150, absolute mean correlation=0.22)

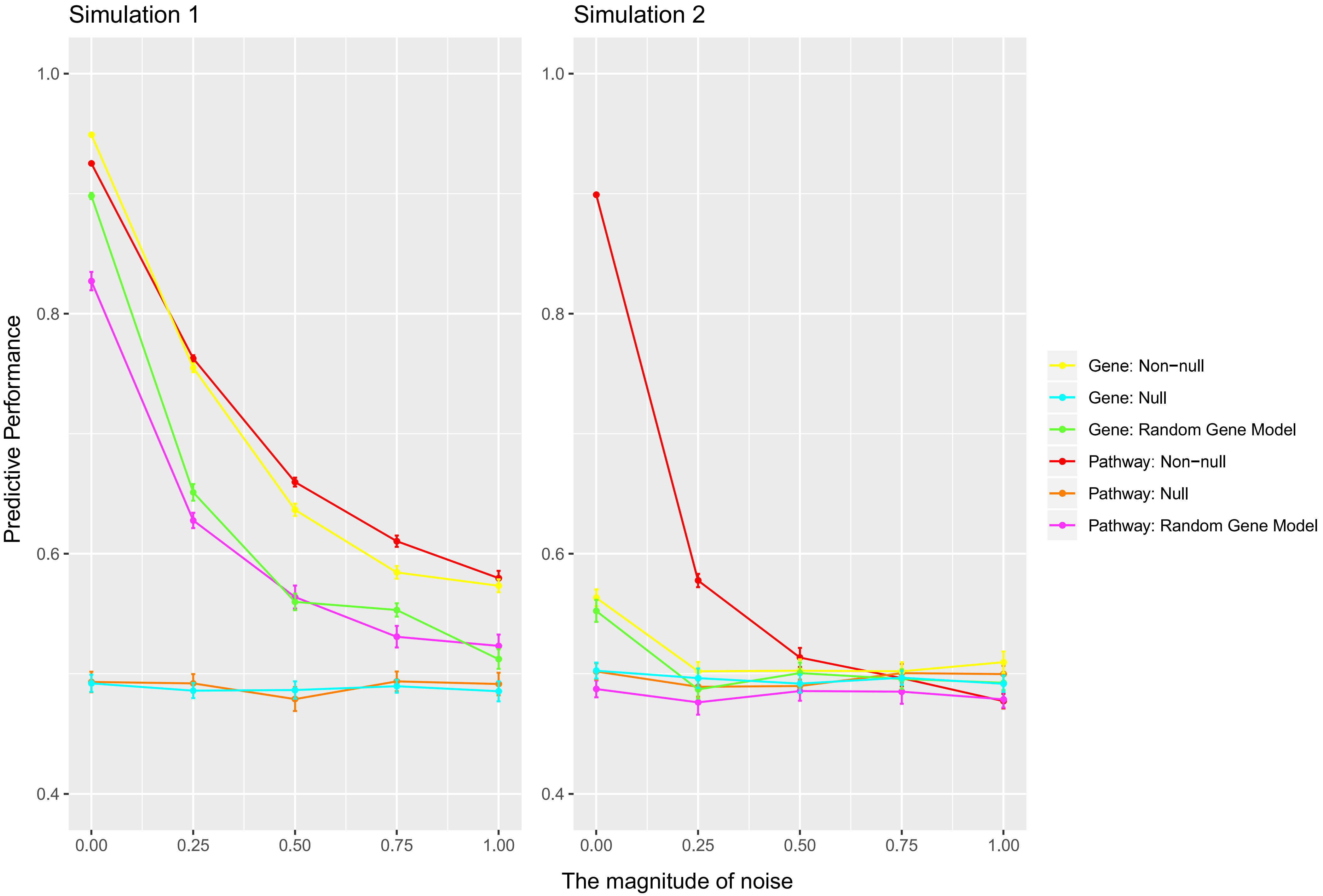

Figure S75: No.9 HALLMARK_G2M_CHECKPOINT (size=200, absolute mean correlation=0.32)

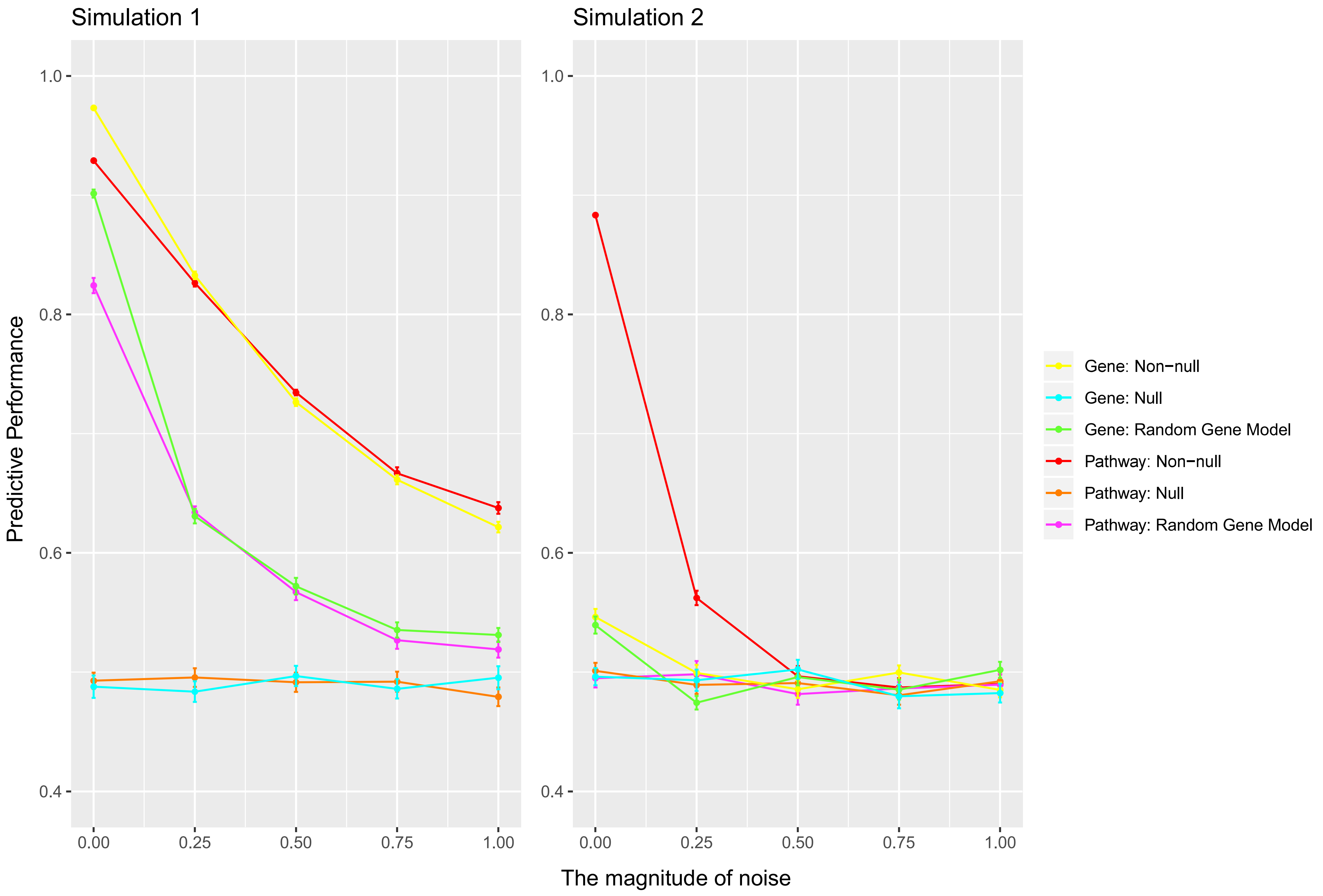

Figure S76: No.10 HALLMARK_APOPTOSIS (size=161, absolute mean correlation=0.22)

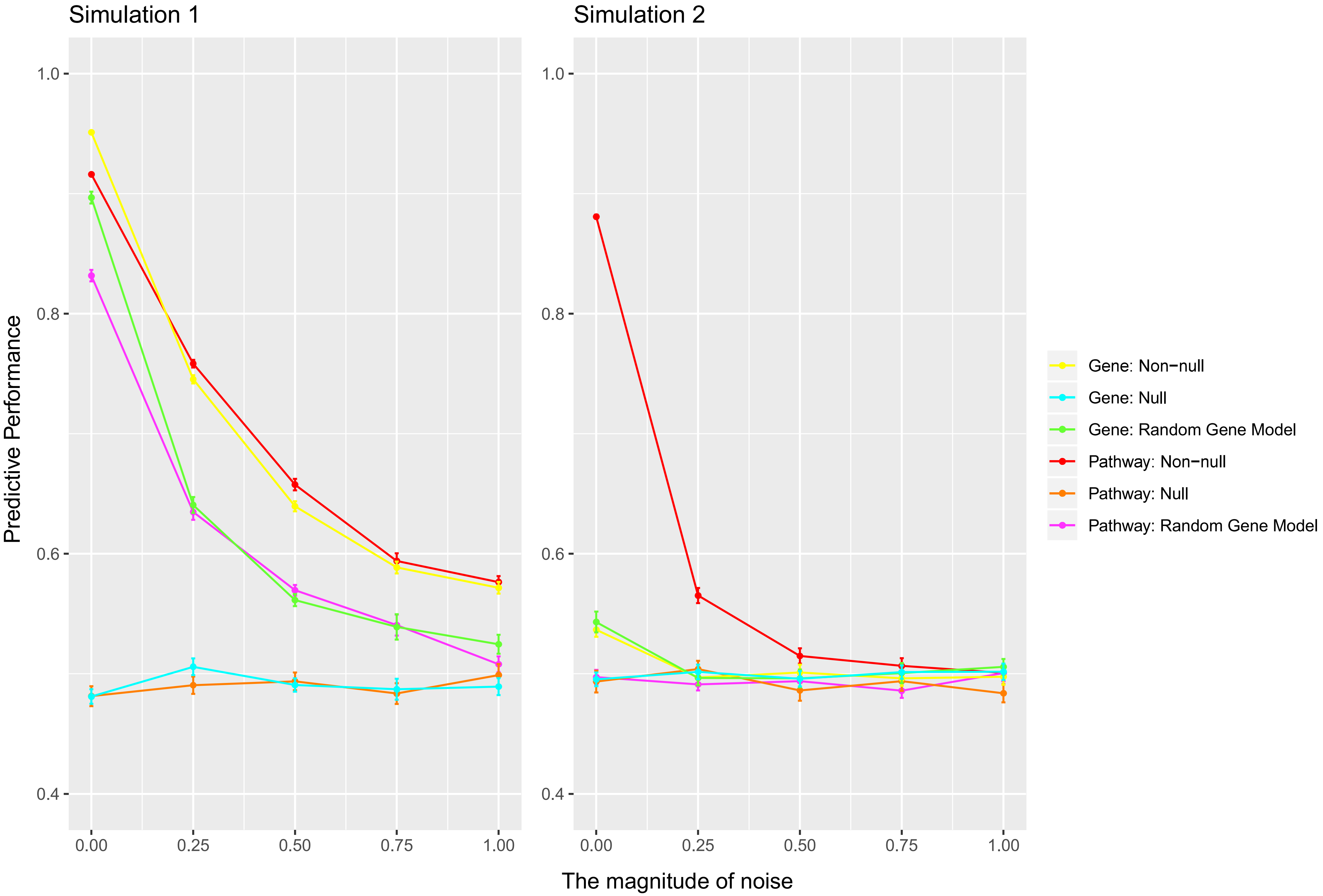

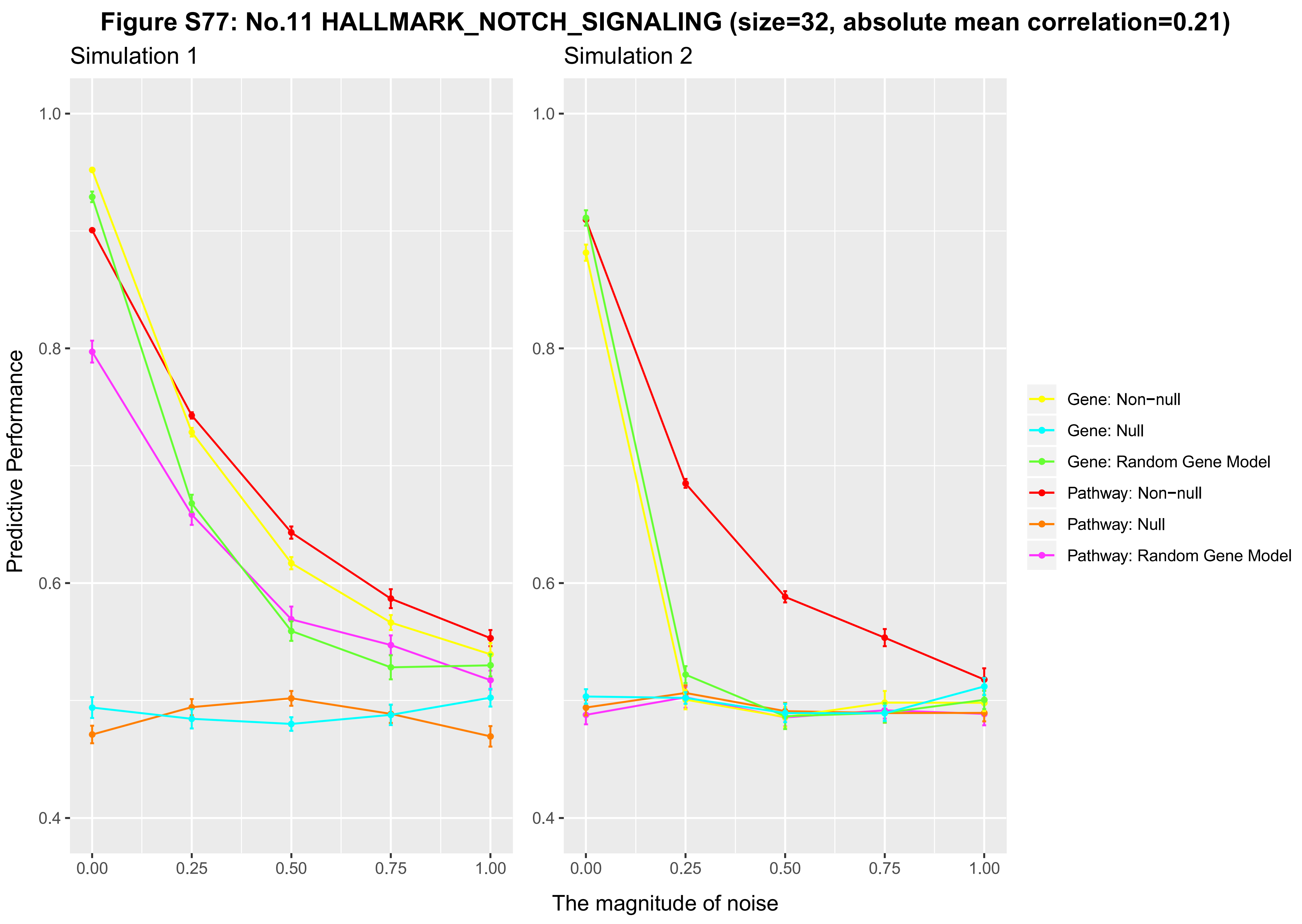
Figure S77: No.11 HALLMARK_NOTCH_SIGNALING (size=32, absolute mean correlation=0.21)
Simulation 1
Simulation 2
Predictive Performance
1.0
0.8
0.6
0.4
0.00
0.25
0.50
0.75
1.00
The magnitude of noise
Gene: Non−null
Gene: Null
Gene: Random Gene Model
Pathway: Non−null
Pathway: Null
Pathway: Random Gene Model

Figure S78: No.12 HALLMARK_ADIPOGENESIS (size=200, absolute mean correlation=0.19)

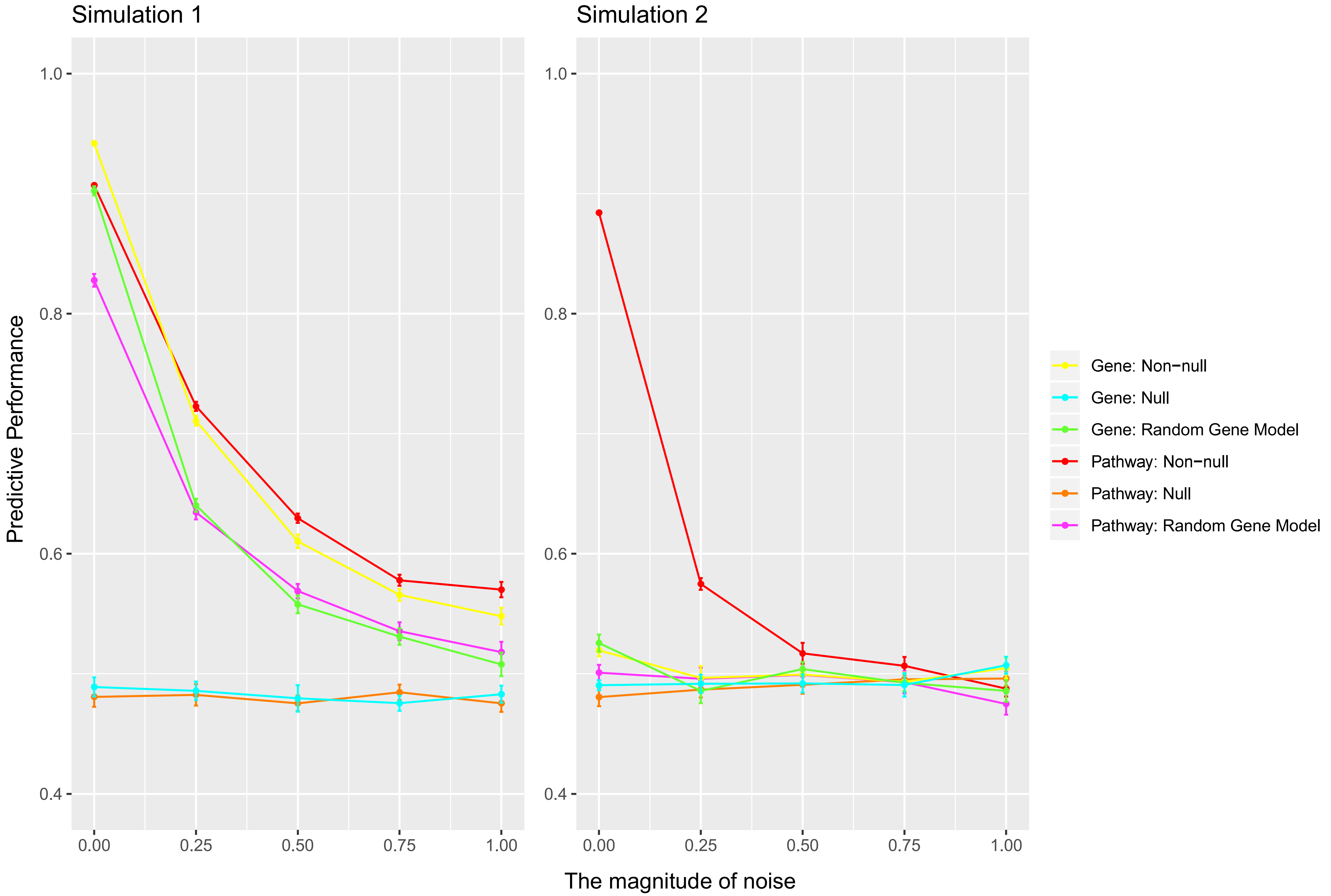

Figure S79: No.13 HALLMARK_ESTROGEN_RESPONSE_EARLY (size=200, absolute mean correlation=0.17

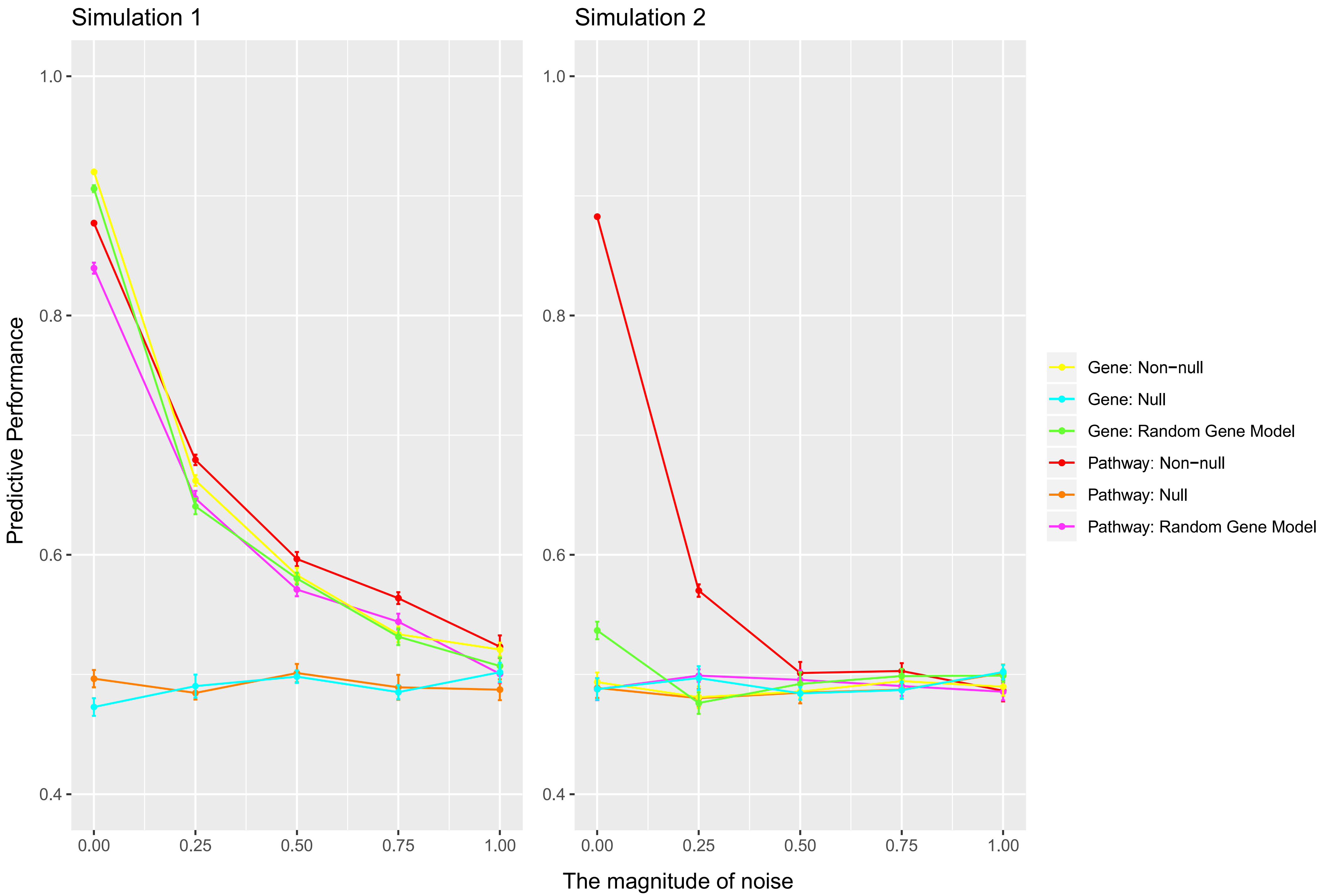

Figure S80: No.14 HALLMARK_ESTROGEN_RESPONSE_LATE (size=200, absolute mean correlation=0.17)

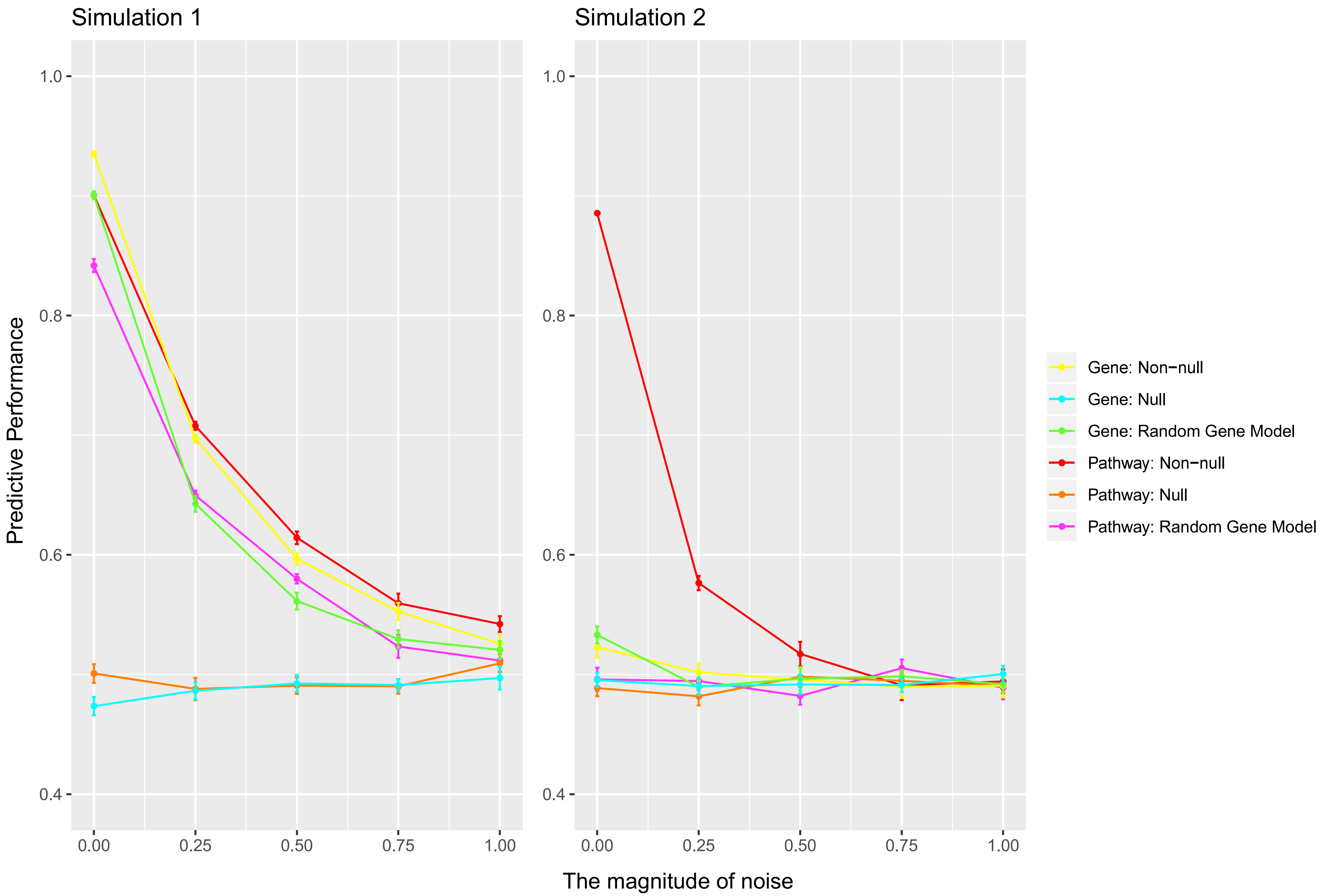

Figure S81: No.15 HALLMARK_ANDROGEN_RESPONSE (size=101, absolute mean correlation=0.18)

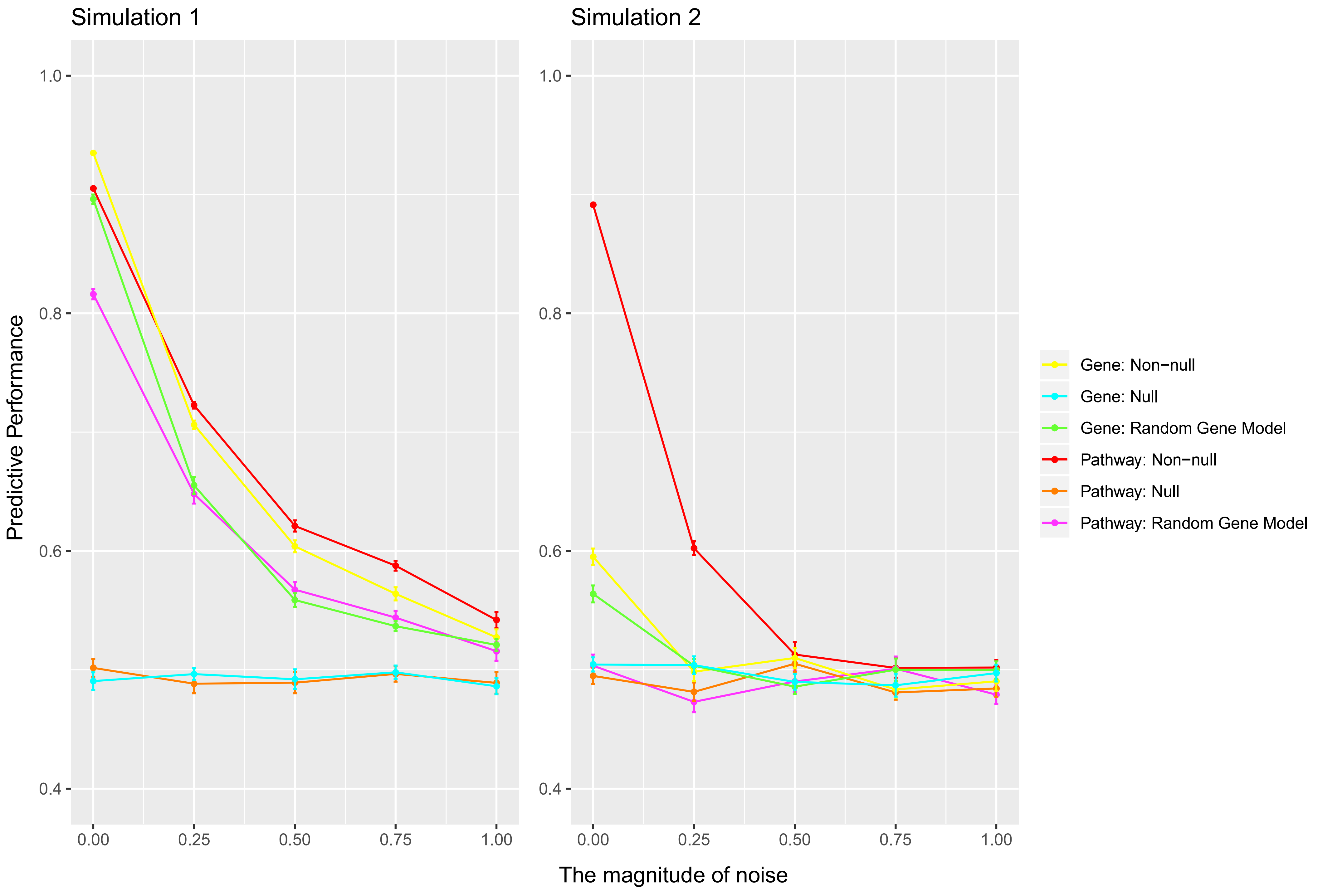

Figure S82: No.16 HALLMARK_MYOGENESIS (size=200, absolute mean correlation=0.18)

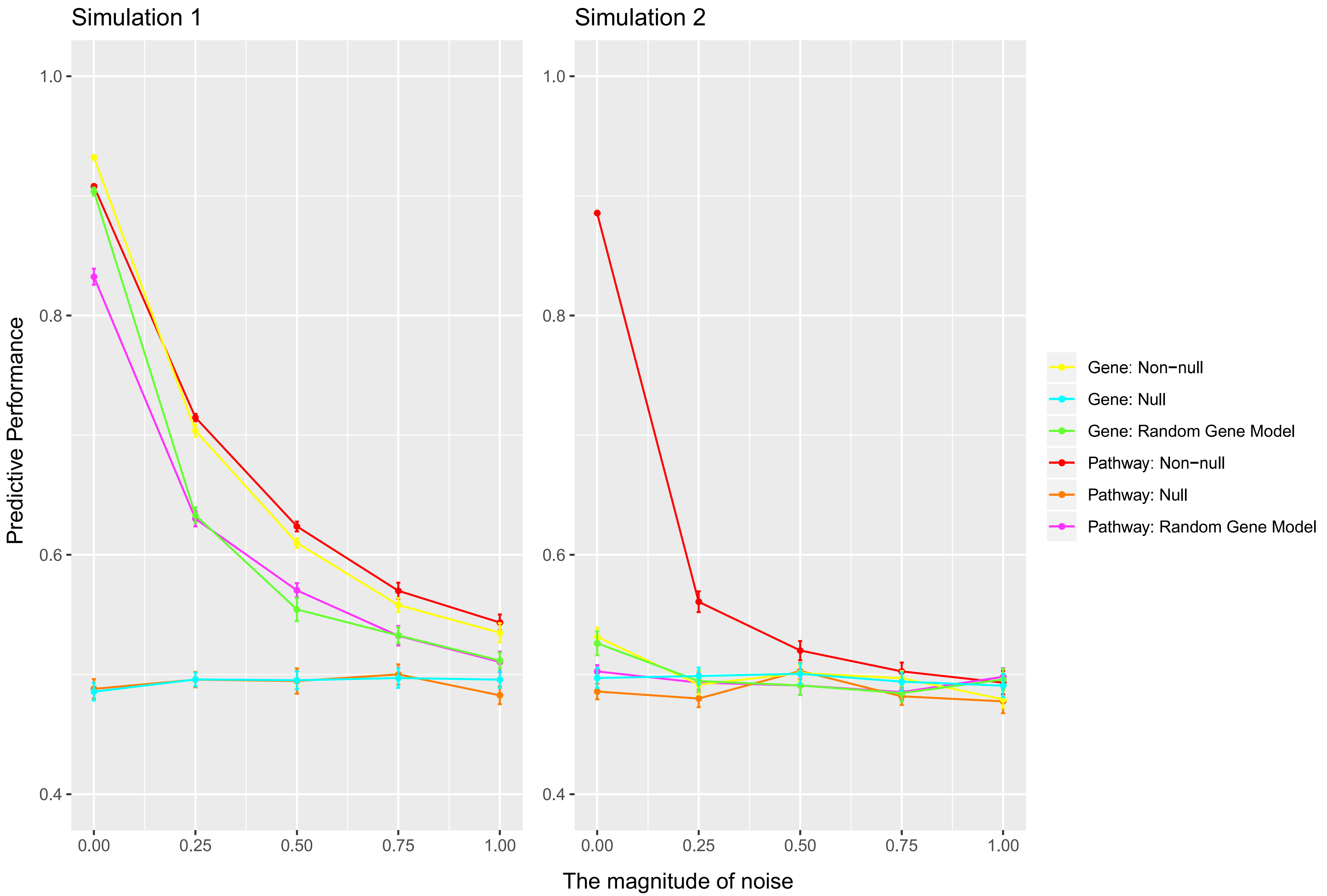

Figure S83: No.17 HALLMARK_PROTEIN_SECRETION (size=96, absolute mean correlation=0.23)

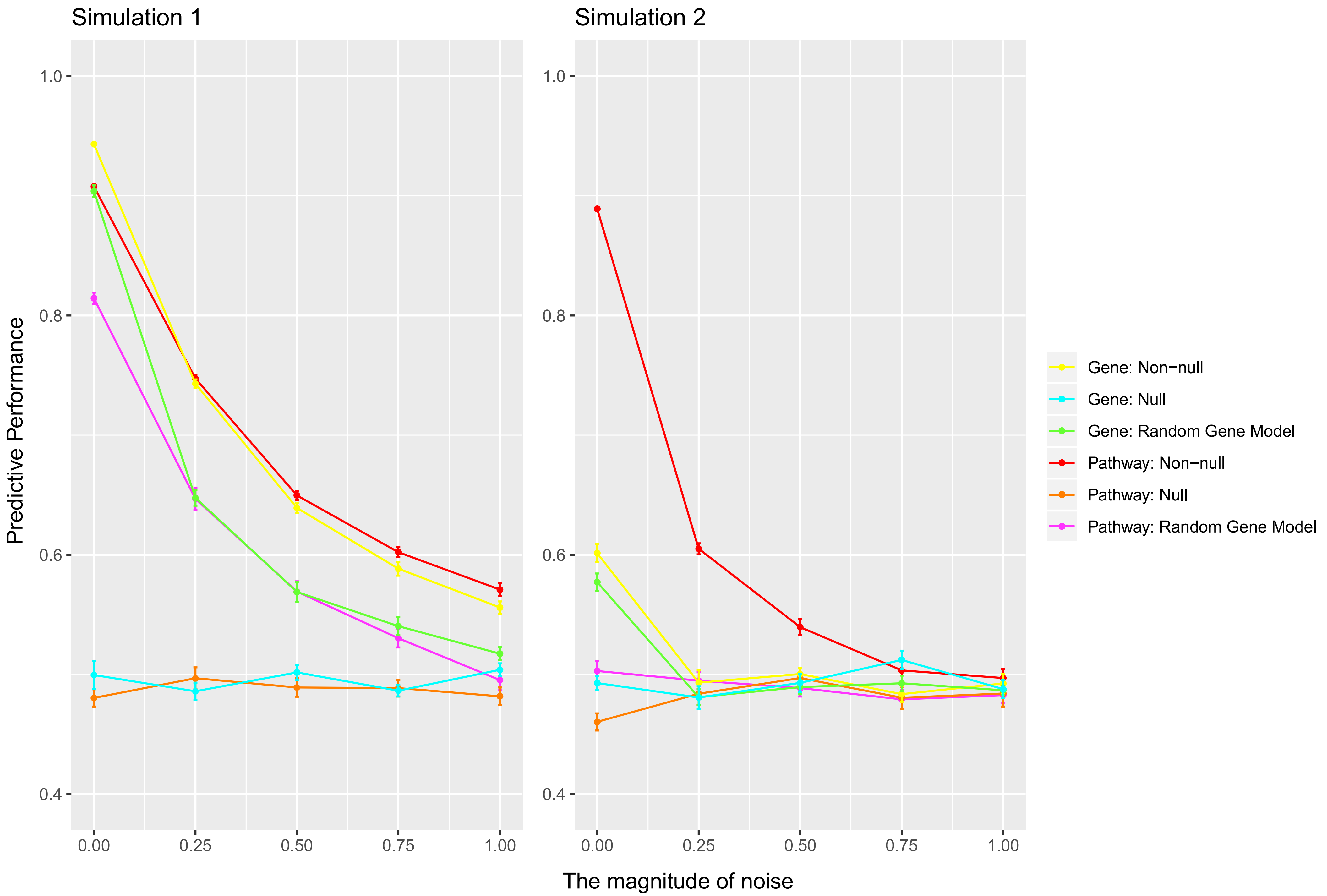

Figure S84: No.18 HALLMARK_INTERFERON_ALPHA_RESPONSE (size=97, absolute mean correlation=0.39

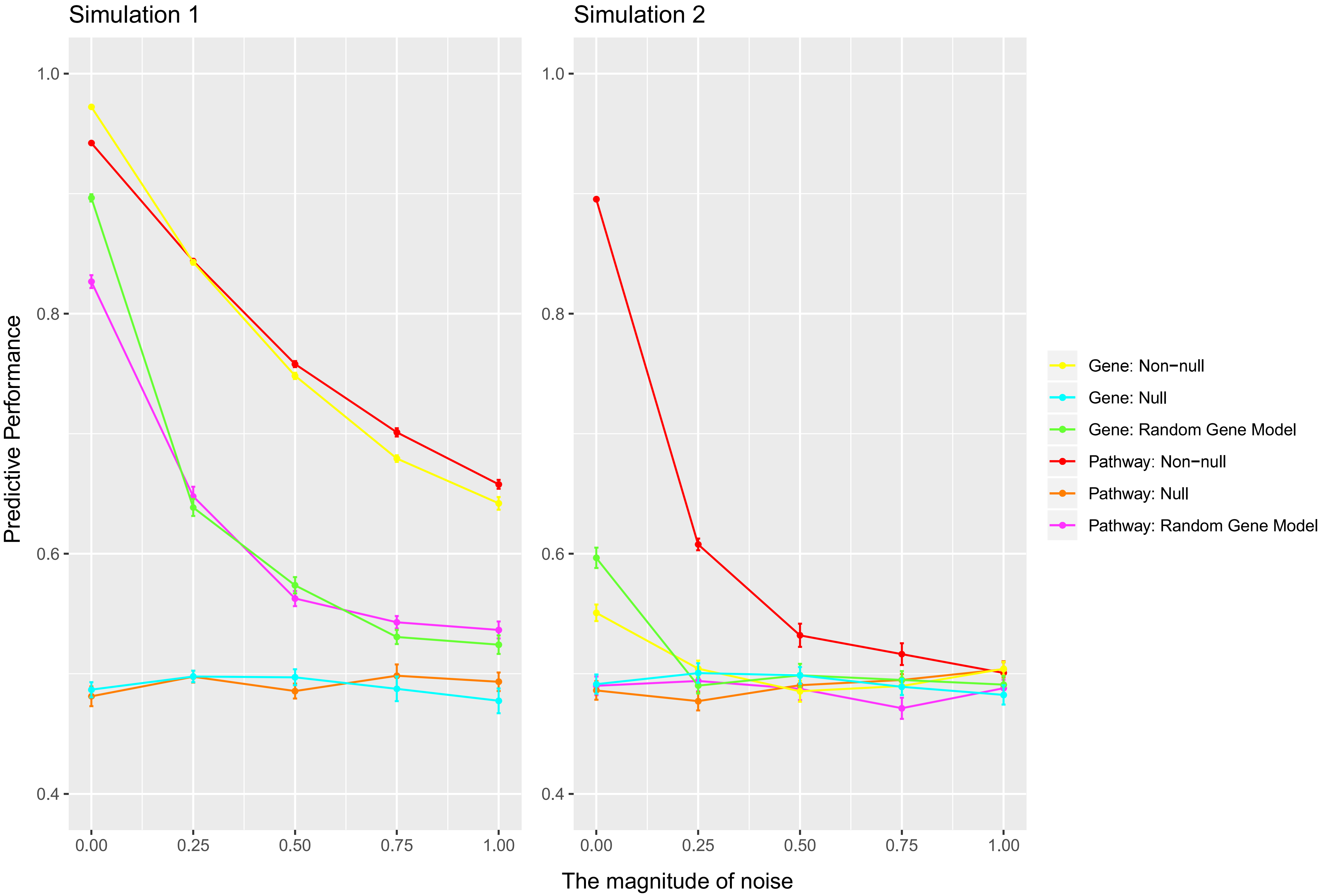

Figure S85: No.19 HALLMARK_INTERFERON_GAMMA_RESPONSE (size=200, absolute mean correlation=0.3

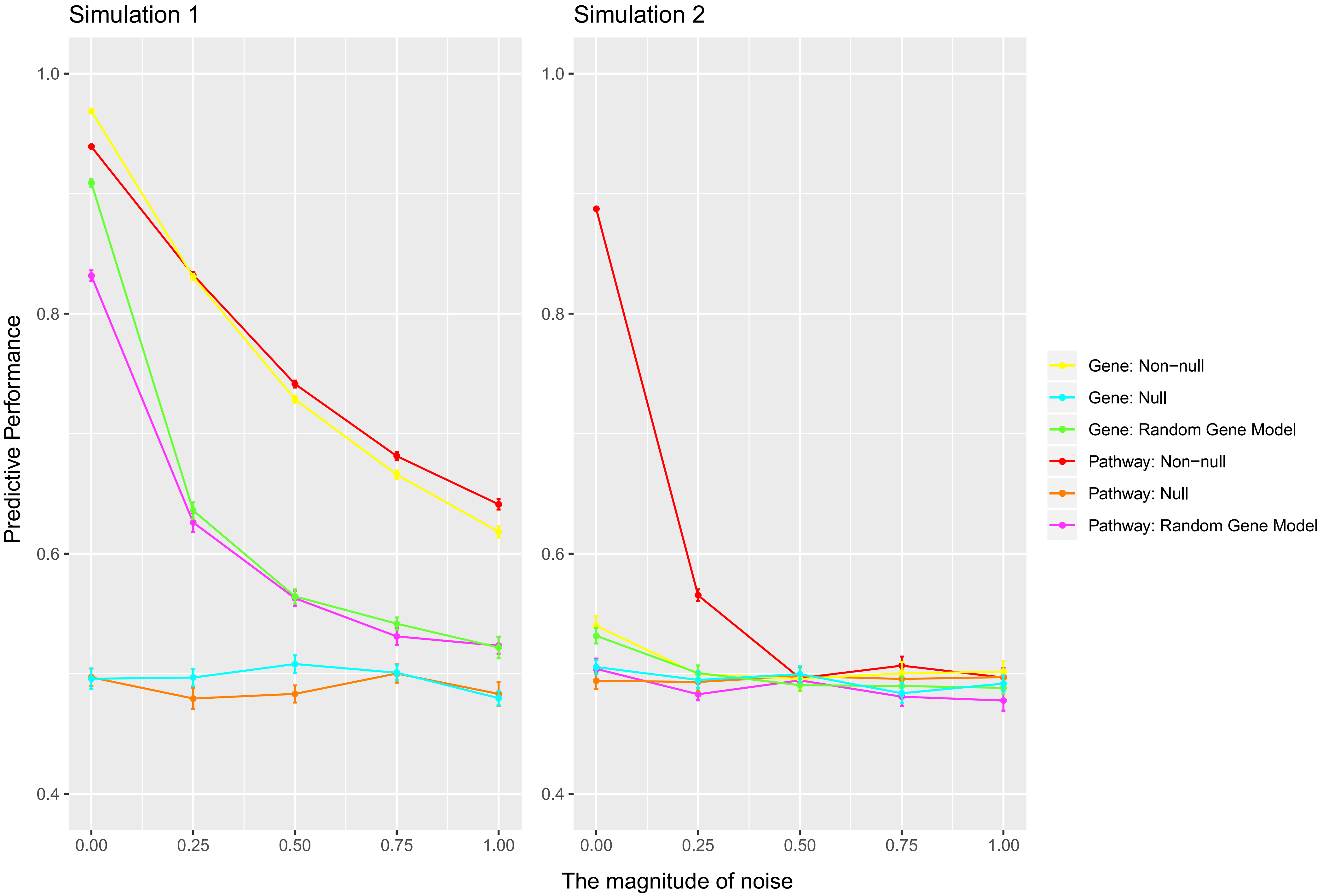

Figure S86: No.20 HALLMARK_APICAL_JUNCTION (size=200, absolute mean correlation=0.18)

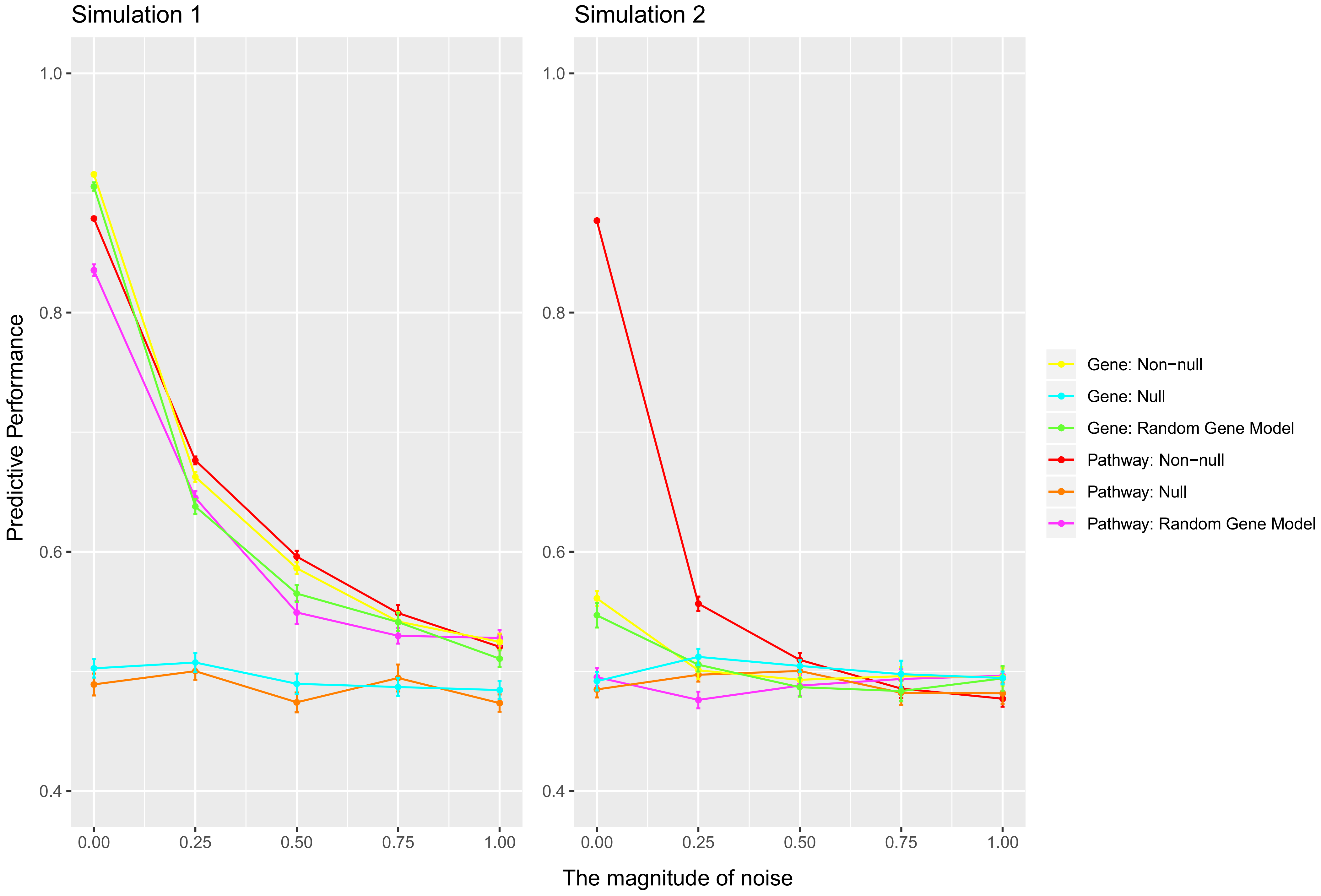

Figure S87: No.21 HALLMARK_APICAL_SURFACE (size=44, absolute mean correlation=0.18)

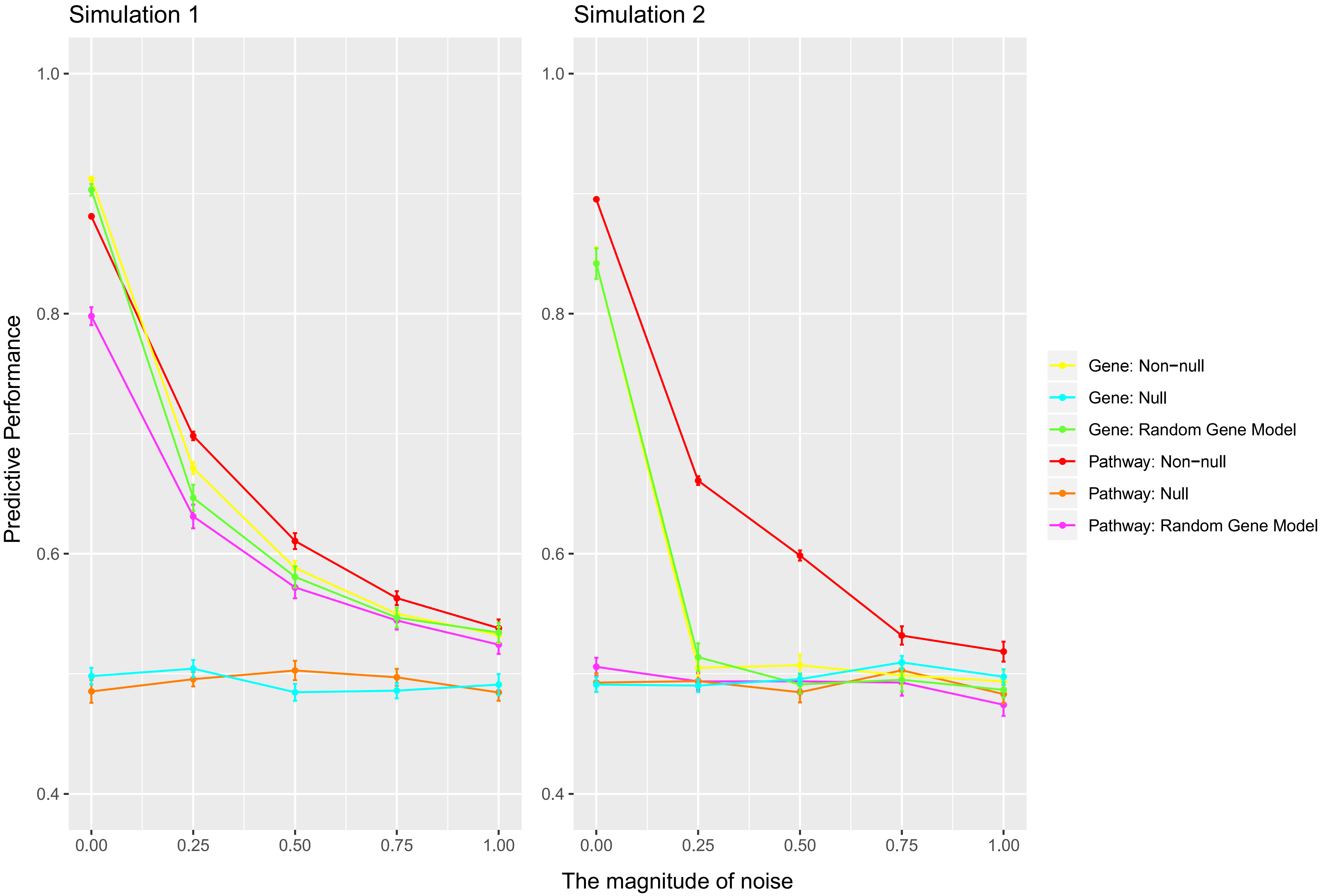

Figure S88: No.22 HALLMARK_HEDGEHOG_SIGNALING (size=36, absolute mean correlation=0.20)

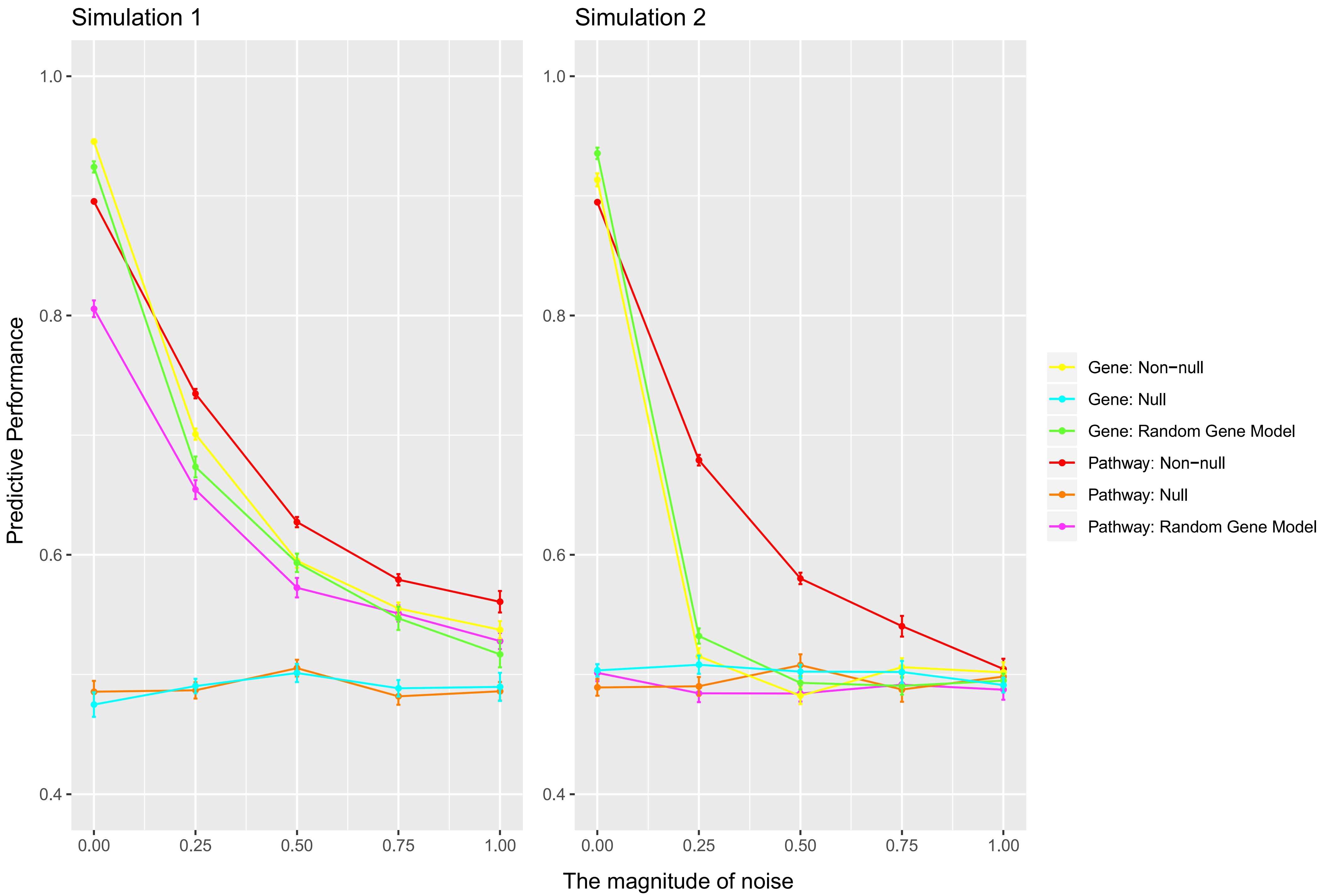

Figure S89: No.23 HALLMARK_COMPLEMENT (size=200, absolute mean correlation=0.23)

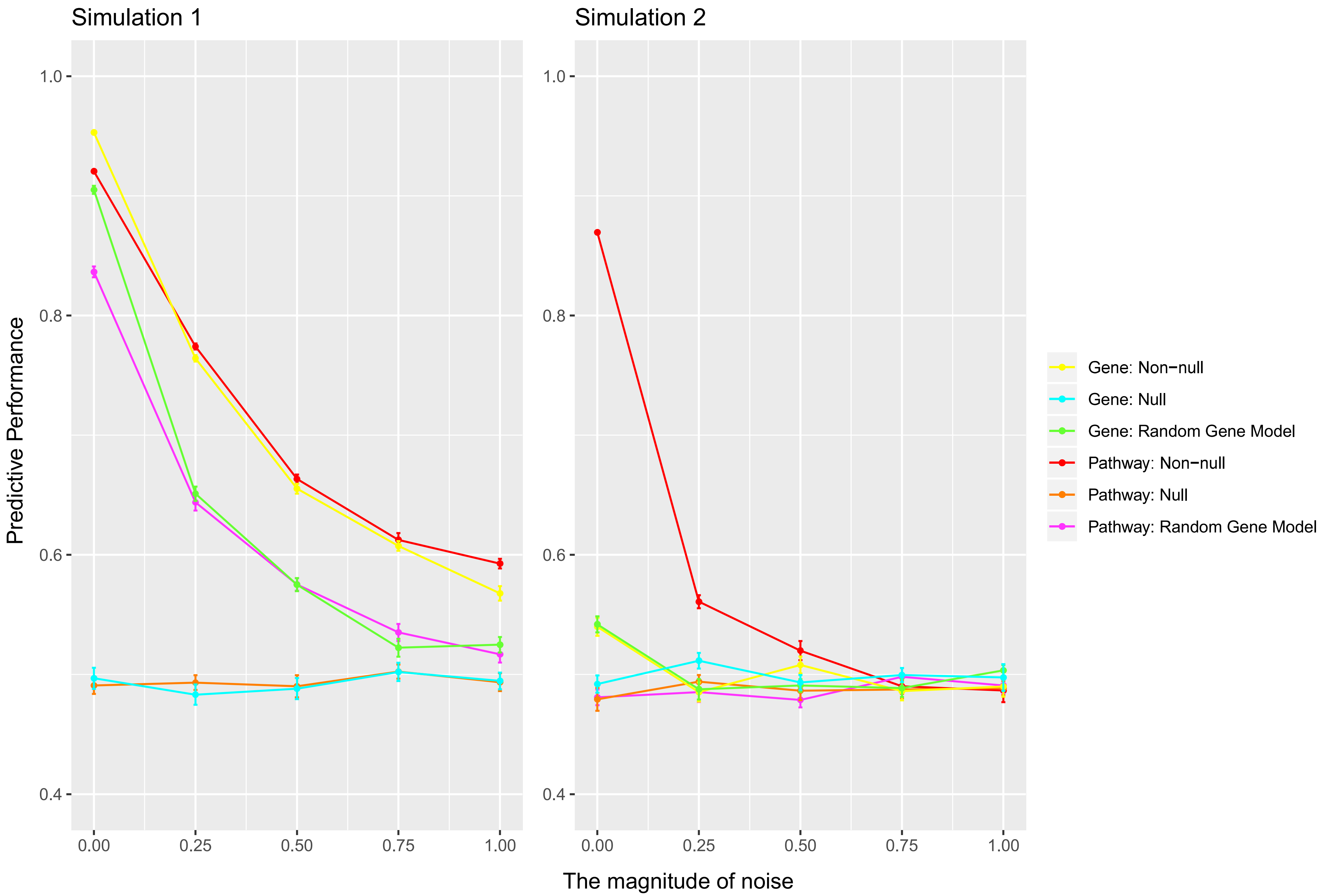

Figure S90: No.24 HALLMARK_UNFOLDED_PROTEIN_RESPONSE (size=113, absolute mean correlation=0.2

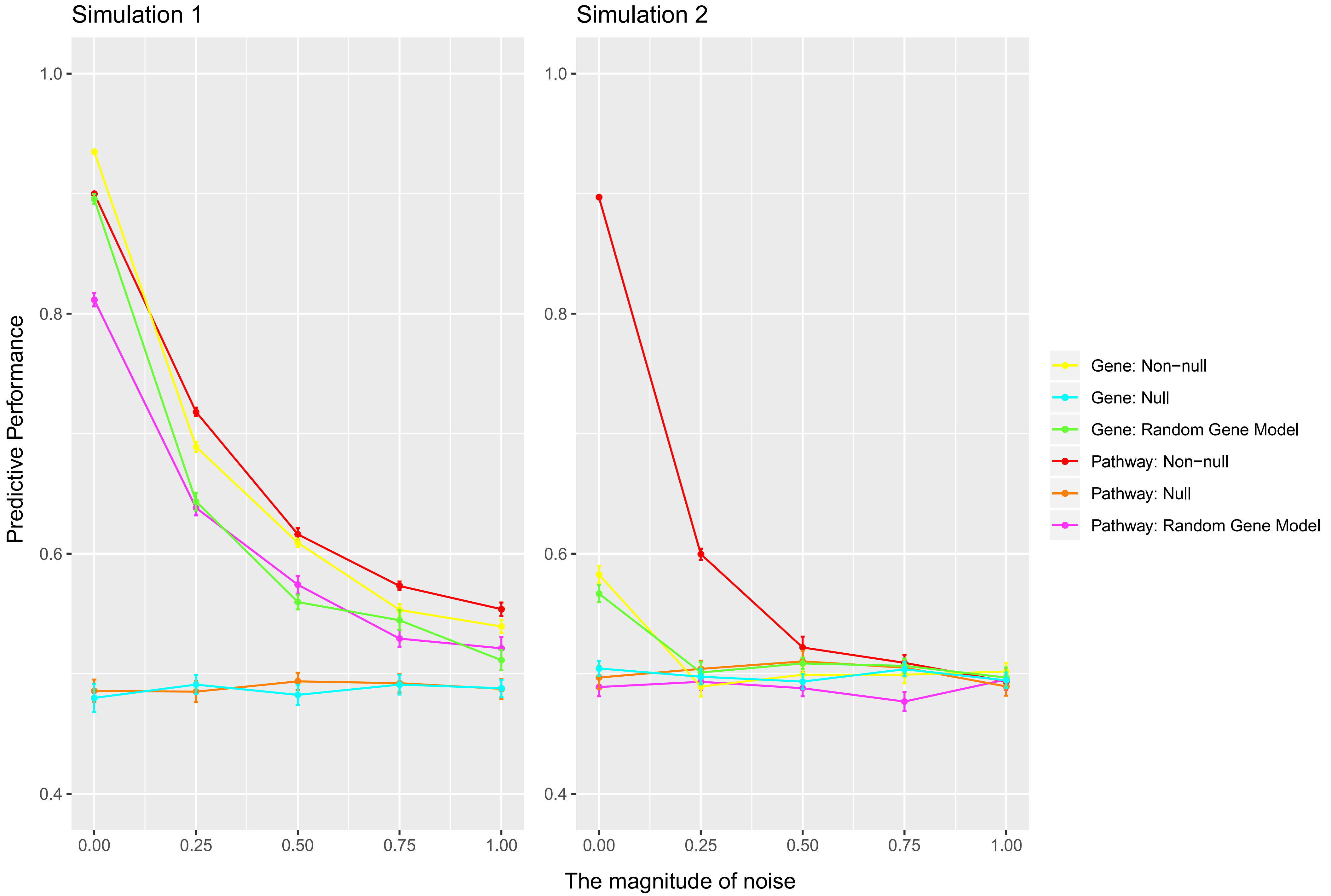

Figure S91: No.25 HALLMARK_PI3K_AKT_MTOR_SIGNALING (size=105, absolute mean correlation=0.21)

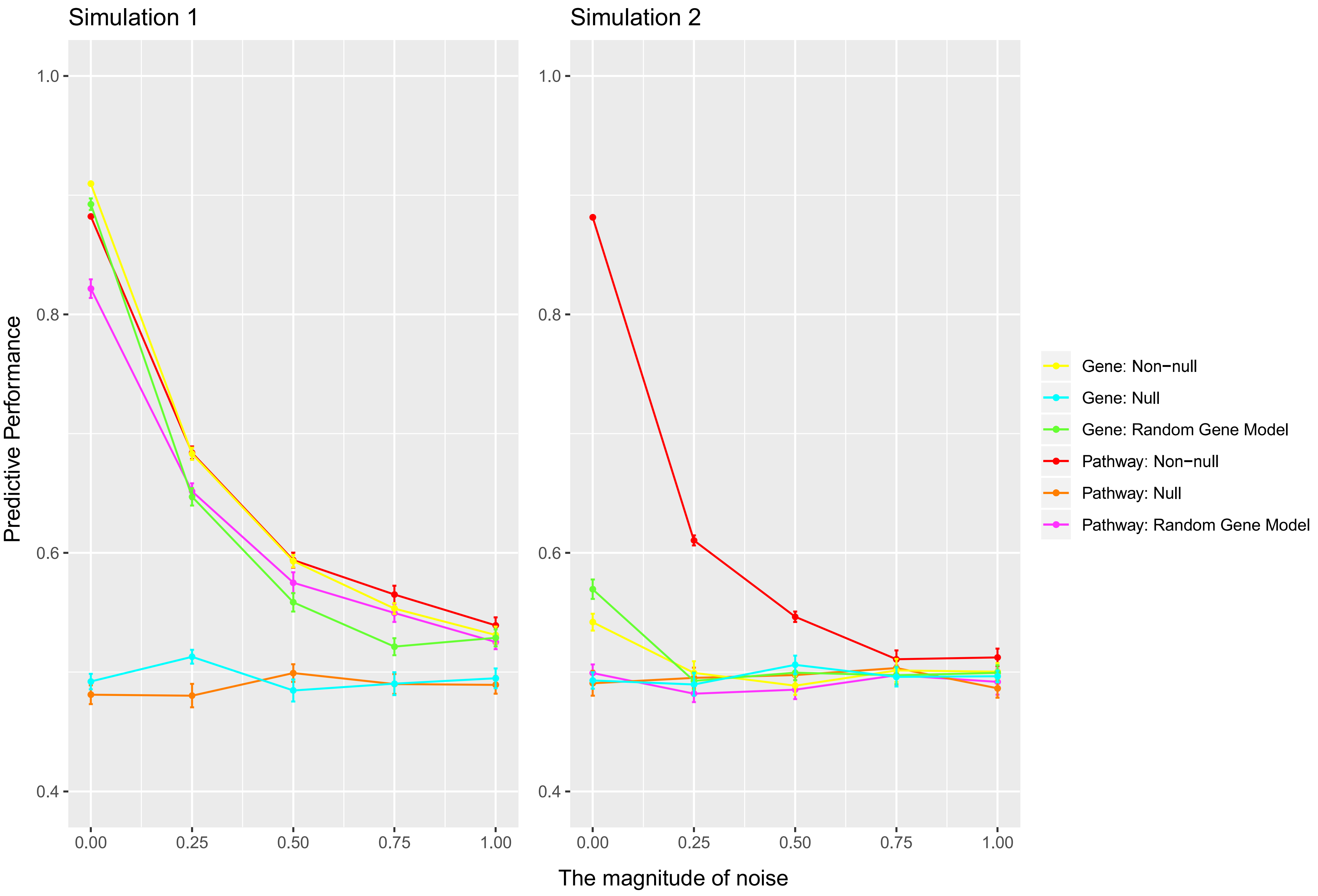

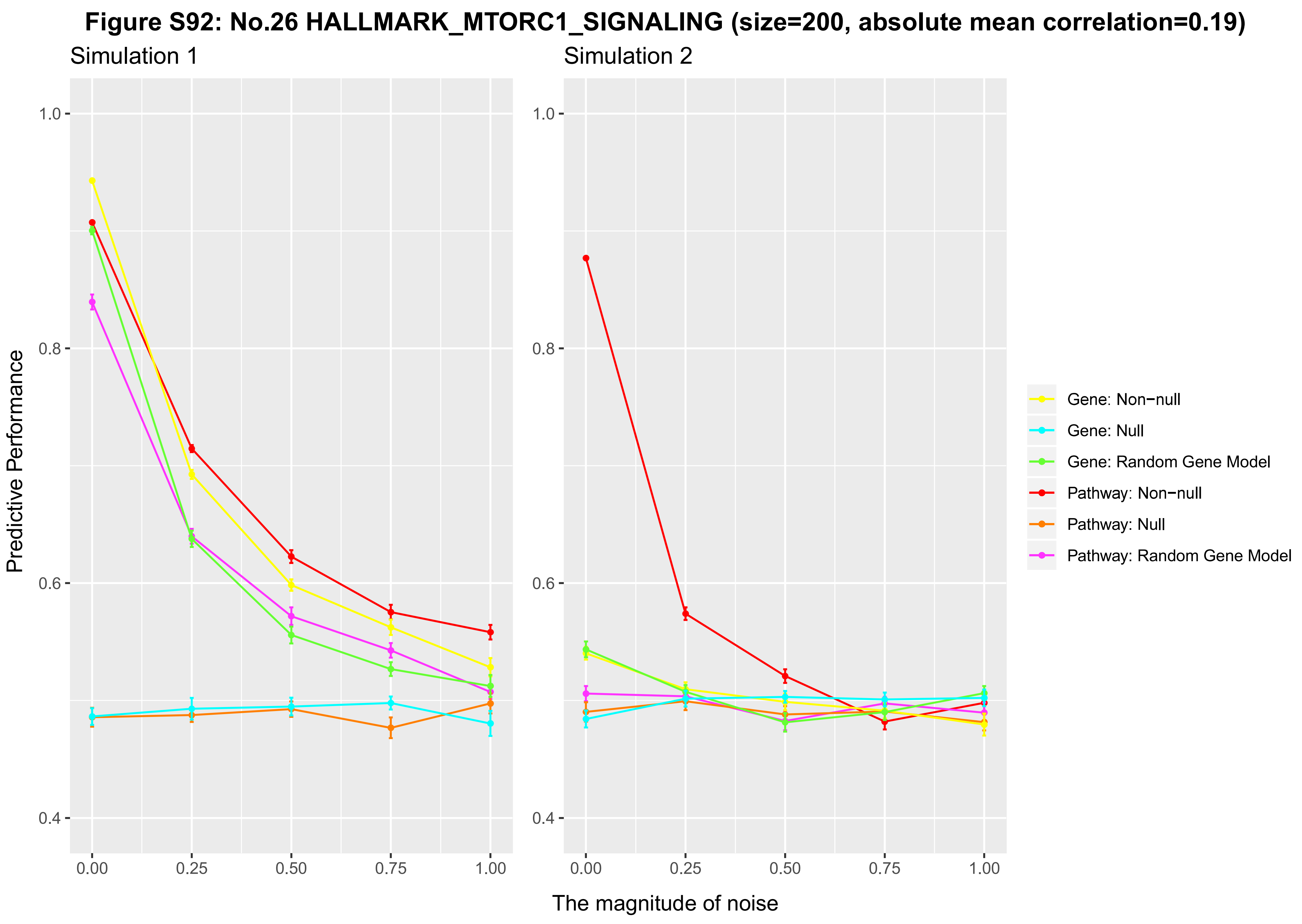
Figure S92: No.26 HALLMARK_MTORC1_SIGNALING (size=200, absolute mean correlation=0.19)
Simulation 1
Simulation 2
Predictive Performance
The magnitude of noise
1.0
0.8
0.6
0.4
0.00
0.25
0.50
0.75
1.00
Gene: Non−null
Gene: Null
Gene: Random Gene Model
Pathway: Non−null
Pathway: Null
Pathway: Random Gene Model

Figure S93: No.27 HALLMARK_E2F_TARGETS (size=200, absolute mean correlation=0.34)

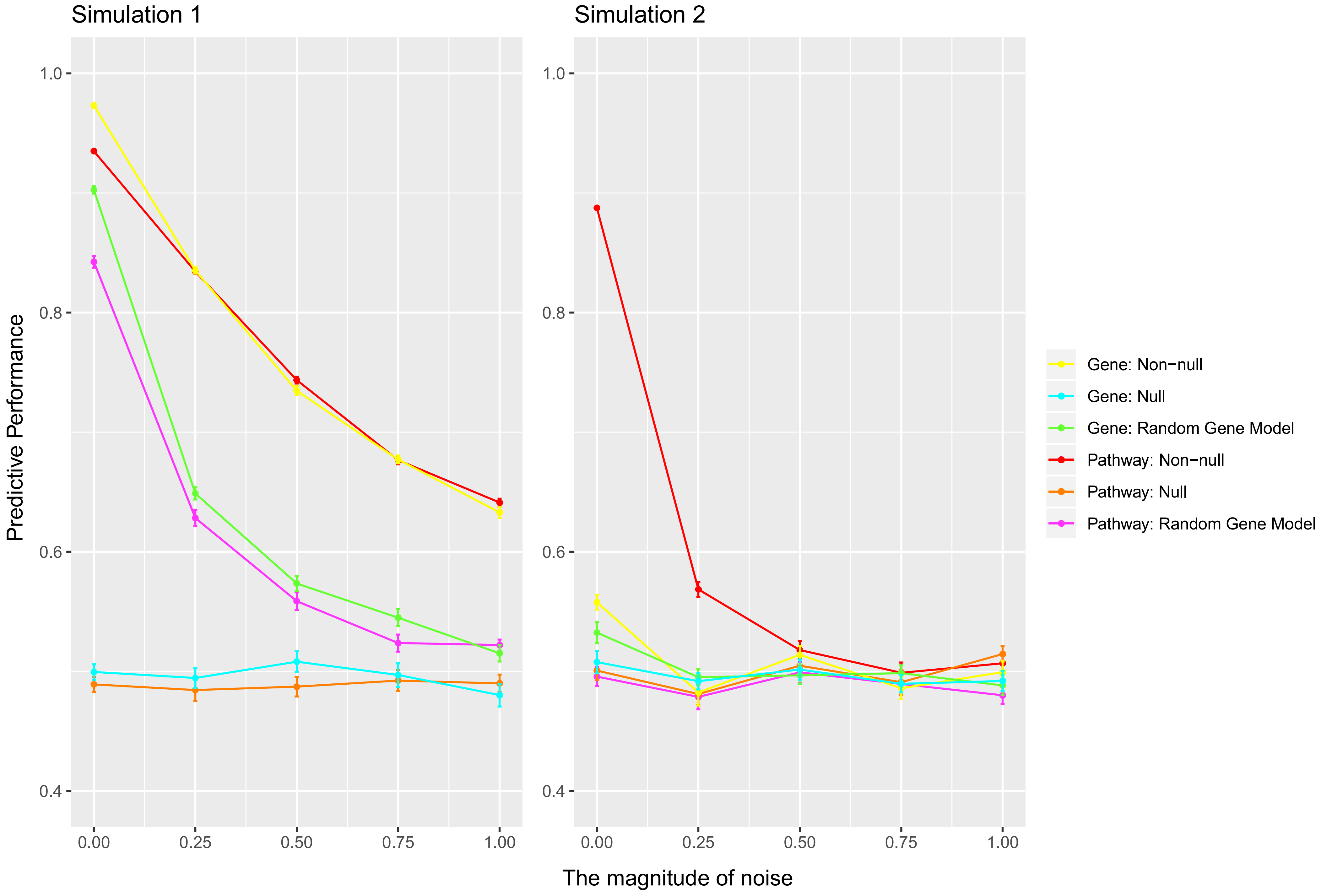

Figure S94: No.28 HALLMARK_MYC_TARGETS_V1 (size=200, absolute mean correlation=0.26)

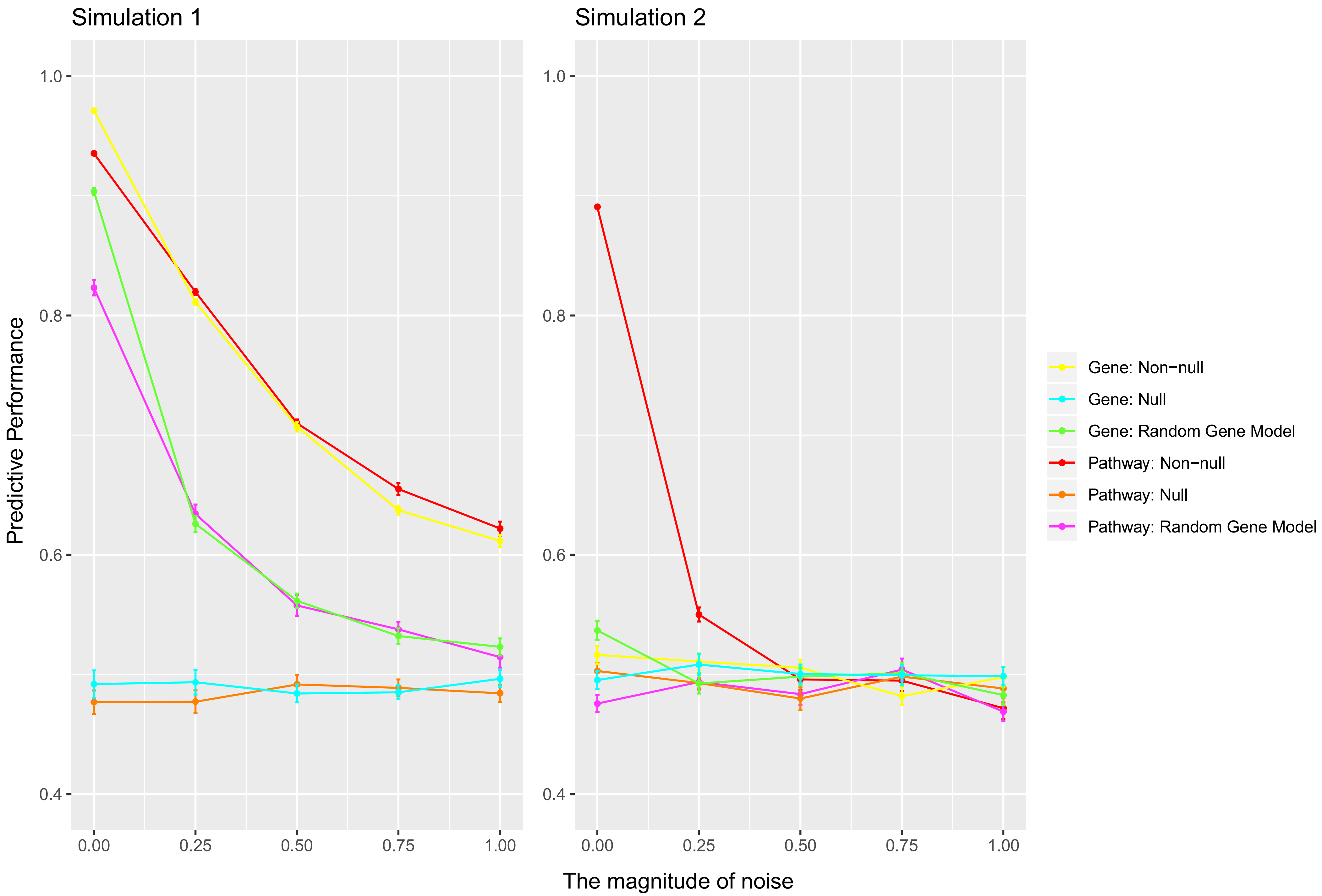

Figure S95: No.29 HALLMARK_MYC_TARGETS_V2 (size=58, absolute mean correlation=0.27)

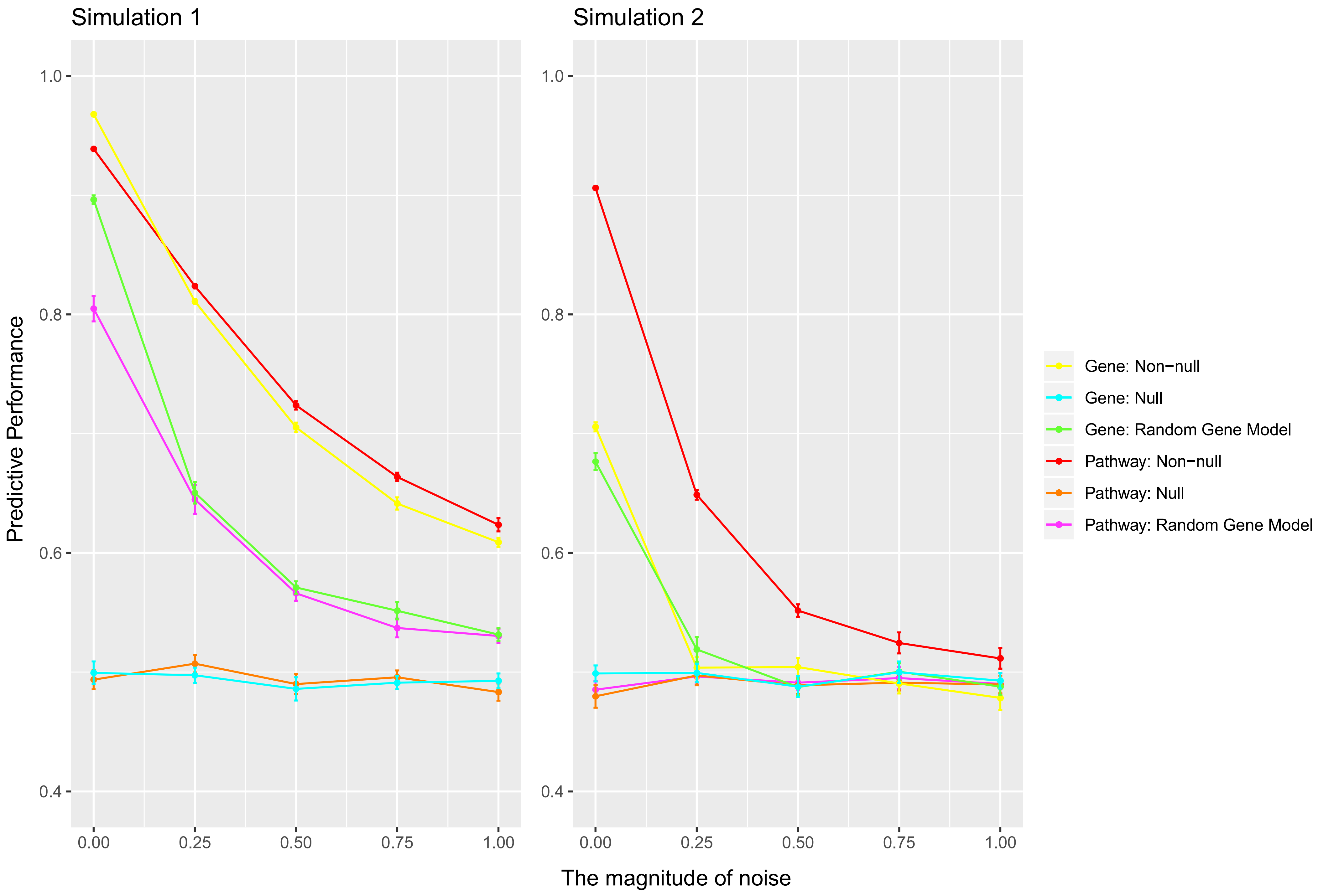

ure S96: No.30 HALLMARK_EPITHELIAL_MESENCHYMAL_TRANSITION (size=200, absolute mean correlatior

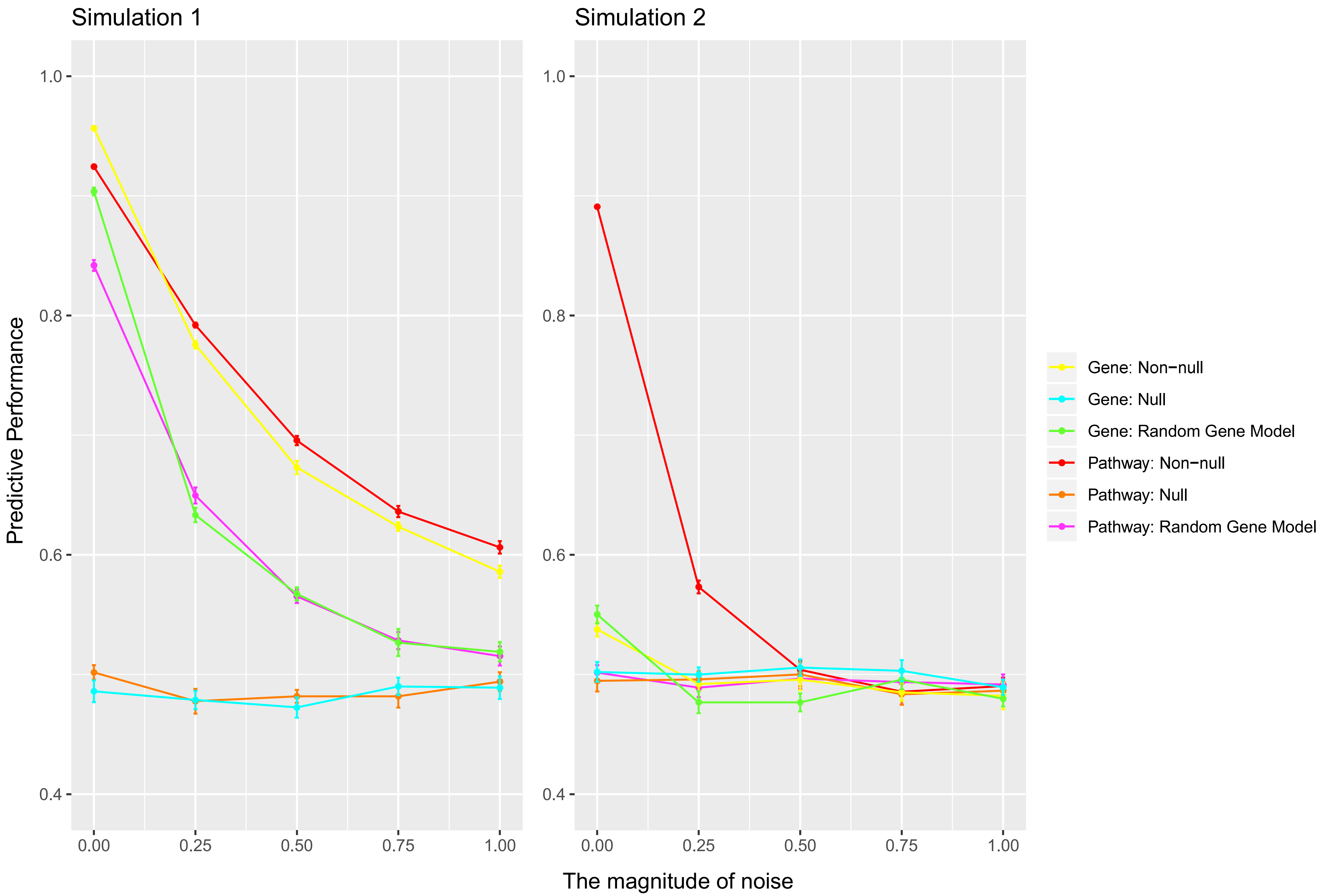

Figure S97: No.31 HALLMARK_INFLAMMATORY_RESPONSE (size=200, absolute mean correlation=0.25)

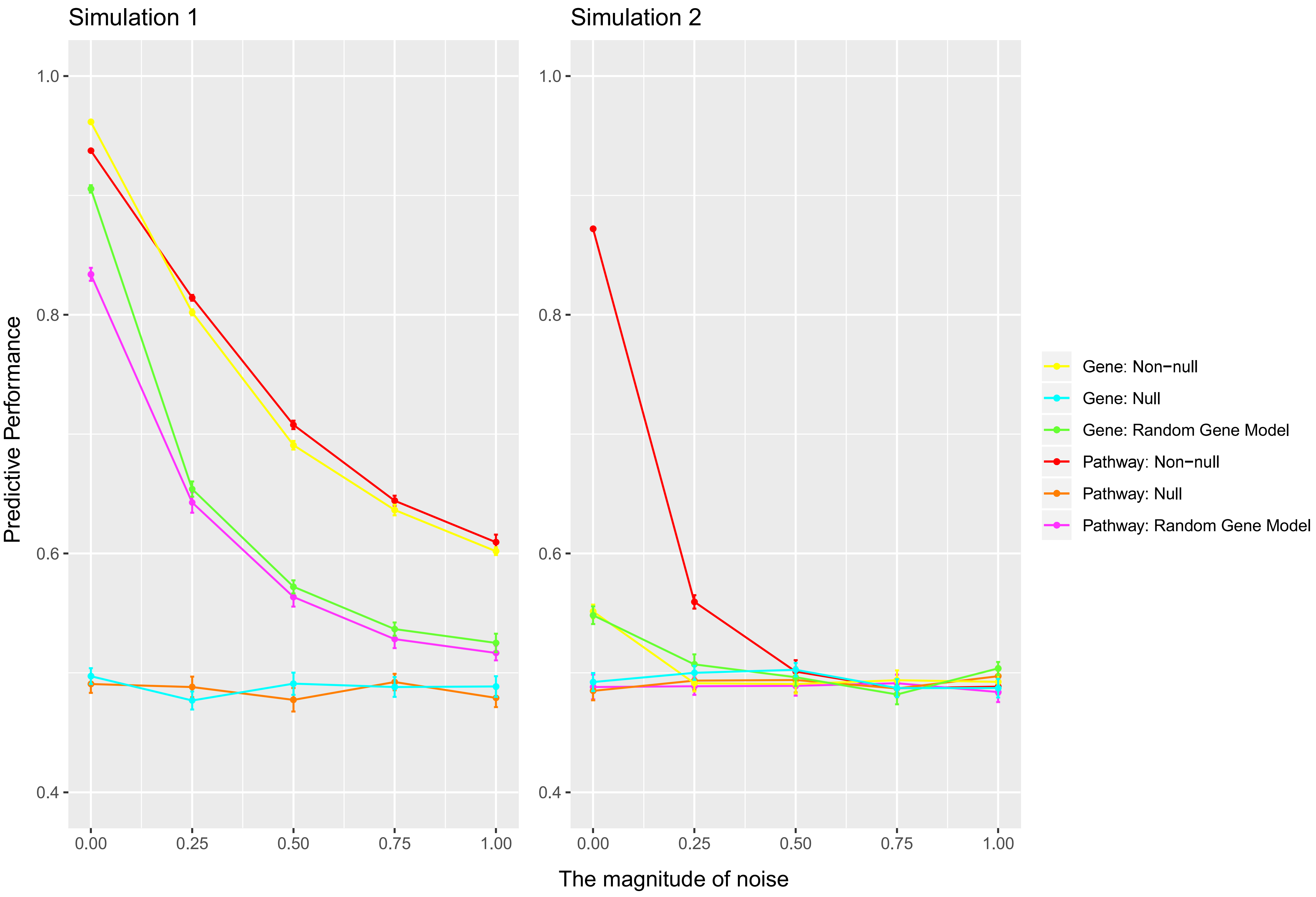

Figure S98: No.32 HALLMARK_XENOBIOTIC_METABOLISM (size=200, absolute mean correlation=0.16)

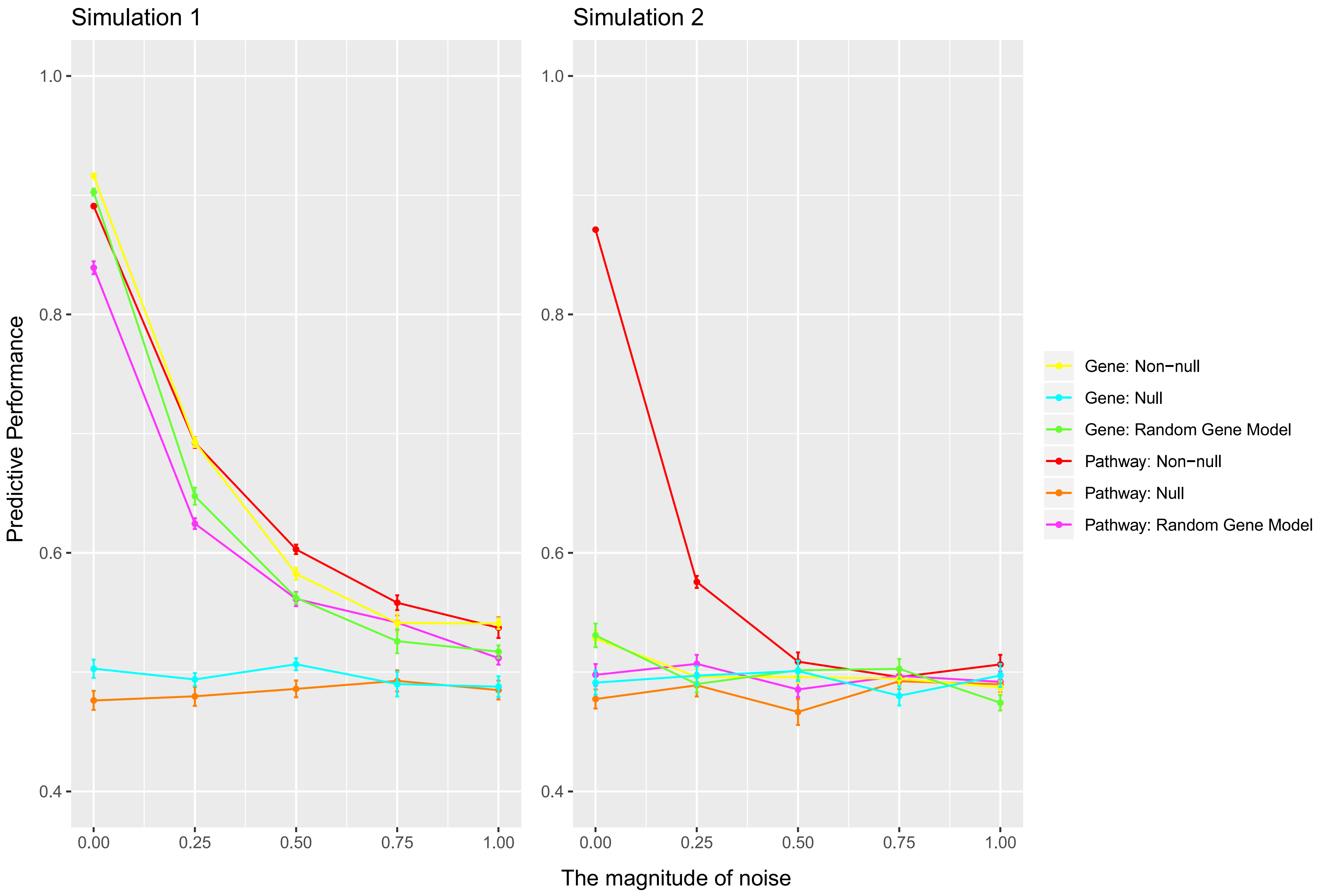

Figure S99: No.33 HALLMARK_FATTY_ACID_METABOLISM (size=158, absolute mean correlation=0.17)

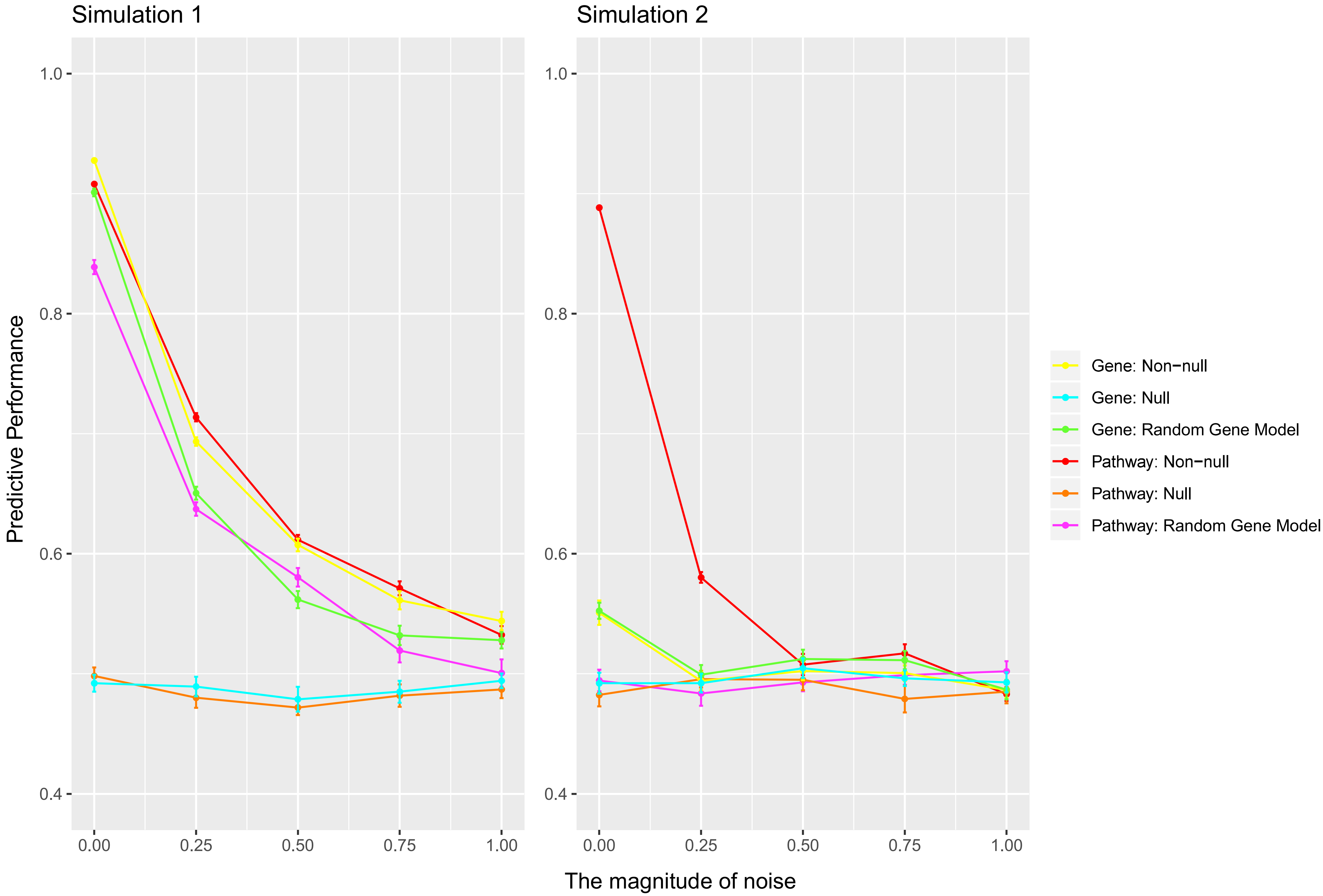

Figure S100: No.34 HALLMARK_OXIDATIVE_PHOSPHORYLATION (size=200, absolute mean correlation=0.2

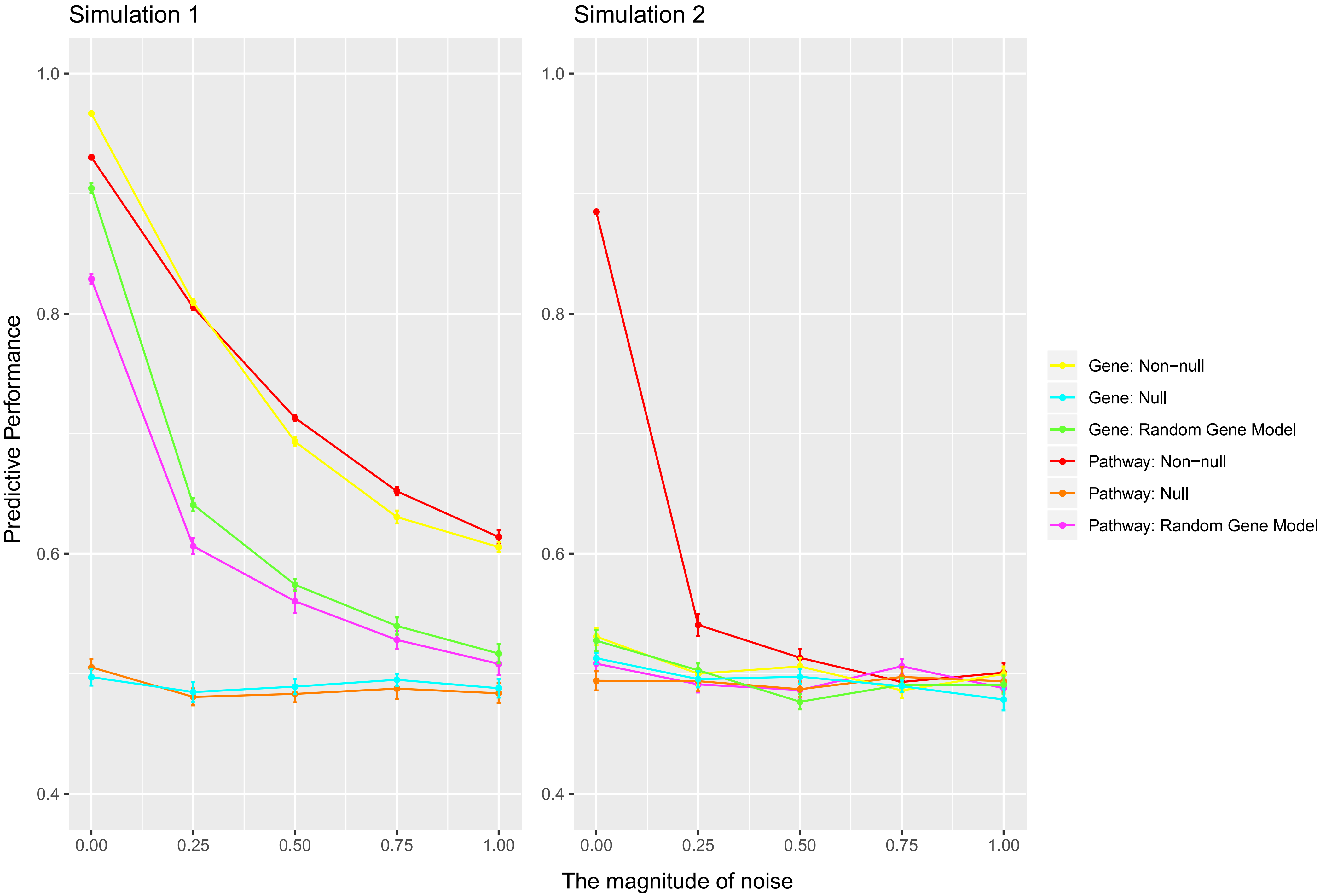

Figure S101: No.35 HALLMARK_GLYCOLYSIS (size=200, absolute mean correlation=0.18)

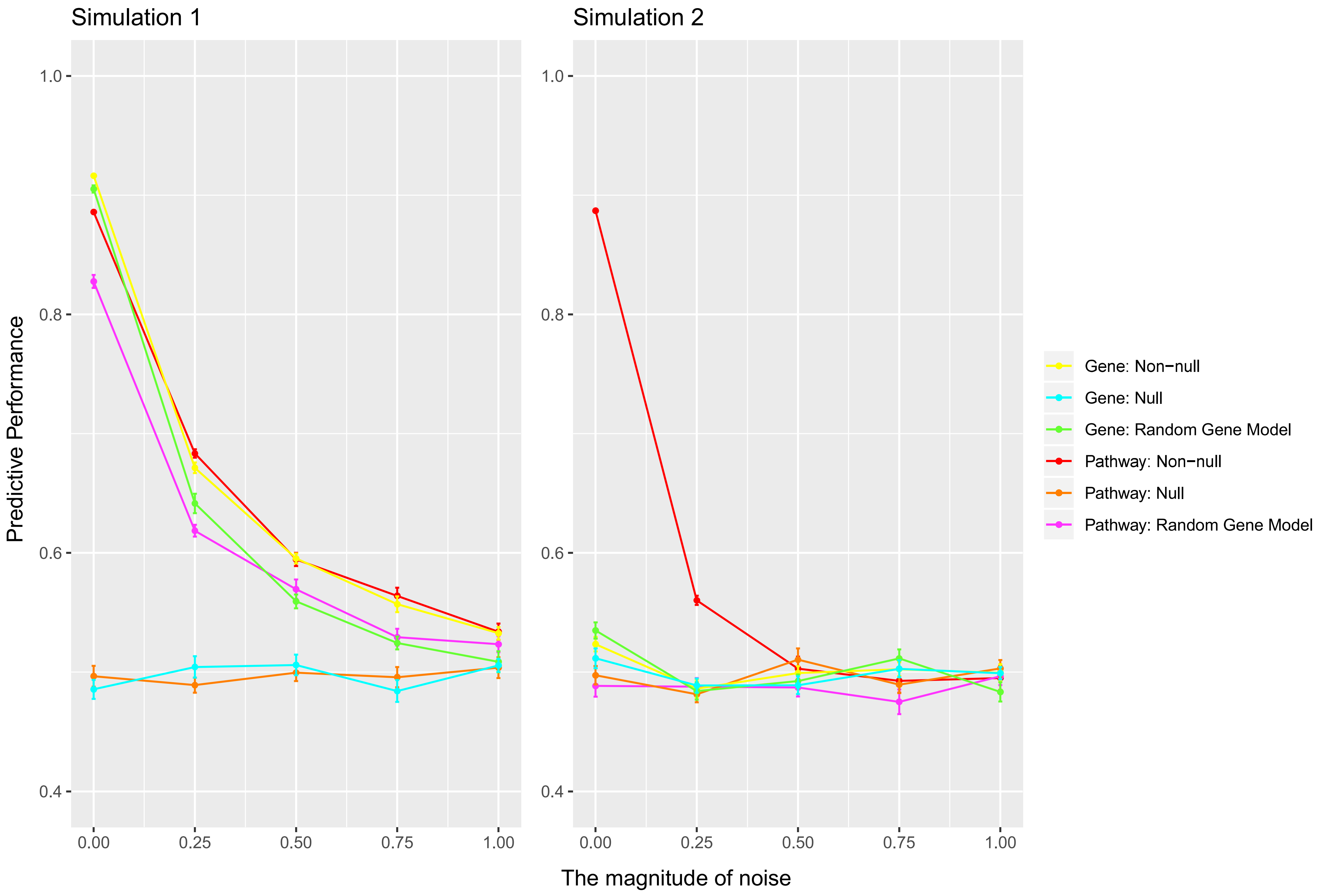

gure S102: No.36 HALLMARK_REACTIVE_OXIGEN_SPECIES_PATHWAY (size=49, absolute mean correlation=

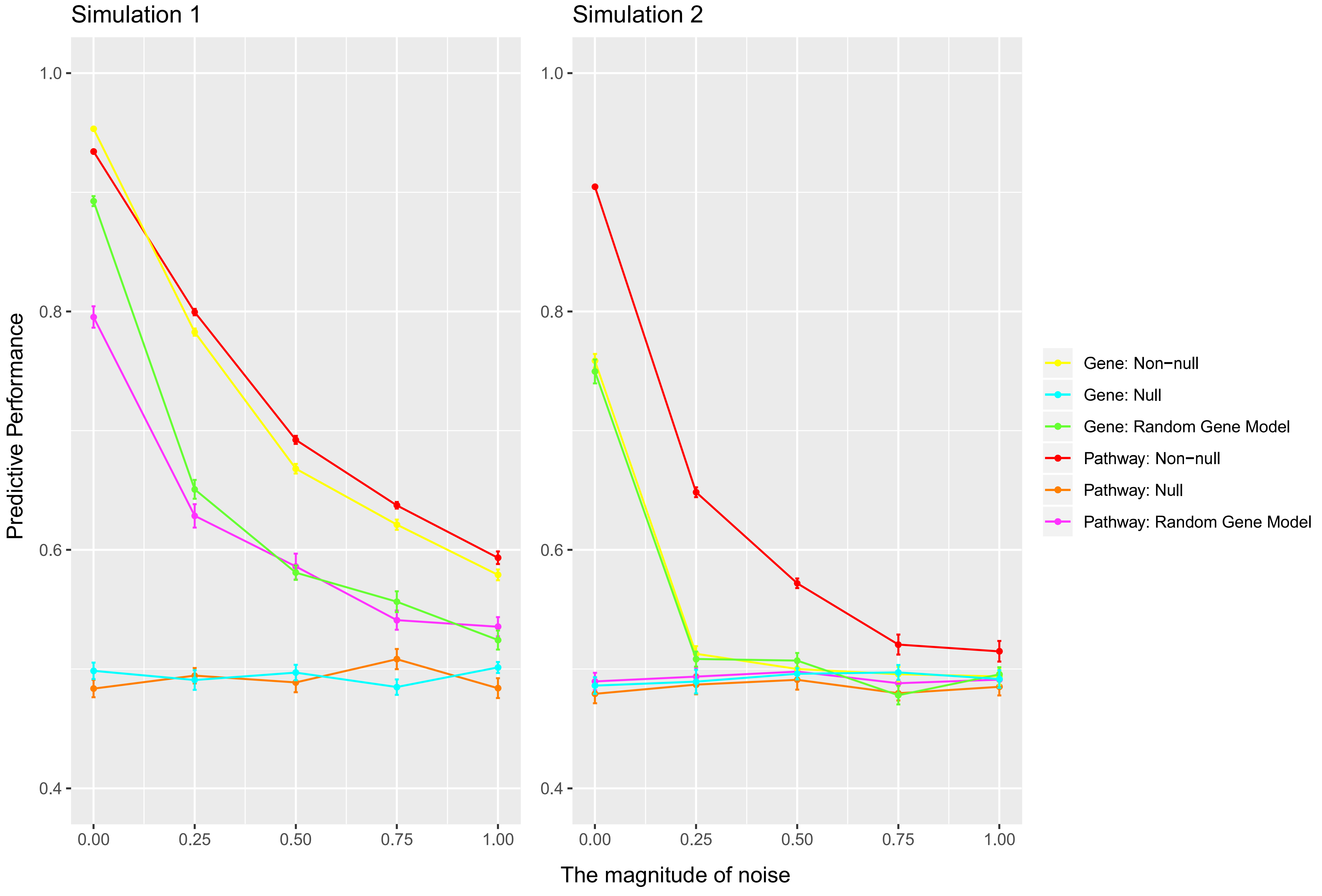

Figure S103: No.37 HALLMARK_P53_PATHWAY (size=200, absolute mean correlation=0.19)

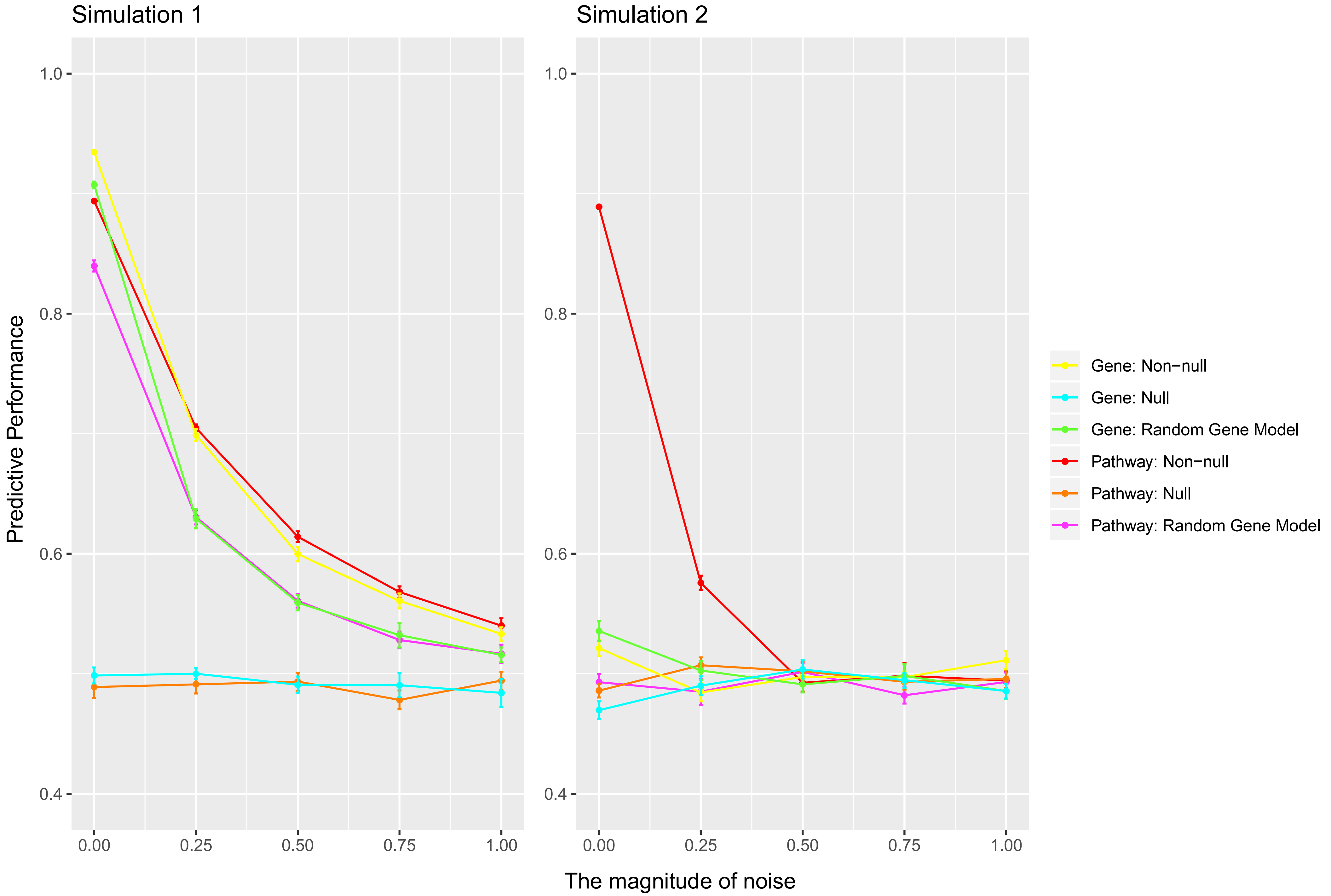

Figure S104: No.38 HALLMARK_UV_RESPONSE_UP (size=158, absolute mean correlation=0.18)

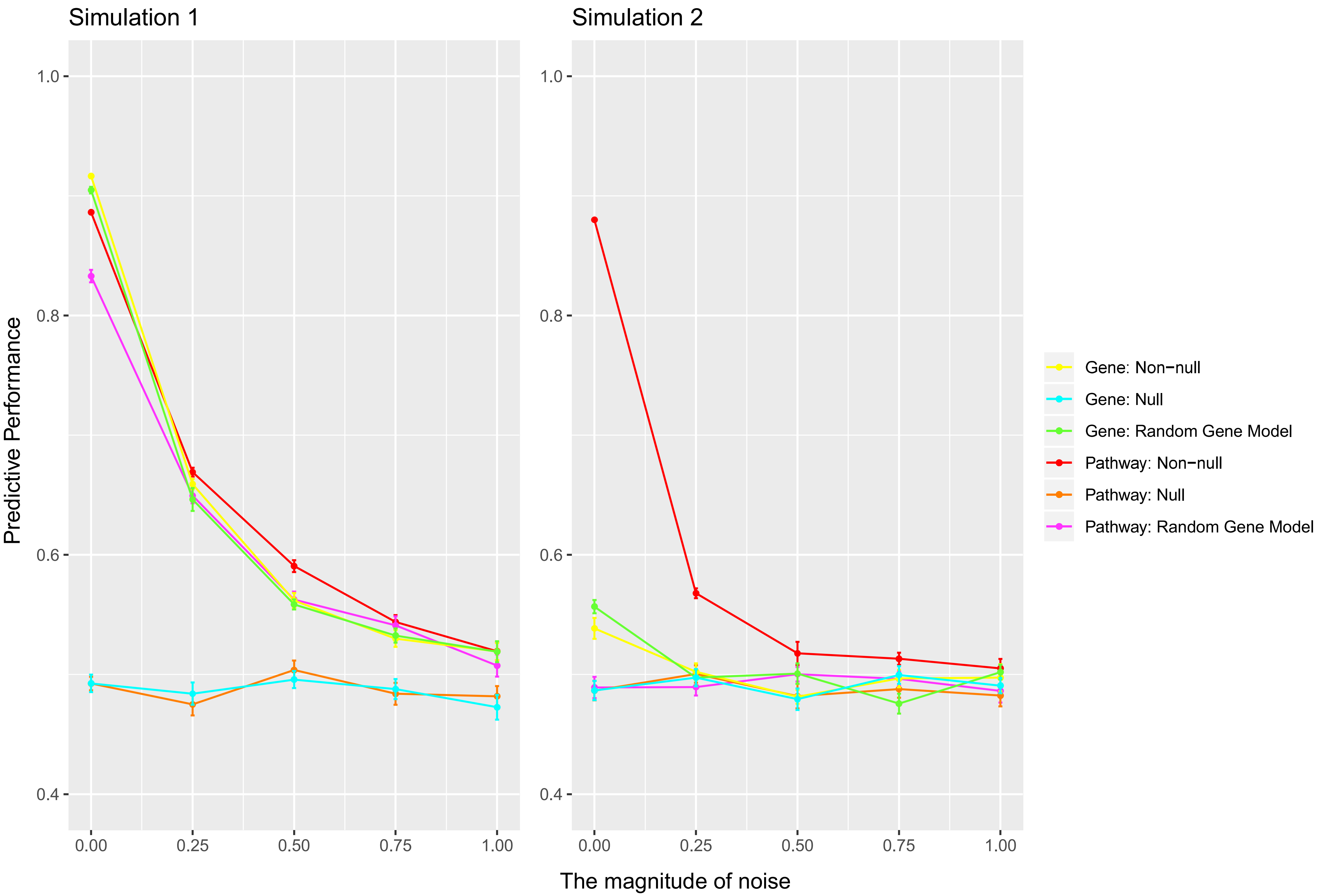

Figure S105: No.39 HALLMARK_UV_RESPONSE_DN (size=144, absolute mean correlation=0.20)

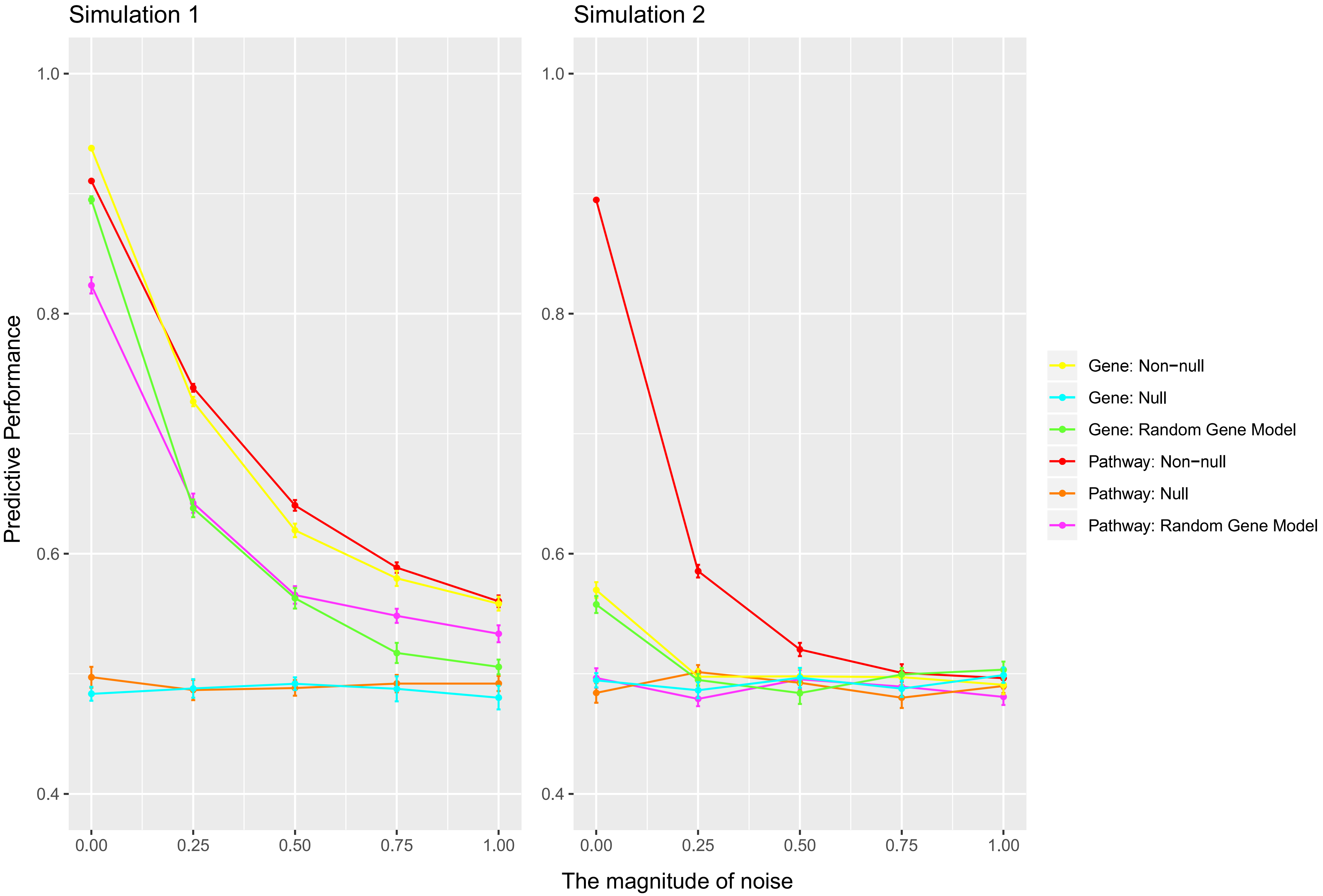

Figure S106: No.40 HALLMARK_ANGIOGENESIS (size=36, absolute mean correlation=0.24)

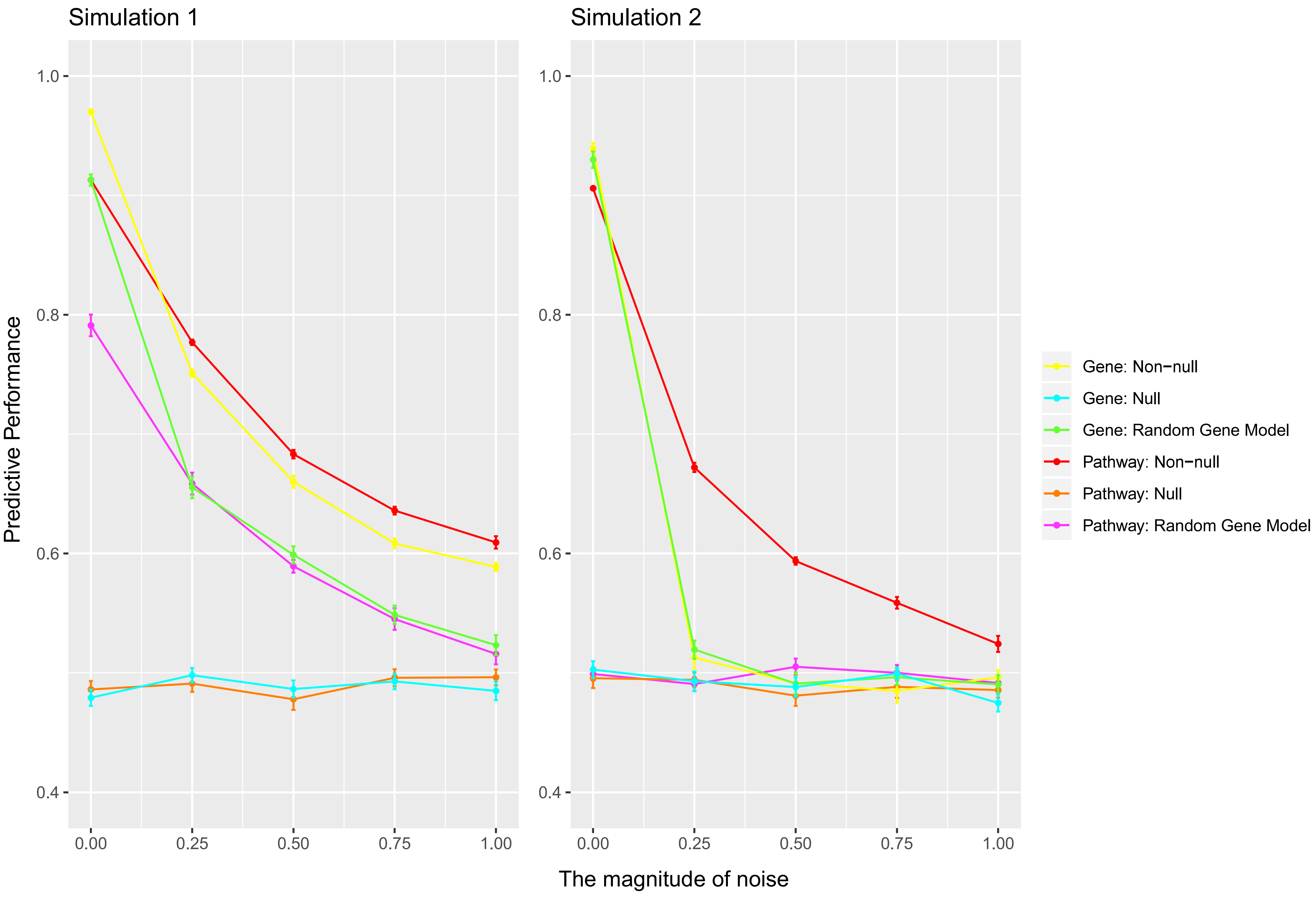

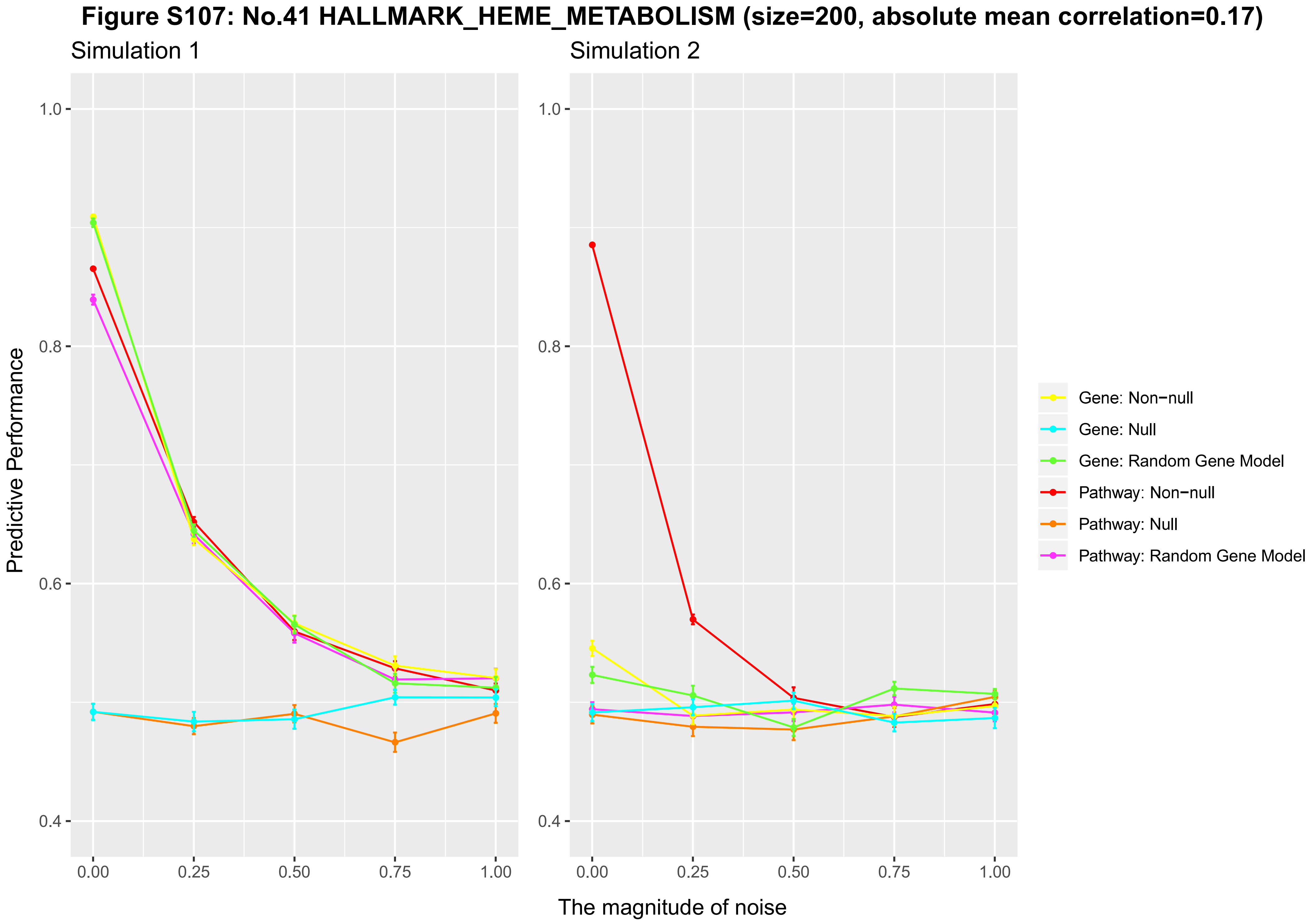
Figure S107: No.41 HALLMARK_HEME_METABOLISM (size=200, absolute mean correlation=0.17)
Simulation 1
Simulation 2
Predictive Performance
The magnitude of noise
1.0
0.8
0.6
0.4
0.00
0.25
0.50
0.75
1.00
Gene: Non−null
Gene: Null
Gene: Random Gene Model
Pathway: Non−null
Pathway: Null
Pathway: Random Gene Model

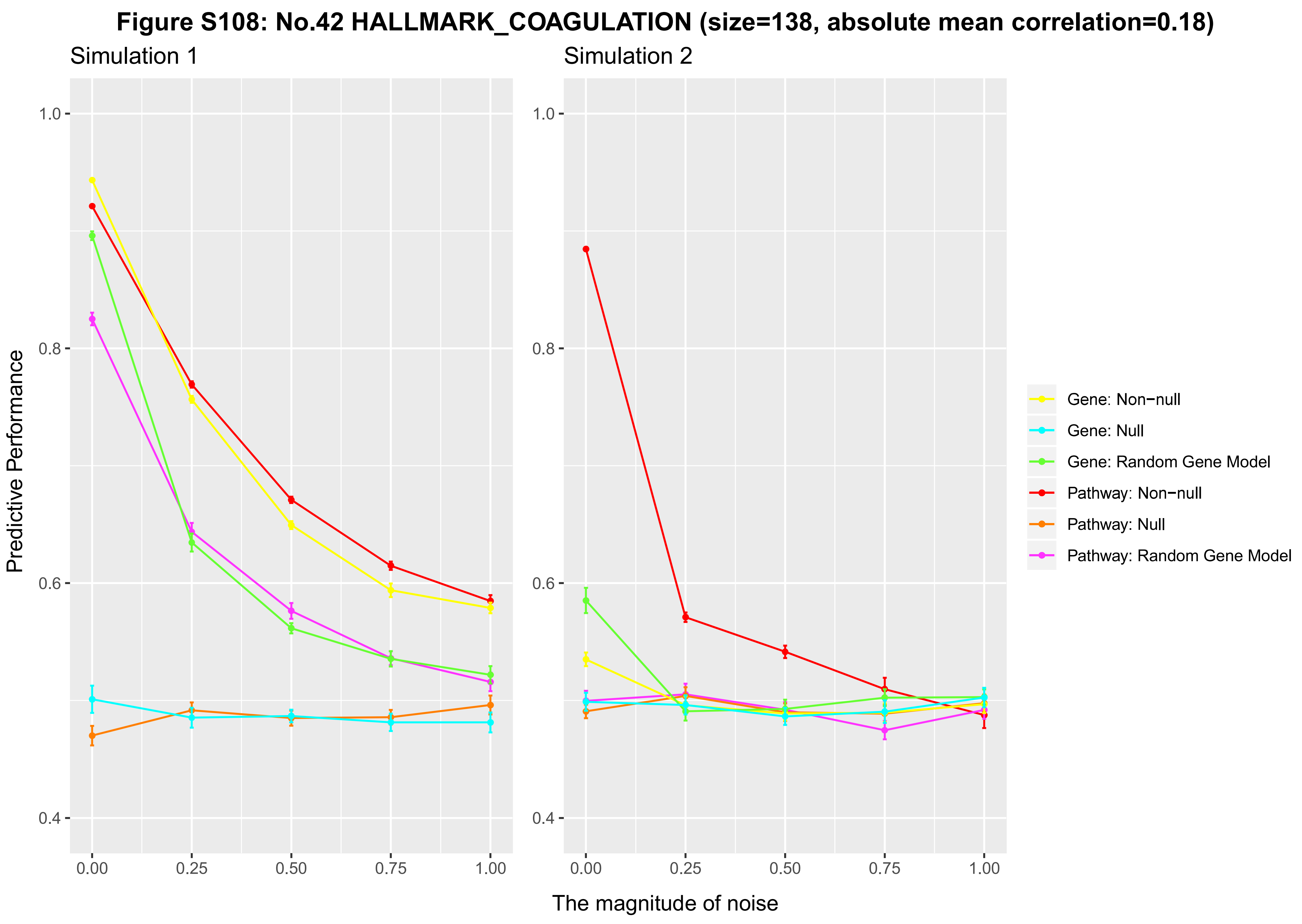

Figure S108: No.42 HALLMARK_COAGULATION (size=138, absolute mean correlation=0.18)
Simulation 1
Simulation 2
Predictive Performance
1.0
0.8
0.6
0.4
0.00
0.25
0.50
0.75
1.00
The magnitude of noise
Gene: Non−null
Gene: Null
Gene: Random Gene Model
Pathway: Non−null
Pathway: Null
Pathway: Random Gene Model

Figure S109: No.43 HALLMARK_IL2_STAT5_SIGNALING (size=200, absolute mean correlation=0.21)

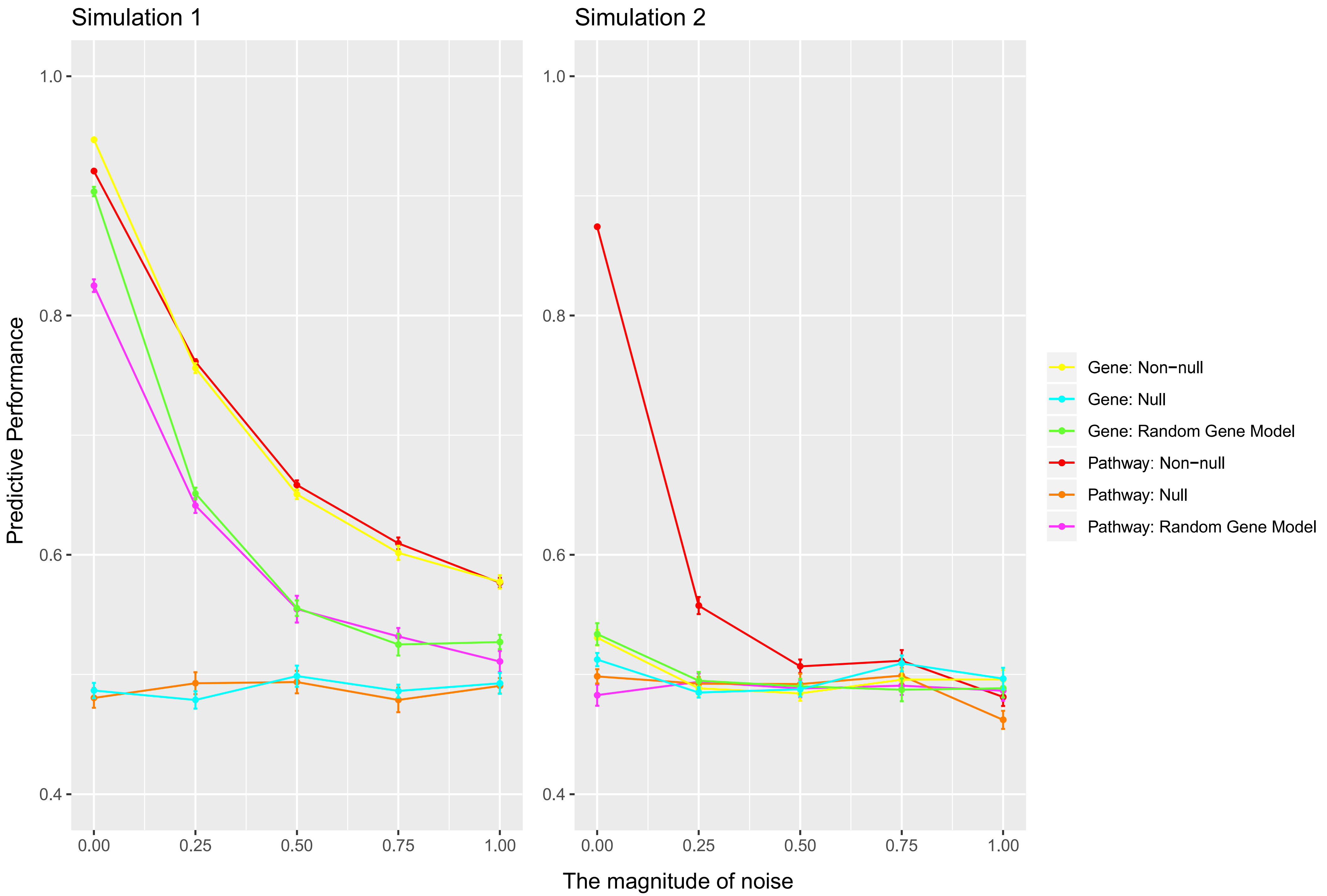

Figure S110: No.44 HALLMARK_BILE_ACID_METABOLISM (size=112, absolute mean correlation=0.16)

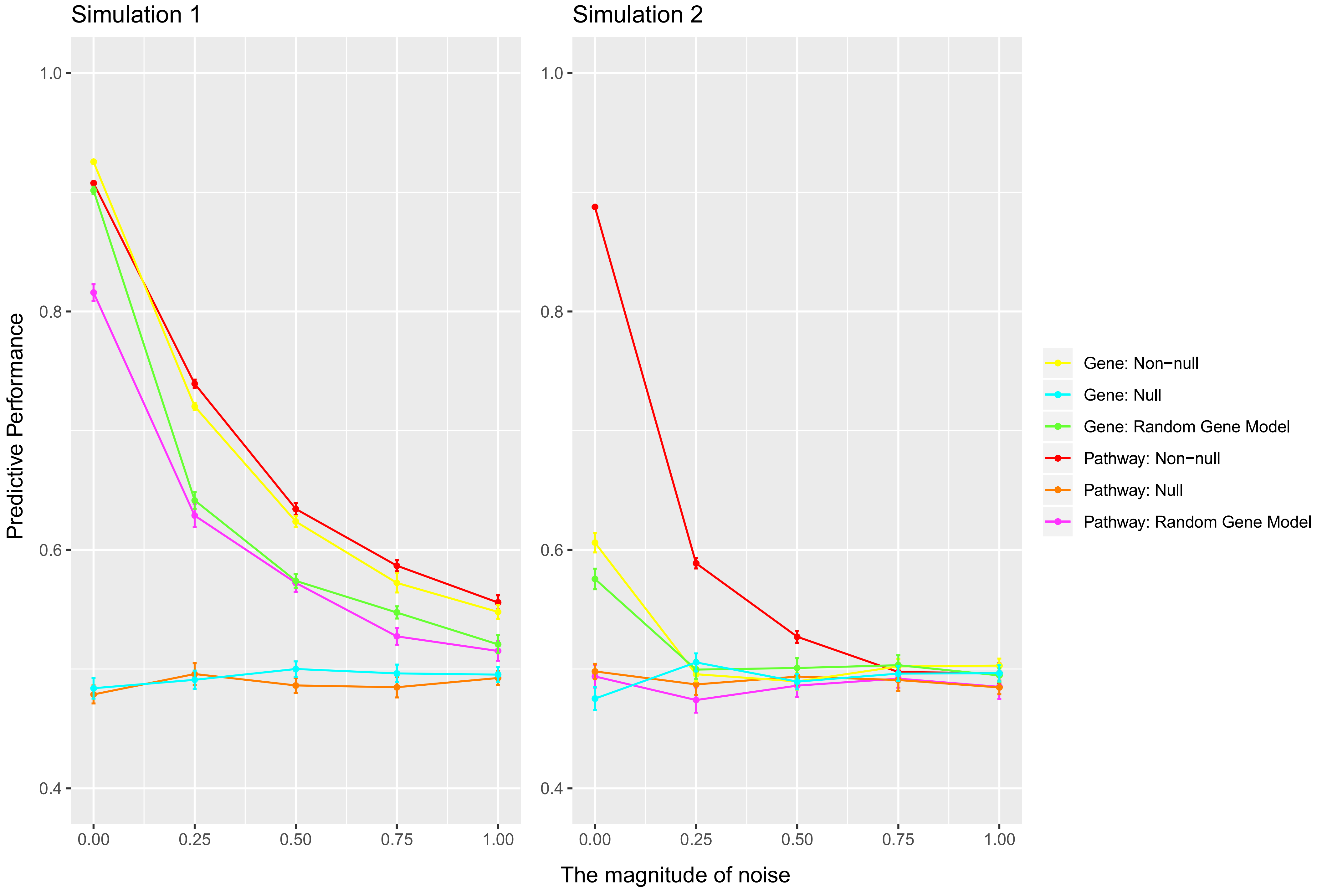

Figure S111: No.45 HALLMARK_PEROXISOME (size=104, absolute mean correlation=0.18)

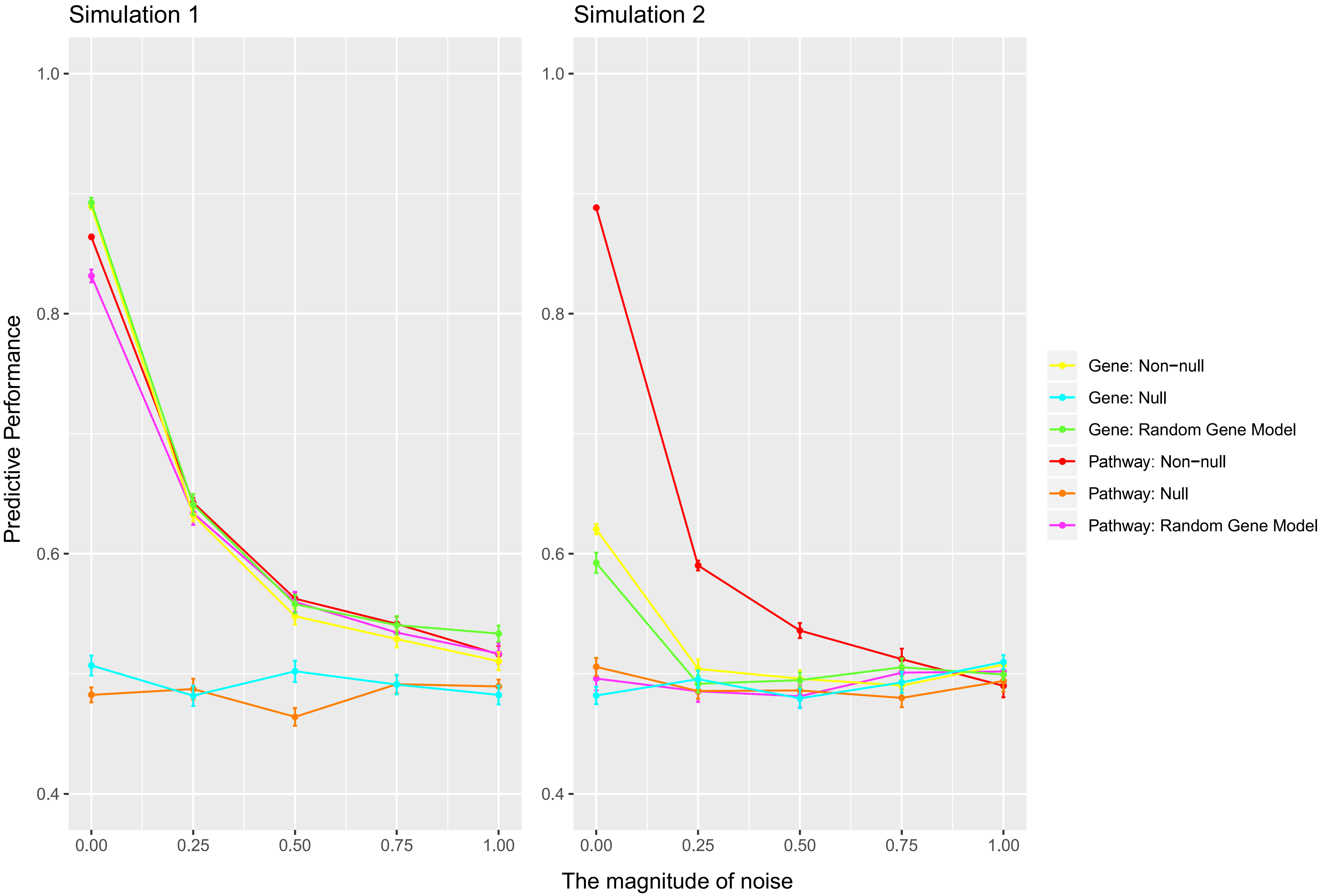

Figure S112: No.46 HALLMARK_ALLOGRAFT_REJECTION (size=200, absolute mean correlation=0.27)

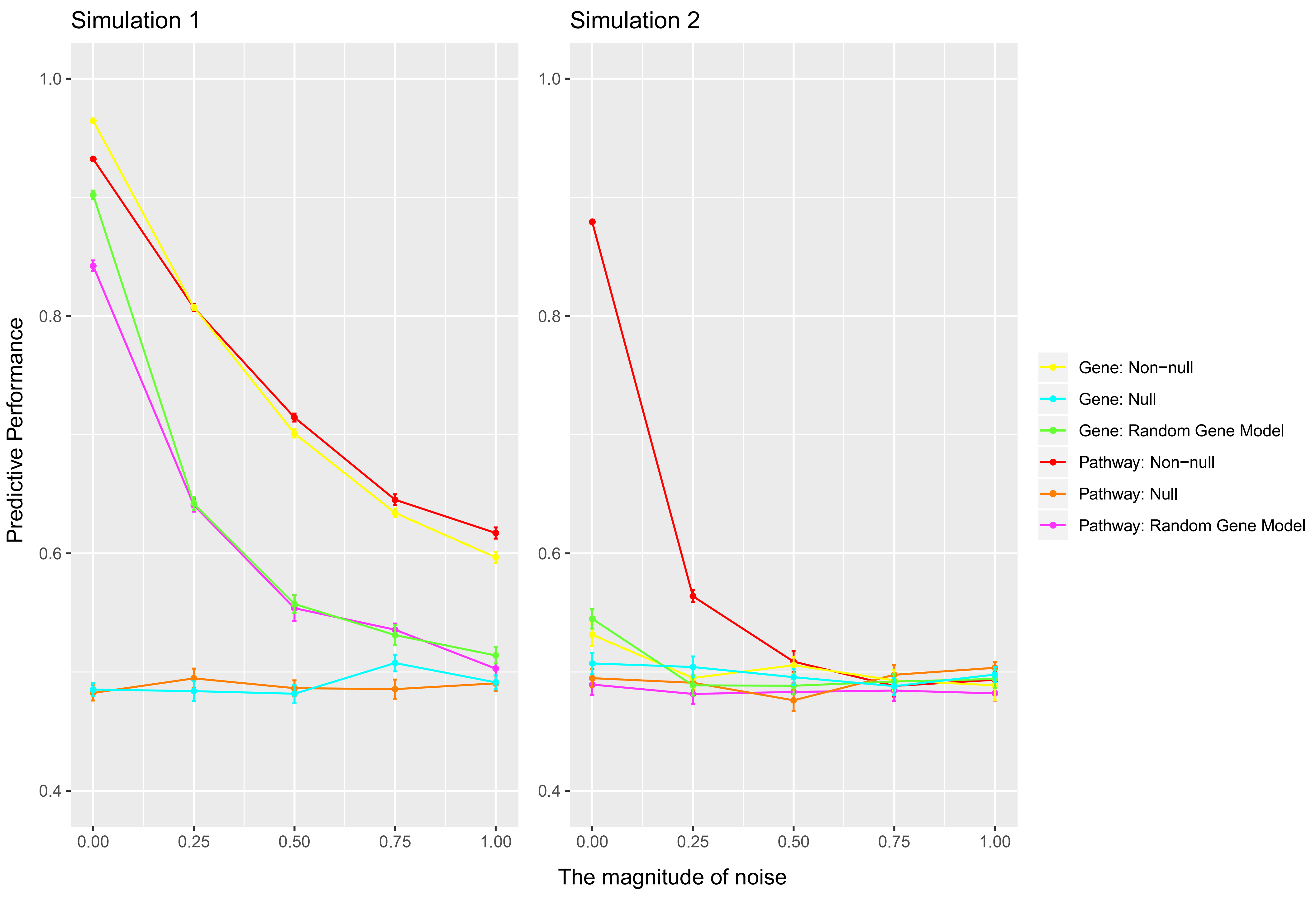

Figure S113: No.47 HALLMARK_SPERMATOGENESIS (size=135, absolute mean correlation=0.15)

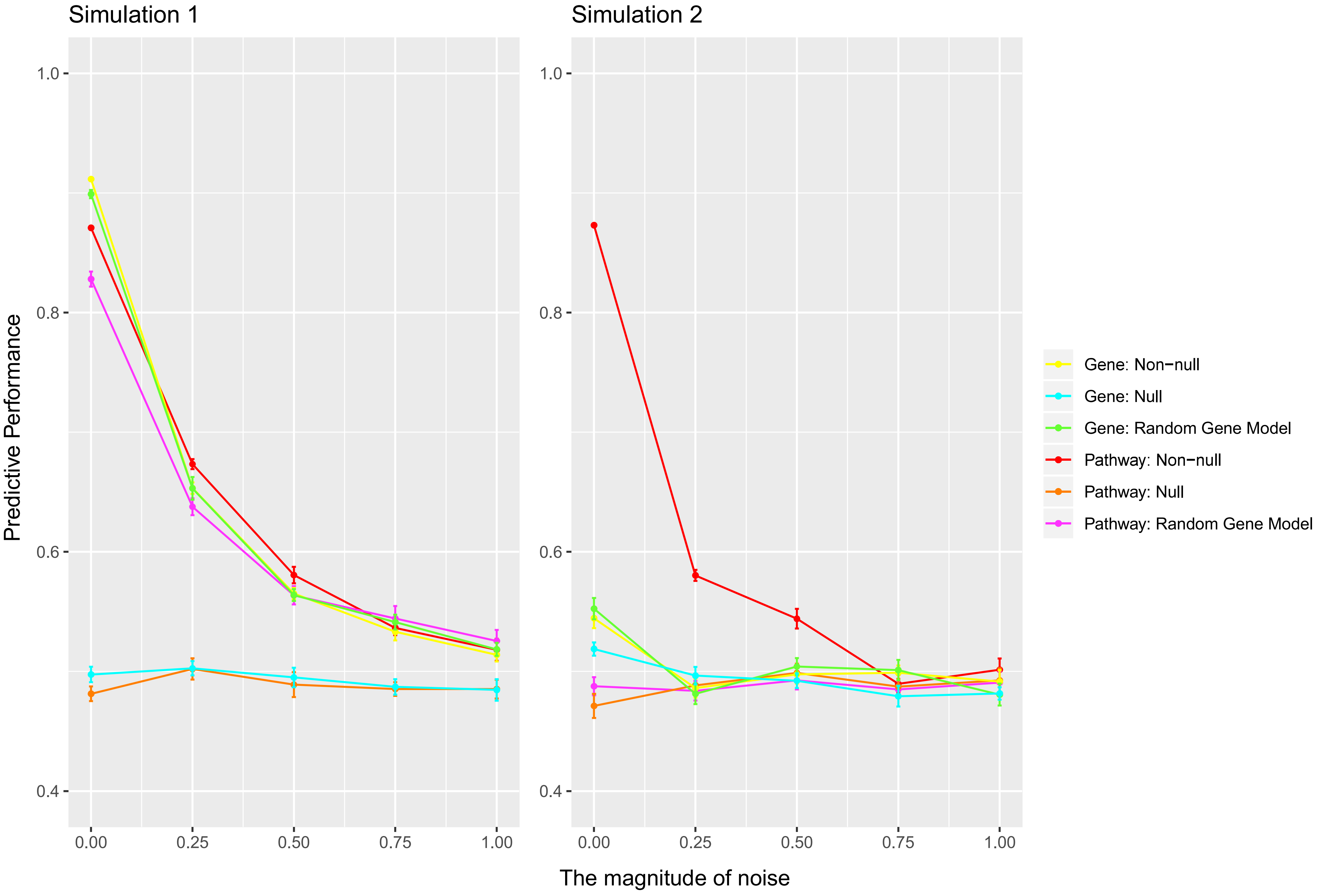

Figure S114: No.48 HALLMARK_KRAS_SIGNALING_UP (size=200, absolute mean correlation=0.20)

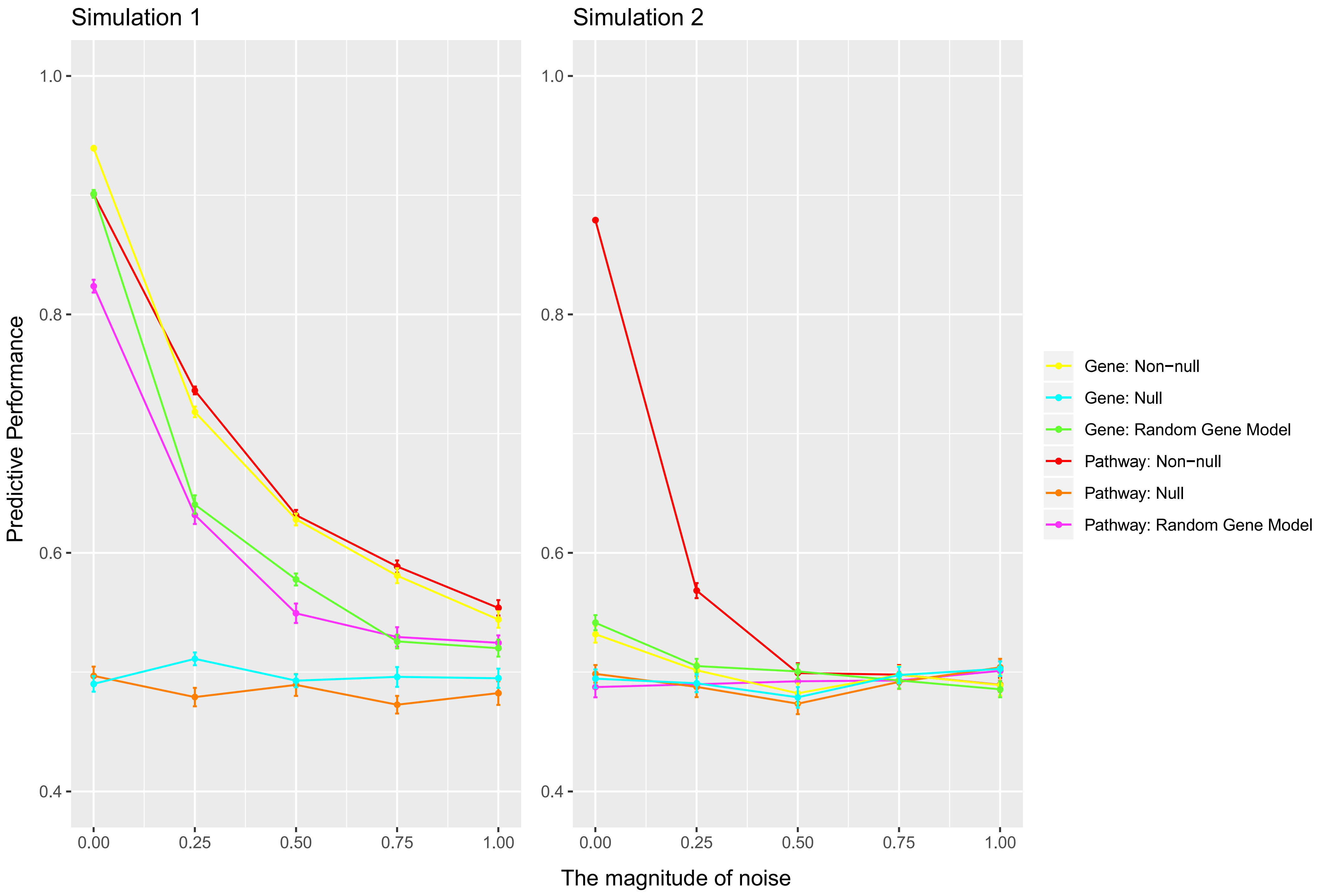

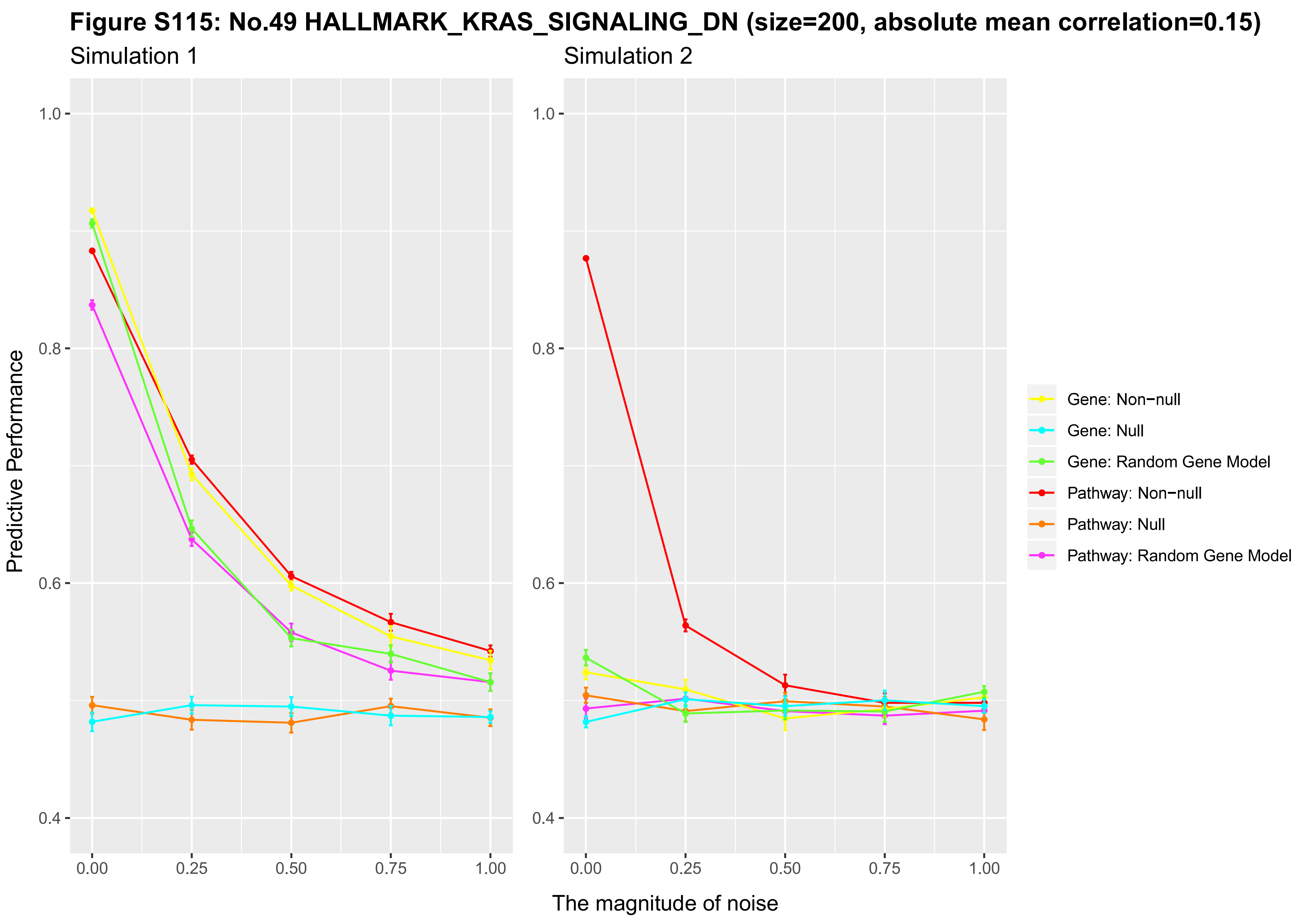
Figure S115: No.49 HALLMARK_KRAS_SIGNALING_DN (size=200, absolute mean correlation=0.15)
Simulation 1
Simulation 2
Predictive Performance
1.0
0.8
0.6
0.4
0.00
0.25
0.50
0.75
1.00
The magnitude of noise
Gene: Non−null
Gene: Null
Gene: Random Gene Model
Pathway: Non−null
Pathway: Null
Pathway: Random Gene Model

Figure S116: No.50 HALLMARK_PANCREAS_BETA_CELLS (size=40, absolute mean correlation=0.19)

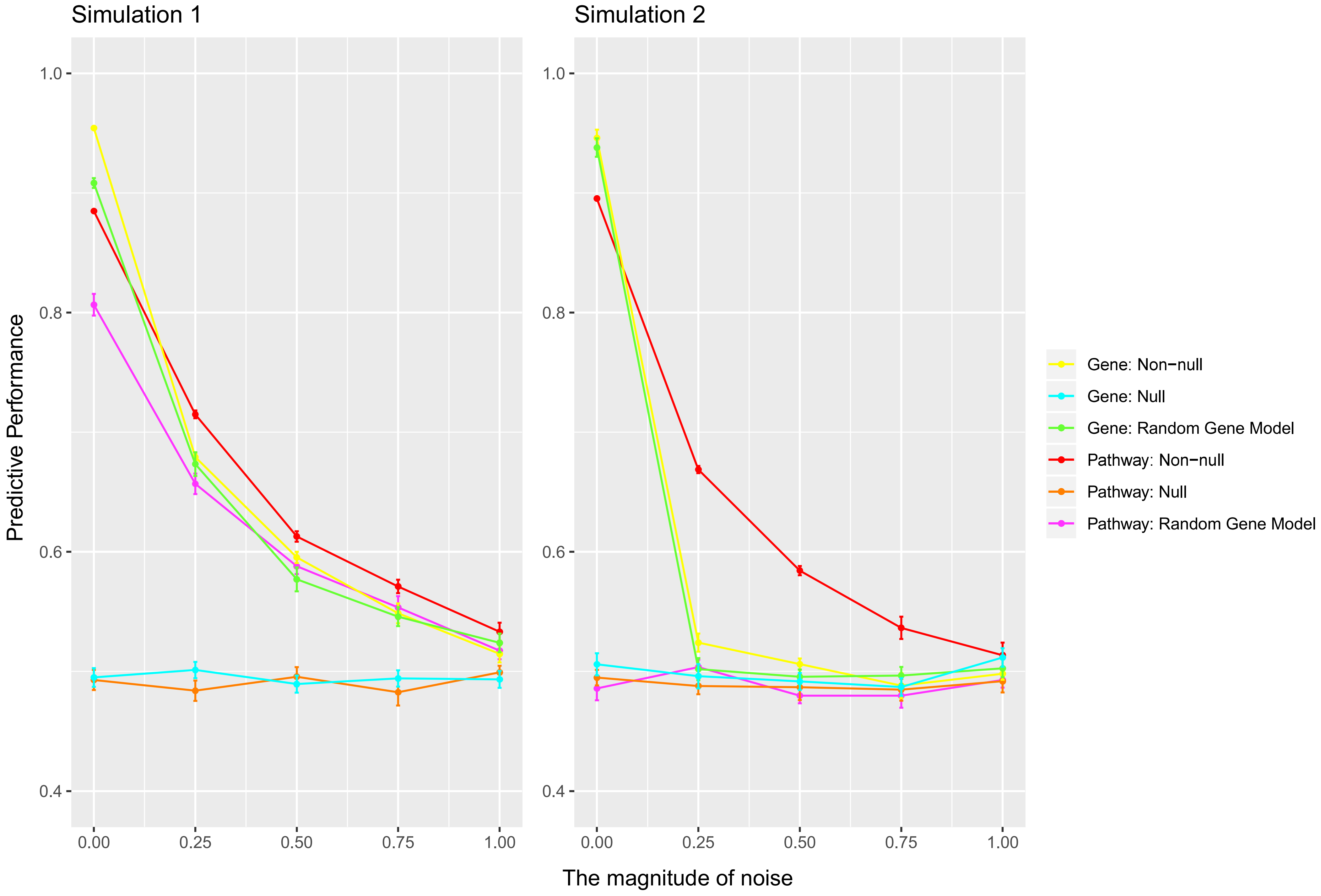

Supplement: Supplementary file 2 — Additional file 2. Supplementary results of associating different pathways in the simulation studies for LGG cohort. [file 12859_2020_3423_MOESM2_ESM.pdf]
